# Measuring Under-Five Mortality: Validation of New Low-Cost Methods

**DOI:** 10.1371/journal.pmed.1000253

**Published:** 2010-04-13

**Authors:** Julie Knoll Rajaratnam, Linda N. Tran, Alan D. Lopez, Christopher J. L. Murray

**Affiliations:** 1 Institute for Health Metrics and Evaluation, University of Washington, Seattle, Washington, United States of America; 2 School of Population Health, University of Queensland, Brisbane, Australia; Harvard Initiative for Global Health, United States of America

## Abstract

**Background:**

There has been increasing interest in measuring under-five mortality as a health indicator and as a critical measure of human development. In countries with complete vital registration systems that capture all births and deaths, under-five mortality can be directly calculated. In the absence of a complete vital registration system, however, child mortality must be estimated using surveys that ask women to report the births and deaths of their children. Two survey methods exist for capturing this information: summary birth histories and complete birth histories. A summary birth history requires a minimum of only two questions: how many live births has each mother had and how many of them have survived. Indirect methods are then applied using the information from these two questions and the age of the mother to estimate under-five mortality going back in time prior to the survey. Estimates generated from complete birth histories are viewed as the most accurate when surveys are required to estimate under-five mortality, especially for the most recent time periods. However, it is much more costly and labor intensive to collect these detailed data, especially for the purpose of generating small area estimates. As a result, there is a demand for improvement of the methods employing summary birth history data to produce more accurate as well as subnational estimates of child mortality.

**Methods and Findings:**

We used data from 166 Demographic and Health Surveys (DHS) to develop new empirically based methods of estimating under-five mortality using children ever born and children dead data. We then validated them using both in- and out-of-sample analyses. We developed a range of methods on the basis of three dimensions of the problem: (1) approximating the average length of exposure to mortality from a mother's set of children using either maternal age or time since first birth; (2) using cohort and period measures of the fraction of children ever born that are dead; and (3) capturing country and regional variation in the age pattern of fertility and mortality. We focused on improving estimates in the most recent time periods prior to a survey where the traditional indirect methods fail. In addition, all of our methods incorporated uncertainty. Validated against under-five estimates generated from complete birth histories, our methods outperformed the standard indirect method by an average of 43.7% (95% confidence interval [CI] 41.2–45.2). In the 5 y prior to the survey, the new methods resulted in a 53.3% (95% CI 51.3–55.2) improvement. To illustrate the value of this method for local area estimation, we applied our new methods to an analysis of summary birth histories in the 1990, 2000, and 2005 Mexican censuses, generating subnational estimates of under-five mortality for each of 233 jurisdictions.

**Conclusions:**

The new methods significantly improve the estimation of under-five mortality using summary birth history data. In areas without vital registration data, summary birth histories can provide accurate estimates of child mortality. Because only two questions are required of a female respondent to generate these data, they can easily be included in existing survey programs as well as routine censuses of the population. With the wider application of these methods to census data, countries now have the means to generate estimates for subnational areas and population subgroups, important for measuring and addressing health inequalities and developing local policy to improve child survival.

*Please see later in the article for the Editors' Summary*

## Abstract 2

n/a

## Editors' Summary

**Background:**

Every year, more than 8 million children die before their fifth birthdays. Most of these deaths occur in developing countries, and most are the result of diseases or combinations of diseases that could have been prevented or treated. Measles, for example, is a major killer in low-income countries and undernutrition contributes to one-third of childhood deaths. Faced with this largely avoidable loss of young lives, in 1990, the United Nations' World Summit for Children pledged to improve the survival of children. Later, in 2000, world leaders set a target of reducing child mortality to one-third of its 1990 level by 2015 as Millennium Development Goal 4. This goal, together with seven others, is designed to alleviate extreme poverty by 2015. In 2006, for the first time since mortality records began, annual deaths among children under five fell below 10 million as a result of public-health programs such as the Measles Initiative, which has reduced global measles mortality by more than two-thirds by vaccinating 500 million children, and the Nothing but Nets campaign, which distributed insecticide-treated antimalaria nets in Africa.

**Why Was This Study Done?:**

Although global under-five mortality is declining, it is unlikely that Millennium Development Goal 4 will be reached by 2015. Indeed, in some countries, little or no progress is being made toward this goal. To improve progress and to monitor the effects of public-health interventions, accurate, up-to-date estimates of national and subnational child mortality rates are essential. In developed countries, vital registration systems—records of all births and deaths—mean that under-five mortality rates can be directly calculated. But many developing countries lack vital registration systems, and child mortality has to be estimated using data collected in surveys. In “complete birth history” surveys, mothers are asked numerous questions about each living child and each dead child. Such surveys can be used to estimate under-five mortality accurately for recent time periods but they are expensive and time-consuming. By contrast, in “summary birth history” surveys, each mother is simply asked how many live births she had and how many of her children have survived. Under-five mortality can be indirectly calculated from this information and the age of the mother, but the current methods for making this calculation cannot provide reliable estimates of under-five mortality more recently than 3 years before the survey. In this study, therefore, the researchers develop methods for estimating more recent under-five mortality rates from summary birth histories.

**What Did the Researchers Do and Find?:**

The researchers used data about all children born and dead children extracted from 169 Demographic and Health Surveys (DHS; a project started in 1984 to help developing countries collect data on health and population trends) covering 70 countries to develop four new methods to estimate under-five mortality. They tested these new methods and a method that combined all four approaches by comparing the estimates of under-five mortality provided by these methods and the standard indirect method to the estimates obtained from an analysis of the complete birth data in the DHS. The new methods all outperformed the standard indirect method, particularly for the most recent 5 years. The researchers also used their new methods to generate estimates of under-five mortality for each of the 233 jurisdictions in Mexico from summary birth histories collected in the 1990, 2000, and 2005 Mexico censuses. The overall trends of these subnational estimates, they report, mirrored those obtained from vital registration data.

**What Do These Findings Mean?:**

These findings suggest that application of the new methods developed by the researchers could significantly improve the accuracy of estimates of under-five mortality based on summary birth history data. The researchers warn that although their methods can provide accurate estimates of recent under-five mortality, they might not capture rapid fluctuations in mortality such as those that occur during wars. However, they suggest, the two questions needed to generate the data required to apply these new methods could easily be included in existing survey programs and in routine censuses. Consequently, systematic application of the methods proposed in this study should provide policy makers with the information about levels, recent trends, and inequalities in child mortality that they need to accelerate efforts to reduce the global toll of childhood deaths.

**Additional Information:**

Please access these Web sites via the online version of this summary at http://dx.doi.org/10.1371/journal.pmed.1000253.

- This study and two related *PLoS Medicine* Research Articles by Obermeyer et al and by Murray et al are further discussed in a *PLoS Medicine* Perspective by Mathers and Boerma
- The United Nations Childrens Fund (UNICEF) works for children's rights, survival, development and protection around the world; it provides information on Millennium Development Goal 4 and its Childinfo website provides detailed statistics about child survival and health (some information in several languages)
- Further information about the Millennium Development Goals is available
- The World Health Organization also has information about Millennium Development Goal 4 and provides estimates of child mortality rates
- Information is also available about the Demographic and Health Surveys

## Introduction

Over the past few decades, and particularly since the World Summit for Children in 1990 [1], there has been growing interest in measuring child mortality, both as a health indicator and, increasingly, as a fundamental measure of human development [2]. This interest has brought renewed attention to the challenge of improving child survival, including a focus on understanding why some countries are making progress and others are not [3]–[9]. Some authors have suggested that declines in child mortality can be at least partially attributed to the improved measurement of child mortality, which facilitates the benchmarking of progress with intervention strategies and ensures a measure of accountability [10]. Increased policy discussion of investment in child health is leading to calls for more timely and more local measurements of child mortality [11]. Nonetheless, despite considerable efforts, our knowledge of trends in child mortality over recent periods, where the impact of intervention strategies is of most interest for policy makers, is weak for many low-income countries [2].

While health interventions to improve child survival are focused on specific diseases or groups of diseases or conditions, there is much public health utility in understanding how they are or are not affecting overall levels of child survival. For example, whereas a specific intervention may prevent deaths because of one particular cause, vulnerable children are often susceptible to other competing causes of death [12],[13]. Improving child survival requires a broad examination of child mortality trends.

A vital registration system that captures all births and deaths is the optimal way to monitor trends in child mortality; however, very few developing countries have complete vital registration systems [14]. Child mortality is generally measured using surveys that ask women to report the births and deaths of their children. Several survey methods exist for capturing this information: complete birth histories capture detailed information on every child alive or dead, including date of birth and date of death; truncated birth histories are the same as complete birth histories but ask only about births and deaths within a specified period of time; summary birth histories ask only how many live births each mother has ever had and how many of them have survived; and questions that ask about the survival of a respondent's last live birth. The two forms that dominate the data landscape are complete birth histories and summary birth histories.

Complete birth histories are the preferred method and have been widely used in large global survey programs, including the World Fertility Surveys (1974–1983), ongoing Demographic and Health Surveys (DHS) (1985–present), and various national survey programs [15]–[17]. Complete birth histories are costly and labor intensive to administer; in the DHS, a complete birth history includes 11 questions for each living child and nine questions for each dead child. The average number of children reported varies by survey (see Table S1), ranging from 1.52 in the 2005 Armenia survey to 5.09 in the 1990 Jordan survey. Surveys conducted in sub-Saharan Africa have the highest number of children reported, whereas surveys from Asia yield fewer average children per respondent. On average, each mother reports 3.65 children; this implies an average of 33–40 questions to complete the birth history [18]. As a consequence, most surveys including complete birth histories have relatively small sample sizes and are designed primarily to generate nationally representative estimates. With increasing concern about equity in child survival, it is arguably as important to be able to measure and monitor child mortality at the subnational level. Complete birth histories are clearly inappropriate for this purpose. There are other drawbacks to complete birth histories as well. The demand upon the respondent to recall dates of births and deaths, especially in high-fertility settings, risks recall bias. In addition, particularly when the overall survey instrument is quite long such as in the DHS, interviewers may misdate events to avoid having to ask further questions about a particular child [19],[20].

In contrast, a minimum of only two items are required for a summary birth history: the number of children ever born and the number who have died (and potentially, the month and year of the mother's first birth). The age of the mother is a standard item of data collected in any census or survey. No time location information is asked of the respondent (note: obviously, minimal time location information is required if questions about time since first birth are asked). Consequently, summary birth history questions have been included in many decennial censuses as well as the UNICEF-sponsored Multiple Indicator Cluster surveys. Census data on summary birth histories, in particular, allow for robust measurements of child mortality for small local areas [21]. It is important to note that summary birth history data are not always captured in the simple two-question format. Often, as in the DHS, a greater number of more specific questions are asked such as the number of surviving sons and daughters living with the respondent, the number not living with the respondent, and the number who have died. From these questions the two key pieces of information on children ever born and children surviving can be computed.

### Historical Development of Methods to Analyze Summary Birth Histories

Methods to estimate child mortality from children ever born and children surviving were first proposed over 50 y ago [22]. Brass, however, laid the foundation for current techniques [23] by observing that there was a strong relationship between the fraction of children ever born who had died for particular age groups of mothers, and population-level child mortality rates. Subsequent refinements of the method were proposed by a number of others [24]–[28], including a method for localizing in time the estimated rates of mortality by Feeney [26]. These methods have been widely applied to estimate the relationship between the fraction of children ever born that have died, tabulated by maternal age, and under-five mortality. Alternative analytical strategies that tabulate children ever born and children dead by years since marriage and time since first birth have been proposed [29],[30], but not widely implemented.

There are three main limitations of existing methods to analyze summary birth histories. First, the responses of women aged 15–19 y and 20–24 y are used to generate estimates of under-five mortality for the most recent time period. Children of these younger mothers typically have higher risks of death compared to children of mothers aged 25–34 y, leading to an overestimation of child mortality for the most recent time period [25],[31]. Because of this bias, the most recent estimates are routinely discarded, meaning that reliable estimates of child mortality can be generated for periods only as recent as 3–6 y prior to a survey. This situation has important policy implications for monitoring progress with intervention programs. Second, standard application of these analytic methods does not generate estimates of uncertainty in the measures of child mortality, severely limiting their capacity to identify statistically significant changes in mortality, particularly from intervention programs. Third, while these methods have a strong theoretical foundation, they have not been validated against estimates from vital registration data or complete birth histories in a wide set of countries.

Overall, this study aimed to improve the use of low-cost summary birth history data to reliably measure changes in child mortality levels. Using available empirical datasets, we developed and validated new methods for analyzing survey information on children ever born and children who have died.

## Methods

### Data

We analyzed all 169 nationally representative standard DHS from 70 countries with complete and summary birth history data that were available in the public domain as of September 1, 2008. On the basis of published critiques, we excluded the 1999 Nigerian survey [32] from the analysis. We also excluded the 1985 El Salvador survey because complete birth histories were not collected. Finally, we excluded the 2005 Moldova survey because it was the only survey in its region and our methods involved establishing and applying regional patterns of fertility and mortality to the data. Table S1 summarizes the 166 surveys included in our analysis.

### Validation Data

We developed and tested our new methods by comparing them to estimates generated from complete birth history data from the DHS. In order to do this, we needed to generate complete birth history–based estimates for any time prior to the survey (i.e., a continuous time series of under-five mortality). First, we created 2-y estimates of under-five mortality as follows. In the manner of Garenne and Gakusi [33], we pooled complete birth history data from all surveys within a country. Pooling the data minimized potential problems with recall bias and the tendency of interviewers to shift births and deaths outside the most recent period of time prior to the survey. We structured the dataset so that the life of each child is broken down into months of observation, with a dummy variable indicating whether the child was alive or died that month. For children who died over age 2 y, the DHS does not collect the age in months that the child died. We assume that deaths over 2 y of age occur at the midpoint of that year of age, i.e., a child who is reported to be 2 y old at death we assume died at 2 y 6 mo of age, and this enables us to locate in time the month of death of each child. In cases where the death occurred in the same year as the survey, we assume that the death occurred at the midpoint between the last birth day and the date of the survey. This structure allowed us to compute monthly age-period probabilities of survival. For each 2-y period with a minimum of 10,000 person months of exposure, we used the monthly probabilities to compute probabilities of survival for the following age groups: 0–1 mo, 1–11 mo, and 1–2 y, 2–3 y, 3–4 y, and 4–5 y. We then derived _5_ *q*_0_, the probability of dying before age 5 y. To create a continuous series from these period measures, we used Loess, a form of local regression, to create a smooth trend of estimates over time [34]. In Loess regression, the α parameter controls the width of the regression window and the weight attached to data points that are farther away from the estimation point; the narrower the window, the more responsive is the regression to local trends. For the validation data, our objective was to create a smooth series of estimates that also reflected short-term fluctuations in child mortality. Therefore, we chose to use small values of α. Since regressions with smaller values of α require more data points, the exact value of α used for each country was determined as a function of the number of data points (α = 10/*n*, where *n* is the number of data points generated from pooled complete birth histories). A country with a single survey yields approximately 12–14 data points from complete birth histories (one estimate every other year going back roughly 24–28 y prior to the survey, depending on the sample size). Countries with multiple surveys yield more data points (for example, the maximum number of data points generated from complete birth histories is 25 in Indonesia with six DHS). The number 10 was chosen in the above formula such that all selected alphas would be lower than 1.0.

We captured sampling and model uncertainty in the validation data using standard simulation methods [35] to generate an uncertainty interval. We assumed the validation estimates to be closest to truth. In the absence of a functioning vital registration system, pooling complete birth histories from nationally representative surveys provides the most complete and accurate picture of child mortality for a given country.

### Methods Overview

**Figure 1:**
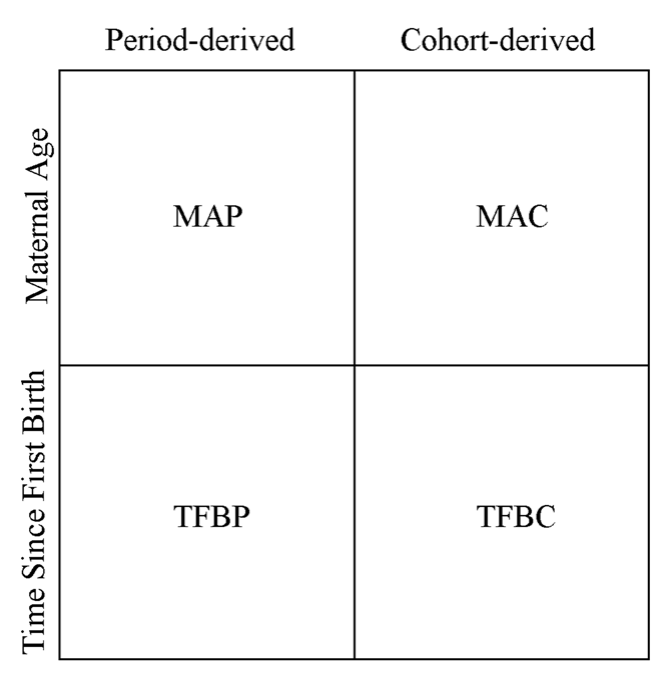
Classification of four new methods to generate estimates of under-five mortality from summary birth history data.

We developed a range of methods that collectively address three methodological issues: (1) determining whether it is better to approximate the average length of exposure to mortality from a mother's set of children using maternal age or time since first birth; (2) determining whether it is better to use cohort or period measures of the fraction of children ever born that are dead; and (3) the need to capture country and regional variation in the age-pattern of fertility and mortality that is not captured by the covariates in the models. Figure 1 illustrates a two-by-two matrix that allows us to evaluate the best set of combinations to address 1 and 2. We develop all four options and a fifth combined method that synthesizes the results from these four. For each of the methods represented in the matrix, we incorporated country and, in the case of the period-derived methods, regional variation. We present estimates and assessments of the performance of each of the five new methods, and we also apply a commonly used version of the standard indirect methods for comparison. In the following sections, we provide detail on each of the five new methods and the standard indirect method for review.

### Standard Indirect Method

Using the schema presented in Figure 1, the standard indirect methods can be classified in the category of maternal age cohort-derived methods. As mentioned earlier, many refinements of Brass' original idea have been developed. One current variant widely applied today is presented in the United Nations' *Manual X* [30] and incorporated in the software package QFIVE produced by the United Nations [36]. This method is based on two regression equations that have been fitted to simulated data, and is largely derived from contributions by Trussell [24] and Feeney [26]. For simplicity, we refer to this method as the standard indirect method throughout this paper. The first regression equation localizes in time the estimates of child mortality obtained from mothers of different ages:
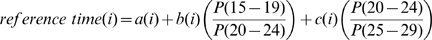
where the reference time for age group *i* is the dependent variable, 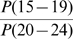 is the parity ratio relating mean children ever born in the age group 15–19 y to the mean children ever born in the age group 20–24 y, 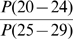 is the parity ratio comparing the 20–24 y to the 25–29 y age groups, and *a*(*i*), *b*(*i*), and *c*(*i*) are the standard model coefficients (listed in Table 48 of *Manual X*).

The second equation estimates a measure of child mortality on the basis of the fraction of children dead out of children ever born (CD/CEB) for a group of mothers:
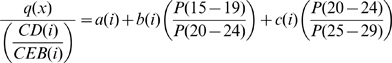
where *q*(*x*) is the probability of dying before age *x*, 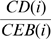 is the ratio of children dead to children ever born for age group *i*, 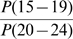, 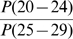 are the same parity ratios as used above, and *a*(*i*), *b*(*i*), and *c*(*i*) are a separate set of coefficients estimated from simulated data. The coefficients are listed in Table 47 of *Manual X*. The fitted equation yields a different measure of child mortality on the basis of the ages of different mothers; for example, _1_ *q*_0_ is predicted for mothers aged 15–19 y, _2_ *q*_0_ for mothers 20–24 y, _3_ *q*_0_ for mothers 25–29 y, _5_ *q*_0_ for mothers 30–34 y, and so on as noted in Table 47 of *Manual X*. As a last step in the standard indirect method, the various measures of child mortality from different age groups of mothers are converted to _5_ *q*_0_ using the Coale and Demeny model life tables. Throughout this paper, we present the standard indirect method using the West model life table, since the West model represents the most general set of mortality patterns and is most often applied when model life tables are used [30].

### Maternal Age Cohort-Derived Method (MAC)

Our MAC method modifies this approach in three ways. First, for all age groups of mothers, we directly estimate _5_ *q*_0_ instead of a different measure of child mortality. In order to obtain uncertainty intervals for these estimates, the model relates the logit of _5_ *q*_0_ to the logit of CD/CEB. We then simply back-transform the predicted logit _5_ *q*_0_ to get predicted _5_ *q*_0_. Second, we include a country random effect in the model for _5_ *q*_0_, which allows us to capture residual systematic variation across countries. Third, we fit our models using empirical datasets rather than simulated data. Because we are using real datasets, we do not use model life tables in any step of the method. The two equations that we have estimated are:
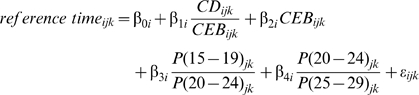
and
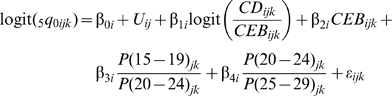
To better reflect the structure of the data and the method, we modify the notation used in the standard indirect approach. The subscripts are indexes for country (*j*), survey (*k*), and age group (*i*). 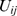 is a country-level random effect. Similar to the standard approach, the models are stratified by age group of mother, meaning one regression is run and coefficients obtained for every age group *i* (15–19 y, 20–24 y, 25–29 y, etc., up to 45–49 y). The first equation is equivalent in concept to Feeney's model for reference time. The observed reference time for estimates from age group *i* is computed from complete birth history data as the average time that deaths to women of age group *i* occurred. To compute this, we subtracted from the time of survey the difference between average age at death of CD and the average age these children would have been if they had survived to the time of the survey. We relate this observed reference time to the ratio of children dead to children ever born (CD/CEB) from the summary birth histories, the average number of children ever born for women of that age group, and the same parity ratios as used in the standard method. This indicates that the expected timing of child deaths that women experience is a function of maternal age, the proportion of children who have died, and the fertility patterns in the population. The predicted reference times for each age group *i* are used to identify the corresponding value of _5_ *q*_0_ from complete birth histories in order to build the second model. This _5_ *q*_0_ is then related to CD/CEB, the country-level random effect 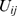, CEB, and the parity ratios. Using the coefficients estimated from the model, we then predict levels of _5_ *q*_0_. Country-level effects are incorporated into the predictions by using the mean and variance of the random effect to generate Bayesian posterior estimates of the country means [37],[38].

### Time since First Birth Cohort-derived Method (TFBC)

We have also developed a similar cohort-derived method where mothers have been grouped in 5-y increments of time since first birth rather than by maternal age. The time localization and _5_ *q*_0_ equations are the same as above except the unit of aggregation for mothers is the time since their first birth. This question has been included in some recent surveys such as the MICS-3.

### Maternal Age Period-Derived Method (MAP)

**Figure 2:**
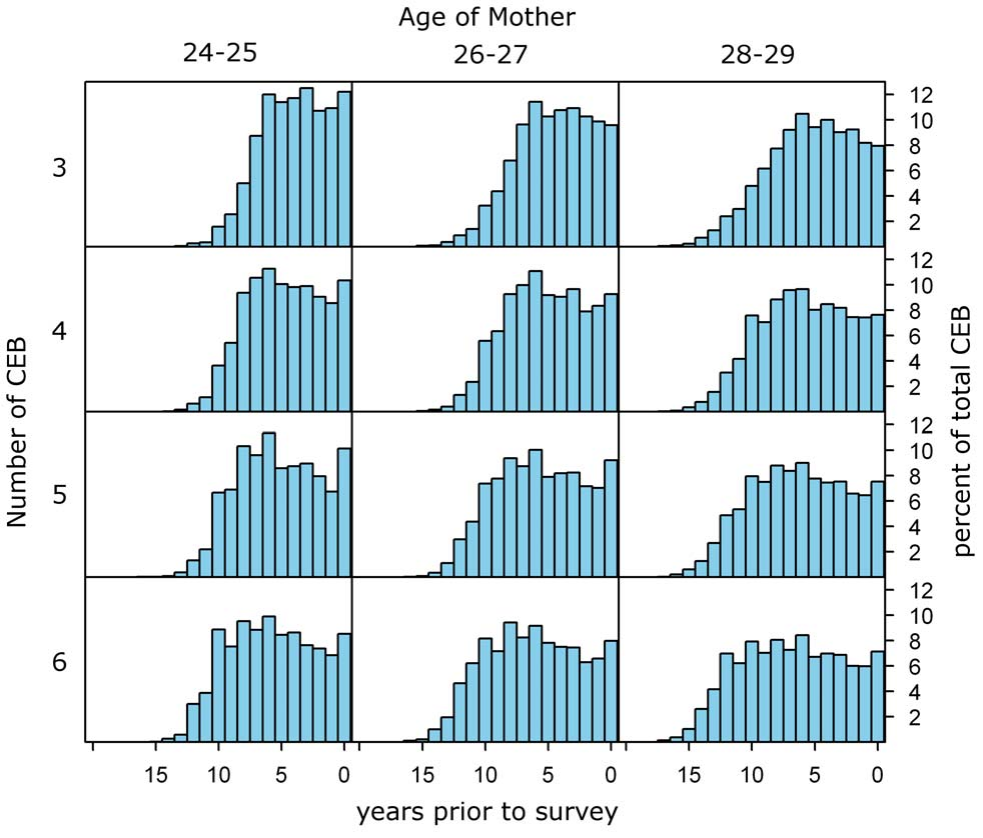
Example distributions of children ever born used to generate the period ratio of CD/CEB in the MAP method (sub-Saharan Africa, West/Central region). Similar distributions over time prior to the survey are generated for each category of single year of age and number of children ever born, for each of the five regions. The same is done for distributions of deaths (not shown in this graph). The distributions from the applicable region are then applied to every mother in the dataset to generate the expected distribution of births and deaths of her children across time prior to the survey. For example, according to the distributions shown above, a 24-y-old woman from this region with three children would be expected to have had 12% of them in the past year, 10.2% in the second year prior to the survey, 10% in the third year prior to the survey, and so on down to small fractions of children expected in the 13th, 14th, and 15th year prior to the survey. For each year prior to the survey, the total number of expected children dead and children ever born is calculated using the expected values across all age groups. The TFBP method applies similar logic, but groups mothers according to the number of years since their first birth rather than by age.

The major problem with the cohort-derived methods is that the most recent estimates of under-five mortality are based only on responses from the youngest mothers. The MAC method eliminates the bias introduced by the higher-than-average levels of mortality affecting children of these mothers. However, there remains the propensity for larger measurement error in the CD/CEB ratio simply because of the small numbers of mothers in this age group. In addition, mortality in recent time periods is occurring in children of older mothers, and the cohort-derived methods do not make use of this information. Another weakness of the cohort-derived methods is that they limit the period of time for which we are able to generate estimates in the past (the MAC method generates an average reference time of 18.1 y prior to the survey for the 45–49 y age group). To address these issues, we estimate a period-based CD/CEB ratio for each year prior to the survey by estimating the time distribution of births and deaths for mothers of a given age who have had a specific number of children. Figure 2 depicts this method graphically, and we describe it below.

**Figure 3:**
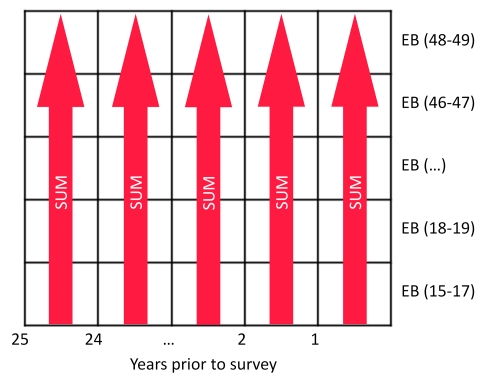
Lexis diagram showing the summation of expected births (EBs) across women of all ages for each year prior to the survey for the MAP method. The same concept is applied to expected deaths for each year prior to the survey and then the two quantities are used to compute the period-derived ratio of CD/CEB.

For each of five regions (Asia; Latin America and the Caribbean; North Africa/Middle East; sub-Saharan Africa, South/East; and sub-Saharan Africa, West/Central), we pool all the responses of mothers of a given age and a given number of children ever born from all surveys in all countries in that region. Using the complete birth histories, we generate the frequency distribution of births and deaths as a function of time prior to the survey. We use age groups of mothers and number of CEB bands, which are fine enough to capture changes in fertility patterns (the distribution of CEB in one age-CEB category compared to another reflects changing fertility patterns) and also to ensure that there are at least 500 women in each region-age-CEB group. It is important to note that the age groupings are not standard 5-y age groups; rather, 2-y age groups are used, with the exception of 15–17 y olds who are grouped into one 3-y age group. Thus, microdata or special tabulations of microdata are required in order to apply these methods. We then apply these distributions to the responses of each mother on the number of children ever born and dead, generating the expected allocation of births and deaths over time (an example is given in the Figure 2 legend). The expected births and expected deaths for each year prior to the survey are summed over all mothers in the survey to generate a period-specific measure of the CD/CEB ratio (as shown in the Lexis diagram, Figure 3). By regressing logit _5_ *q*_0_ from complete birth histories on the logit of the period CD/CEB ratio and a country-level random effect, as shown in the equation below, we obtain the parameters required to map from CD/CEB to _5_ *q*_0_.
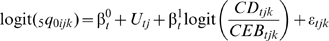
In the period-derived method, the regressions are run for each single year of calendar time (index *t* in the equation) rather than by 5-y age groups, going back 25 y prior to the survey. 25 y was selected as the cut-off point because the relationship between CD/CEB and _5_ *q*_0_ gets weaker (the *R* ^2^ value for the bivariate regression is lower than 90%) for time periods greater than 25 y prior to the survey (see Figure S1).

### Time since First Birth Period-Derived Method (TFBP)

To generate the TFBP estimates, we repeat the steps described for the MAP. The difference occurs in the creation of the frequency distributions of births and deaths across time prior to the survey. Here, mothers are grouped by time since first birth rather than age. The frequency distributions are then applied in the same way as the MAP method to produce period measures of CD/CEB and then included in similar regression models to generate estimates of _5_ *q*_0_.

### Combined Method

In order to create a summary measure, we apply Loess local regression to all the estimates generated from each of the four methods. We exclude the estimate produced by the 15–19-y-old maternal age group since the MAC estimates for this group are still biased upward. Loess enables us to systematically combine all the estimates but does not restrict the predictions to a linear trend over time. We used an alpha value of 0.5 because it allowed for local fluctuations while still applying some smoothing to the data.

### Metrics of Fit and Validation

We use three metrics to assess how well the _5_ *q*_0_ values generated by each method and the standard indirect method compare to the _5_ *q*_0_s in the validation dataset. (1) Average relative error: assigns greater importance to the same absolute error in estimating _5_ *q*_0_ in lower mortality populations. (2) Mean of residuals (bias): captures whether a method is systematically over- or underestimating _5_ *q*_0_. It is a measure of absolute error, in that the same difference in _5_ *q*_0_ counts equally regardless of the level of _5_ *q*_0_. (3) Standard deviation of residuals: captures how much variation there is across country-years in how the method performs compared to the validation measurement.

### In- and Out-of-Sample Analysis

We perform our model development and assess fit and validation two ways. First, we include all data in the sample used to develop the model and compute metrics of fit and validation for this in-sample dataset. Second, we perform an out-of-sample analysis whereby we randomly select 80% of the surveys from which to build our model. We then use the model to predict for the remaining 20%. We repeat this process five times and average the fit and validation results across all out-of-sample groups.

### Uncertainty

We designed our methods and their implementation to estimate uncertainty in the predicted values of _5_ *q*_0_ by sampling from the variance-covariance matrix of the beta coefficients and from the distributions of the residual error terms [35]. These measures of uncertainty capture the parameter uncertainty in mapping between CD/CEB and levels of _5_ *q*_0_. We did not want to propagate fundamental uncertainty, which reflects single-survey errors, only the uncertainty in the underlying true value of under-five mortality. To characterize the uncertainty in the combined method, we draw 1,000 samples from the distributions of each data point of the four methods and generate Loess regressions for each draw. Then we obtain the distribution of the set of predicted estimates from the 1,000 Loess regressions every 0.5 y. In the Loess process, the same information from mothers is reflected four times, which artificially decreases the uncertainty interval by a factor of one-quarter. To obtain the uncertainty interval for the combined method, we first compute the standard error of the distribution of 1,000 Loessed estimates at every 0.5 y, then multiply by 4 and compute the 95% uncertainty interval using the corrected standard error. We have also propagated uncertainty in the underlying validation estimates into our measures comparing the performance of our methods relative to the validation _5_ *q*_0_s.

### Mexico Application

**Figure 4:**
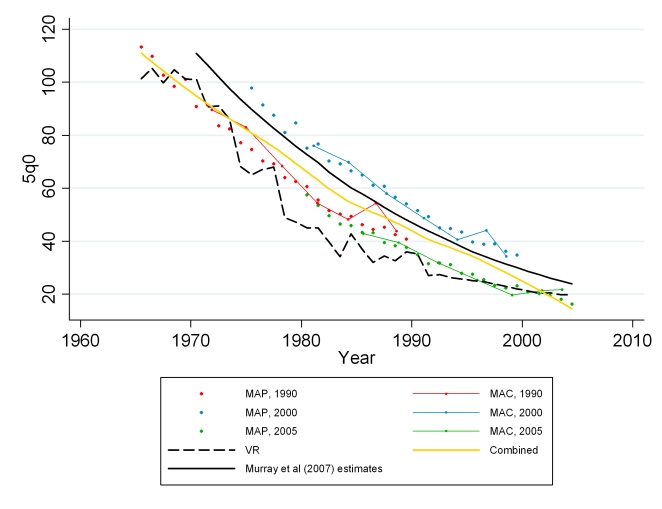
Estimates of under-five mortality generated from summary birth histories from three censuses (1990, 2000, 2005) in Mexico using MAP, MAC, and the combined methods. Summary birth history estimates are compared with national-level estimates from vital registration, the standard indirect method, and Murray et al. [2].

We apply our models to summary birth history data at the Mexican jurisdiction level from the 1990, 2000, and 2005 censuses [39]. The Mexican censuses only include information on maternal age (no question about time since first birth is asked). Therefore, only the MAP and MAC methods were applied. We first applied the models using the Bayesian posterior mean predicted for Mexico from the DHS model. The results showed the 1990 and 2005 estimates to be fairly consistent, whereas the estimates from the 2000 census were generally higher in comparison (see Figure 4). There were some differences in how the summary birth history questions were asked in the 2000 census compared to the other two censuses. The 2000 census asked not only about the number of children who have survived to the time of the census but also asked directly about the number of children who had died. This difference in the instrument may contribute to the systematic differences between the under-five mortality estimates from the 2000 census. To help regulate this inconsistency and to generate a consistent series of subnational estimates, we calibrated the model to the estimates from Murray and colleagues [2] by including the census-specific estimates of CD/CEB and the Murray et al. estimates of 5*q*0 as validation data points in the estimation process for both MAC and MAP. We then added back the census-specific residual so the census data replicated the Murray et al. results at a national level. The combined method using Loess regression was applied to the resulting MAC and MAP predictions, synthesizing estimates across methods and across censuses. We compare estimates from summary birth histories to those generated from vital registration data and to Murray and colleagues' estimates at the national level to confirm the performance of the methods. When generating estimates for each jurisdiction, we average the estimates from the combined method into four 5-y periods to reduce noise owing to small numbers.

## Results

### Performance of New Methods

**Figure 5:**
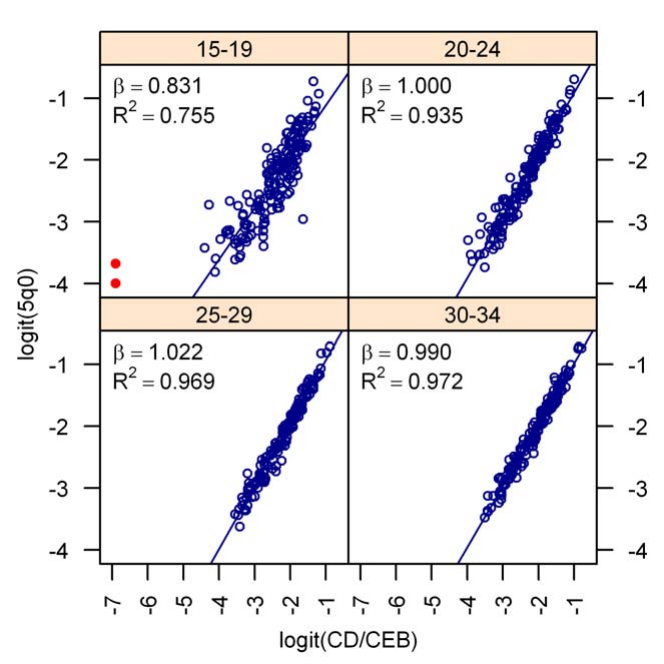
The bivariate relationship between child mortality and the CD/CEB ratio after logit transformation. The relationship between the cohort measure of CD/CEB for four different age groups of mothers is shown. The red points are outliers that were excluded from the model.

**Figure 6:**
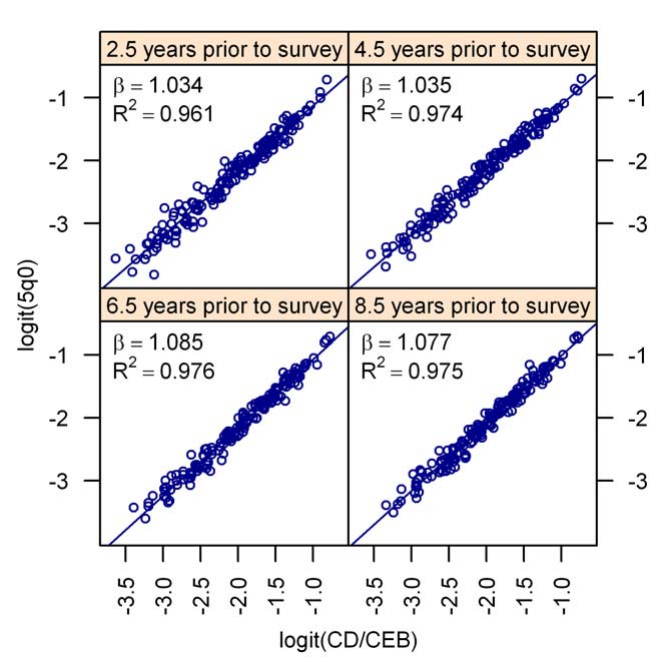
The bivariate relationship between child mortality and the ratio of children dead to children ever born after logit transformation. The relationship for the period measure of CD/CEB for four periods prior to the survey is shown.

Figure 5 shows the bivariate relationship between the logit of CD/CEB and the logit of _5_ *q*_0_ for four age groups of mothers. Figure 6 presents the same relationship for the period measure of CD/CEB and under-five mortality for four different periods of time prior to the survey. The complete set of bivariate relationships for all age groups, time since first birth groups, and years prior to survey for MAP and TFBP are included in Figure S1. The strength of this relationship is the underpinning of all methods that use summary birth histories to derive estimates of child mortality. In our analysis, we have excluded from the regression models a few influential outlier surveys (these are identified in the graphs of Figure S1).

Tables 1–3 show the regression results for each of the models: MAC, MAP, TFBC, and TFBP. Note that the coefficients on the logit of the CD/CEB ratio are very close to 1 and significant (none of the confidence intervals for the coefficients overlap zero), which is reflective of the strength of the bivariate relationship between them, as seen in Figures 5–6. The values of *R* ^2^ in these regressions are all above 90%, which indicates that variation in _5_ *q*_0_ is almost completely explained by the CD/CEB ratio and the country-level random effect in our models.

**Table 1:** Regression results for cohort-derived methods relating under-five mortality to the cohort-computed ratio of CD/CEB.

Method	Age/TSFB Category	*n*	CD/CEB Coefficient	95% CI (Lower)	95% CI (Upper)	Variance of Random Effect	*R* ^2^ ^a^
MAC	15–19	164	0.56	0.47	0.64	0.0804	0.96
MAC	20–24	166	0.89	0.81	0.96	0.0107	0.97
MAC	25–29	166	0.99	0.94	1.03	0.0000	0.97
MAC	30–34	166	0.97	0.93	1.01	0.0006	0.97
MAC	35–39	166	0.97	0.93	1.02	0.0000	0.97
MAC	40–44	166	1.00	0.92	1.07	0.0066	0.97
MAC	45–49	164	1.02	0.96	1.08	0.0000	0.92
TFBC	0–4	165	0.86	0.80	0.92	0.0094	0.97
TFBC	5–9	165	0.97	0.93	1.01	0.0000	0.97
TFBC	10–14	165	0.99	0.95	1.03	0.0001	0.98
TFBC	15–19	165	0.96	0.92	1.00	0.0000	0.97
TFBC	20–24	165	0.98	0.92	1.03	0.0017	0.97
TFBC	25–29	165	1.00	0.93	1.07	0.0000	0.93
TFBC	30–34	161	0.81	0.70	0.91	0.0119	0.93

Note that in all regressions, both the dependent and independent variables are transformed using the logit transformation.

a
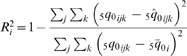
 where *i* = group, *j* = country, *k* = survey, 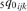 = true _5_ *q*_0_ value for group *ijk*, 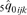 = predicted 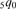 value for group *ijk*, and 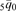 = average _5_ *q*_0_ value for group *i*.

**Table 2 pmed-1000253-t002:** Regression results for MAP-derived method relating under-five mortality to the period-computed ratio of CD/CEB.

Method	Year Prior to Survey	*n*	CD/CEB Coefficient	95% CI (Lower)	95% CI (Upper)	Variance of Random Effect	*R* ^2^ ^a^
MAP	0.5	98	1.14	1.07	1.21	0.0174	0.98
MAP	1.5	166	1.06	1.02	1.11	0.0052	0.97
MAP	2.5	166	1.04	1.00	1.08	0.0054	0.98
MAP	3.5	166	1.03	1.00	1.07	0.0033	0.98
MAP	4.5	166	1.04	1.01	1.07	0.0019	0.98
MAP	5.5	166	0.98	0.95	1.01	0.0023	0.98
MAP	6.5	166	1.08	1.06	1.11	0.0003	0.98
MAP	7.5	166	1.05	1.02	1.07	0.0000	0.98
MAP	8.5	166	1.08	1.05	1.10	0.0000	0.97
MAP	9.5	166	1.01	0.98	1.04	0.0001	0.97
MAP	10.5	166	1.10	1.07	1.14	0.0004	0.97
MAP	11.5	166	0.99	0.95	1.02	0.0003	0.96
MAP	12.5	166	1.06	1.02	1.09	0.0000	0.96
MAP	13.5	166	1.04	1.01	1.08	0.0000	0.95
MAP	14.5	166	1.06	1.02	1.10	0.0000	0.95
MAP	15.5	166	1.05	1.01	1.09	0.0000	0.94
MAP	16.5	166	1.04	1.00	1.08	0.0000	0.94
MAP	17.5	166	1.04	0.99	1.08	0.0000	0.94
MAP	18.5	166	1.06	1.02	1.11	0.0006	0.94
MAP	19.5	166	0.96	0.92	1.01	0.0043	0.94
MAP	20.5	166	1.08	1.03	1.13	0.0033	0.95
MAP	21.5	163	1.00	0.95	1.04	0.0031	0.95
MAP	22.5	162	1.05	1.00	1.09	0.0020	0.95
MAP	23.5	153	0.99	0.94	1.04	0.0072	0.96
MAP	24.5	147	1.01	0.96	1.07	0.0093	0.96

Note that in all regressions, both the dependent and independent variables are transformed using the logit transformation.

a
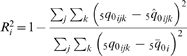
 where *i* = group, *j* = country, *k* = survey, 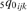 = true 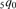 value for group *ijk*, 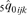 = predicted _5_ *q*_0_ value for group *ijk*, and 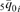 = average 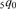 value for group *i*.

**Table 3:** Regression results for TFBP-derived method relating under-five mortality to the period-computed ratio of CD/CEB.

Method	Year Prior to Survey	*n*	CD/CEB Coefficient	95% CI (Lower)	95% CI (Upper)	Variance of Random Effect	*R* ^2^ ^a^
TFBP	0.5	96	1.12	1.03	1.22	0.0262	0.97
TFBP	1.5	164	1.11	1.06	1.16	0.0089	0.97
TFBP	2.5	164	1.07	1.02	1.11	0.0064	0.98
TFBP	3.5	164	1.05	1.01	1.10	0.0092	0.98
TFBP	4.5	164	1.04	1.01	1.08	0.0035	0.98
TFBP	5.5	164	0.99	0.96	1.03	0.0055	0.98
TFBP	6.5	164	1.07	1.04	1.10	0.0011	0.98
TFBP	7.5	164	1.04	1.01	1.07	0.0021	0.98
TFBP	8.5	164	1.05	1.03	1.08	0.0000	0.98
TFBP	9.5	164	1.00	0.98	1.03	0.0005	0.98
TFBP	10.5	164	1.07	1.04	1.11	0.0012	0.98
TFBP	11.5	164	0.98	0.95	1.01	0.0005	0.97
TFBP	12.5	164	1.02	0.99	1.06	0.0000	0.96
TFBP	13.5	164	1.03	0.99	1.06	0.0000	0.96
TFBP	14.5	164	1.04	1.00	1.07	0.0000	0.95
TFBP	15.5	164	1.04	1.01	1.08	0.0008	0.95
TFBP	16.5	164	1.02	0.99	1.06	0.0000	0.95
TFBP	17.5	164	1.03	0.99	1.06	0.0000	0.95
TFBP	18.5	164	1.03	0.99	1.08	0.0021	0.95
TFBP	19.5	164	0.95	0.91	1.00	0.0043	0.95
TFBP	20.5	164	1.05	1.00	1.11	0.0063	0.96
TFBP	21.5	161	0.99	0.95	1.04	0.0045	0.96
TFBP	22.5	160	1.02	0.97	1.07	0.0065	0.96
TFBP	23.5	151	0.98	0.92	1.03	0.0123	0.97
TFBP	24.5	145	0.99	0.93	1.06	0.0191	0.97

Note that in all regressions, both the dependent and independent variables are transformed using the logit transformation.

a
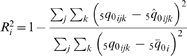
 where *i* = group, *j* = country, *k* = survey, 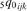 = true 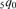 value for group *ijk*, 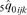 = predicted 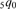 value for group *ijk*, and 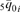 = average 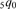 value for group *i*.

**Figure 7:**
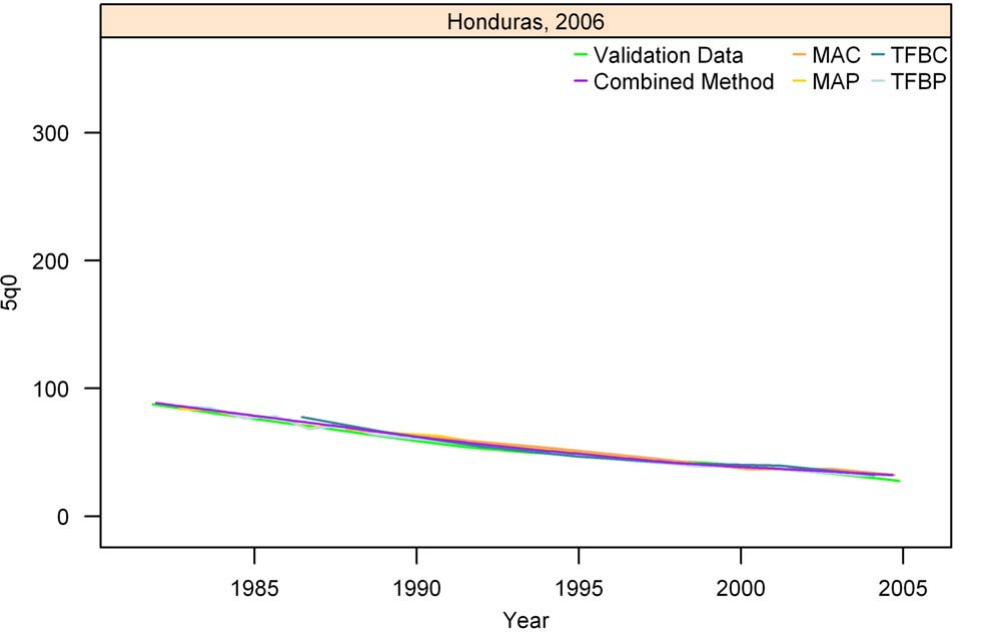
Estimates of under-five mortality generated from summary birth histories using MAP, MAC, TFBP, TFBC, and Combined method. Honduras, 2006. The gold standard derived from complete birth histories is also shown. The six examples in Figures 7-12 represent different levels of performance of the Combined method, in order from best to worst.

Figures 7–12 show the estimates for each of the MAC, MAP, TFBC, TFBP, and Loess methods for six selected surveys (the full set of graphs for every survey in our analysis can be found in Figure S2). The surveys shown in Figures 7–12 were selected to represent a range of levels of performance, with Honduras 2006 (Figure 7) reflecting very good performance and Nigeria 2003 (Figure 12) reflecting poor performance. The four methods are generally very consistent. The period-derived methods tend to be slightly noisier over time than the cohort-derived methods—overall, the standard deviations of the residuals are 14.55 and 13.66 for MAP and TFBP, respectively, and 12.04 and 13.17 for MAC and TFBC (performance results are further discussed below). However, while the period-derived methods yield slightly greater error and noise, they do generate estimates closer to the time of survey as well as farther back in time than the cohort-derived methods. Uncertainty is not shown in these graphs for simplicity, but has been determined for each method.

**Figure 8 pmed-1000253-g008:**
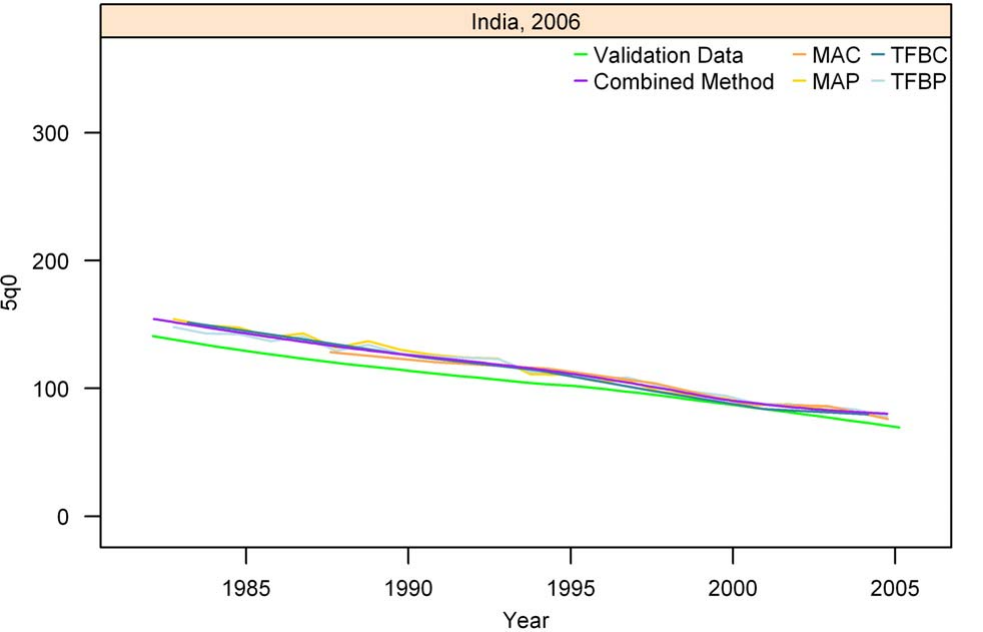
Estimates of under-five mortality generated from summary birth histories using MAP, MAC, TFBP, TFBC, and Combined method. India, 2006. The gold standard derived from complete birth histories is also shown. The six examples in Figures 7-12 represent different levels of performance of the Combined method, in order from best to worst.

**Figure 9 pmed-1000253-g009:**
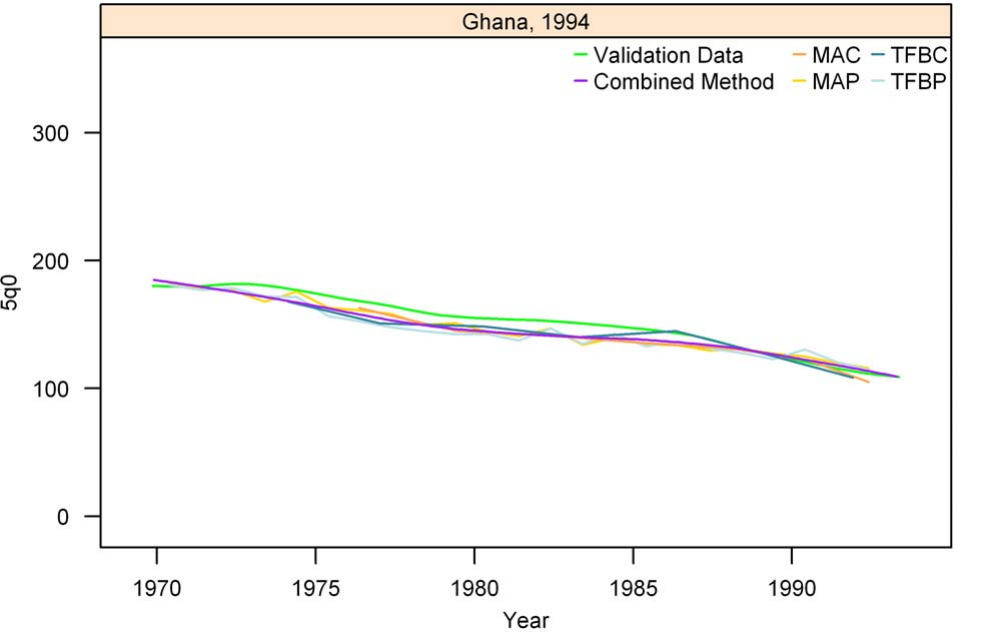
Estimates of under-five mortality generated from summary birth histories using MAP, MAC, TFBP, TFBC, and Combined method. Ghana, 1994. The gold standard derived from complete birth histories is also shown. The six examples in Figures 7-12 represent different levels of performance of the Combined method, in order from best to worst.

**Figure 10 pmed-1000253-g010:**
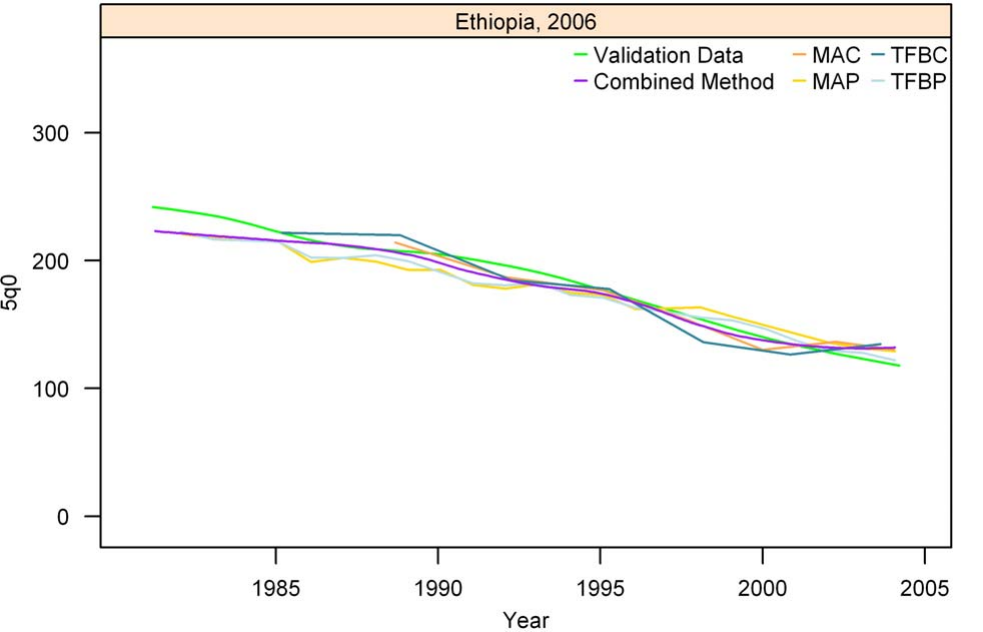
Estimates of under-five mortality generated from summary birth histories using MAP, MAC, TFBP, TFBC, and Combined method. Ethiopia, 2006. The gold standard derived from complete birth histories is also shown. The six examples in Figures 7-12 represent different levels of performance of the Combined method, in order from best to worst.

**Figure 11 pmed-1000253-g011:**
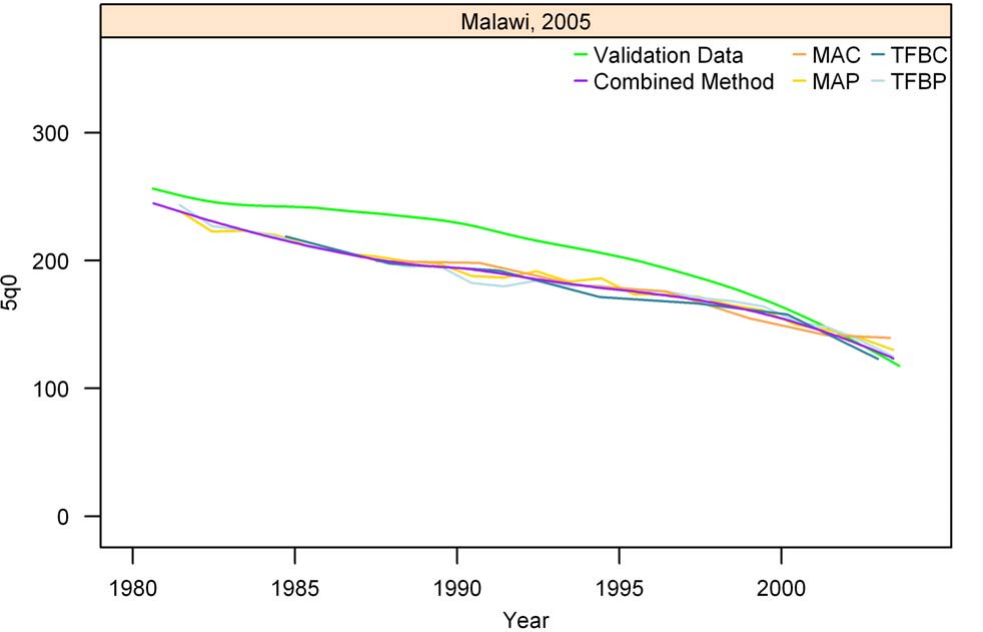
Estimates of under-five mortality generated from summary birth histories using MAP, MAC, TFBP, TFBC, and Combined method. Malawi, 2005. The gold standard derived from complete birth histories is also shown. The six examples in Figures 7-12 represent different levels of performance of the Combined method, in order from best to worst.

**Figure 12:**
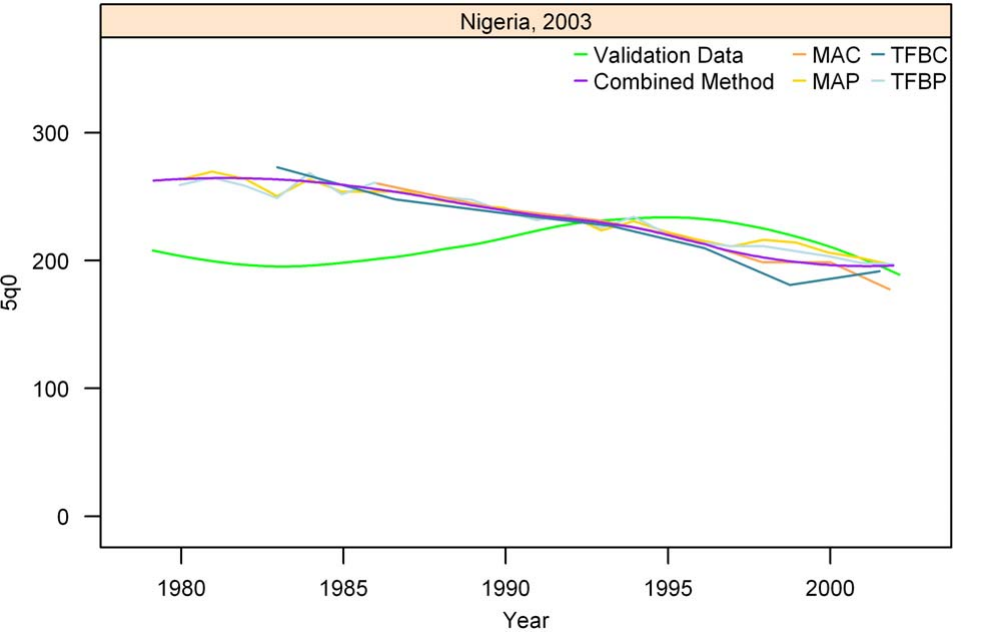
Estimates of under-five mortality generated from summary birth histories using MAP, MAC, TFBP, TFBC, and Combined method. Nigeria, 2003. The gold standard derived from complete birth histories is also shown. The six examples in Figures 7-12 represent different levels of performance of the Combined method, in order from best to worst.

**Figure 13:**
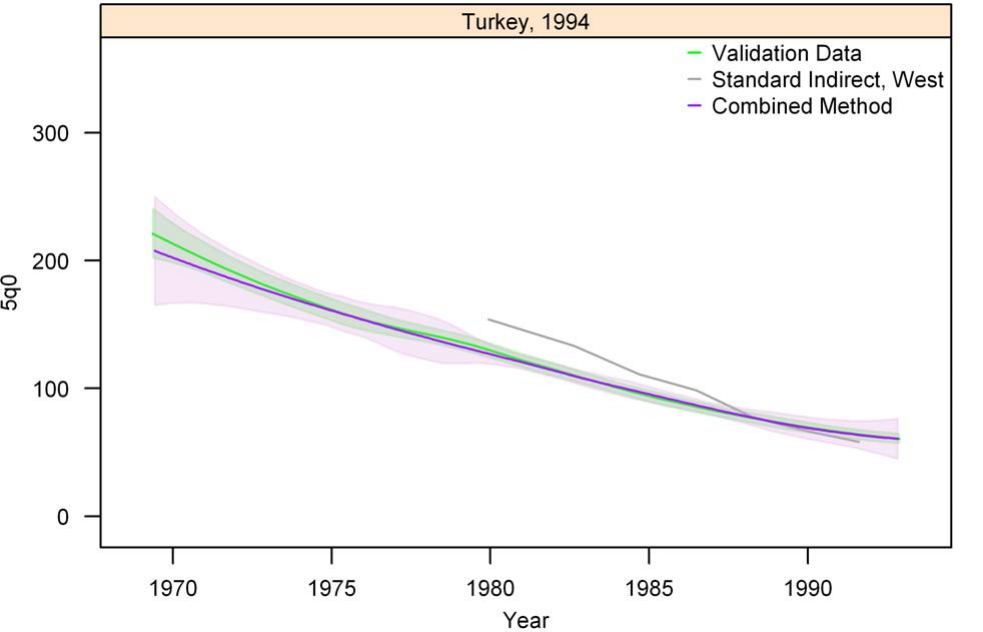
Graphs of estimates from summary birth histories using the best-performing combined method and the standard indirect (West) method. Section I, Turkey, 1994. The gold standard generated from complete birth histories is also shown. Section I (Figures 13–16) shows four surveys that best illustrate the improvement in measurement of under-five mortality when the combined method is used as compared to the standard method. Section II (Figures 17–20) shows four surveys that provide a more balanced representation of the performance of the combined method, including the surveys that perform at the best, 33rd, 66th, and worst percentiles, in that order, on the basis of the standard deviation of residuals metric.

Figures 13–20 show the combined method with uncertainty, compared to the validation data and standard method, for several countries. The four surveys shown in section I (Figures 13–16) reflect the four surveys that best illustrate the improvement in performance of the combined method as compared to the standard indirect method. Note that the characteristic overestimation seen in the most recent periods prior to the survey with the standard method (Bolivia 1998 provides a classic example, Figure 15) is no longer present with the combined method. The four surveys in section II (Figures 17–20) reflect the best, 33rd, 66th, and worst percentiles of performance of the combined method (on the basis of the standard deviation of residuals metric). One major weakness of the new method is highlighted in the graph for Rwanda 2001 (Figure 20)—dramatic shifts in child mortality are not well-reflected by these methods. The uncertainty interval generated from summary birth history data is wider at the ends—most immediately prior to the survey and further back in time. The interval is also wider where the four methods are less concordant with each other. On average, the uncertainty intervals from summary birth histories are a little less than twice as wide as those generated from complete birth histories.

**Figure 14 pmed-1000253-g014:**
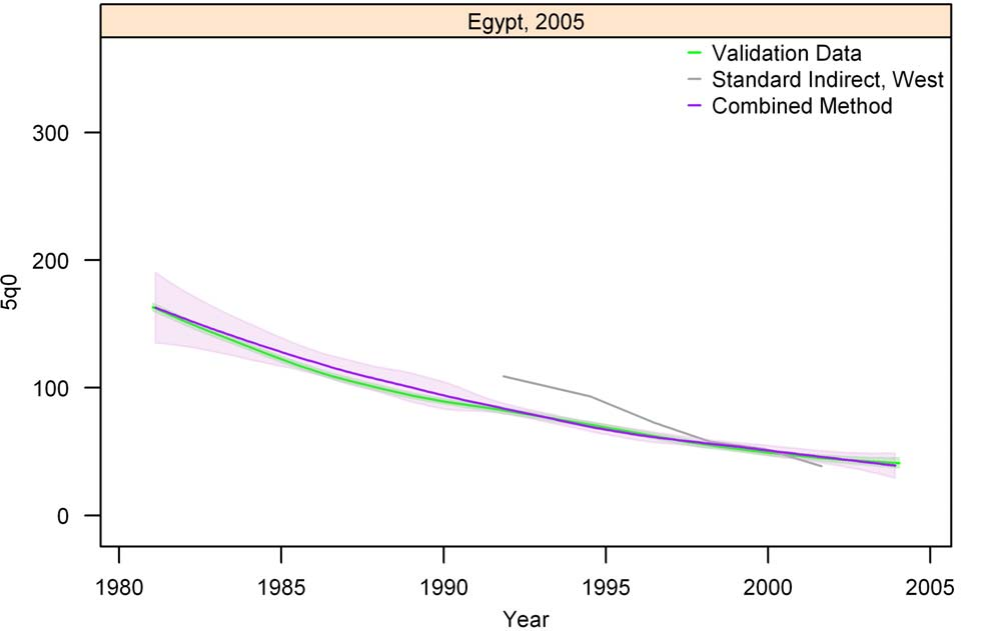
Graphs of estimates from summary birth histories using the best-performing combined method and the standard indirect (West) method. Section I, Egypt, 2005. The gold standard generated from complete birth histories is also shown. Section I (Figures 13–16) shows four surveys that best illustrate the improvement in measurement of under-five mortality when the combined method is used as compared to the standard method. Section II (Figures 17–20) shows four surveys that provide a more balanced representation of the performance of the combined method, including the surveys that perform at the best, 33rd, 66th, and worst percentiles, in that order, on the basis of the standard deviation of residuals metric.

**Figure 15 pmed-1000253-g015:**
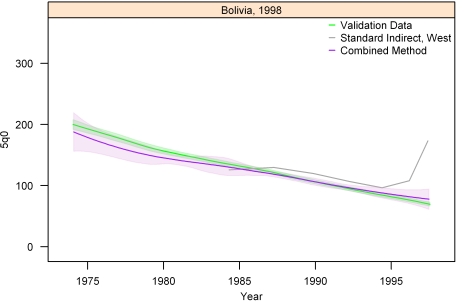
Graphs of estimates from summary birth histories using the best-performing combined method and the standard indirect (West) method. Section I, Bolivia, 1998. The gold standard generated from complete birth histories is also shown. Section I (Figures 13–16) shows four surveys that best illustrate the improvement in measurement of under-five mortality when the combined method is used as compared to the standard method. Section II (Figures 17–20) shows four surveys that provide a more balanced representation of the performance of the combined method, including the surveys that perform at the best, 33rd, 66th, and worst percentiles, in that order, on the basis of the standard deviation of residuals metric.

**Figure 16 pmed-1000253-g016:**
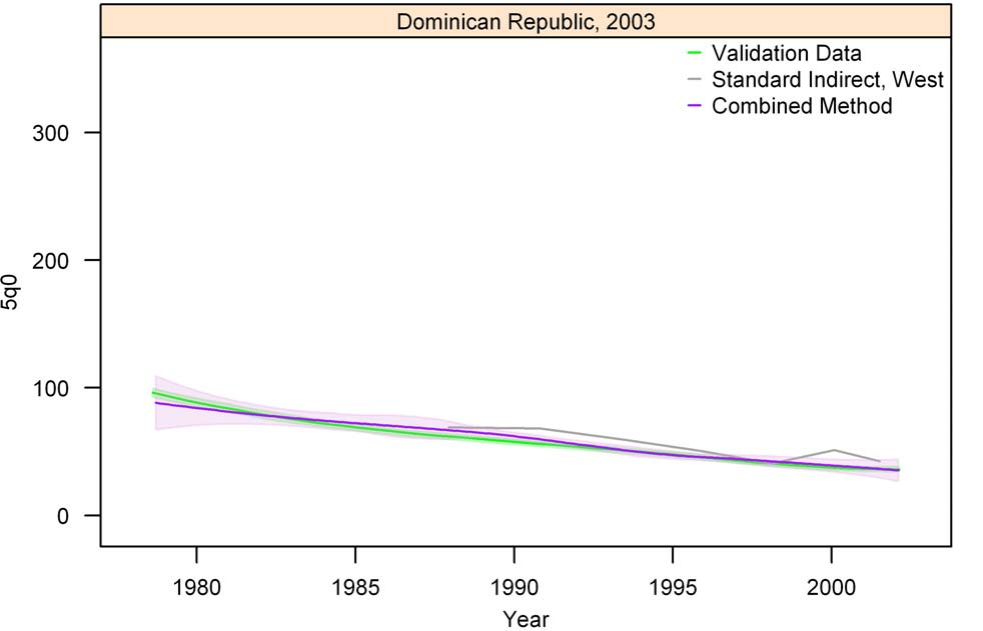
Graphs of estimates from summary birth histories using the best-performing combined method and the standard indirect (West) method. Section I, Dominican Republic, 2003. The gold standard generated from complete birth histories is also shown. Section I (Figures 13–16) shows four surveys that best illustrate the improvement in measurement of under-five mortality when the combined method is used as compared to the standard method. Section II (Figures 17–20) shows four surveys that provide a more balanced representation of the performance of the combined method, including the surveys that perform at the best, 33rd, 66th, and worst percentiles, in that order, on the basis of the standard deviation of residuals metric.

**Figure 17 pmed-1000253-g017:**
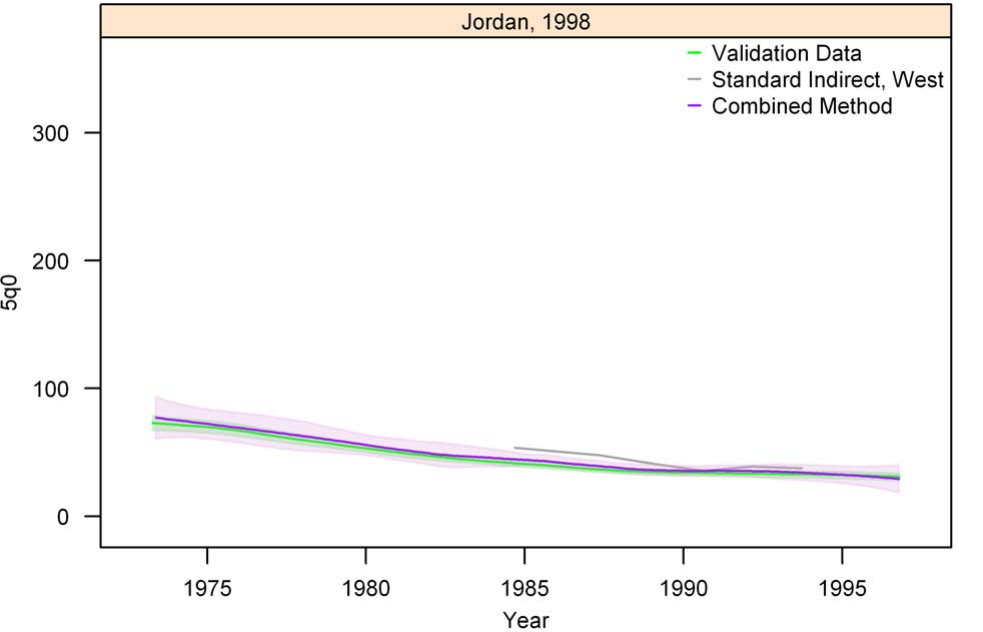
Graphs of estimates from summary birth histories using the best-performing combined method and the standard indirect (West) method. Section II, Jordan, 1998. The gold standard generated from complete birth histories is also shown. Section I (Figures 13–16) shows four surveys that best illustrate the improvement in measurement of under-five mortality when the combined method is used as compared to the standard method. Section II (Figures 17–20) shows four surveys that provide a more balanced representation of the performance of the combined method, including the surveys that perform at the best, 33rd, 66th, and worst percentiles, in that order, on the basis of the standard deviation of residuals metric.

**Figure 18 pmed-1000253-g018:**
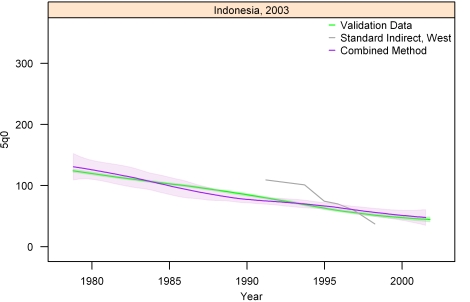
Graphs of estimates from summary birth histories using the best-performing combined method and the standard indirect (West) method. Section II, Indonesia, 2003. The gold standard generated from complete birth histories is also shown. Section I (Figures 13–16) shows four surveys that best illustrate the improvement in measurement of under-five mortality when the combined method is used as compared to the standard method. Section II (Figures 17–20) shows four surveys that provide a more balanced representation of the performance of the combined method, including the surveys that perform at the best, 33rd, 66th, and worst percentiles, in that order, on the basis of the standard deviation of residuals metric.

**Figure 19 pmed-1000253-g019:**
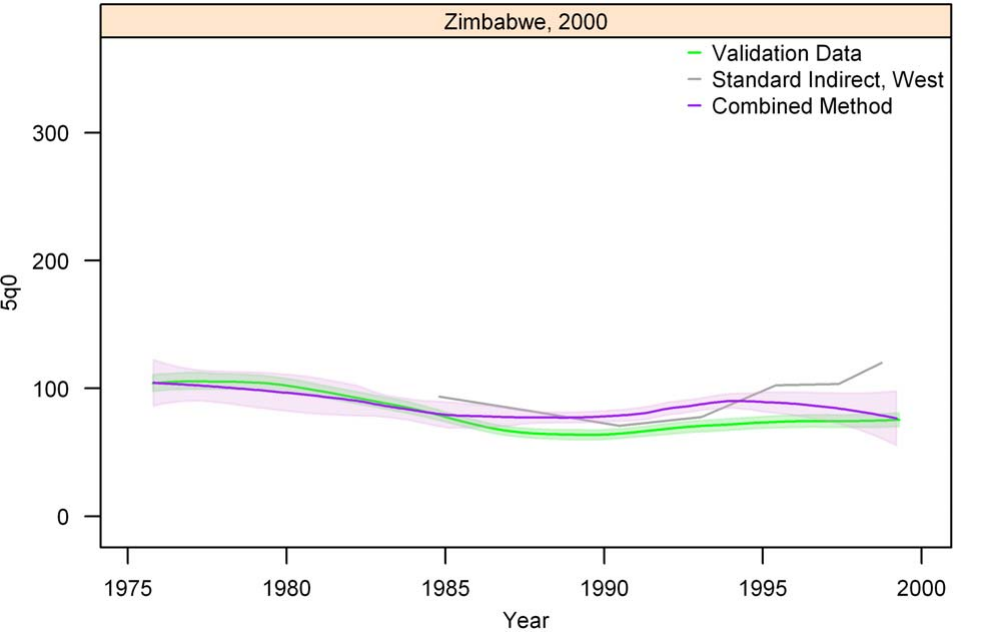
Graphs of estimates from summary birth histories using the best-performing combined method and the standard indirect (West) method. Section II, Zimbabwe, 2000. The gold standard generated from complete birth histories is also shown. Section I (Figures 13–16) shows four surveys that best illustrate the improvement in measurement of under-five mortality when the combined method is used as compared to the standard method. Section II (Figures 17–20) shows four surveys that provide a more balanced representation of the performance of the combined method, including the surveys that perform at the best, 33rd, 66th, and worst percentiles, in that order, on the basis of the standard deviation of residuals metric.

**Figure 20:**
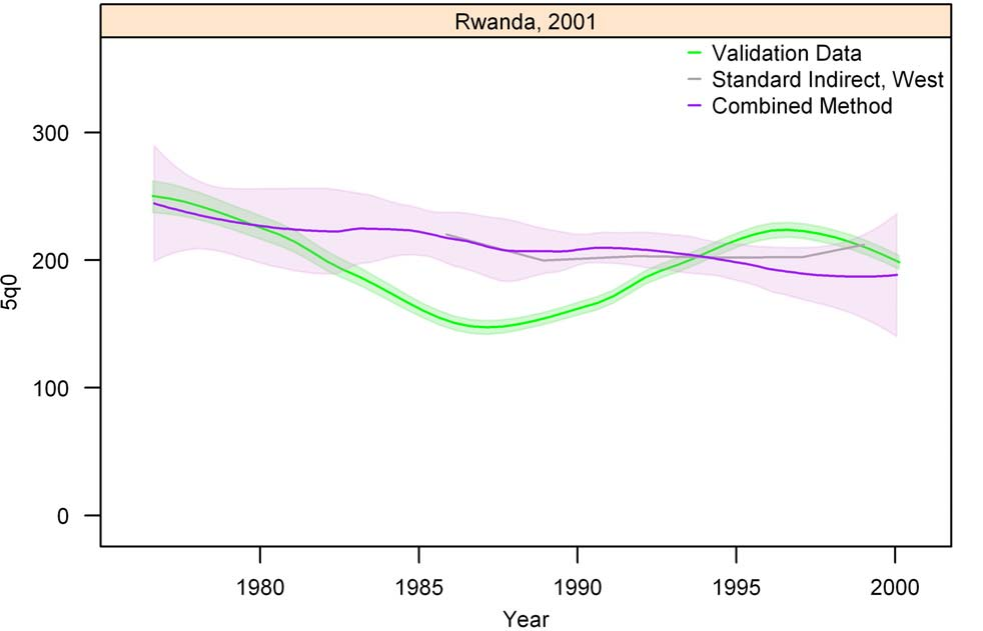
Graphs of estimates from summary birth histories using the best-performing combined method and the standard indirect (West) method. Section II, Rwanda, 2001. The gold standard generated from complete birth histories is also shown. Section I (Figures 13–16) shows four surveys that best illustrate the improvement in measurement of under-five mortality when the combined method is used as compared to the standard method. Section II (Figures 17–20) shows four surveys that provide a more balanced representation of the performance of the combined method, including the surveys that perform at the best, 33rd, 66th, and worst percentiles, in that order, on the basis of the standard deviation of residuals metric.

**Figure 21:**
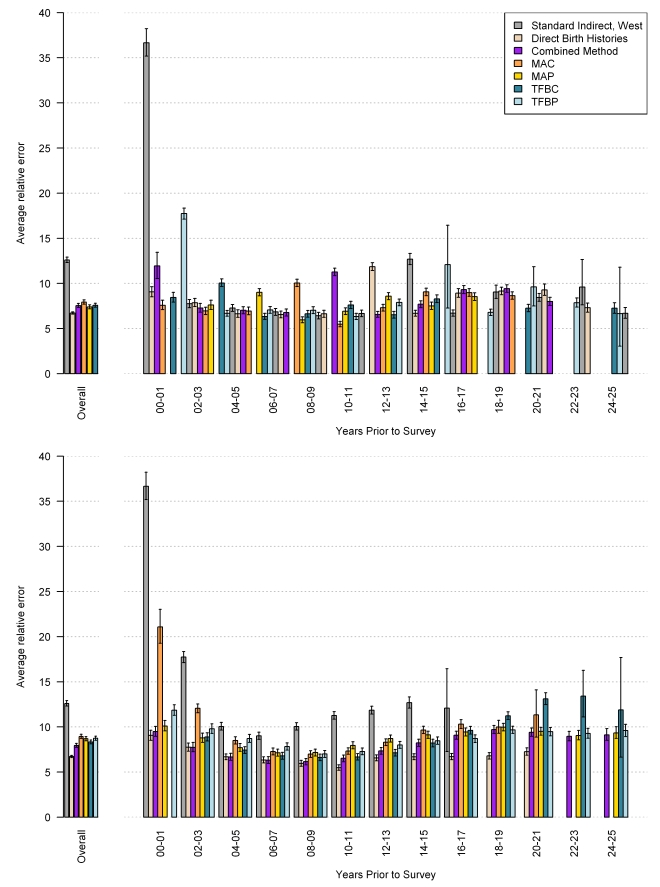
Performance of MAC, TFBC, MAP, TFBP, and combined methods as measured by average relative error. Estimates generated by complete birth histories from a single survey are also compared to the validation dataset. The top section of each panel represents the in-sample performance, and the bottom section is the average out-of-sample performance across all five hold-out groups.

**Figure 22 pmed-1000253-g022:**
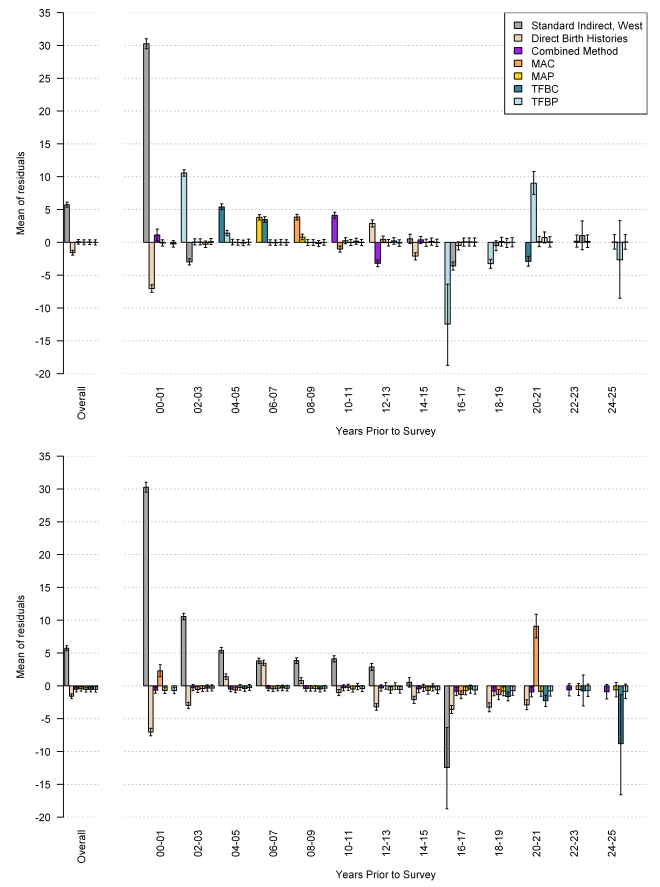
Performance of MAC, TFBC, MAP, TFBP, and combined methods as measured by mean of residuals. Estimates generated by complete birth histories from a single survey are also compared to the validation dataset. The top section of each panel represents the in-sample performance, and the bottom section is the average out-of-sample performance across all five hold-out groups.

**Figure 23:**
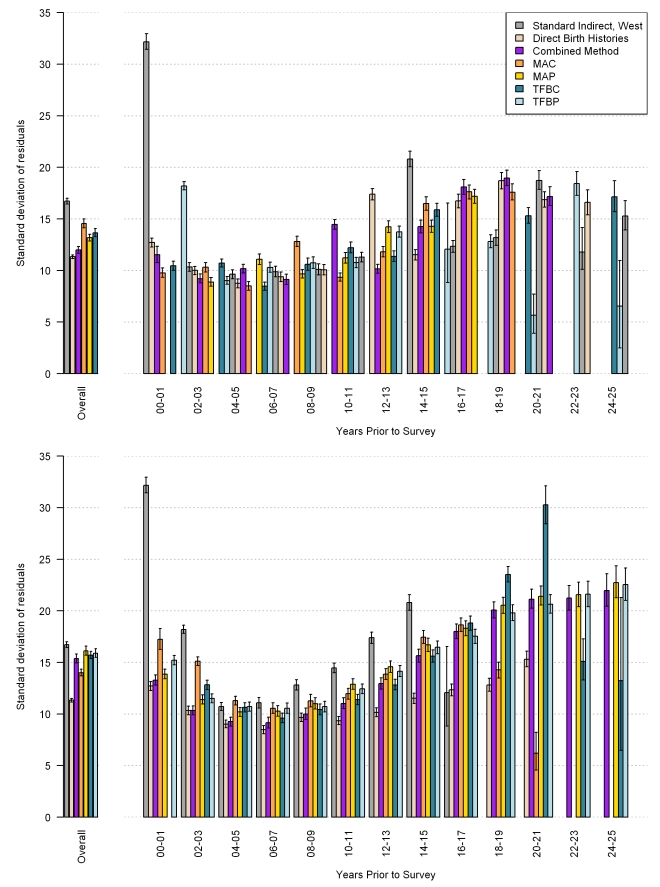
Performance of MAC, TFBC, MAP, TFBP, and combined methods as measured by standard deviation of residuals. Estimates generated by complete birth histories from a single survey are also compared to the validation dataset. The top section of each panel represents the in-sample performance, and the bottom section is the average out-of-sample performance across all five hold-out groups.

The performance of each method, overall and by time prior to the survey, as measured by average relative error, is shown in Figure 21, with mean of residuals in Figure 22, and the standard deviation of residuals in Figure 23. The upper graph shows the performance of the in-sample analysis, whereas the lower graph shows the average performance across all out-of-sample subgroups. We also compare estimates of _5_ *q*_0_ using complete birth histories from a single survey to the estimates of _5_ *q*_0_, which rely on pooled data from multiple surveys using these same metrics of fit (shown in the graph legend as direct birth histories). As expected, the average relative error in each of the MAC, MAP, TFBP, TFBC, and combined methods increases when predicting out of sample. However, it remains lower than the error associated with the standard method across all time periods. The cohort and period-derived methods perform similarly with respect to average relative error; however, the period-derived methods have the additional benefit of allowing for estimation closer to the time of the survey and further back in time as well. The combined method performs best and is always lower than the standard method, both in and out of sample. In fact, the out-of-sample analysis indicates that the combined method results in a 43.7% (95% confidence interval [CI] 42.2–45.2) reduction in error over all time periods and a 53.3% (95% CI 51.3–55.2) reduction in error averaged over the 5 y prior to the survey.

Figure 21 also suggests that, in the 5 y prior to the survey, the combined method using summary birth history data performs about as well as estimates generated from complete birth histories from a single survey (the rose-colored bar). Recall that the validation dataset relies on pooled data from complete birth histories from all DHS within a country. We have compared estimates generated from complete birth history data for a single DHS survey to the validation dataset; these are the rose-colored bars in Figures 21–23. Figure 22 suggests, however, that the estimates from single-survey complete birth histories are biased downward in the 5 y prior to the survey, whereas the combined method yields nearly unbiased estimates in the same time period. Figure 23 reflects the variation in performance across surveys. The standard deviation of residuals is less for all five new methods compared to the standard indirect method, especially in the recent time periods.

### Application of Methods for Mexico

**Figure 24:**
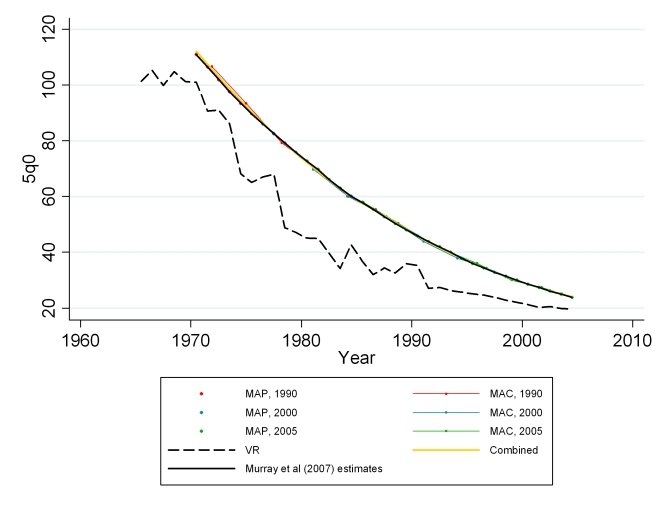
Estimates of under-five mortality generated from summary birth histories from three censuses (1990, 2000, 2005) in Mexico using MAP, MAC, and the combined methods. Summary birth history estimates are calibrated to national-level estimates from Murray et al. [2].

**Figure 25:**
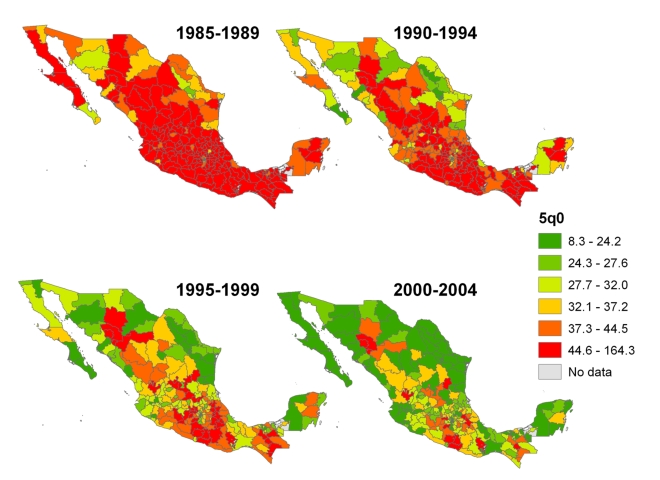
Estimates of under-five mortality by jurisdiction in Mexico, using the combined method approach of applying Loess regression to MAP and MAC estimates from the 1990, 2000, and 2005 censuses.

Figure 24 shows national-level estimates of under-five mortality by applying the MAC and MAP methods to the 1990, 2000, and 2005 censuses, calibrated to the estimates from Murray et al. [2]. Estimates from vital registration are also shown. The summary birth history estimates are necessarily consistent with the estimates from Murray et al. and also mirror the overall trend of the estimates from vital registration, with some convergence of the series in the most recent years. This convergence is due largely to the improvement of the vital registration system in Mexico [14],[40]. Figure 25 shows estimates of under-five mortality generated for each of 233 jurisdictions in Mexico for four time periods, 1985–1989, 1990–1994, 1995–1999, and 2000–2004. The four maps show an overall dramatic decline in mortality between 1985 and 2004. However, the decline was not homogenous across all parts of Mexico. Under-five mortality remains high in certain regions of Mexico, including jurisdictions in the states of Chihuahua, Jalisco, Guerrero, and Puebla. The application of these methods to generate subnational estimates is particularly impressive considering that the total population size in some of these jurisdictions is less than 30,000. These methods will provide a powerful tool for policy makers seeking to reduce the overall level of child mortality as well as disparities within the country.

## Discussion

Using available empirical datasets, we developed new methods for analyzing survey information on children ever born and children who have died to estimate child mortality for periods immediately before the survey or census. We validated our methods both internally using direct estimates from complete birth histories, and externally with an out-of-sample prediction analysis. We also incorporated uncertainty estimation to improve the overall performance of these low-cost methods to reliably measure changes in child mortality levels.

In an era of increasing global concern with improving child survival, the need to assess the impact of global disease control strategies and investments is becoming increasingly urgent. More than 8 million children continue to die each year as a result of largely preventable conditions. While there has been impressive progress in reducing child mortality over recent decades, much more remains to be done. Timely information about the success of intervention programs is critical for policy debates, yet current methods to measure child mortality cannot meet this need. Existing methods require an extensive array of questions to assess complete birth histories of women, and hence substantial resources, thus limiting their applicability for assessing inequalities in child mortality among local populations. In this paper, we have proposed new methods for measuring child mortality that overcome these important limitations and, in addition, provide uncertainty estimates for the formal statistical assessment of trends in child survival.

The results represent a dramatic improvement in our ability to estimate under-five mortality using summary birth history data. Not only are the new methods more accurate than standard indirect methods, they also incorporate measures of uncertainty and, in the case of the period-derived methods, cover a broader time range than the standard method. We can now better characterize what we really know about child mortality.

Much can be learned immediately about both national levels and inequalities in child mortality from the application of these methods to the 2000 round of census data where questions on children ever born and children surviving were routinely asked of respondents in more than 95 countries. We illustrated an example with the application of these methods to Mexican censuses.

Additionally, the estimation of under-five mortality in populations heavily affected by HIV can be improved because of these new methods. Because the complete birth history data from the DHS reflect the increased variation across different maternal age groups (and categories of time since first birth) in mortality of children, the relationship between the CD/CEB ratio and under-five mortality is better captured in these contexts than in the standard indirect model. It is important to acknowledge, however, that these patterns are still generalized over regions (period-derived methods) and over time (all methods), so the more extreme effects of HIV, which are observed in the complete birth histories, are tempered by these methods. Further, though improved, the method still makes the assumption that there is no correlation between survival of the potential respondents to the survey and the survival of their children, which is not the case in populations affected by HIV. In these populations, estimates will still be biased downward during periods of significant HIV mortality.

These few questions will be asked of most respondents in developing countries in the coming 2010 census round. We intend to provide a user-friendly application as well as the open source code for applying these methods in R. With this tool, analysts in countries will be able to easily apply the methods to assess levels and recent trends in child mortality and its distribution within countries. The application will allow users to apply the models presented in this paper to summary birth history data. Microdata or specially tabulated data (e.g., by 2-y maternal age groups) is required for application of the period-derived methods. Standard tabulated data (e.g., by 5-y maternal age groups) can be used with the cohort-derived methods. R is a free software package for statistical analysis and can be downloaded at http://www.r-project.org/. Until the application is completed and released, the full results from all models and a tutorial on how to apply them can be downloaded from the *PLoS Medicine* (Text S1) or the Institute for Health Metrics and Evaluation (www.healthmetricsandevaluation.org) Web sites. This analysis, in turn, will provide a critical evidence base to guide intervention strategies for countries and the donor community.

Given the simplicity of summary birth histories, we would argue that the two basic questions (or a set of questions from which these two basic quantities can be calculated) should be routinely included in all household surveys. This design, together with a commitment to facilitating public access to the survey results, would allow further analyses and a stronger evidence base across populations to monitor progress in global child-survival strategies. The reduction of inequalities in child mortality is a key component of this strategy, the evidence base for which can be assembled from routine application of our methods.

There remain some important limitations with our methods. Summary birth histories can only approximately locate reported births and deaths in time. Our cohort-derived methods use maternal age or time since first birth to estimate the time period during which a woman's children were exposed to the risk of death between ages 0 and 5 y. Error remains in the estimation of time prior to the survey to which the cohort estimates apply. Similarly, when we create and apply regional distributions of births and deaths across time prior to the survey in the period-derived methods, we are generalizing the time localization of births and deaths across countries and across time. As a result, unique mortality experiences within a particular country and/or at a particular time will not be reflected as well. Although we selected an alpha value to minimize this error, the application of local regression in the combined method smoothes the data and introduces the risk that short-term fluctuations in mortality will not be well-reflected. We do not believe our methods work well in cases such as the Rwandan or Cambodian genocides, for example, when there are extreme changes in mortality rates over a short period of time.

Finally, some limitations may exist in the application of these methods to other data sources where summary birth histories are captured differently than in the DHS. For example, the Mexican censuses ask about children ever born and children surviving (or children who have died) for every female of reproductive age. The female herself is not necessarily the respondent, and many fewer questions are asked than are found on the DHS instrument from which these models were developed. In the application to Mexican censuses, we observe some differences between the results of the methods as applied to each census (the 2000 census yields systematically higher estimates than the 1990 or 2005 censuses). It is possible that differences in the survey instruments contributed to the inconsistency. Nevertheless, all estimates were generally consistent in trend with vital registration data and with estimates of under-five mortality in Mexico from other sources [2]. One solution to address the problem of discrepant data sources in Mexico (assuming no knowledge as to which data source is best) is to calibrate the data sources at a national level before generating subnational estimates. Other techniques also exist for addressing discrepancies in estimates from multiple sources (the Loess method used by Murray et al. [2] being one example).

Despite these limitations, the methods proposed here represent a major advance on current practice and offer the prospect of vastly increasing our knowledge about levels, recent trends, and inequalities in child mortality. If we are to make rapid progress with the unfinished agenda of reducing child deaths, policy and practice must be better informed by more comprehensive, relevant, and timely information. Systematic application of the methods proposed here will establish that evidence base, and thereby increase accountability among countries and the global health community to accelerate efforts to reduce the global toll of child deaths.

## Supporting Information

Figure S1 Bivariate relationships between CD/CEB and 5*q*0 for each age or time-since-first-birth group (for cohort-derived methods) and each year prior to the survey (for period-derived methods).
(1.51 MB PDF)

Figure S2 Results from MAC, MAP, TFBC, TFBP, and combined methods applied to each of 166 DHS.
(5.93 MB PDF)

Table S1 Characteristics of the DHS used to build models relating under-five mortality to the CD/CEB ratio and other covariates.
(0.18 MB PDF)

Text S1 Application of summary birth history methods for estimating under-five mortality.
(7.27 MB ZIP)

## Abbreviations

CD/CEB: children dead to children ever born
CI: confidence interval
DHS: Demographic and Health Survey
MAC: maternal age cohort-derived method
MAP: maternal age period-derived method
TFBC: time since first birth cohort-derived method
TFBP: time since first birth period-derived method
