# Supplementary figures and images for: Measuring Under-Five Mortality: Validation of New Low-Cost Methods
Source: PLoS Med. 2010 Apr 13;7(4):e1000253. doi: 10.1371/journal.pmed.1000253 (PMC2854123; doi:10.1371/journal.pmed.1000253)

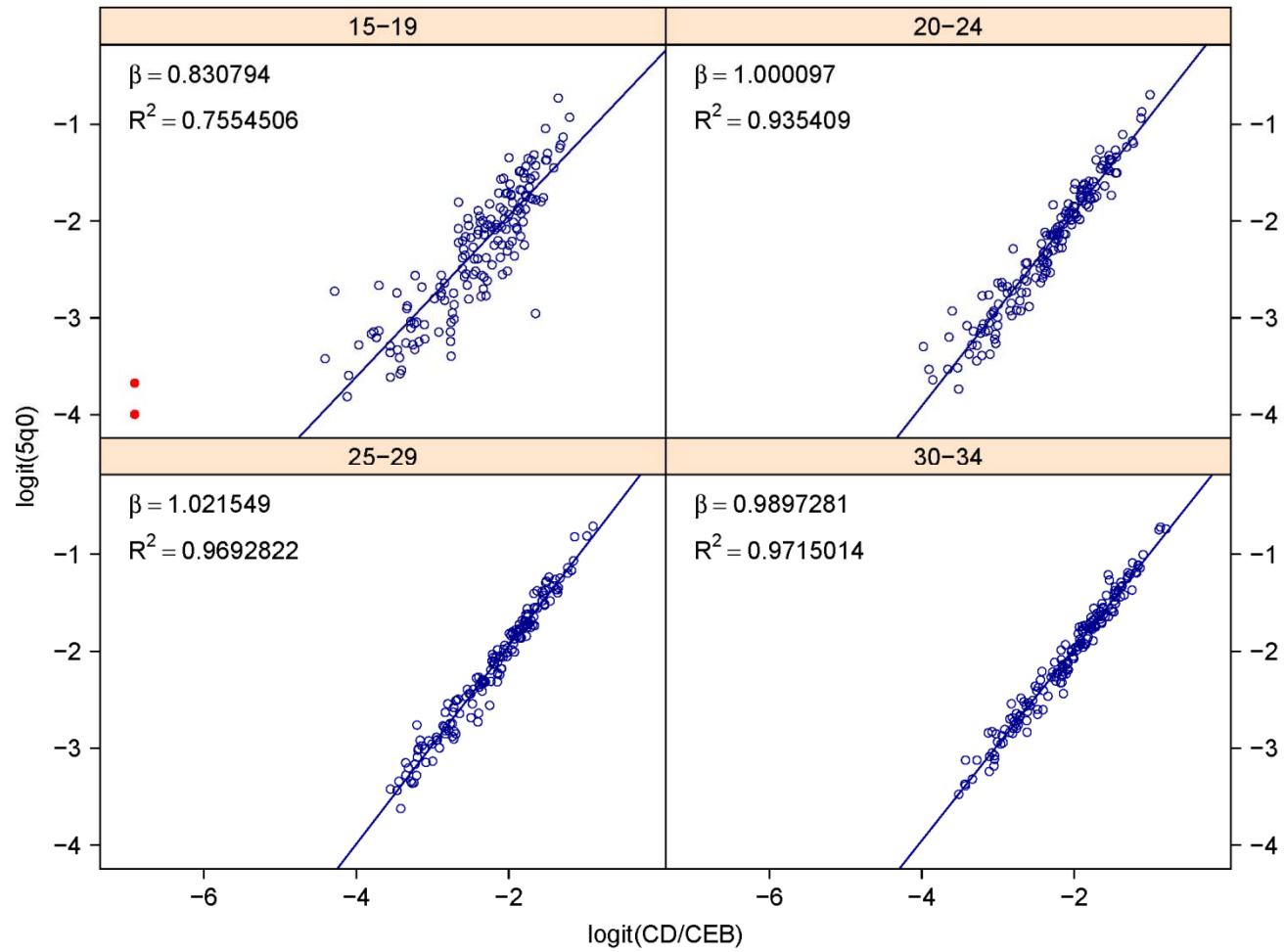

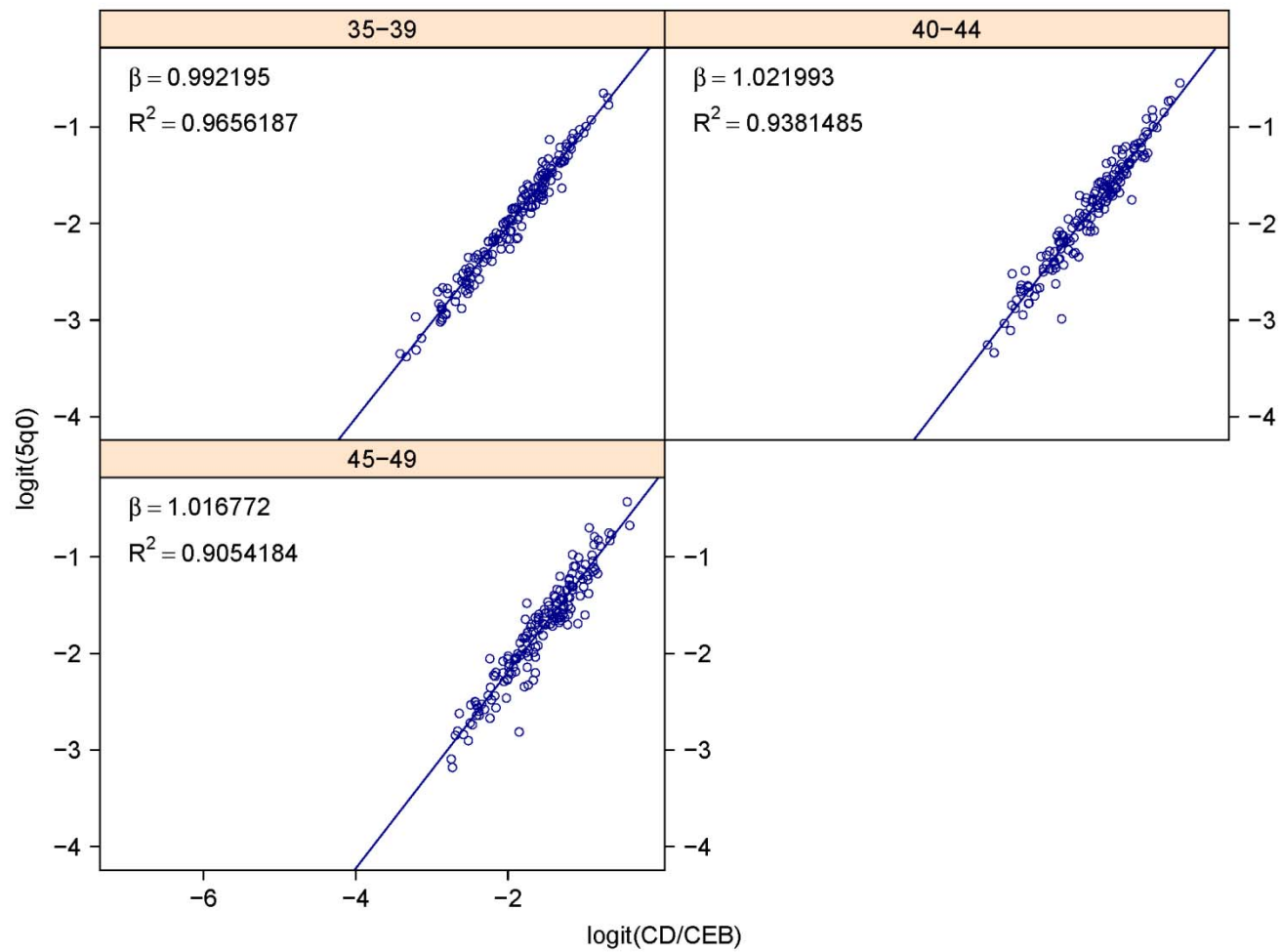

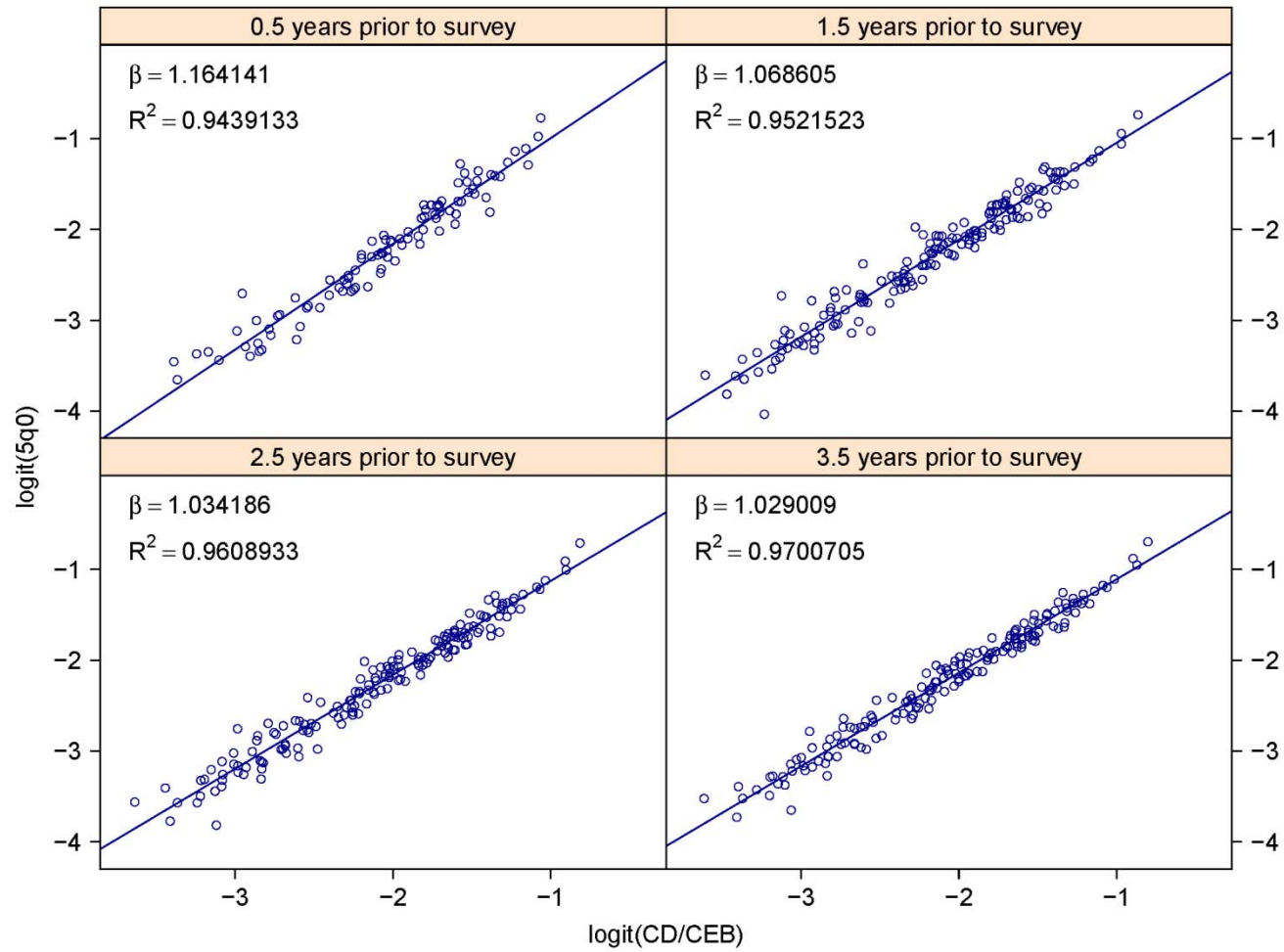

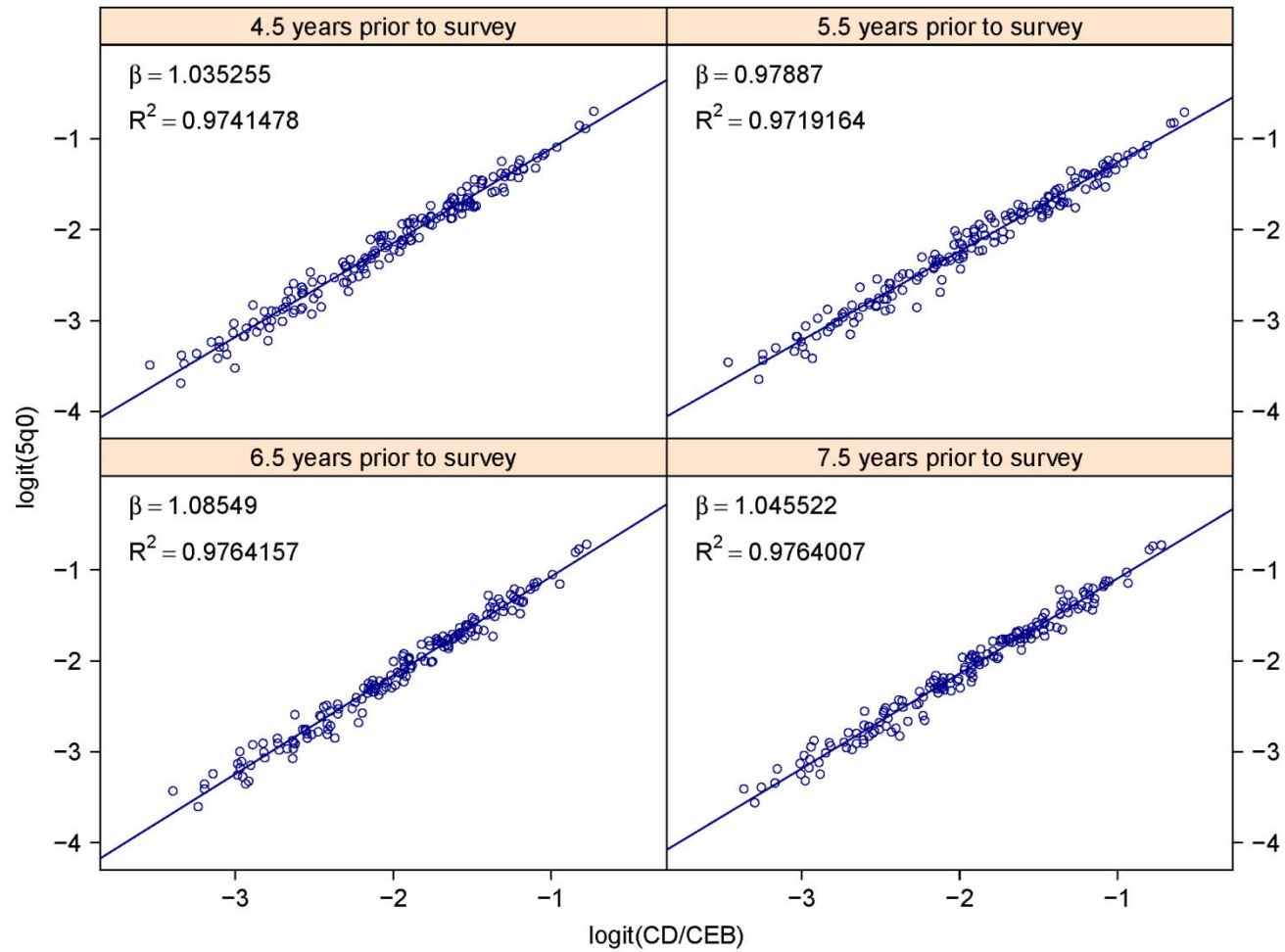

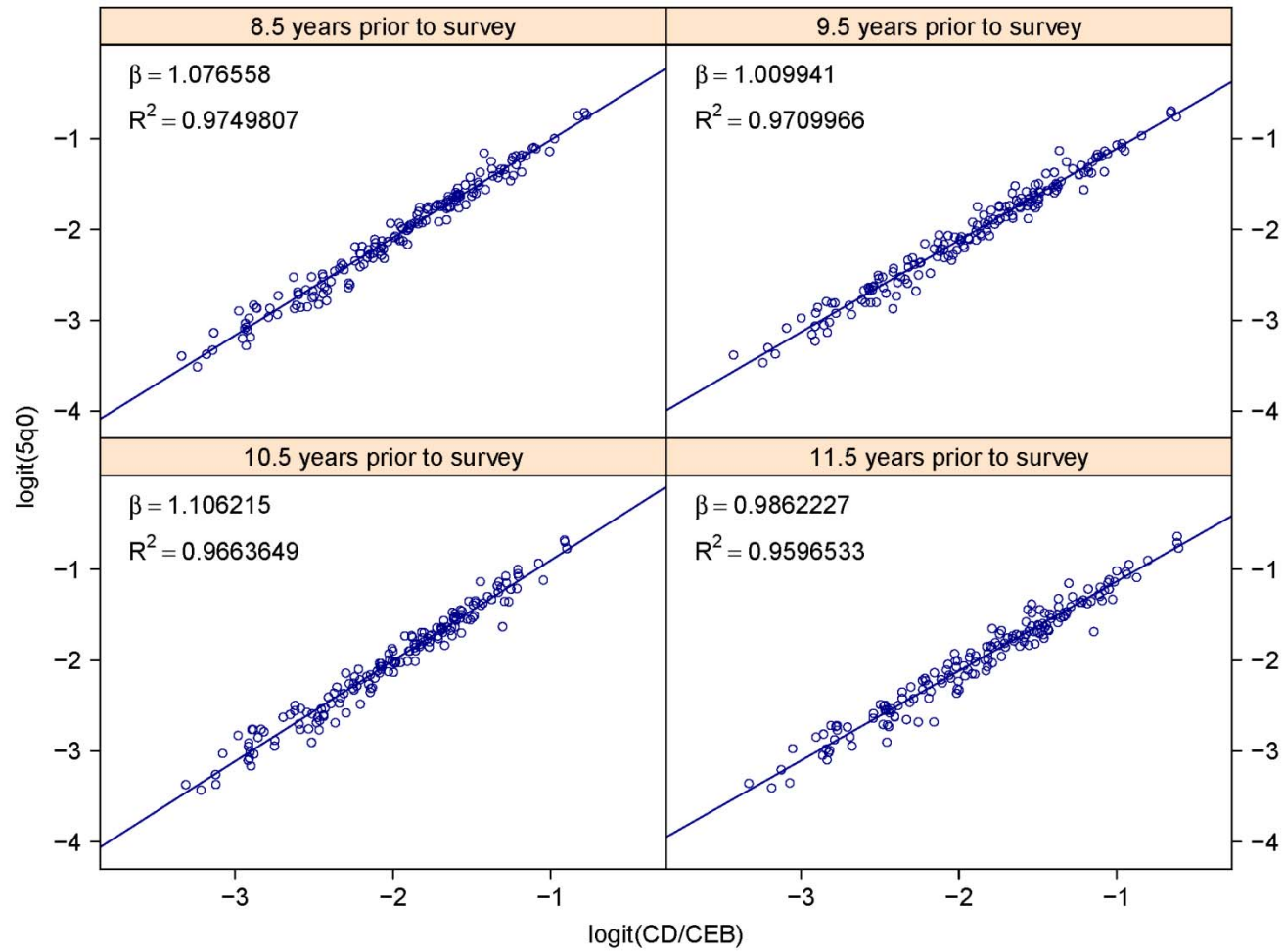

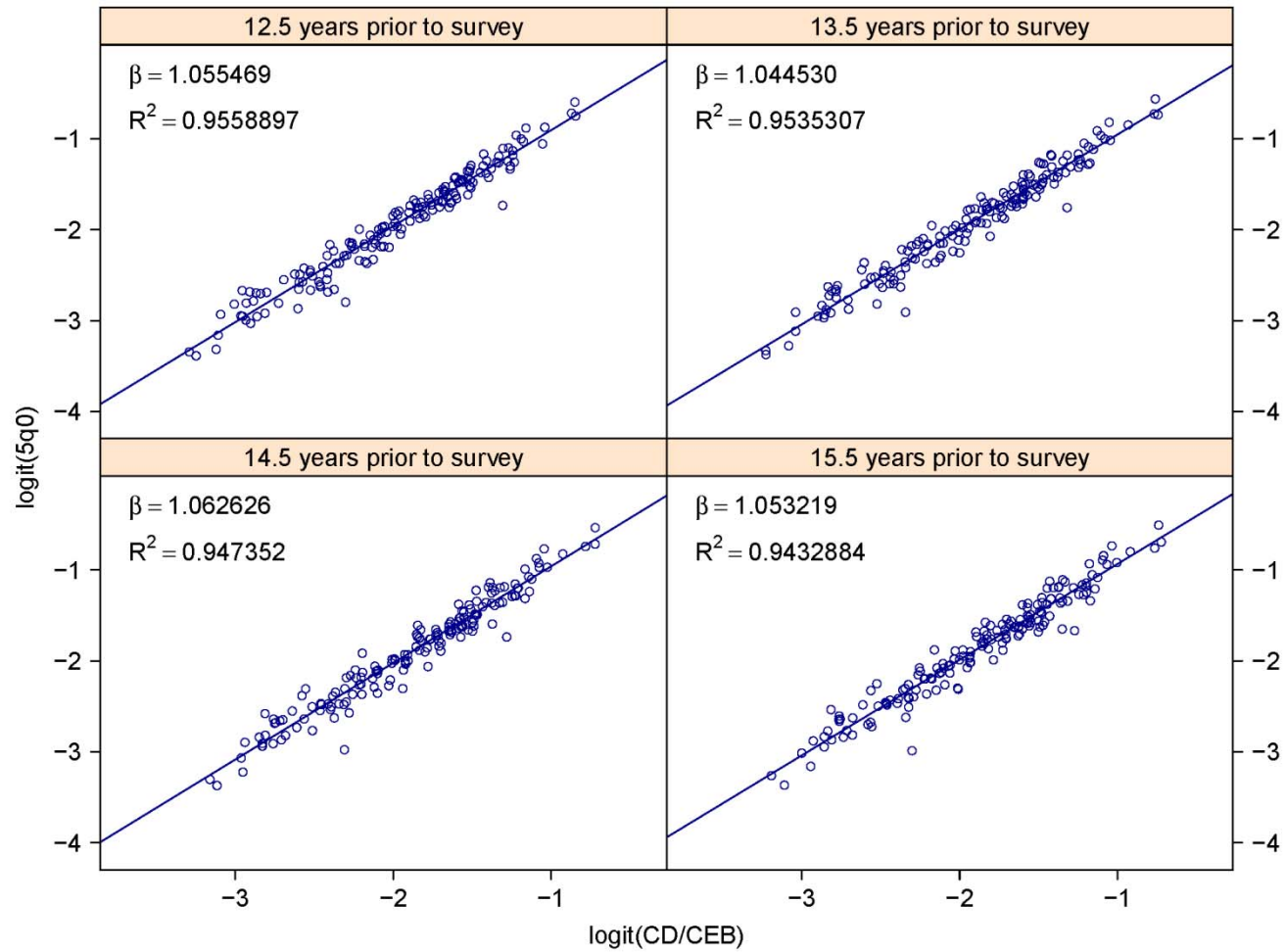

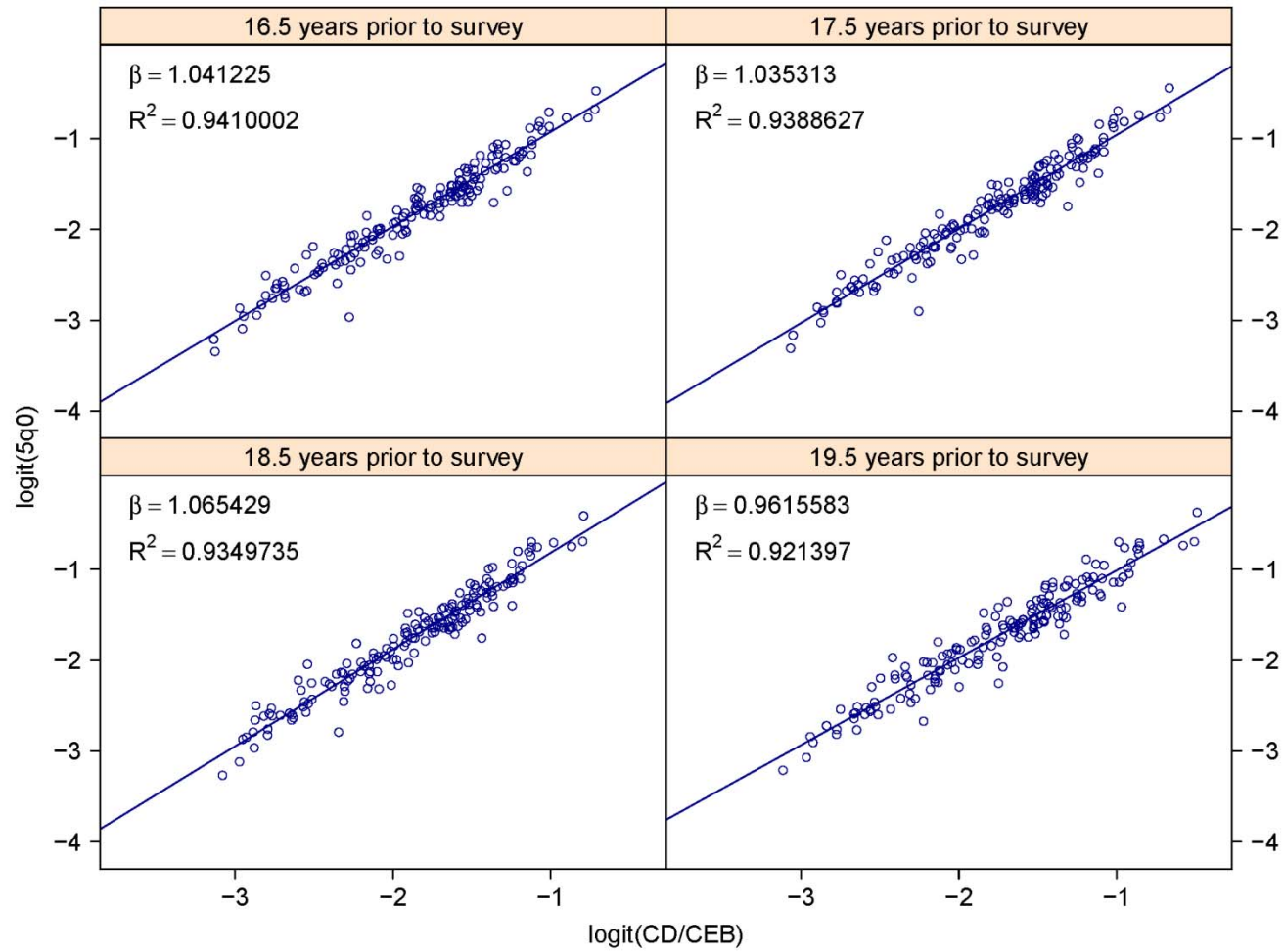

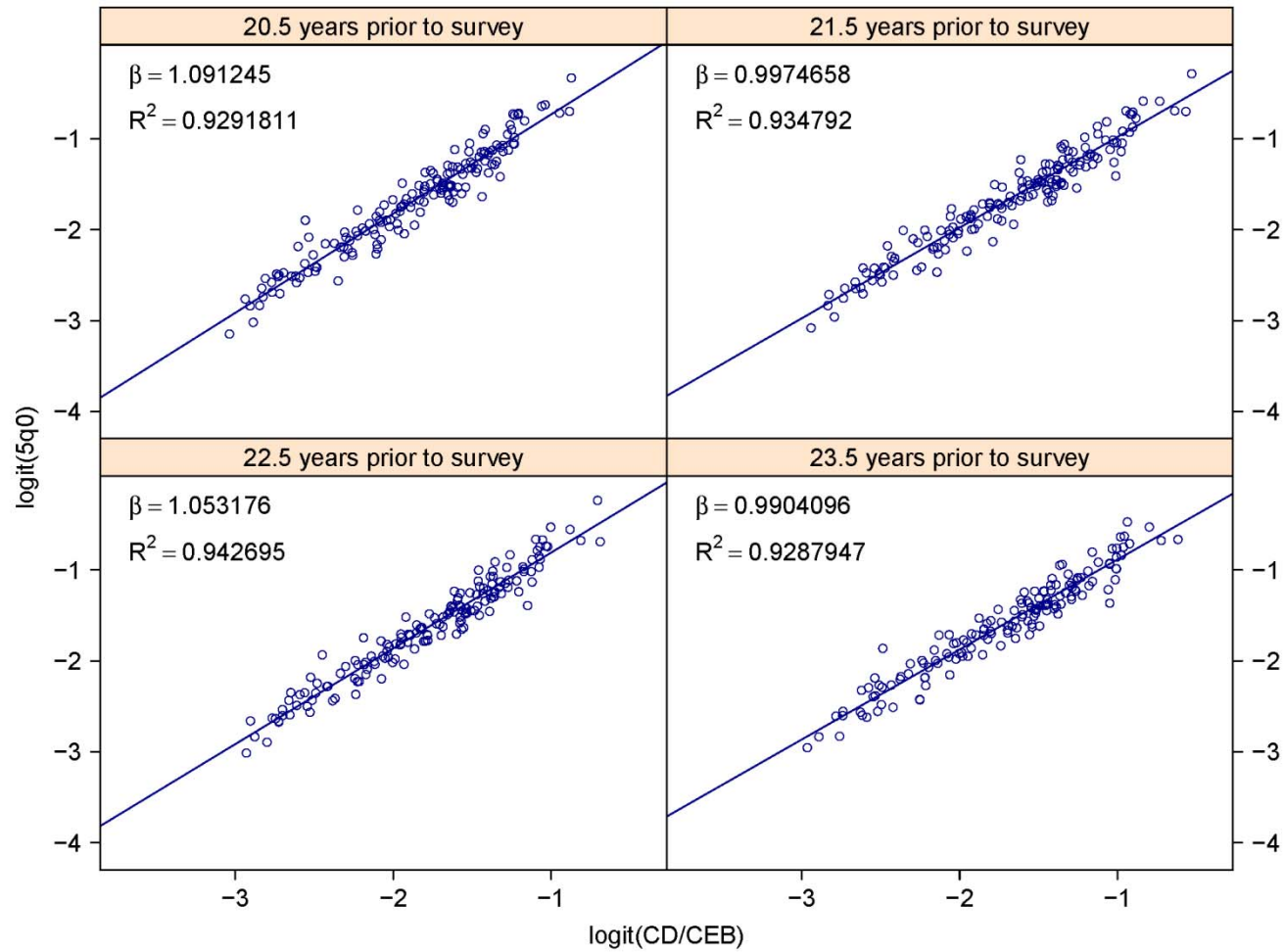

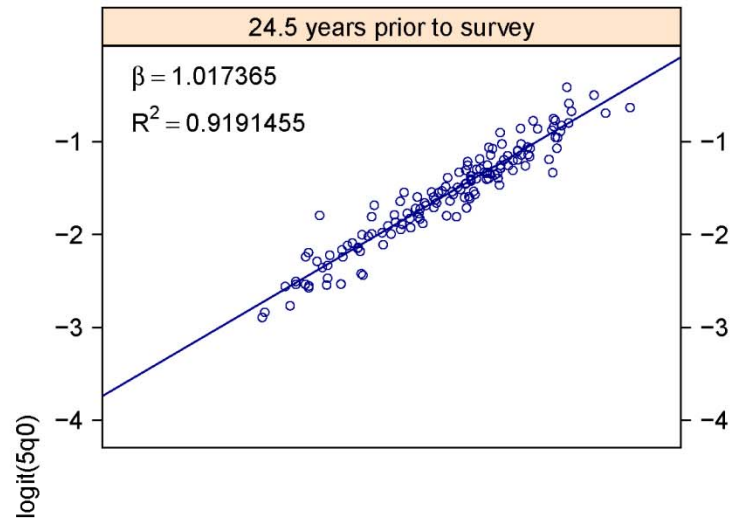

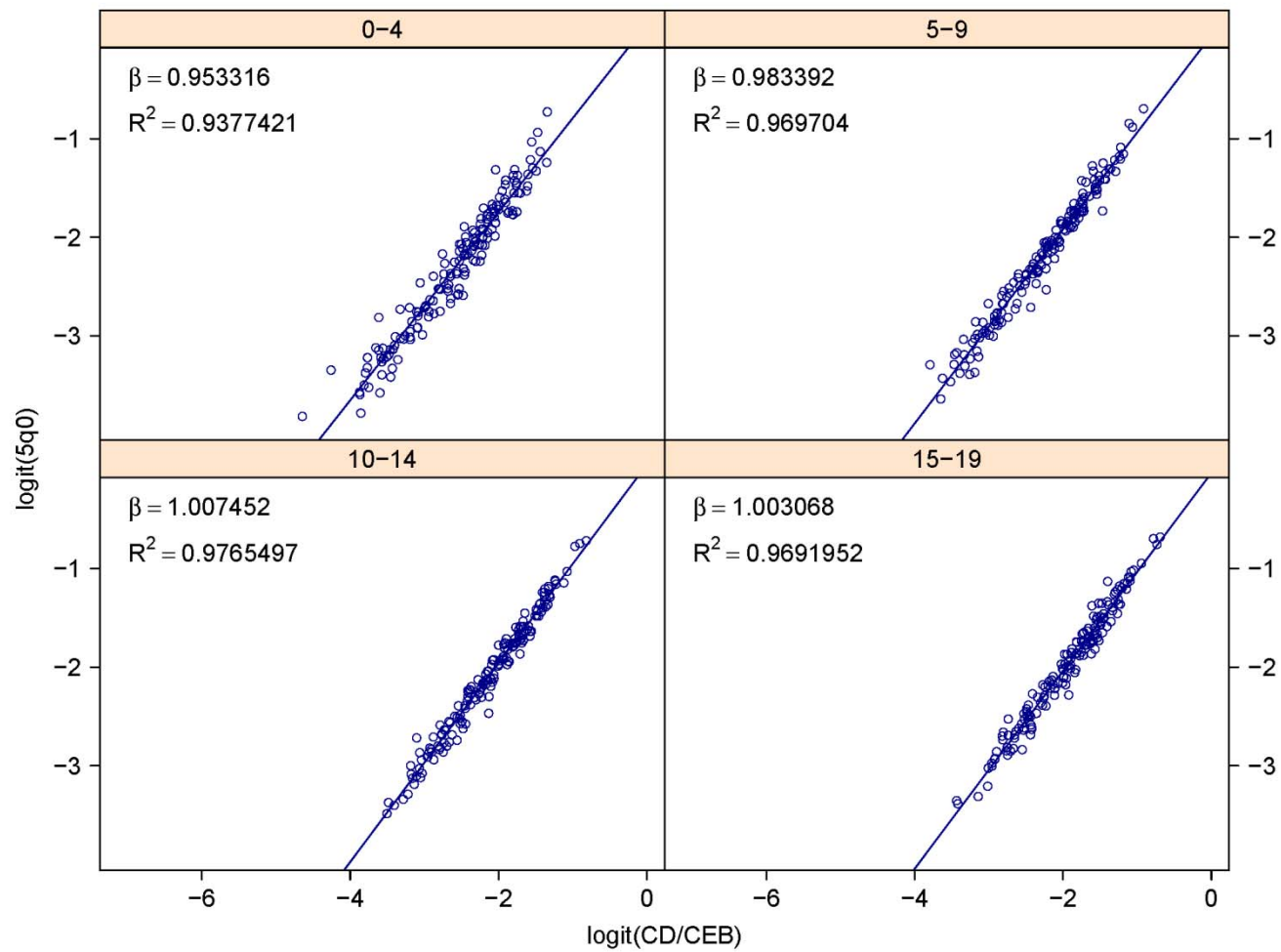

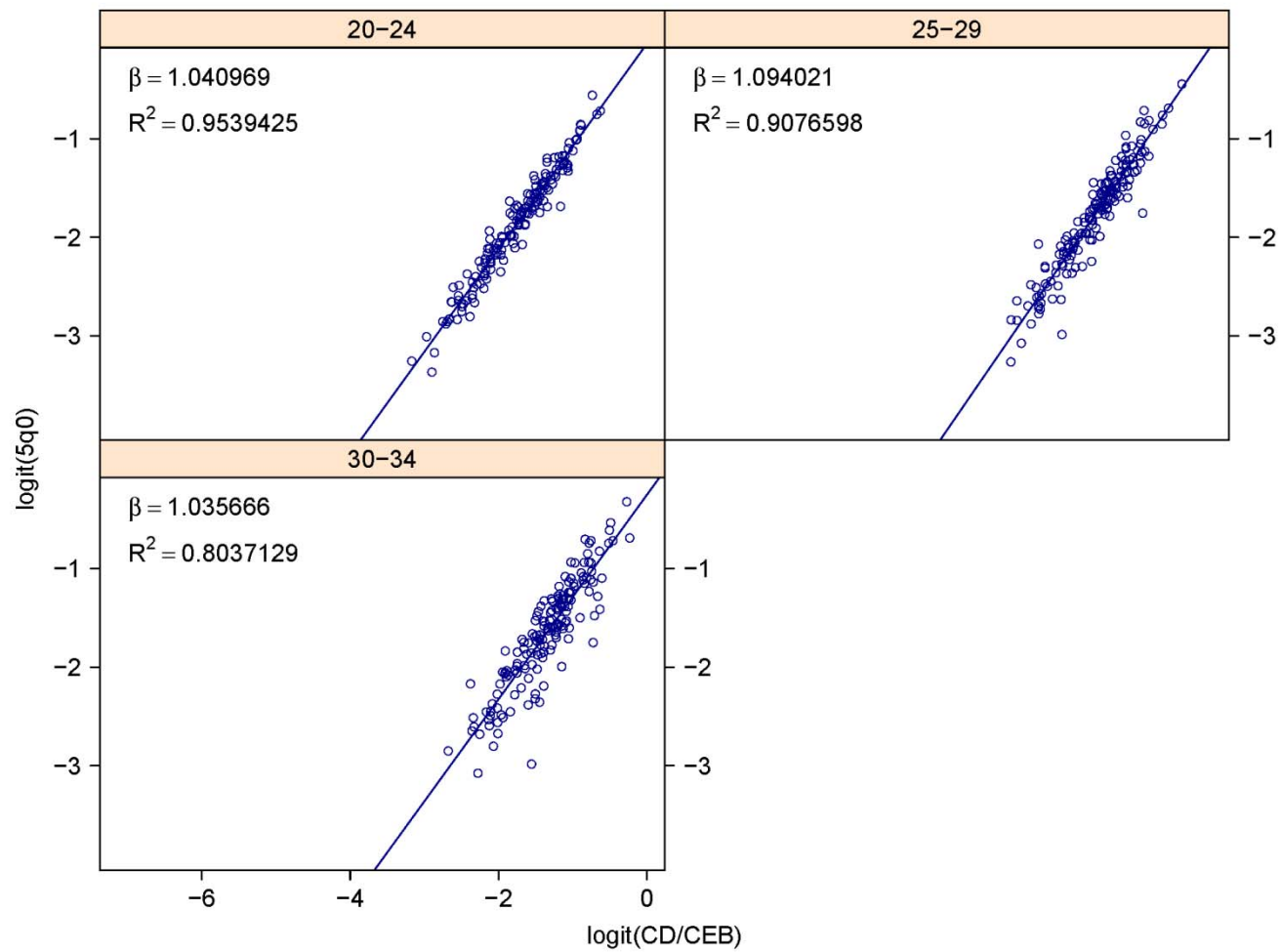

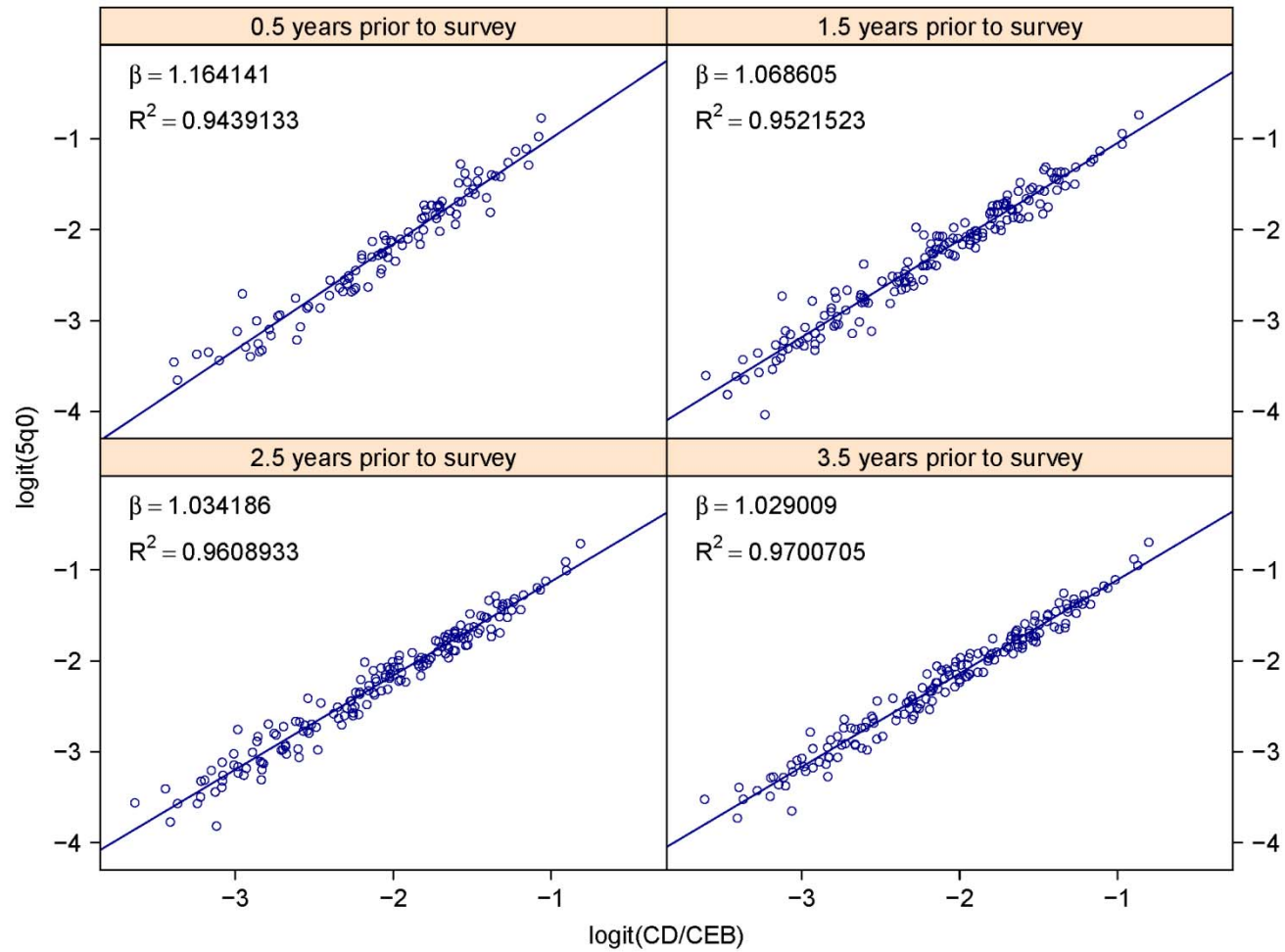

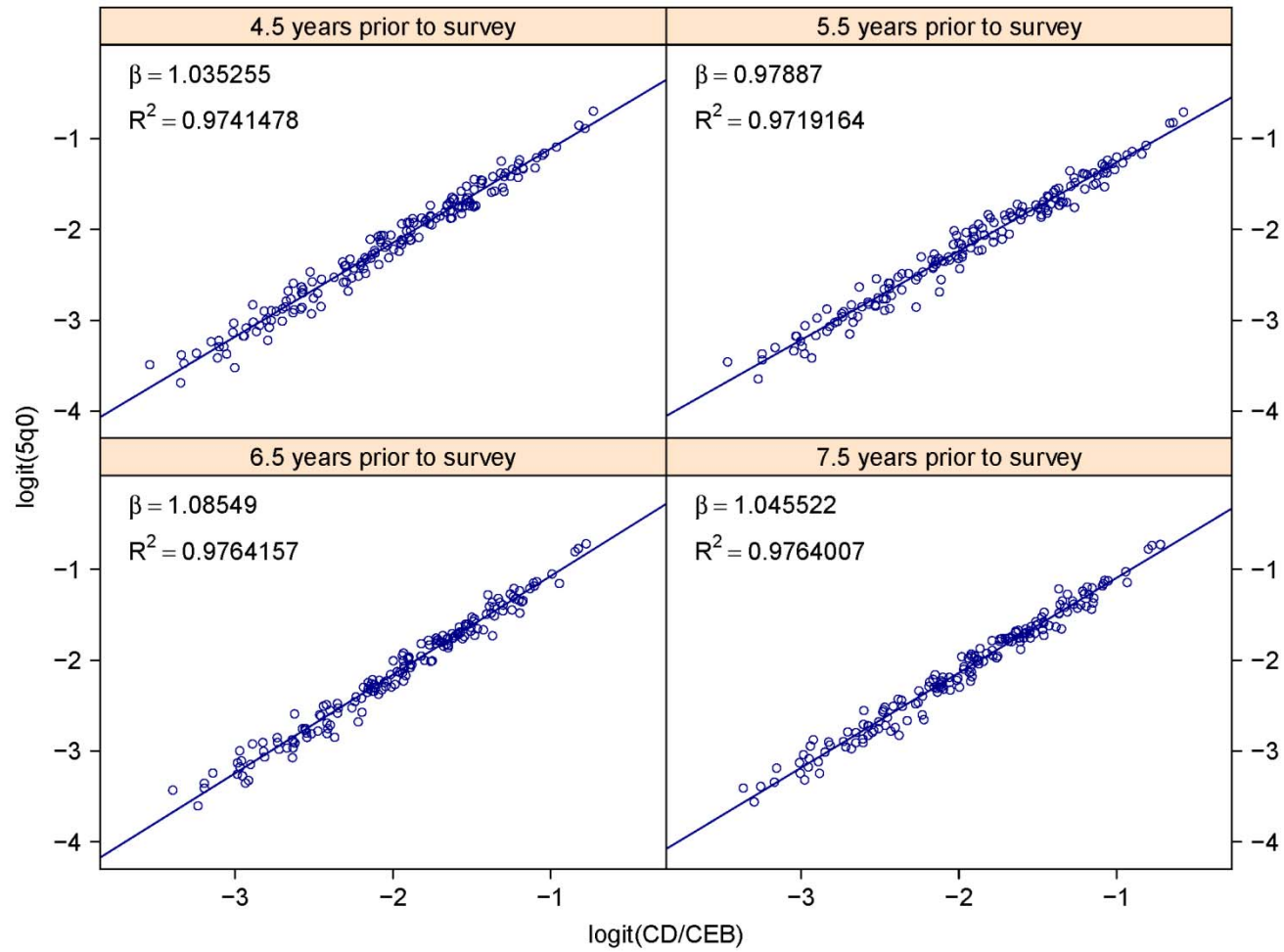

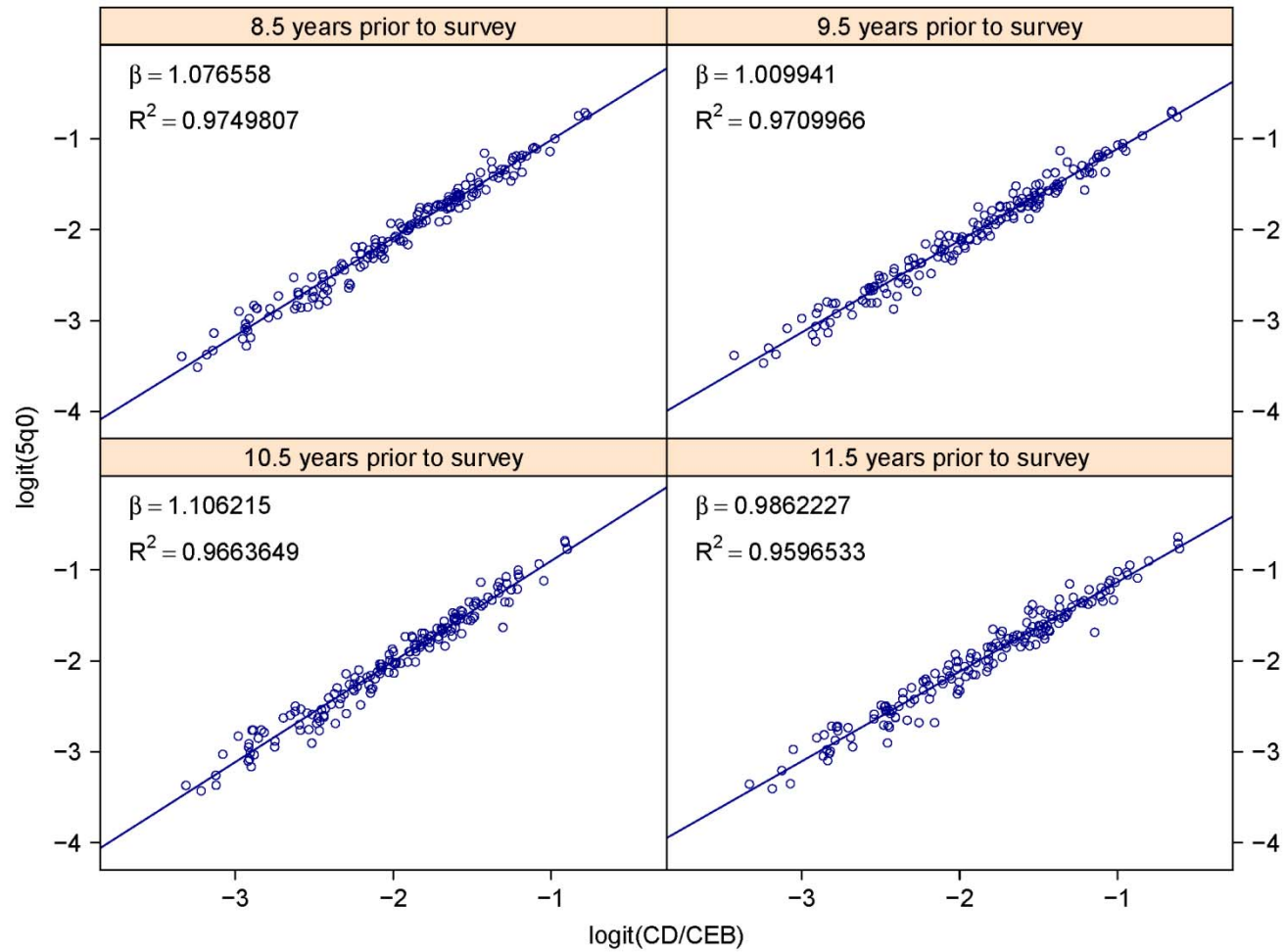

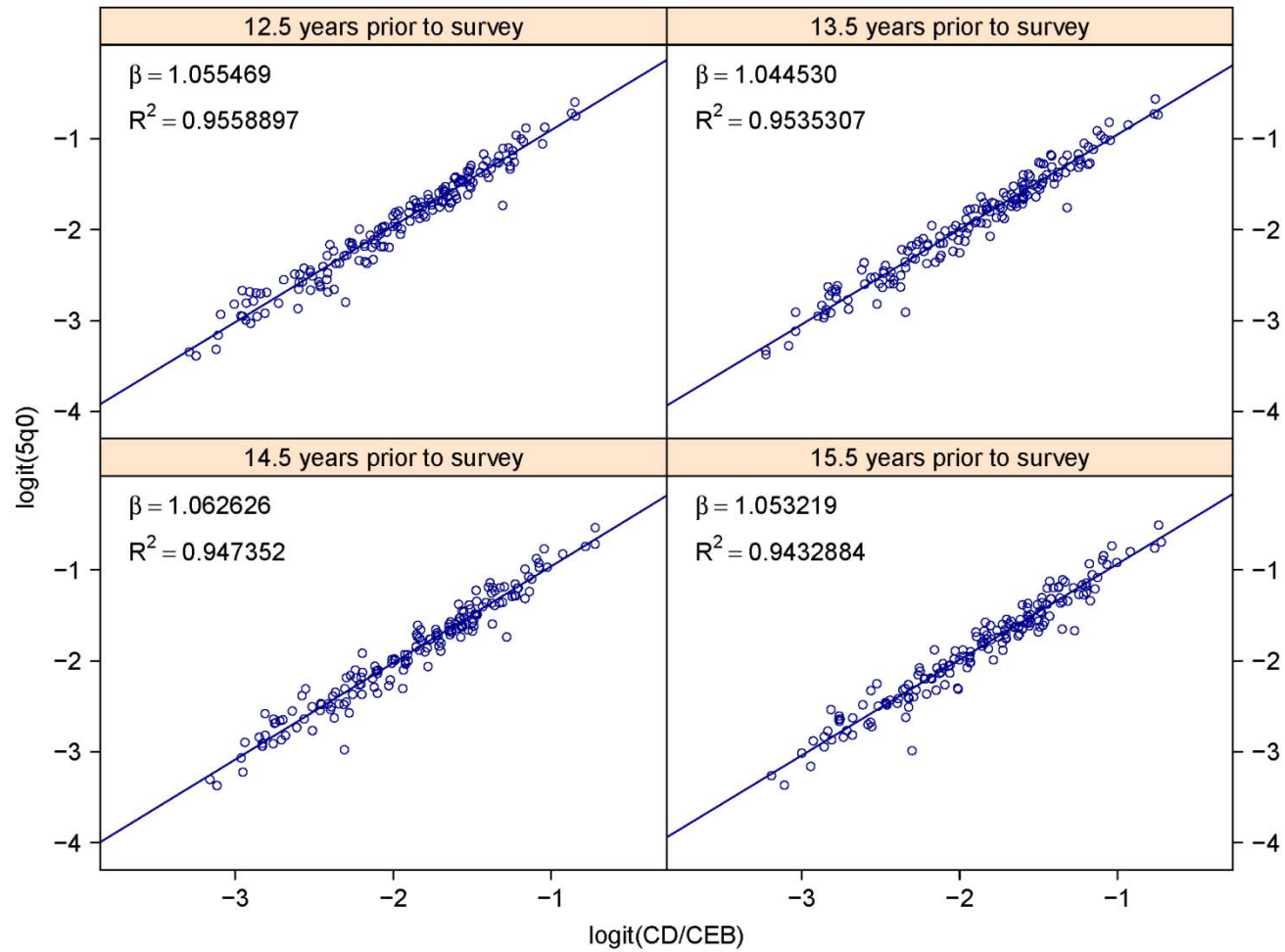

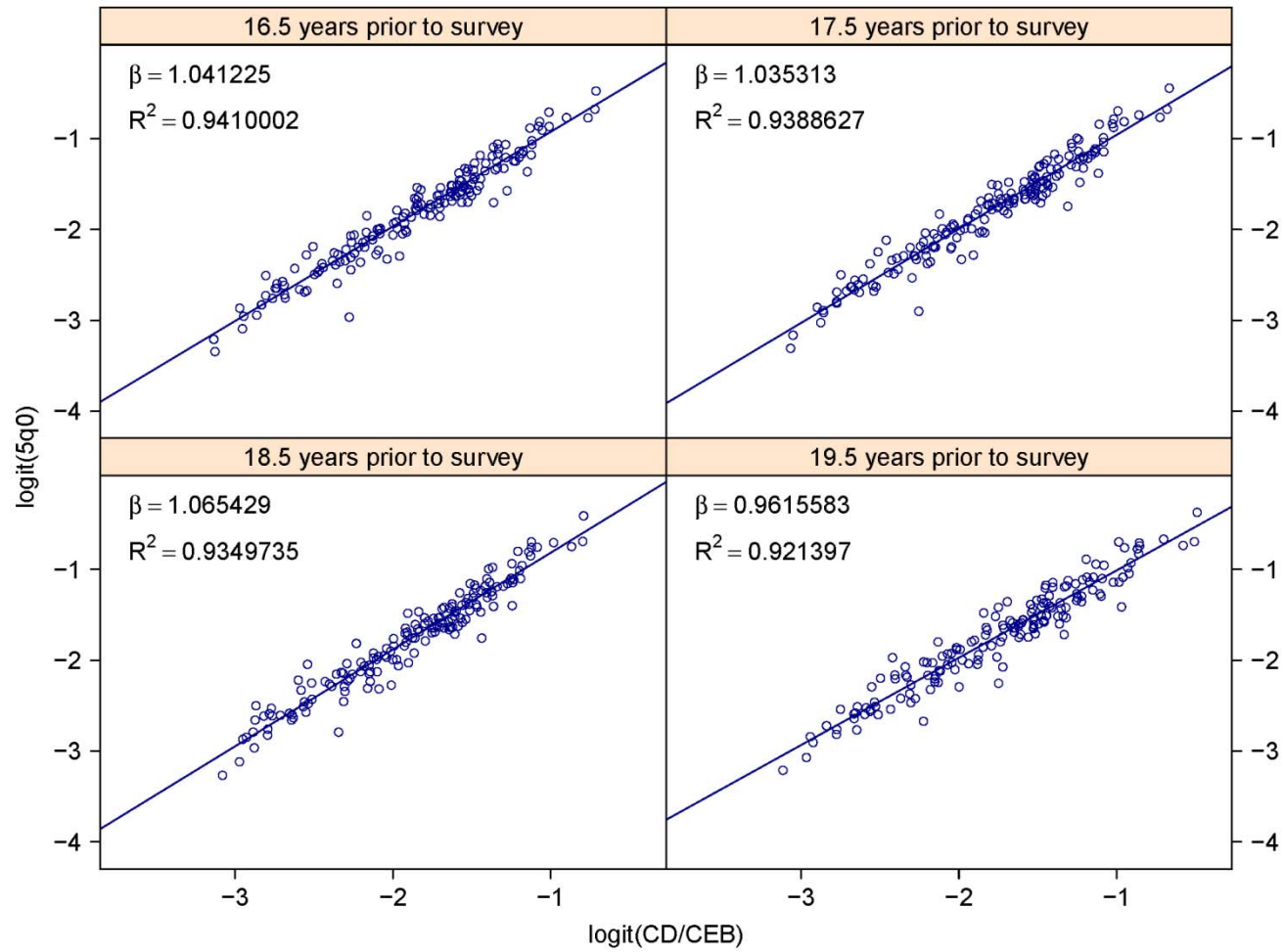

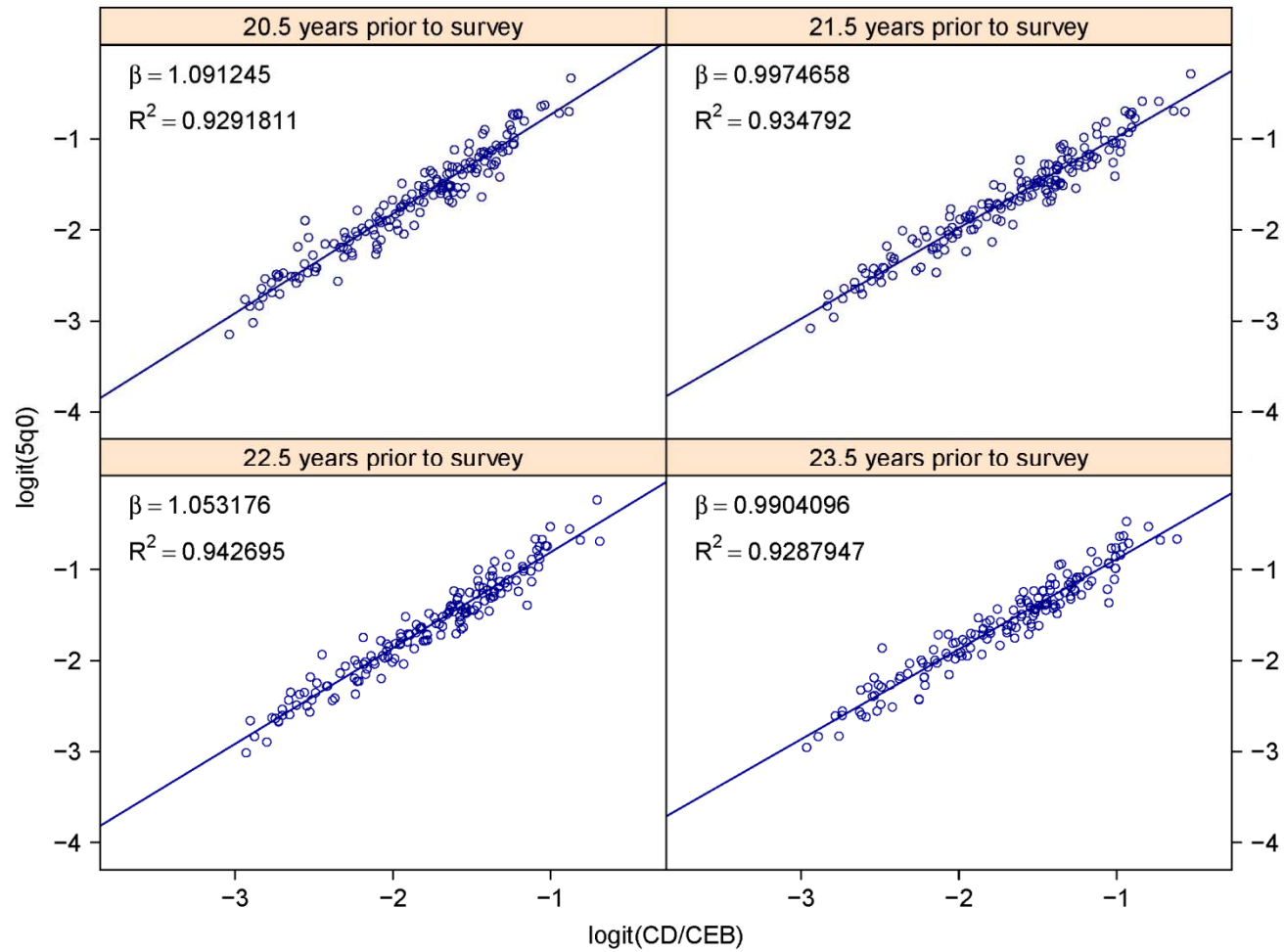

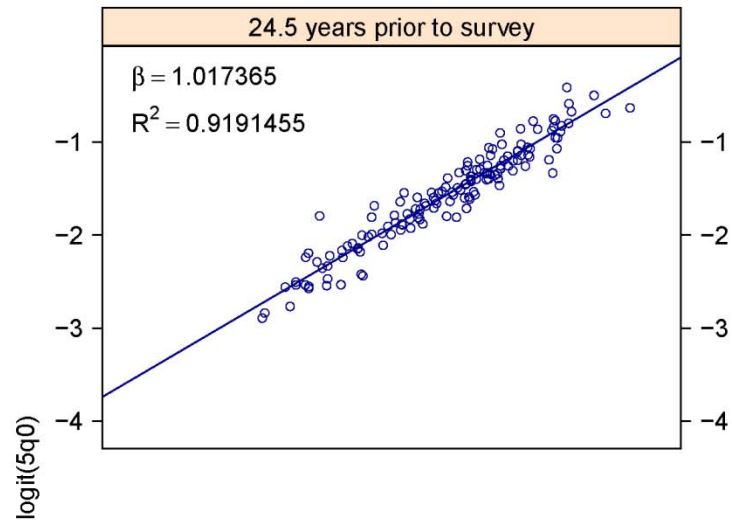

Supplement: Figure S1 — Bivariate relationships between CD/CEB and 5q0 for each age or time-since-first-birth group (for cohort-derived methods) and each year prior to the survey (for period-derived methods). (1.51 MB PDF) [file pmed.1000253.s001.pdf]
