# Supplementary material for: Measuring Under-Five Mortality: Validation of New Low-Cost Methods
Source: PLoS Med. 2010 Apr 13;7(4):e1000253. doi: 10.1371/journal.pmed.1000253 (PMC2854123; doi:10.1371/journal.pmed.1000253)
Supplement: Figure S2 — Results from MAC, MAP, TFBC, TFBP, and combined methods applied to each of 166 DHS. (5.93 MB PDF) [file pmed.1000253.s002.pdf]

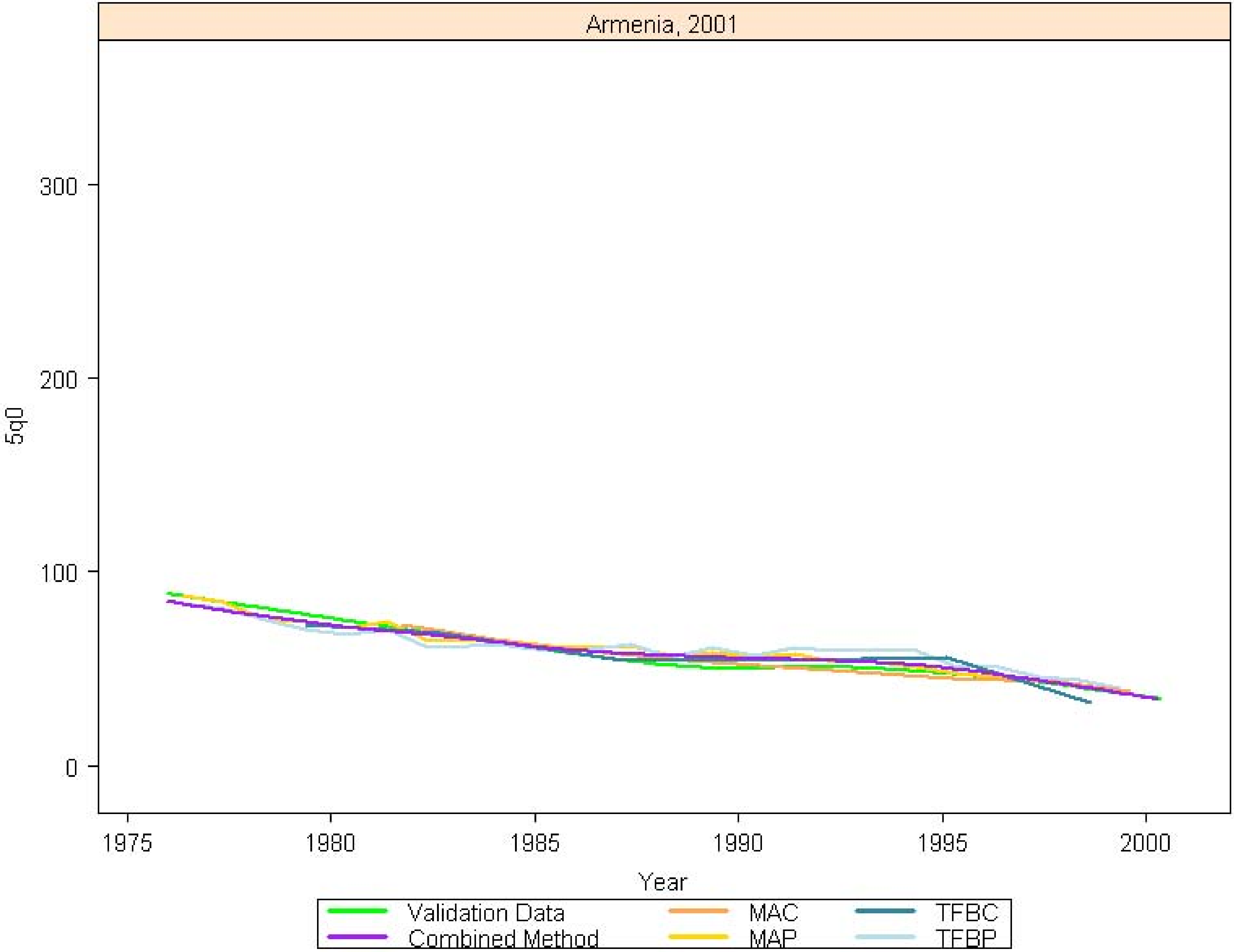
Armenia, 2001
300
200
100
0
5q0
1975
1980
1985
1990
1995
2000
Year
Validation Data
Combined Method
MAC
MAP
TFBC
TFBP

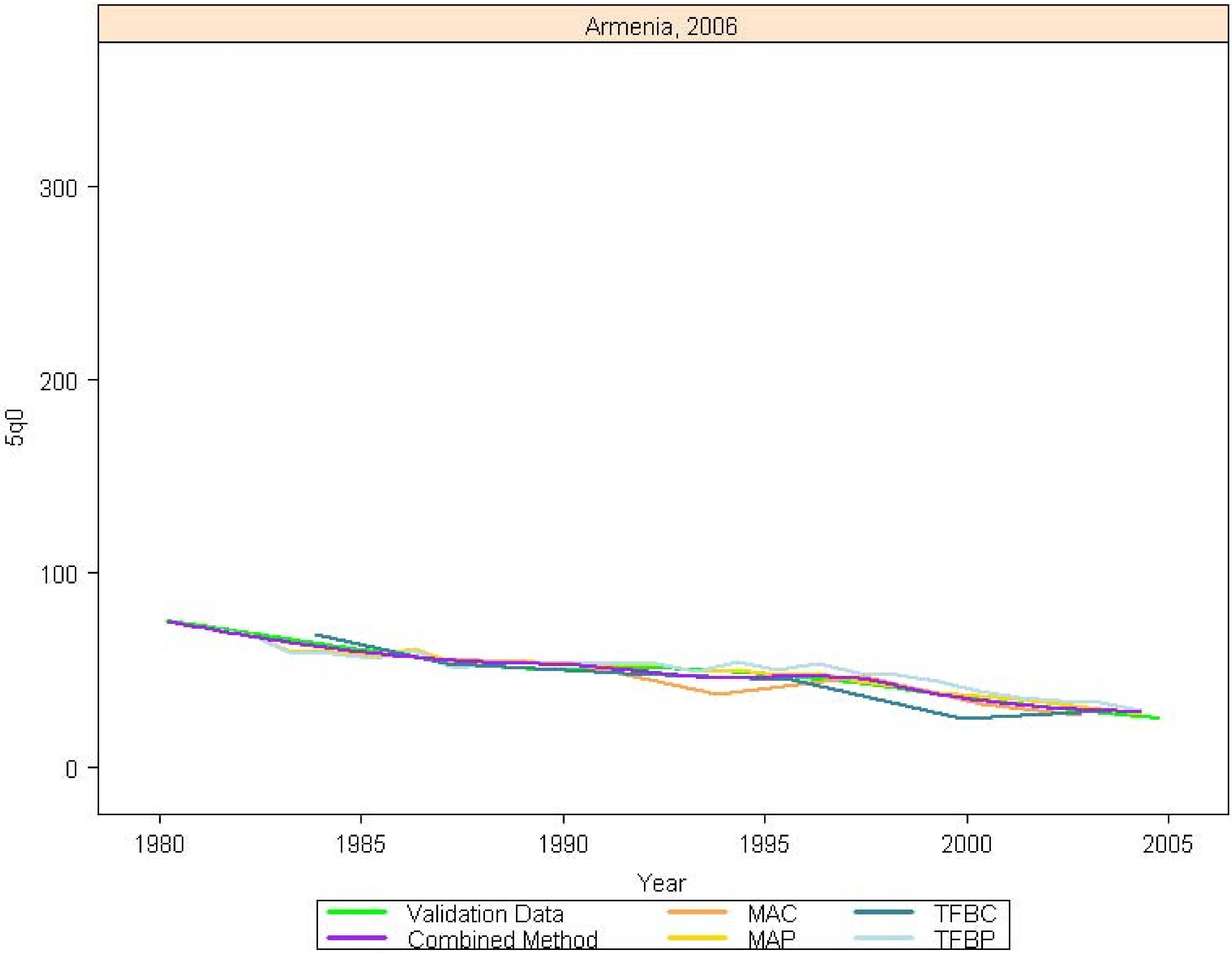
Armenia, 2006
300
200
100
0
5q0
1980
1985
1990
1995
2000
2005
Year
Validation Data
Combined Method
MAC
MAP
TFBC
TFBP

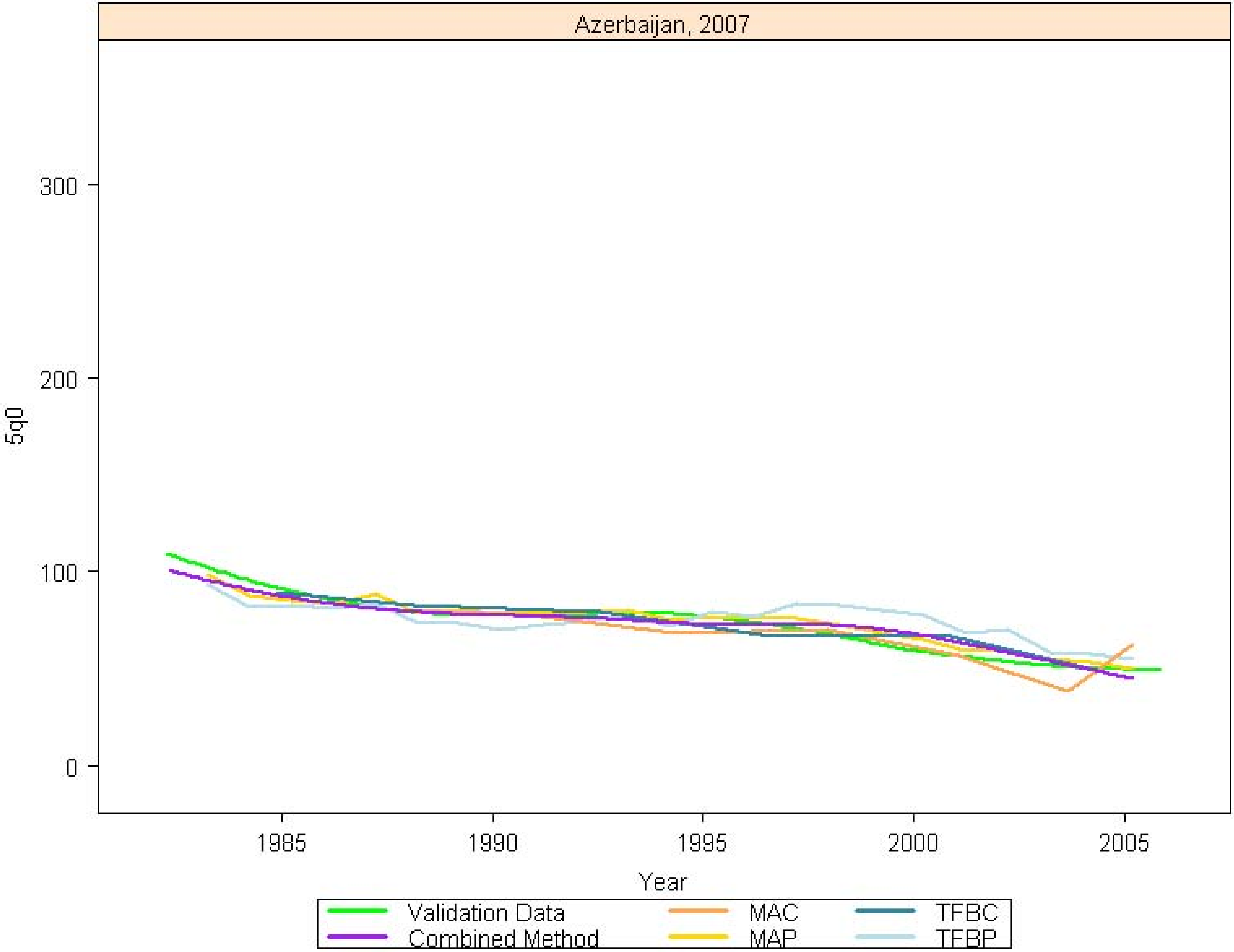

Azerbaijan, 2007
300
200
100
0
5q0
1985
1990
1995
2000
2005
Year
Validation Data
Combined Method
MAC
MAP
TFBC
TFBP

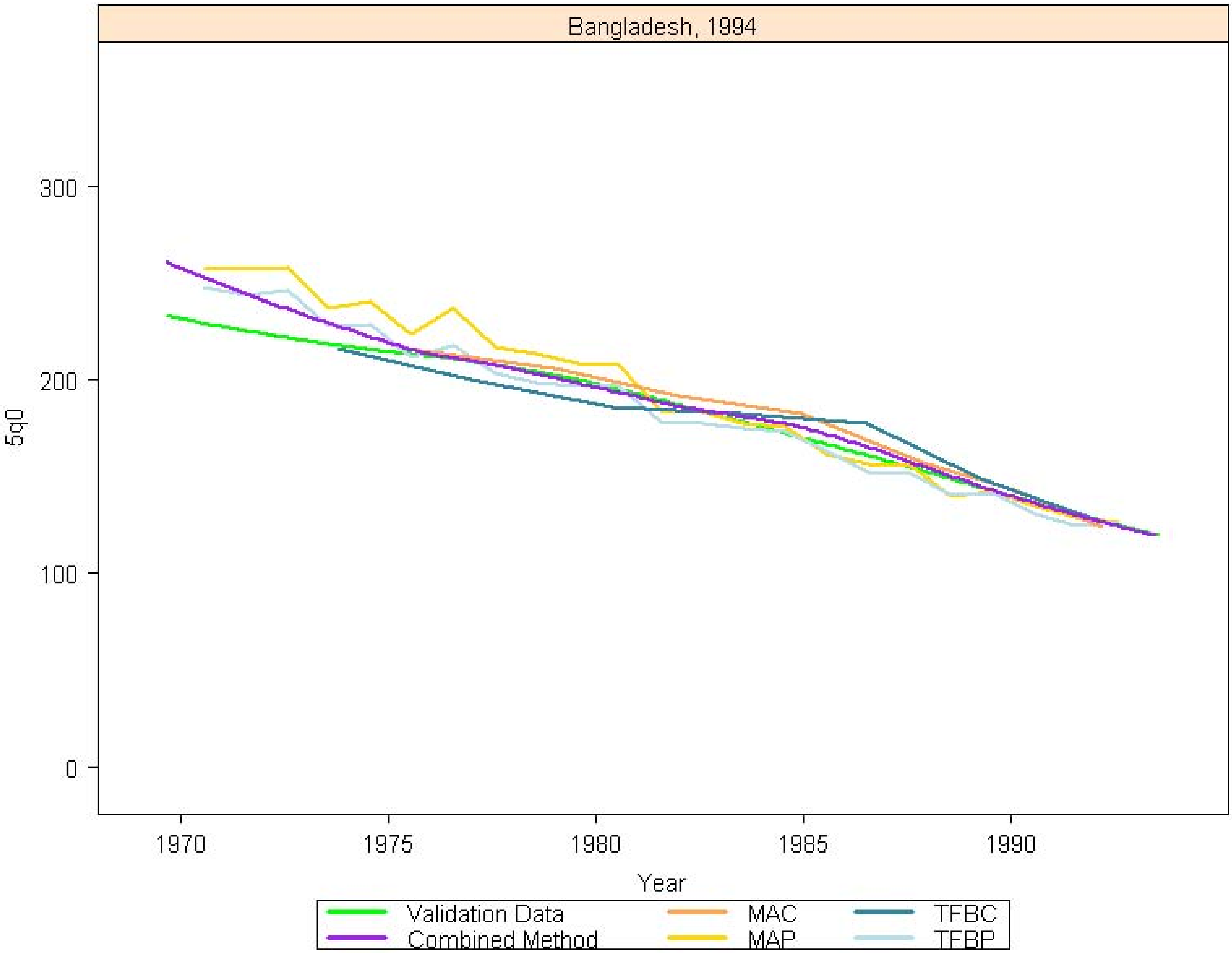
Bangladesh, 1994
300
200
100
0
5q0
1970
1975
1980
1985
1990
Year
Validation Data
Combined Method
MAC
MAP
TFBC
TFBP

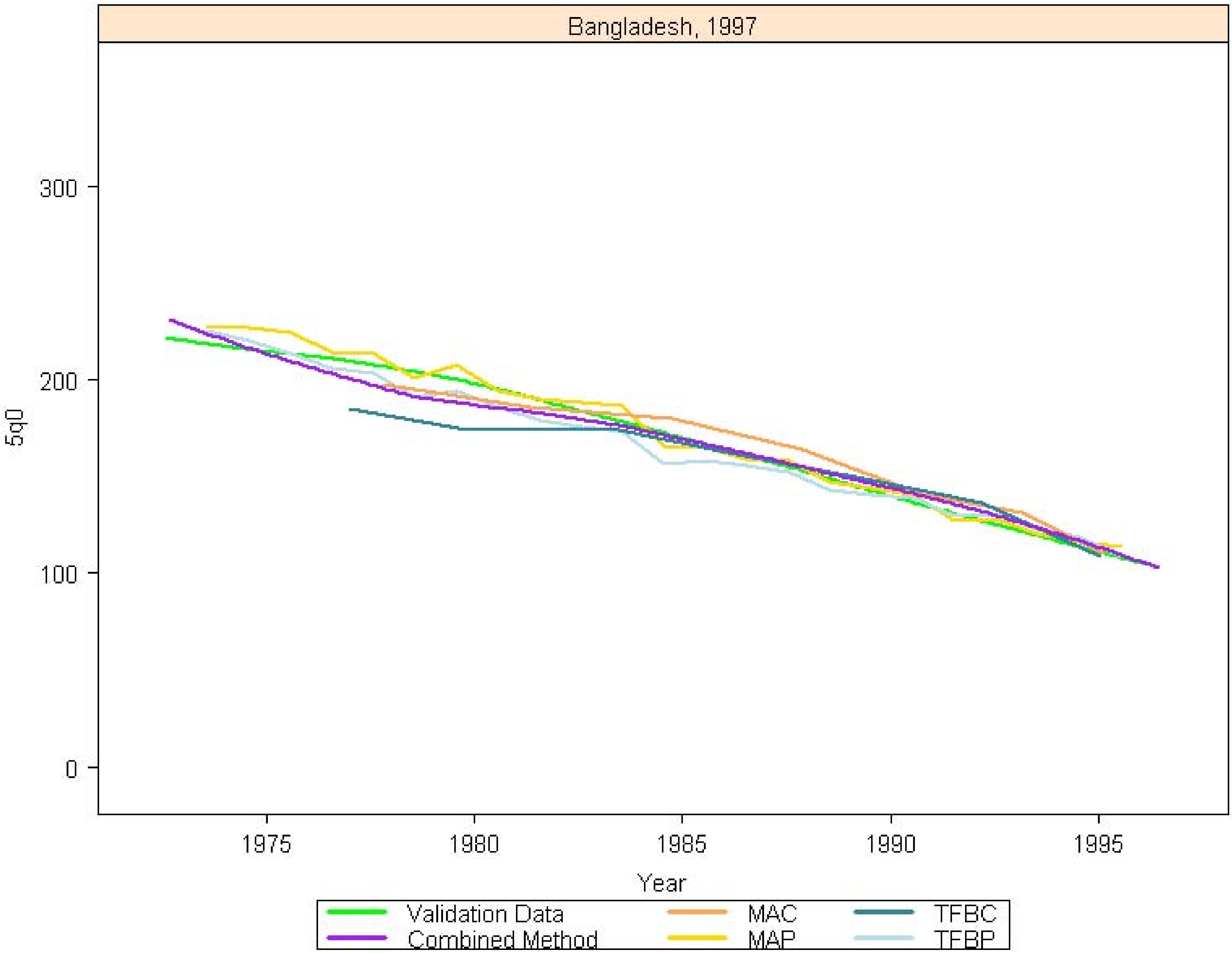
Bangladesh, 1997
300
200
100
0
5q0
1975
1980
1985
1990
1995
Year
Validation Data
Combined Method
MAC
MAP
TFBC
TFBP

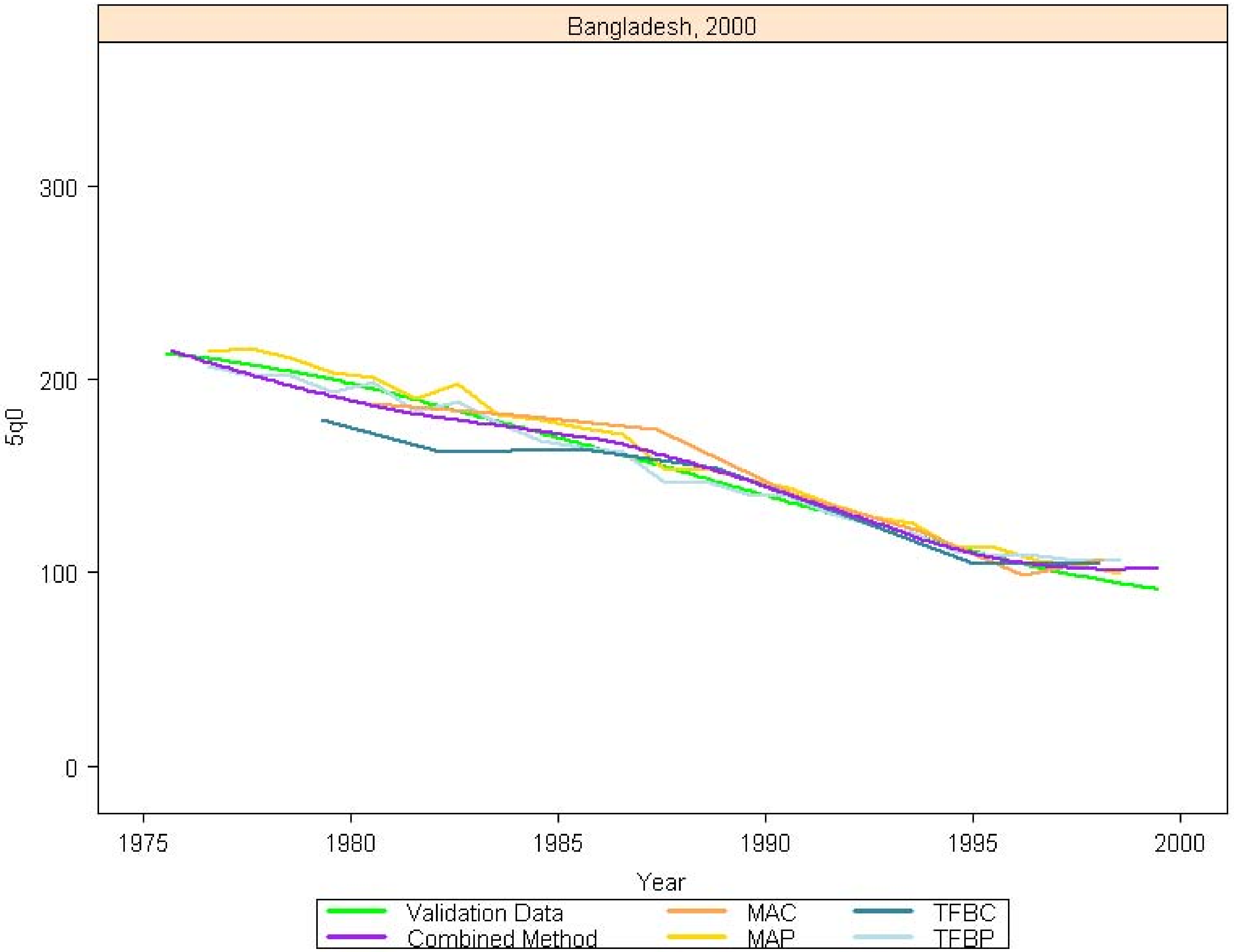
Bangladesh, 2000
300
200
100
0
5q0
1975
1980
1985
1990
1995
2000
Year
Validation Data
Combined Method
MAC
MAP
TFBC
TFBP

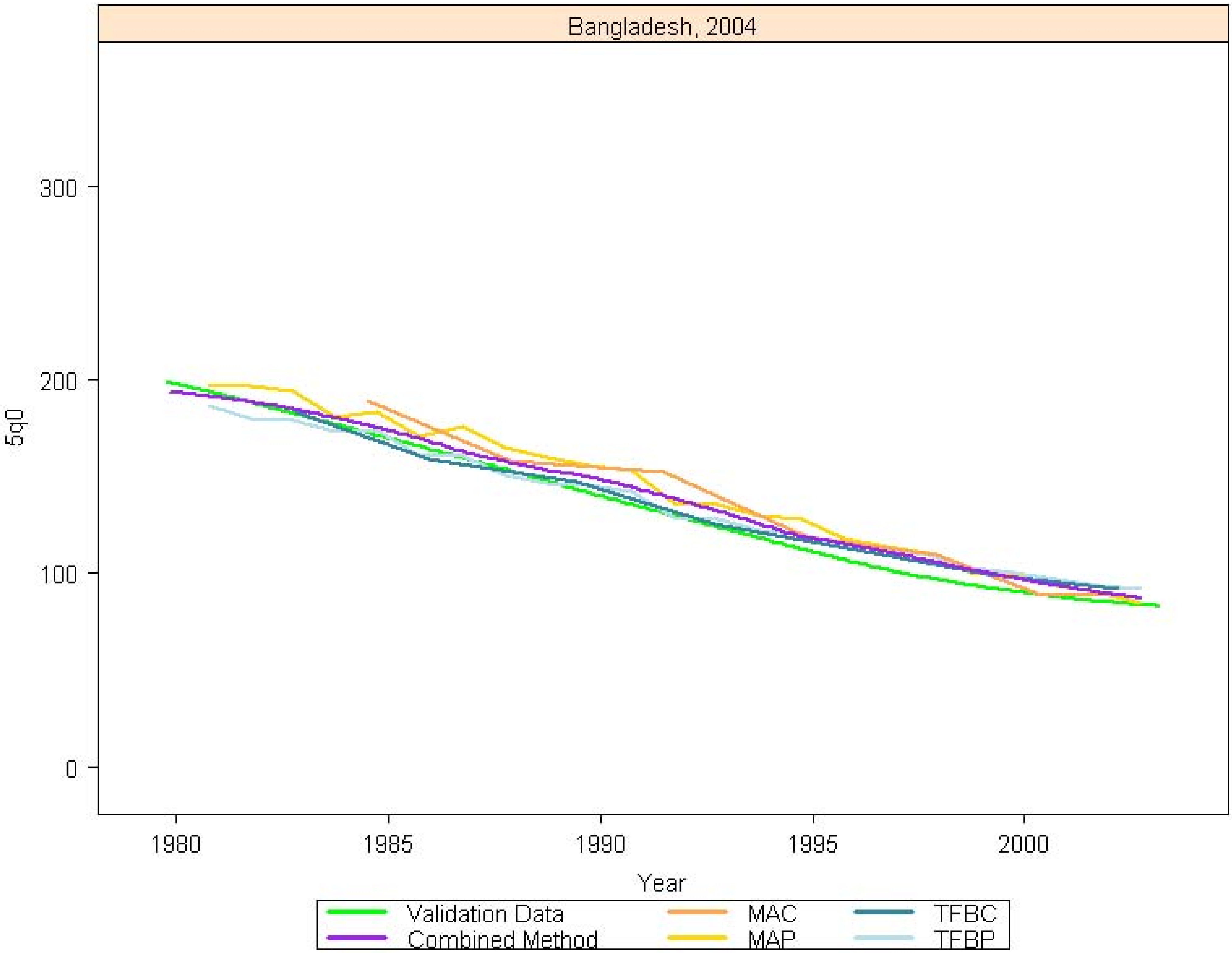

Bangladesh, 2004
300
200
100
0
5q0
1980
1985
1990
1995
2000
Year
Validation Data
Combined Method
MAC
MAP
TFBC
TFBP

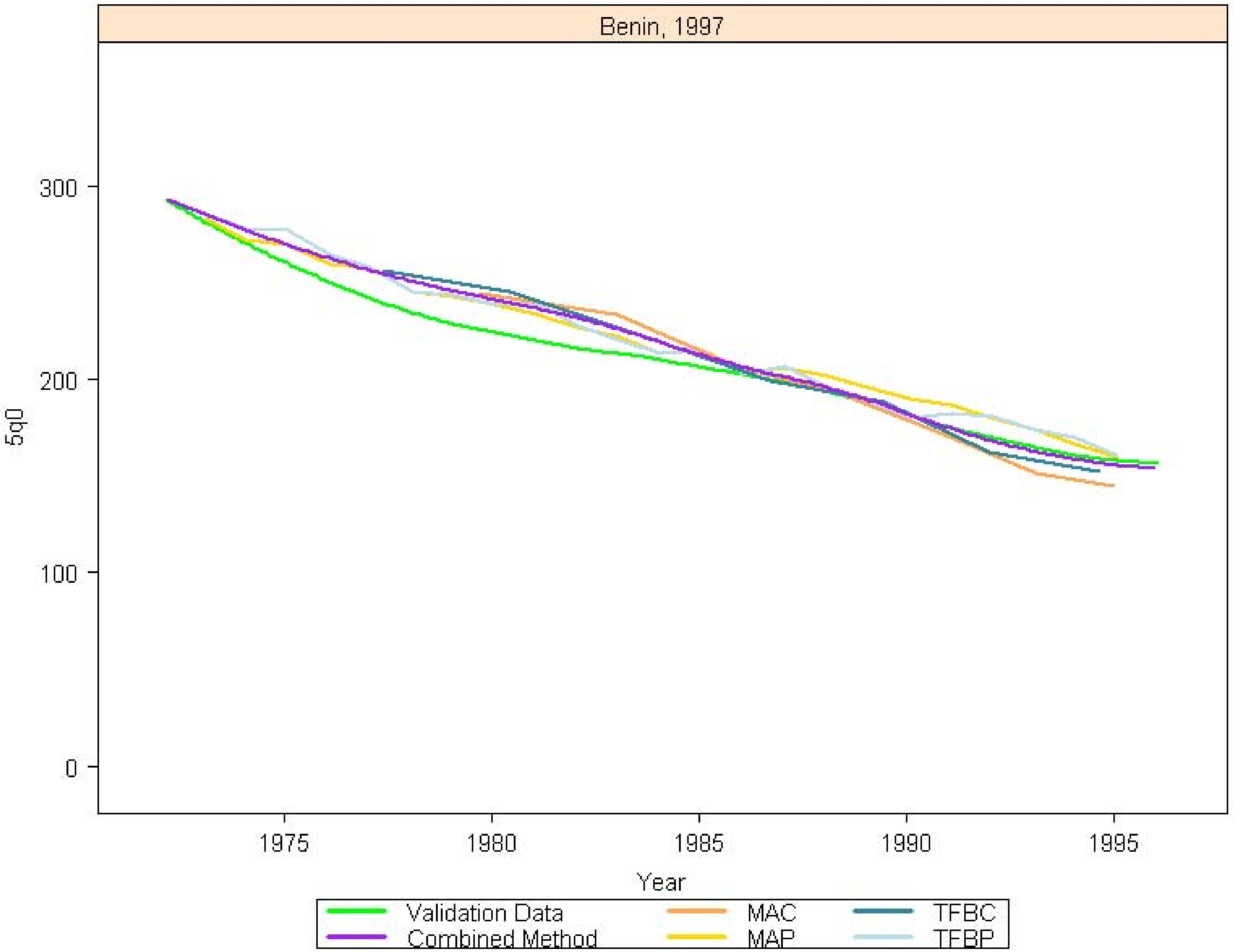
Benin, 1997
300
200
100
0
5q0
1975
1980
1985
1990
1995
Year
Validation Data
Combined Method
MAC
MAP
TFBC
TFBP

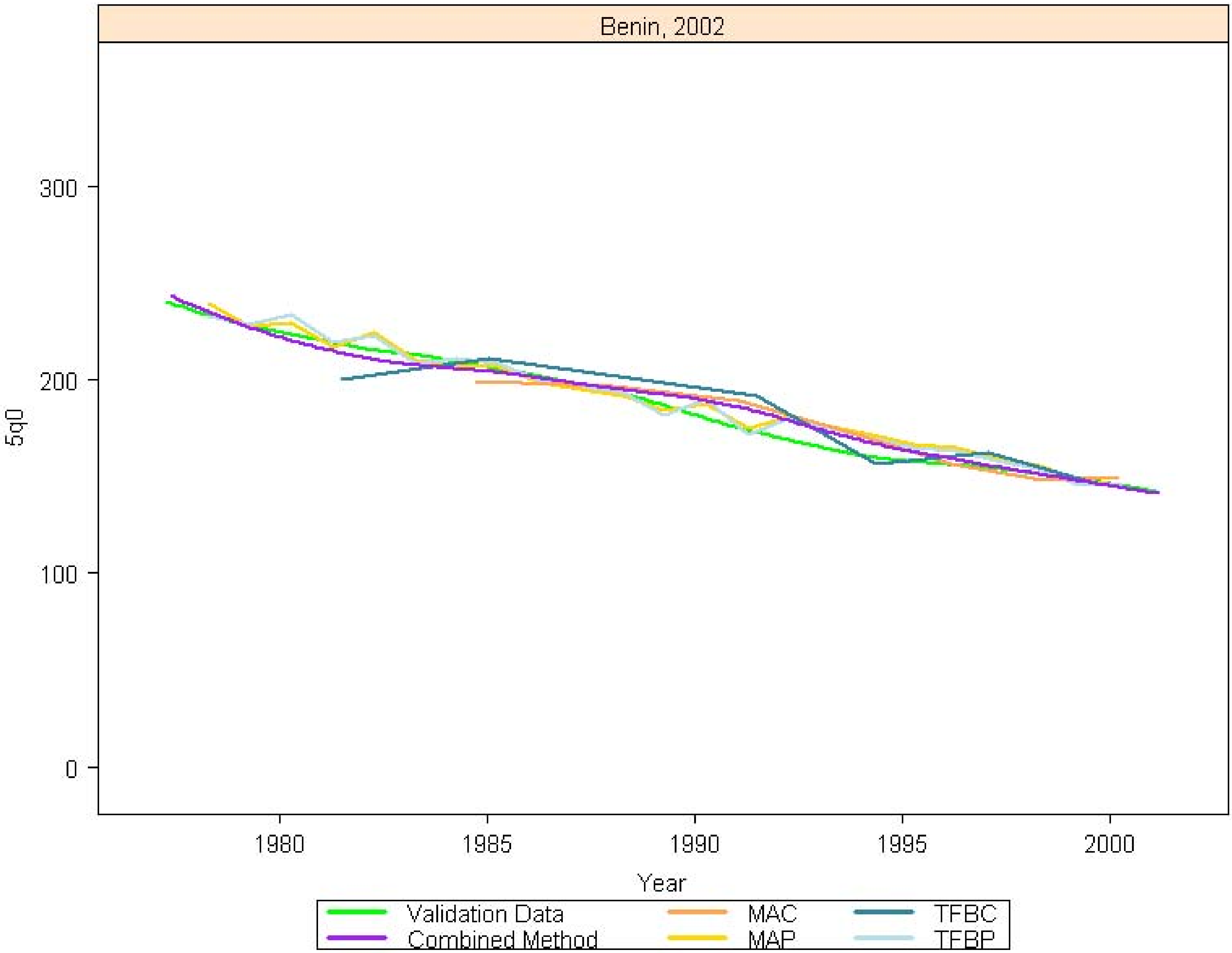

Benin, 2002
300
200
100
0
5q0
1980
1985
1990
1995
2000
Year
Validation Data
Combined Method
MAC
MAP
TFBC
TFBP

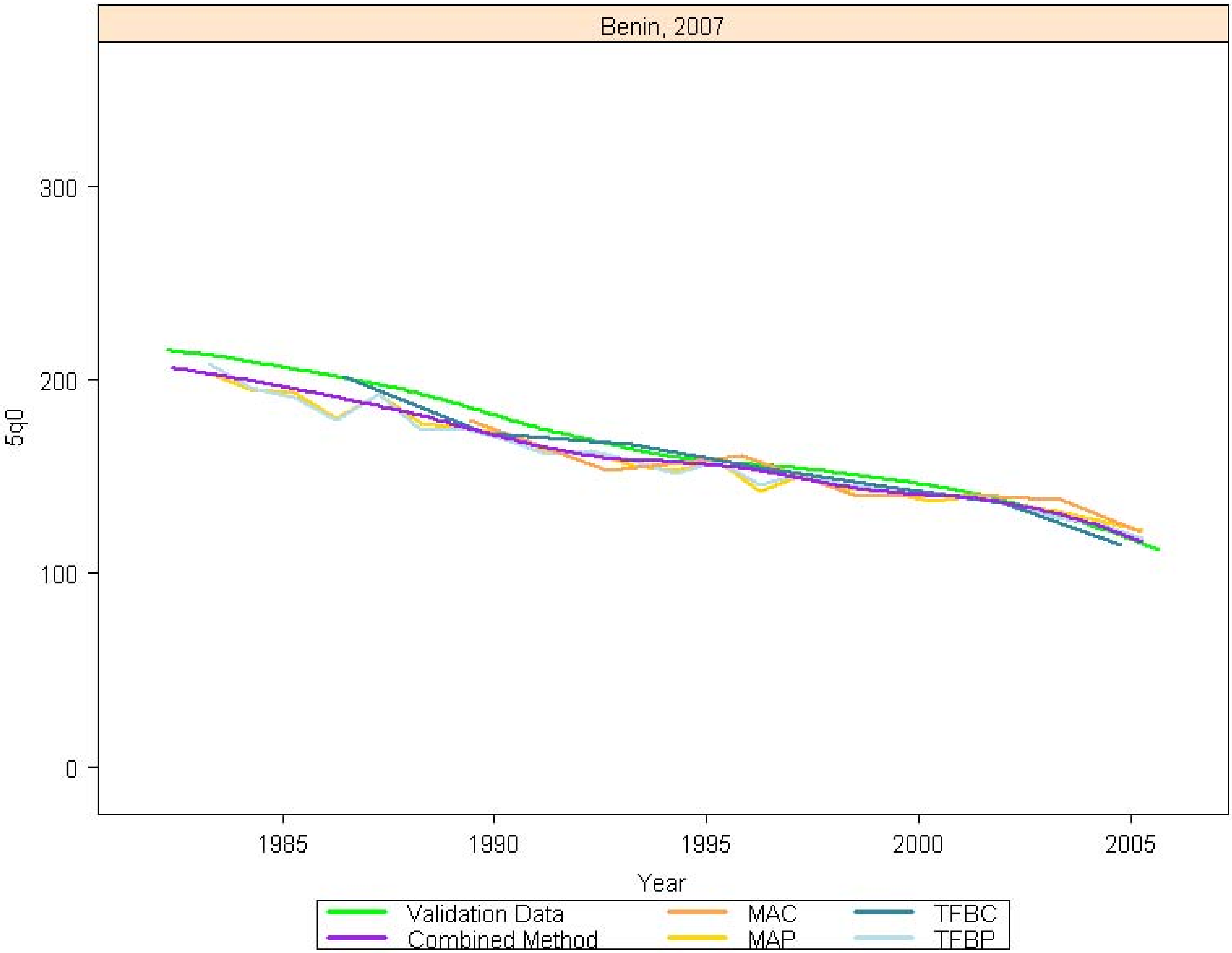

Benin, 2007
300
200
100
0
5q0
1985
1990
1995
2000
2005
Year
Validation Data
Combined Method
MAC
MAP
TFBC
TFBP

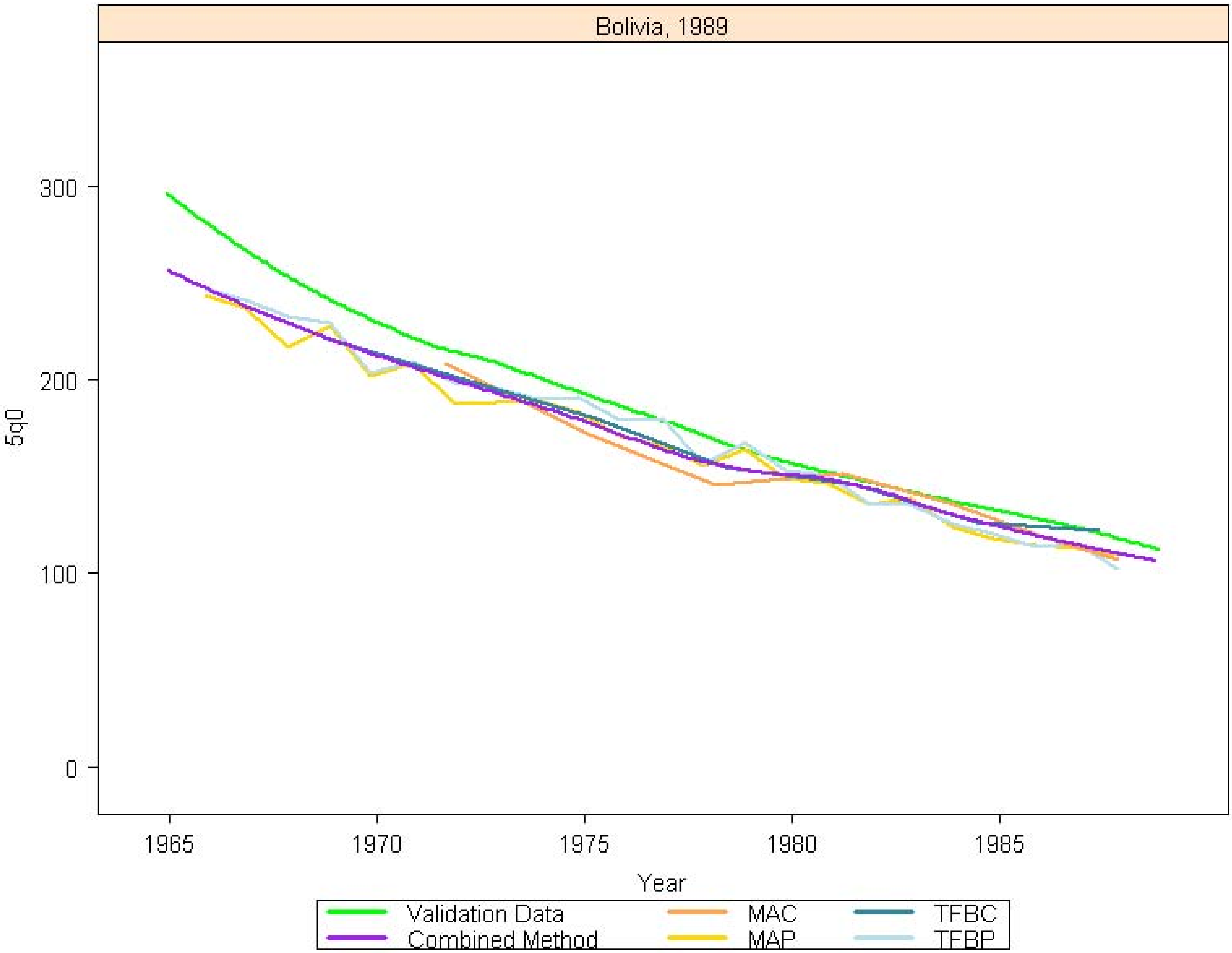
Bolivia, 1989
300
200
100
0
5q0
1965
1970
1975
1980
1985
Year
Validation Data
Combined Method
MAC
MAP
TFBC
TFBP

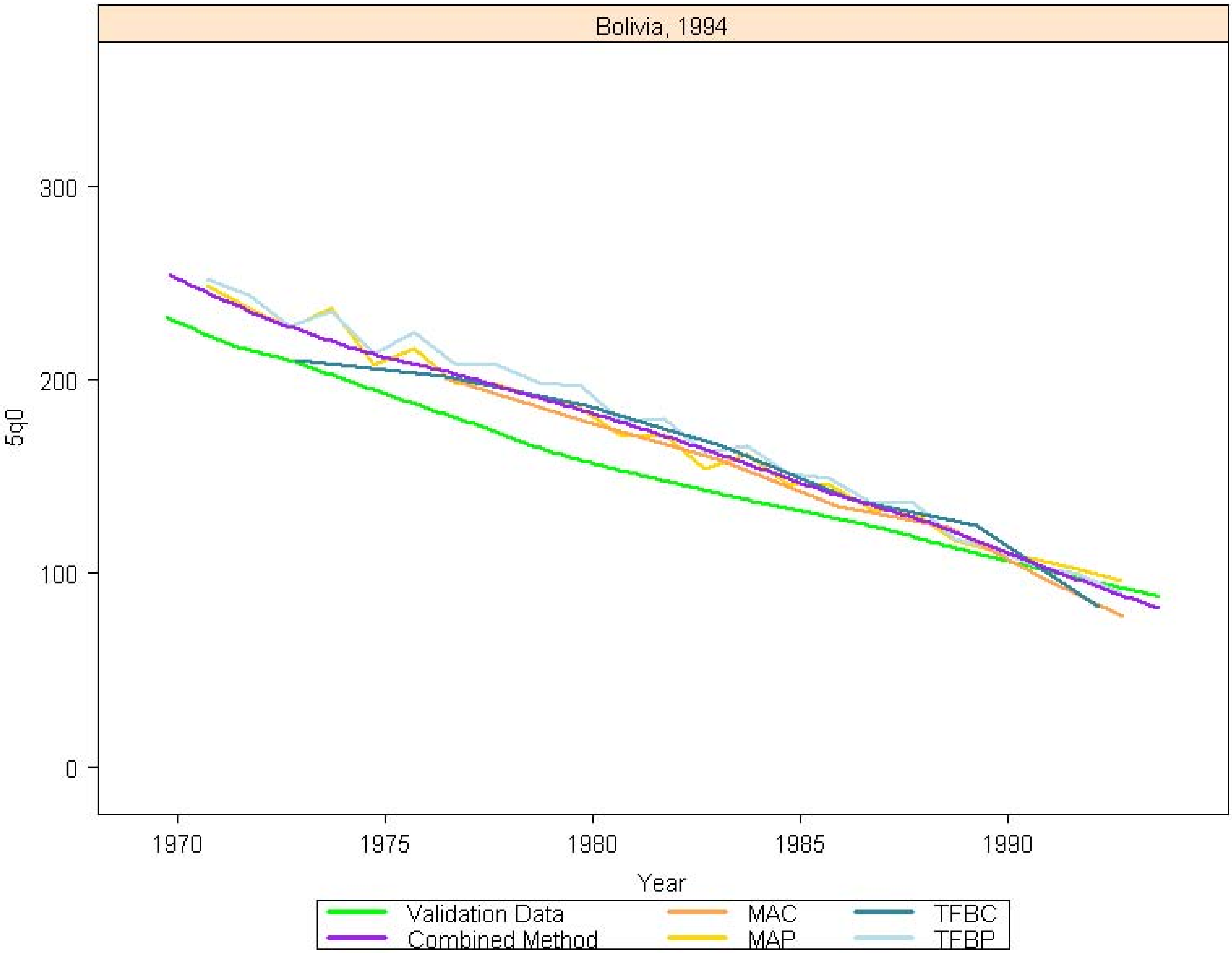
Bolivia, 1994
300
200
100
0
5q0
1970
1975
1980
1985
1990
Year
Validation Data
Combined Method
MAC
MAP
TFBC
TFBP

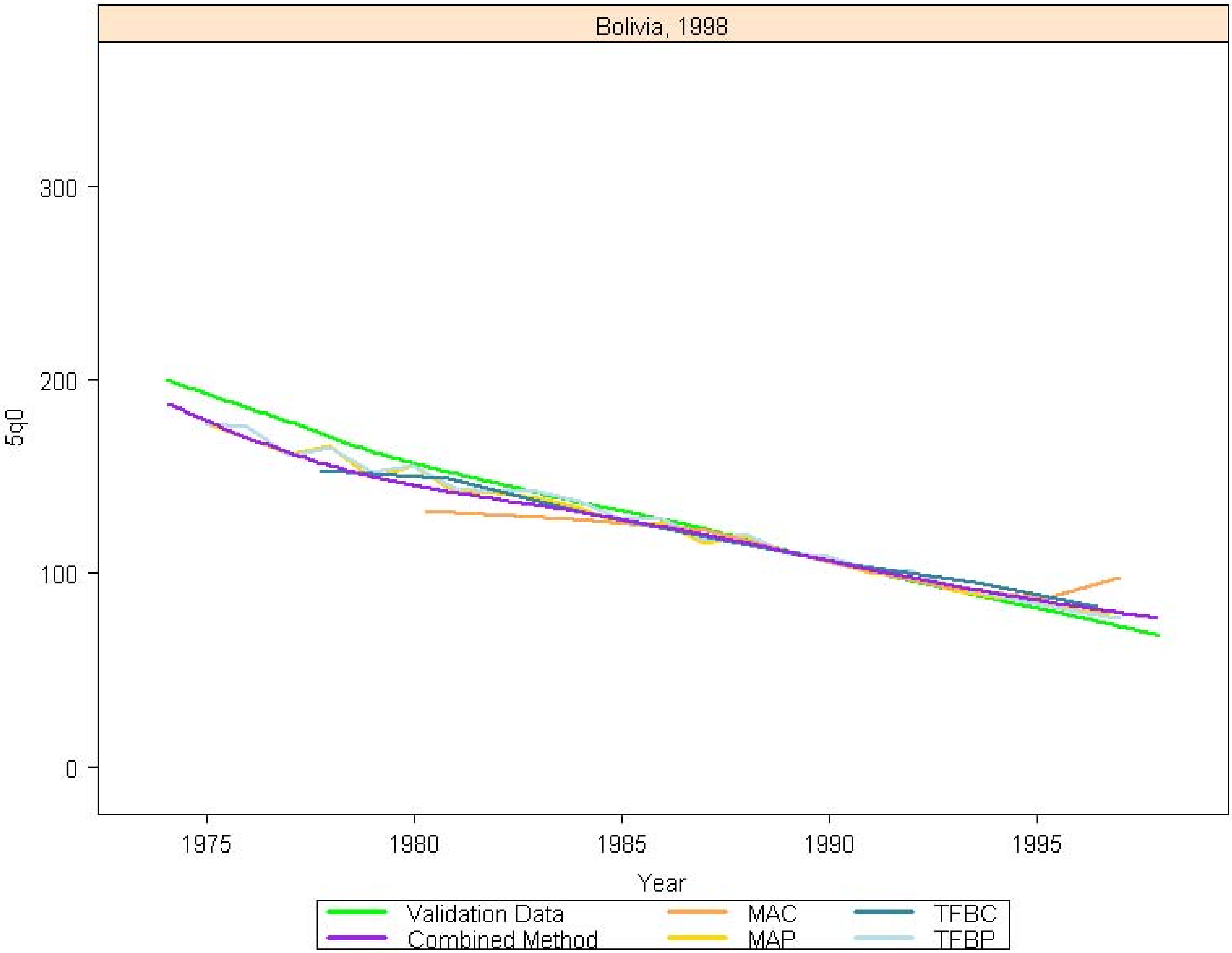

Bolivia, 1998
300
200
100
0
5q0
1975
1980
1985
1990
1995
Year
Validation Data
Combined Method
MAC
MAP
TFBC
TFBP

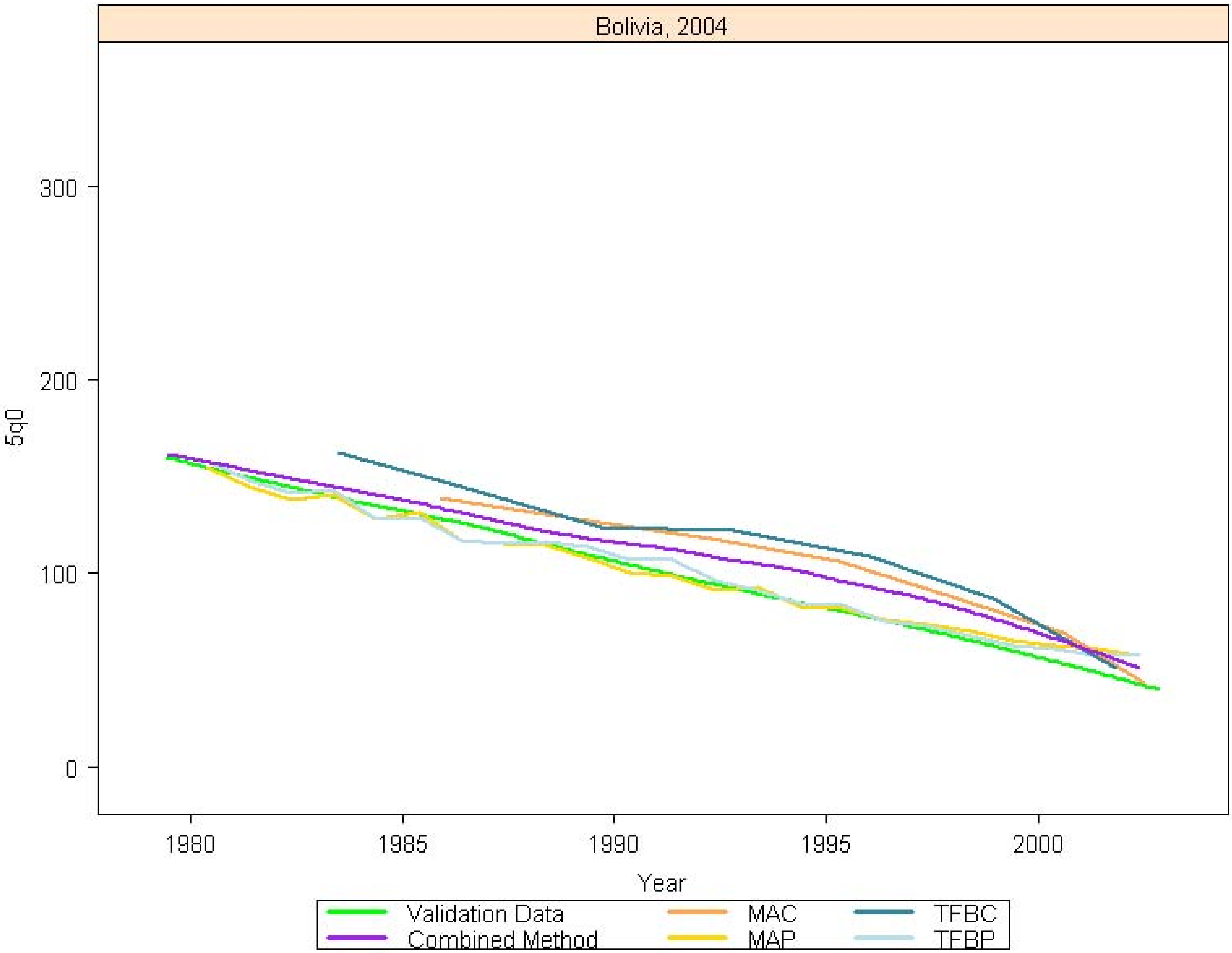
Bolivia, 2004
300
200
100
0
5q0
1980
1985
1990
1995
2000
Year
Validation Data
Combined Method
MAC
MAP
TFBC
TFBP

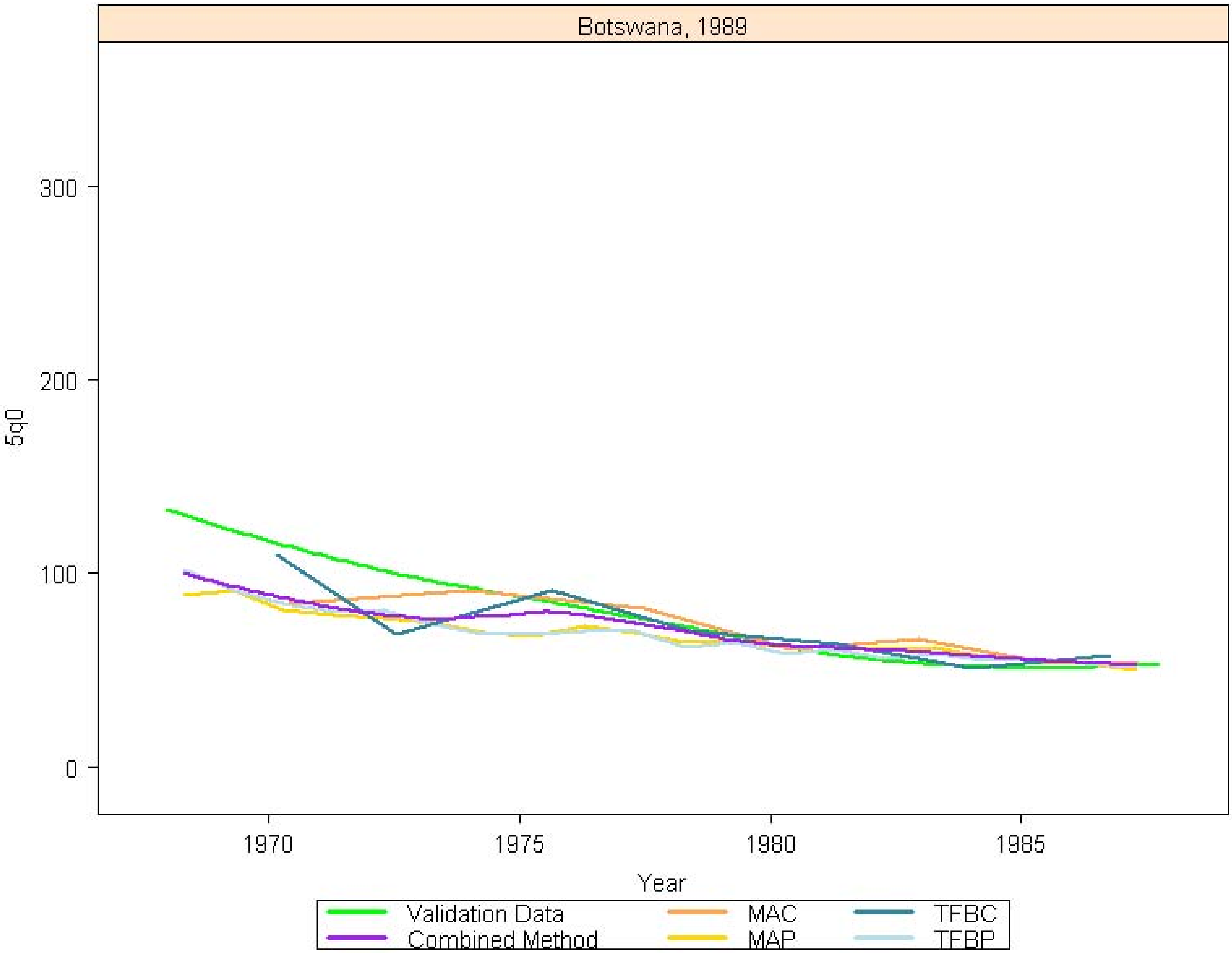
Botswana, 1989
5q0
300
200
100
0
1970
1975
1980
1985
Year
Validation Data
Combined Method
MAC
MAP
TFBC
TFBP

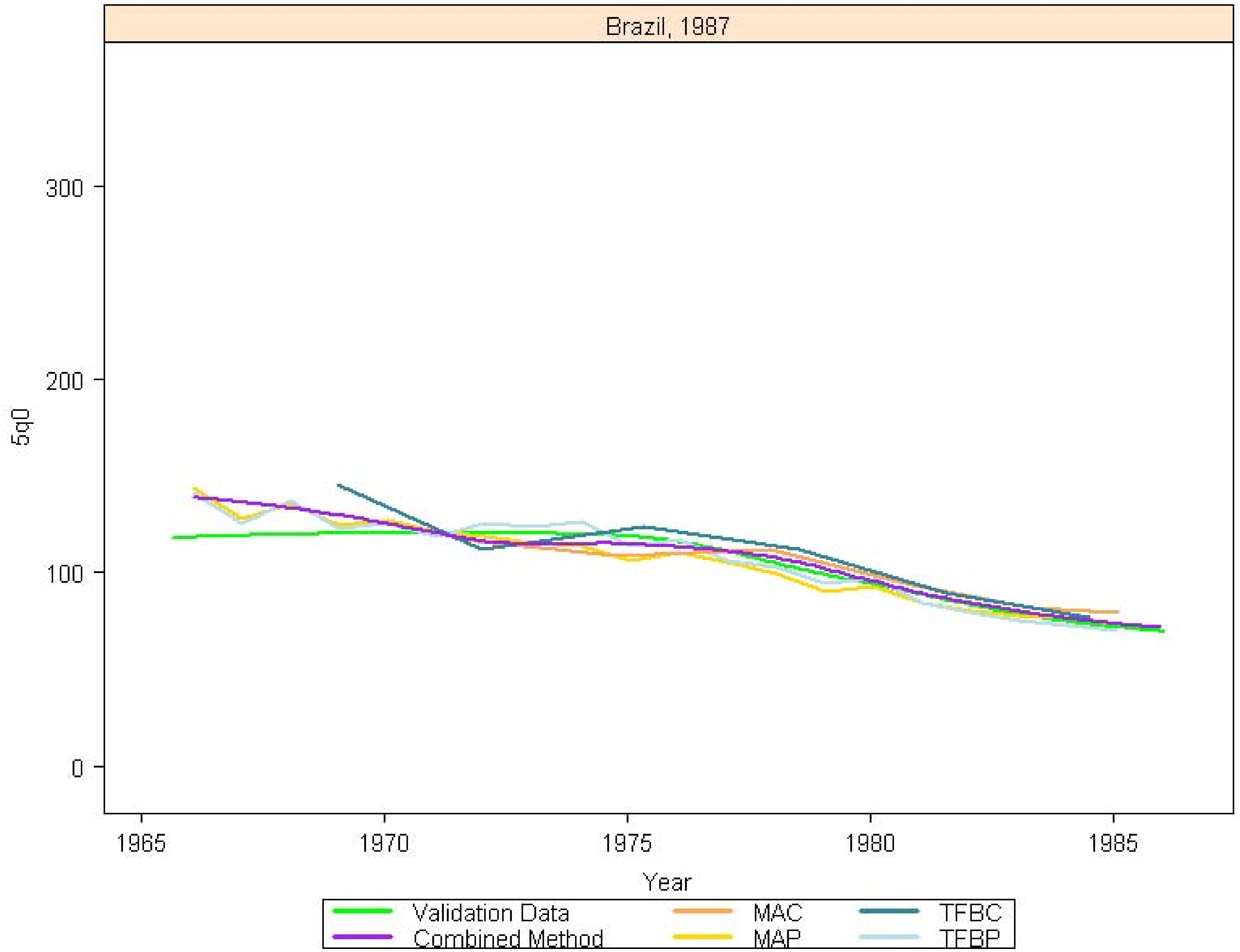

Brazil, 1987
5q0
300
200
100
0
1965
1970
1975
1980
1985
Year
Validation Data
Combined Method
MAC
MAP
TFBC
TFBP

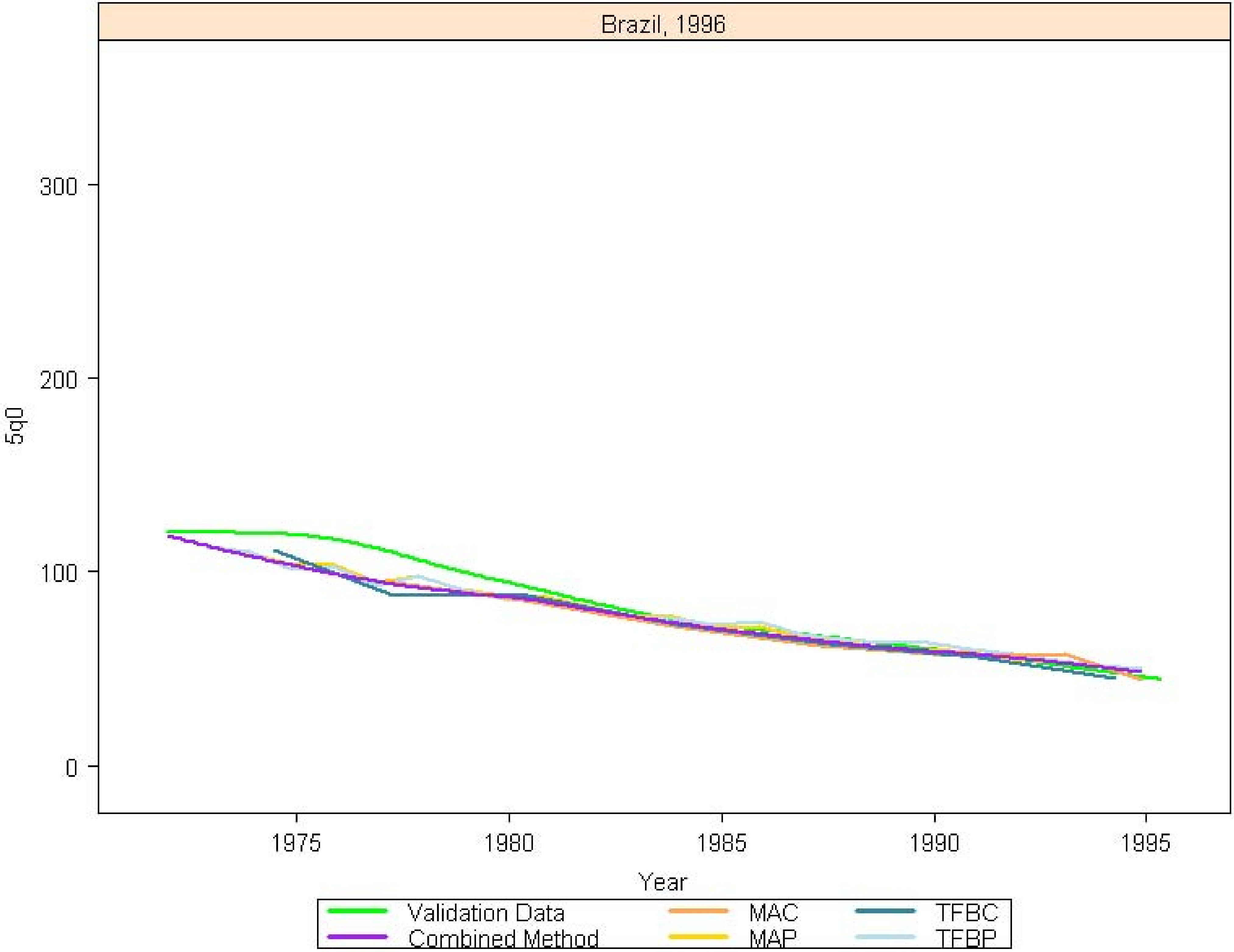
Brazil, 1996
300
200
100
0
5q0
1975
1980
1985
1990
1995
Year
Validation Data
Combined Method
MAC
MAP
TFBC
TFBP

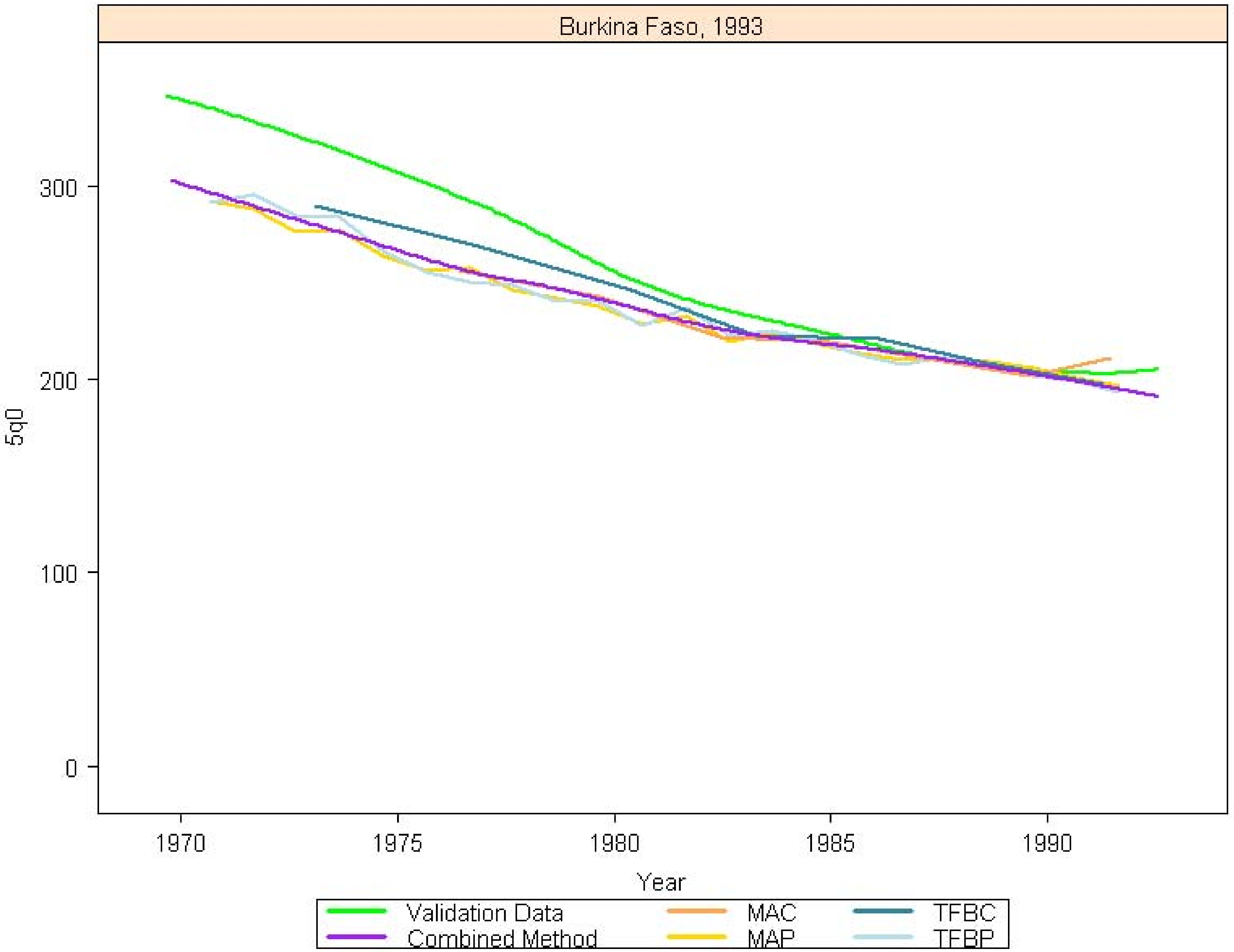

Burkina Faso, 1993
300
200
100
0
5q0
1970
1975
1980
1985
1990
Year
Validation Data
Combined Method
MAC
MAP
TFBC
TFBP

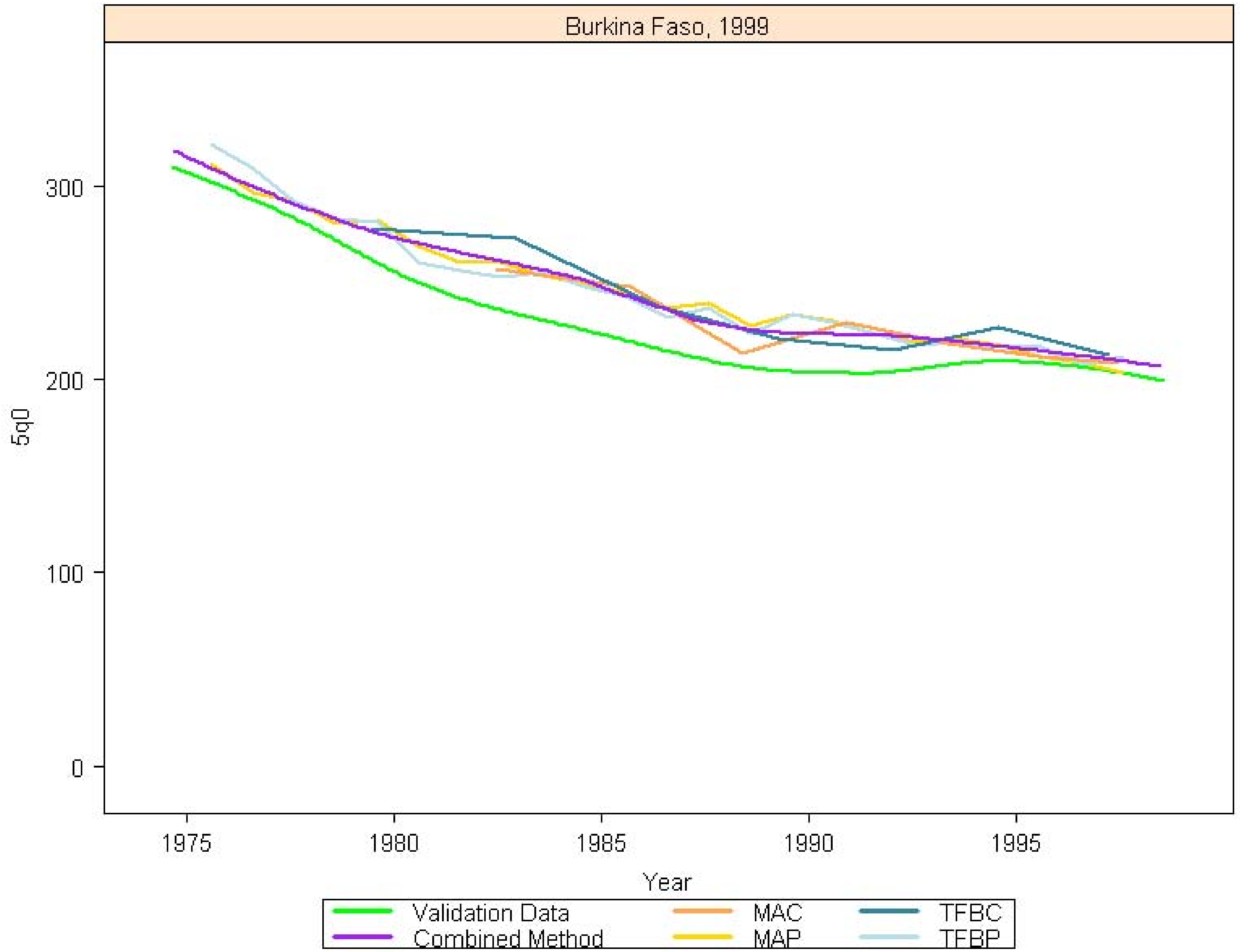
Burkina Faso, 1999
300
200
100
0
5q0
1975
1980
1985
1990
1995
Year
Validation Data
Combined Method
MAC
MAP
TFBC
TFBP

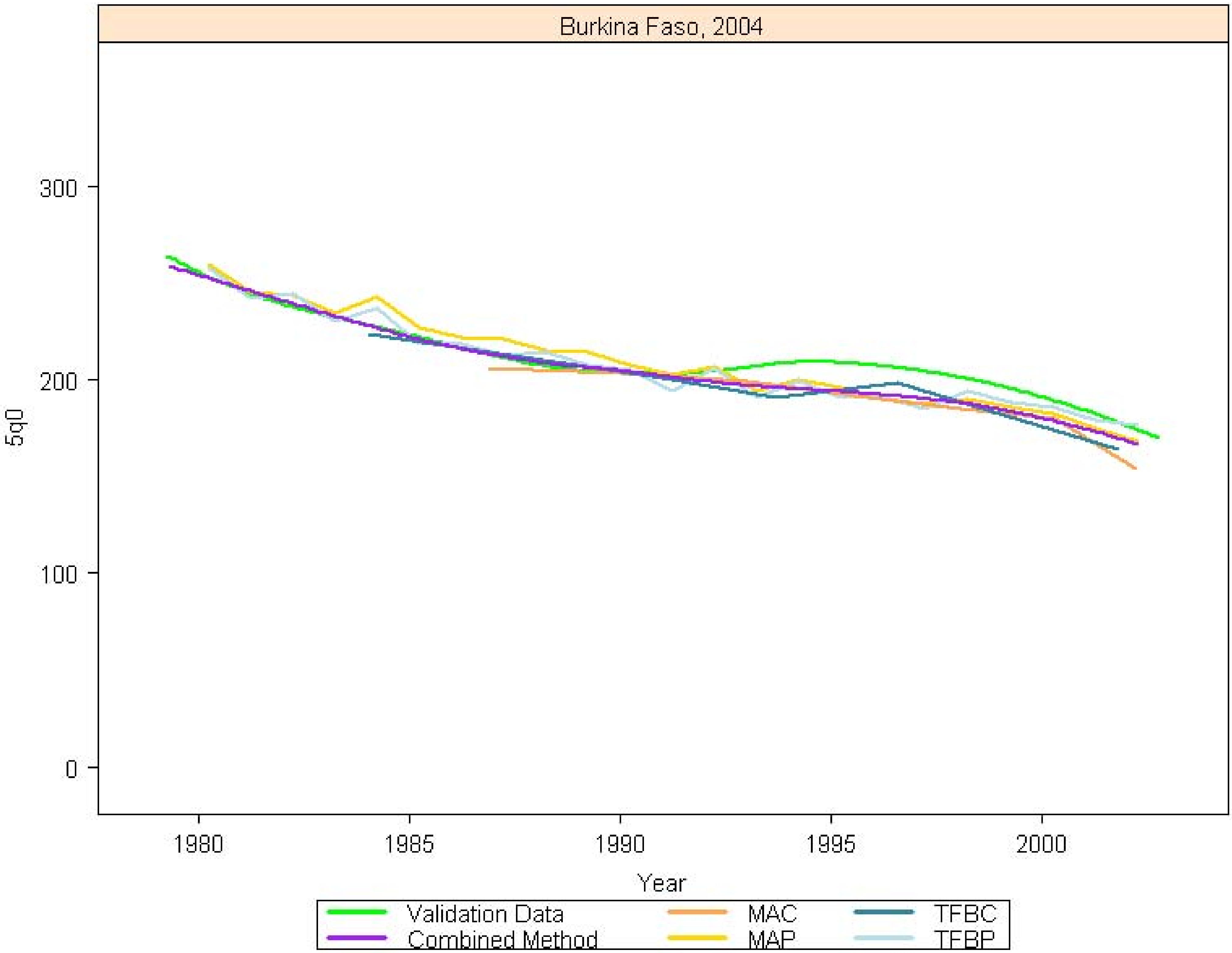
Burkina Faso, 2004
300
200
100
0
5q0
1980
1985
1990
1995
2000
Year
Validation Data
Combined Method
MAC
MAP
TFBC
TFBP

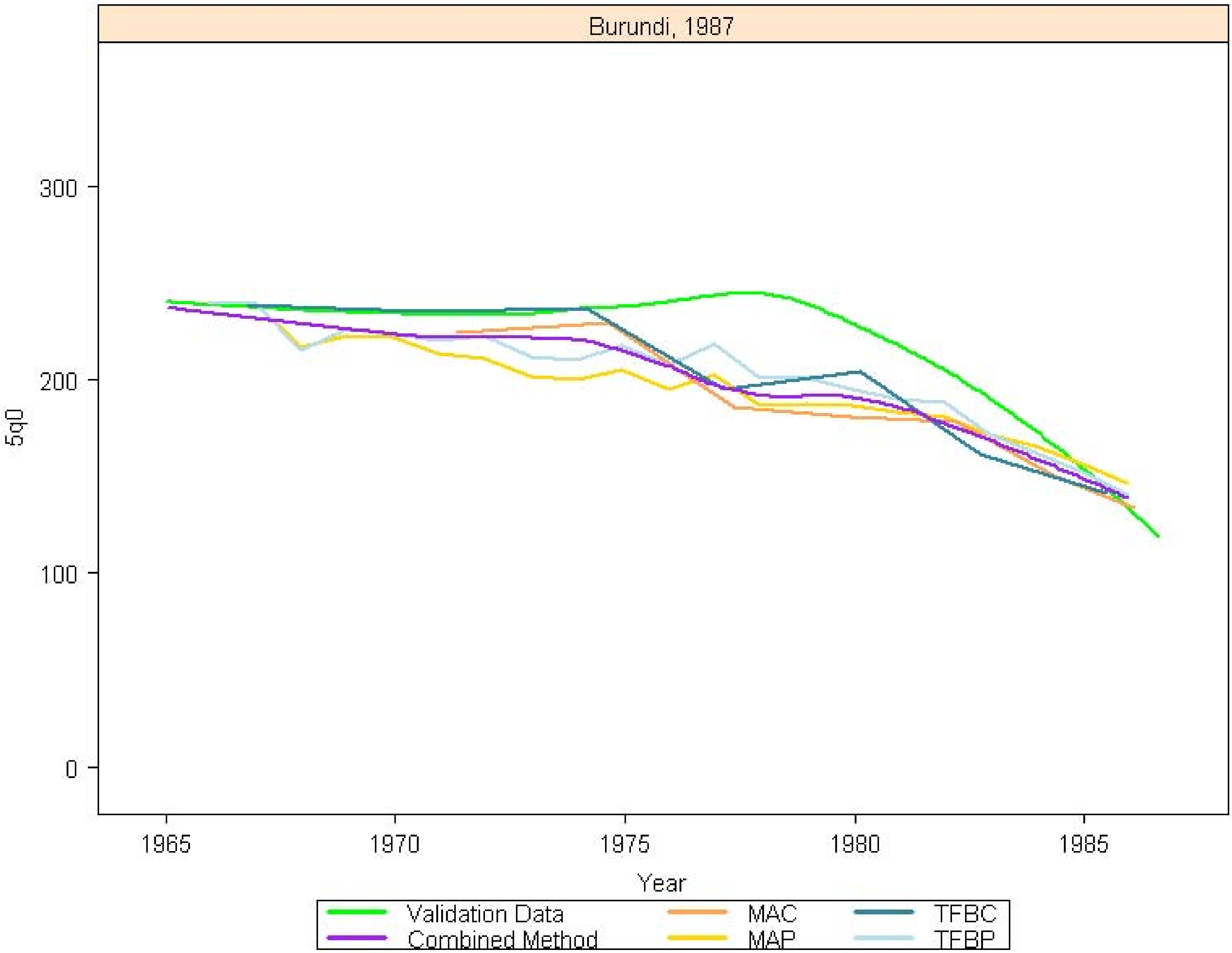

Burundi, 1987
300
200
100
0
5q0
1965
1970
1975
1980
1985
Year
Validation Data
Combined Method
MAC
MAP
TFBC
TFBP

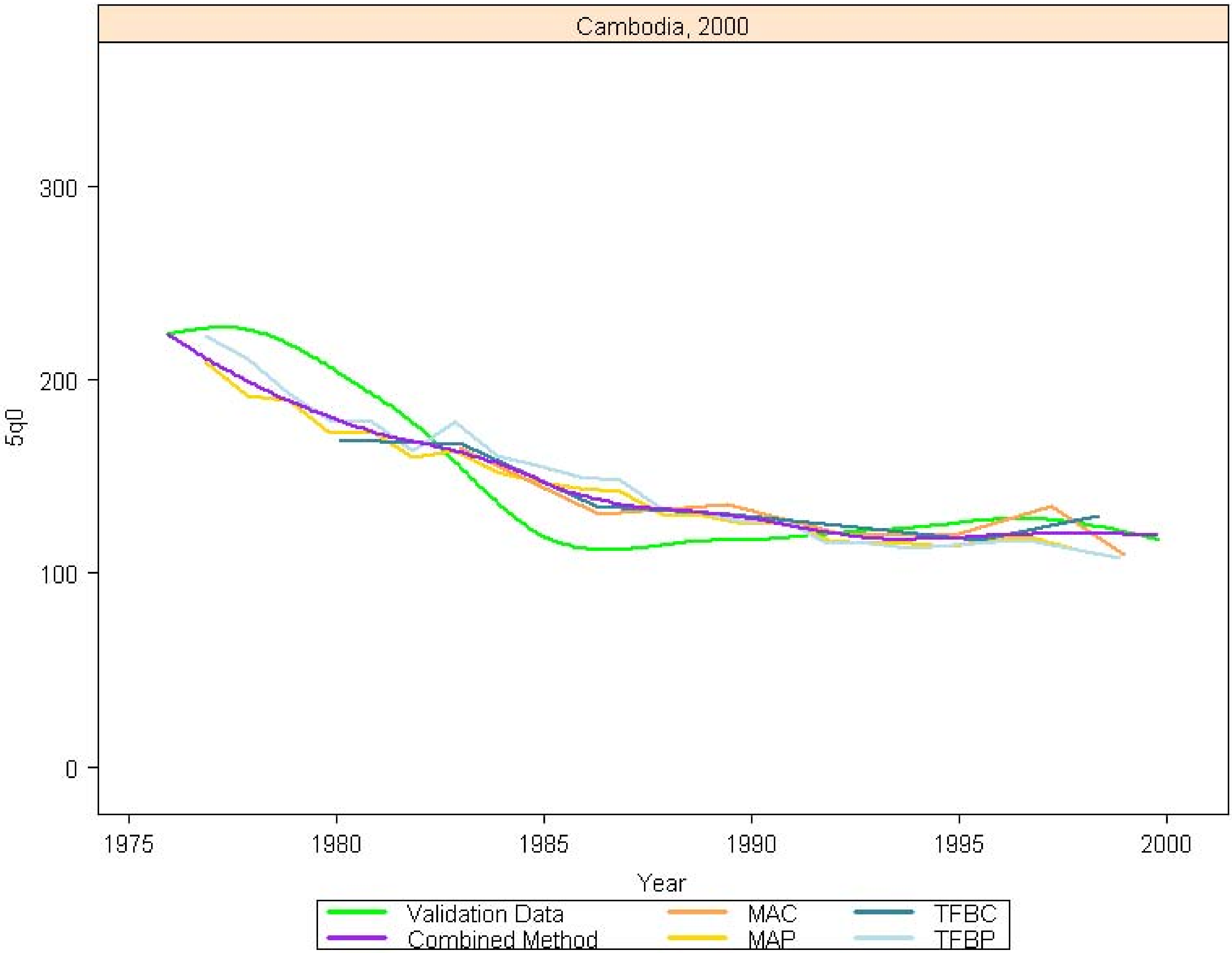
Cambodia, 2000
5q0
300
200
100
0
1975
1980
1985
1990
1995
2000
Year
Validation Data
Combined Method
MAC
MAP
TFBC
TFBP

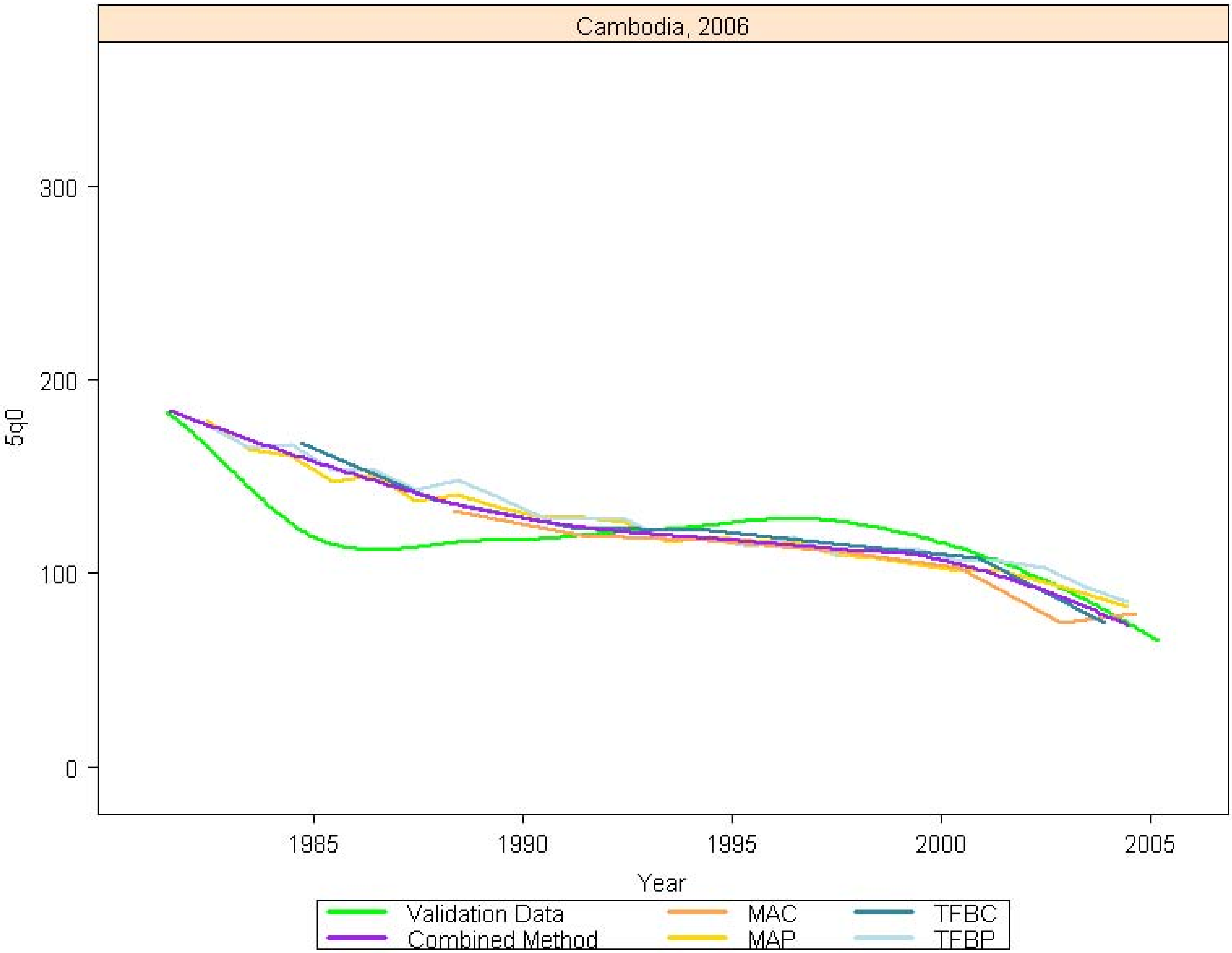

Cambodia, 2006
300
200
100
0
5q0
1985
1990
1995
2000
2005
Year
Validation Data
Combined Method
MAC
MAP
TFBC
TFBP

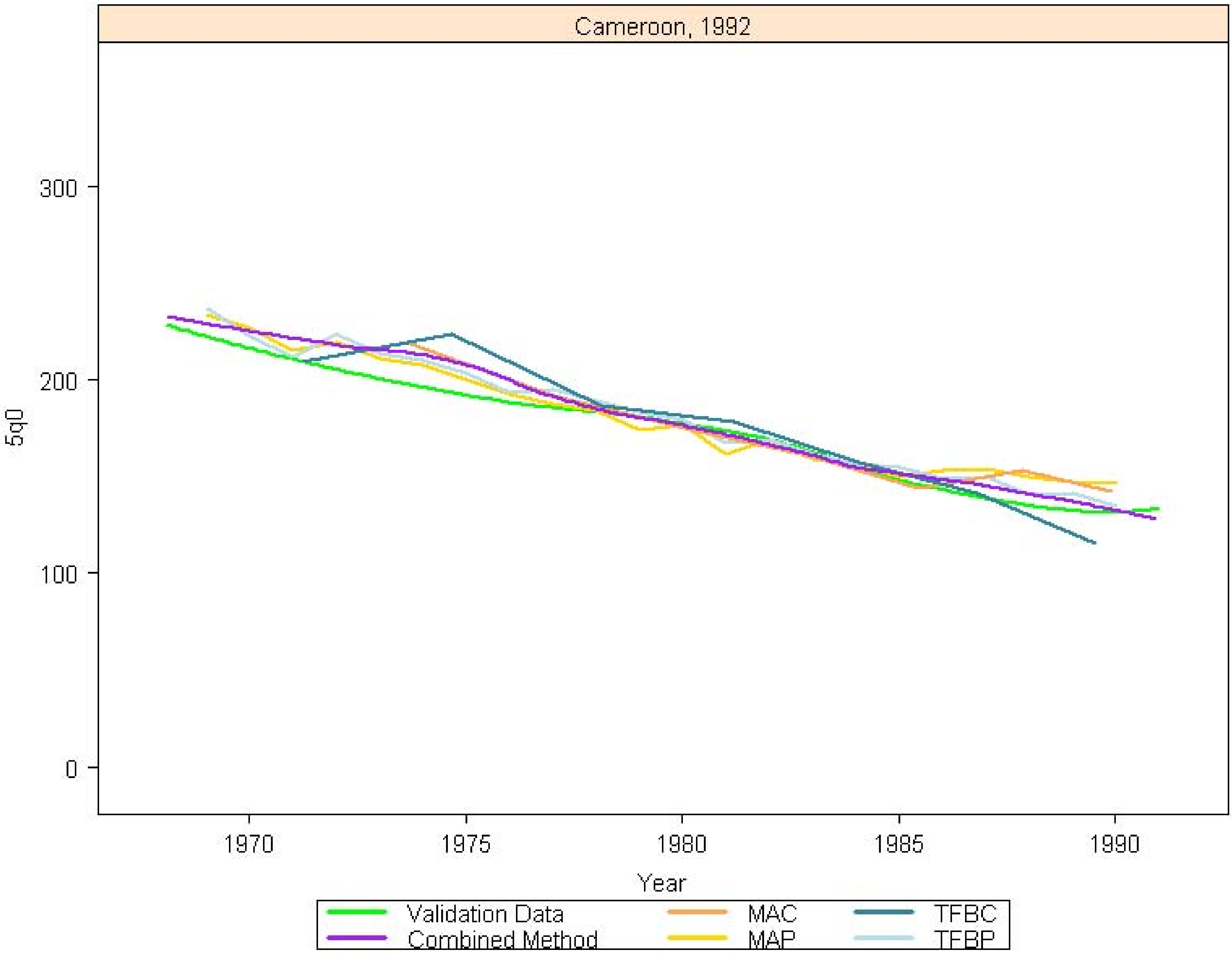
Cameroon, 1992
300
200
100
0
5q0
1970
1975
1980
1985
1990
Year
Validation Data
Combined Method
MAC
MAP
TFBC
TFBP

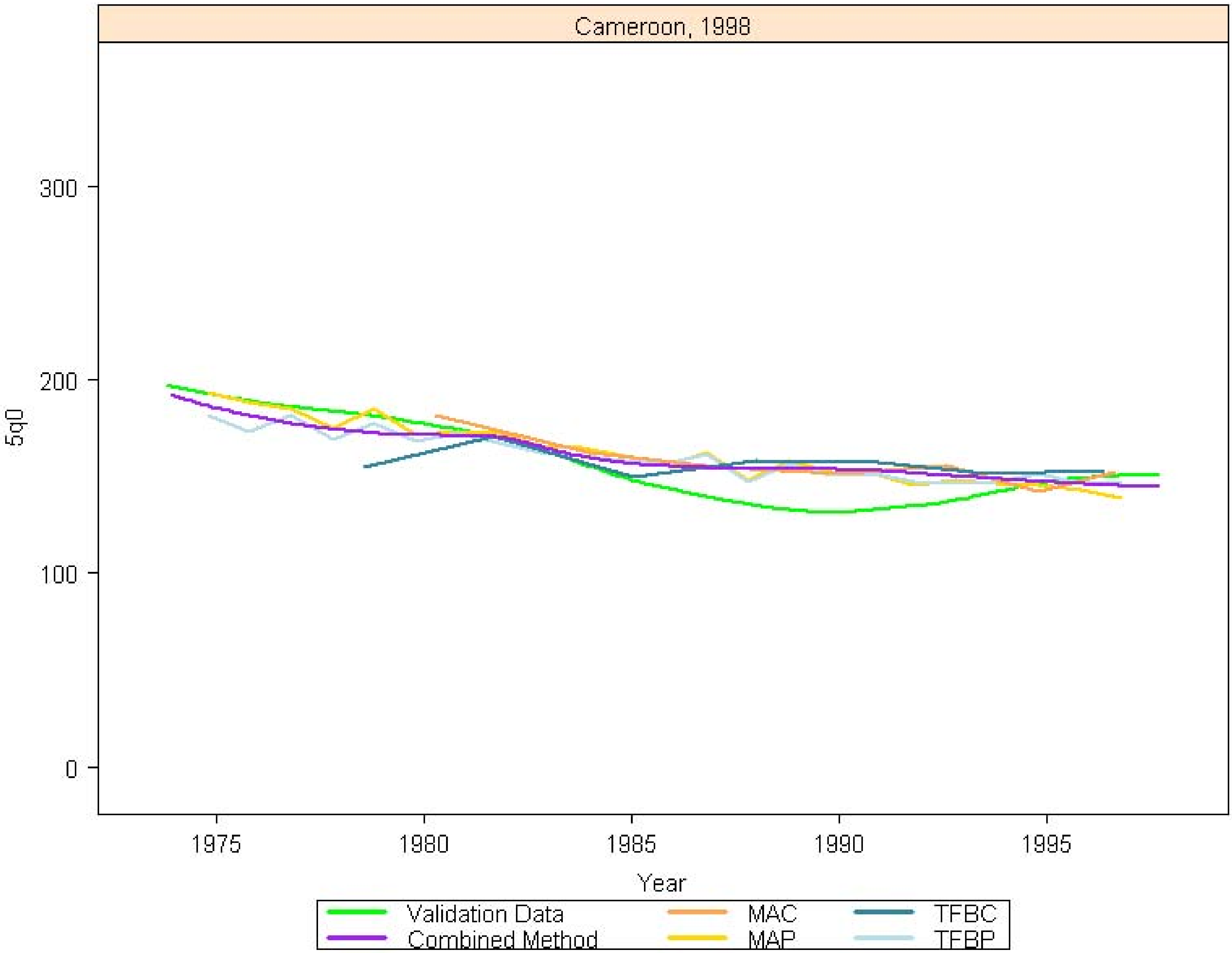
Cameroon, 1998
300
200
100
0
5q0
1975
1980
1985
1990
1995
Year
Validation Data
Combined Method
MAC
MAP
TFBC
TFBP

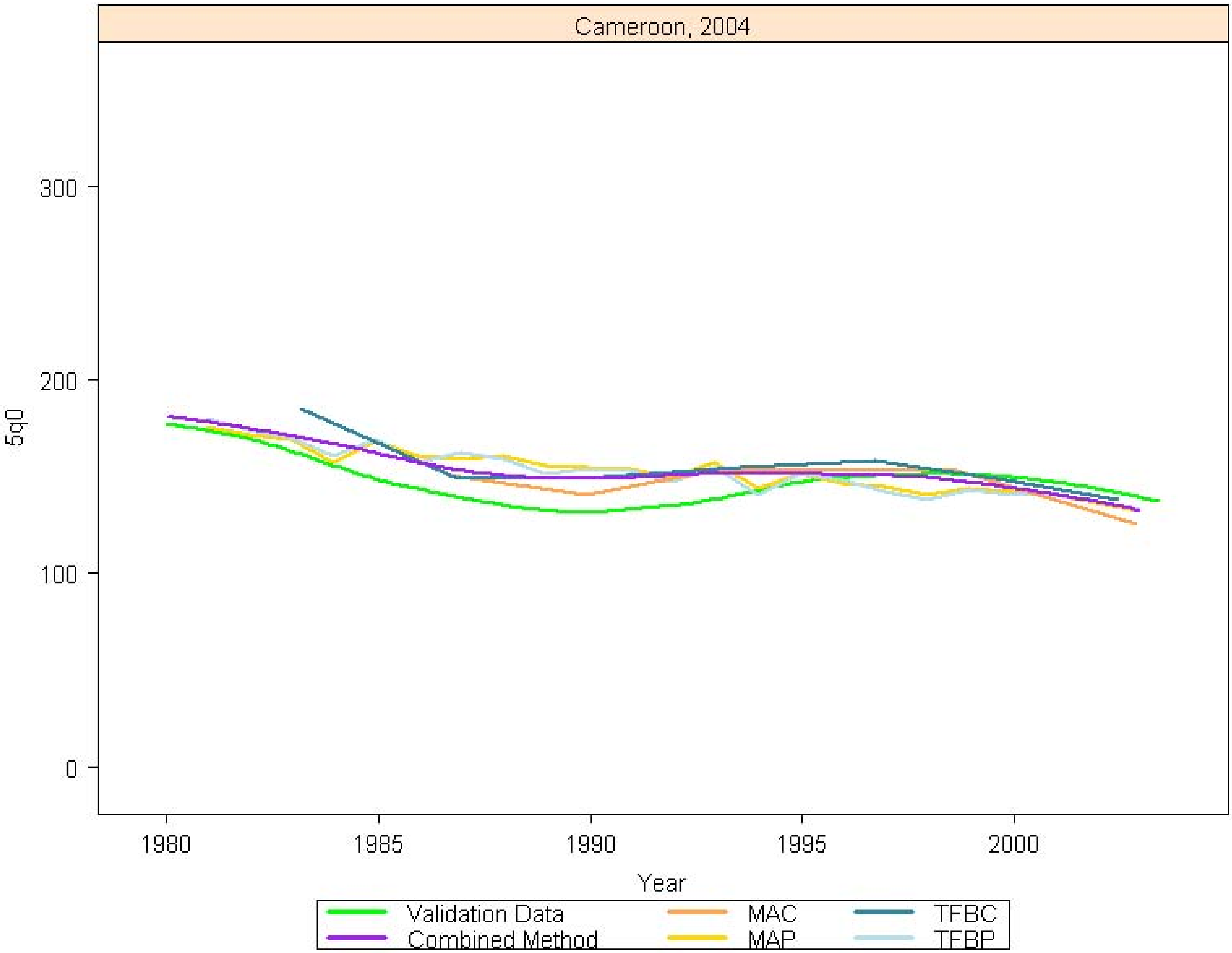
Cameroon, 2004
300
200
100
0
5q0
1980
1985
1990
1995
2000
Year
Validation Data
Combined Method
MAC
MAP
TFBC
TFBP

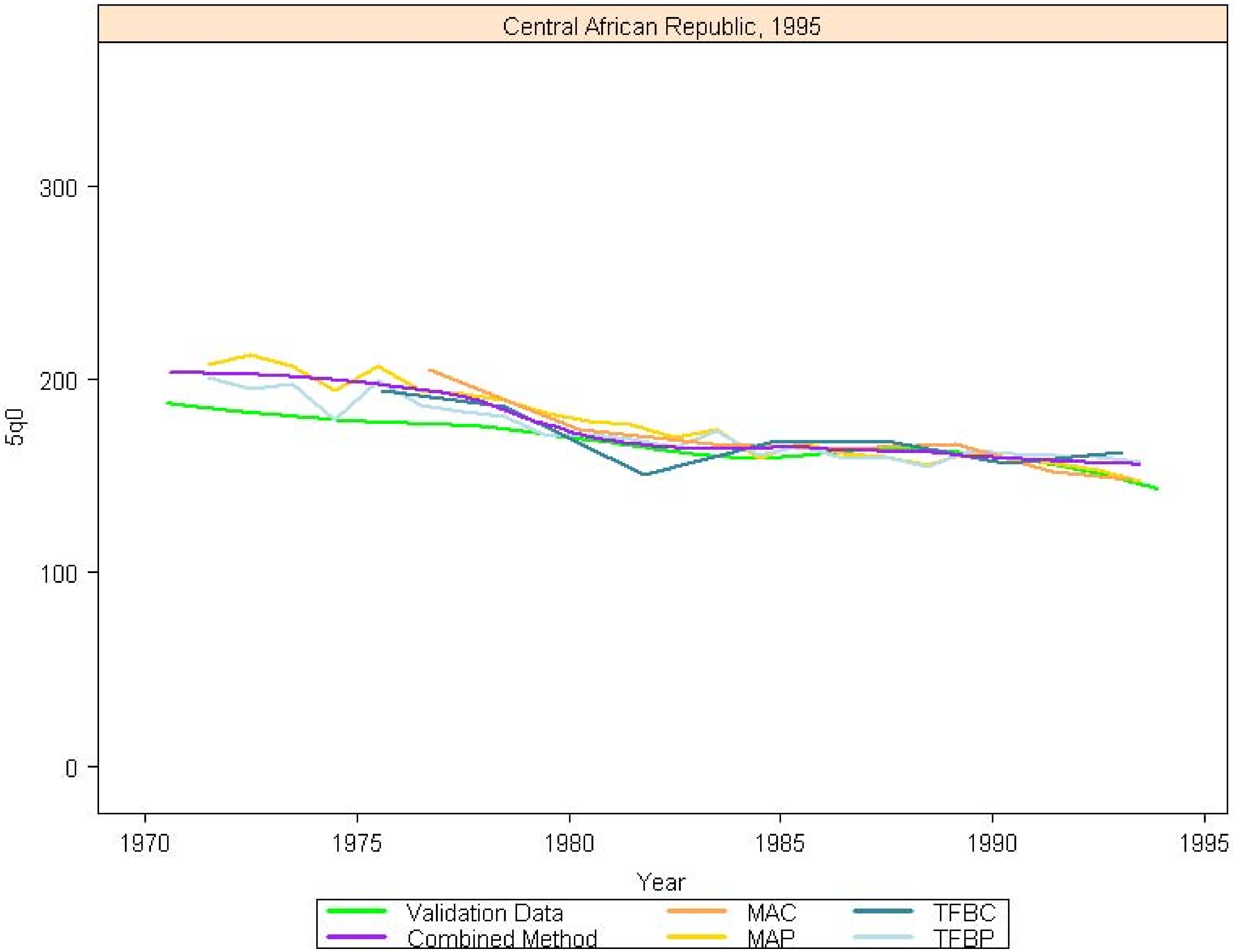
Central African Republic, 1995
300
200
100
0
5q0
1970
1975
1980
1985
1990
1995
Year
Validation Data
Combined Method
MAC
MAP
TFBC
TFBP

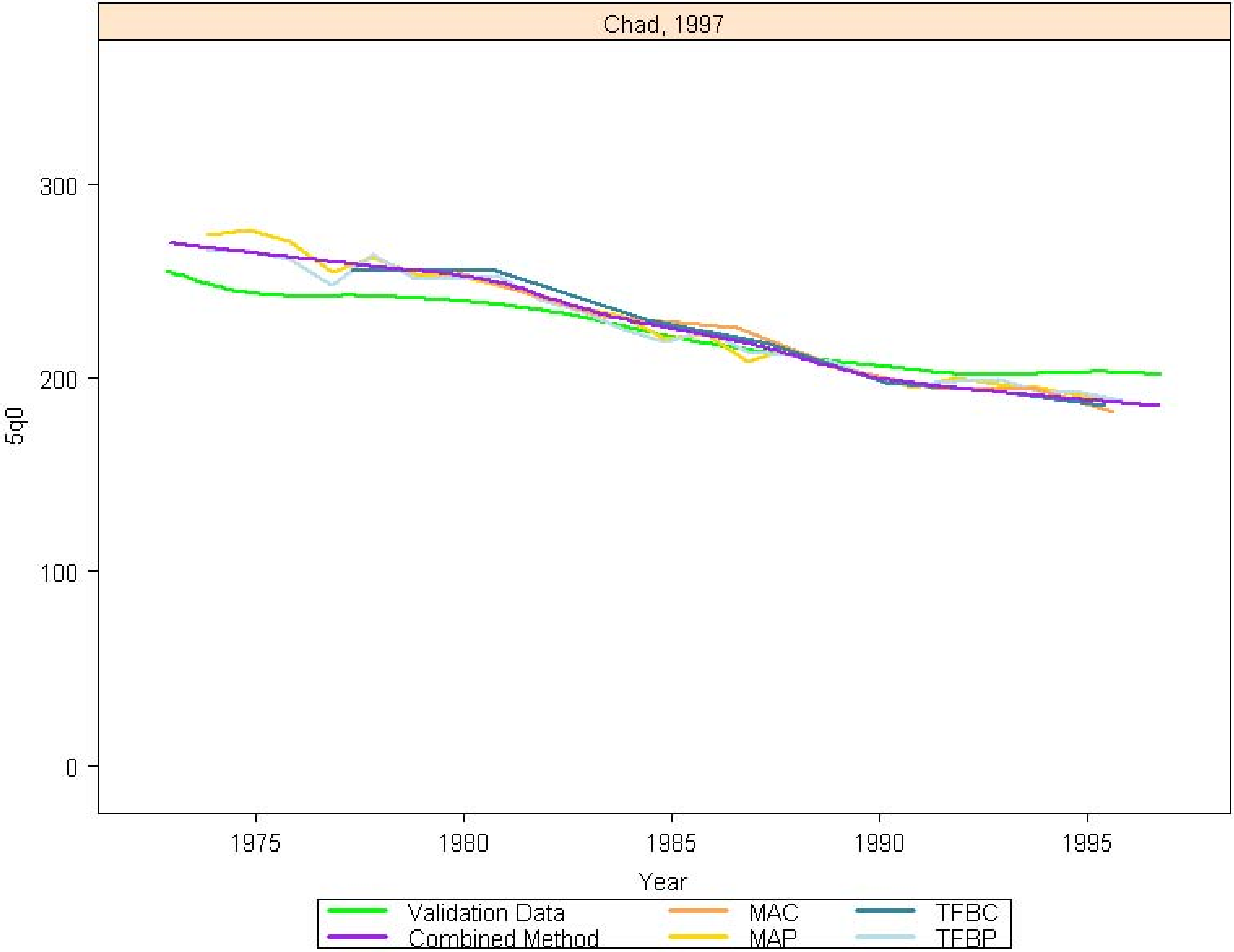

Chad, 1997
300
200
100
0
5q0
1975
1980
1985
1990
1995
Year
Validation Data
Combined Method
MAC
MAP
TFBC
TFBP

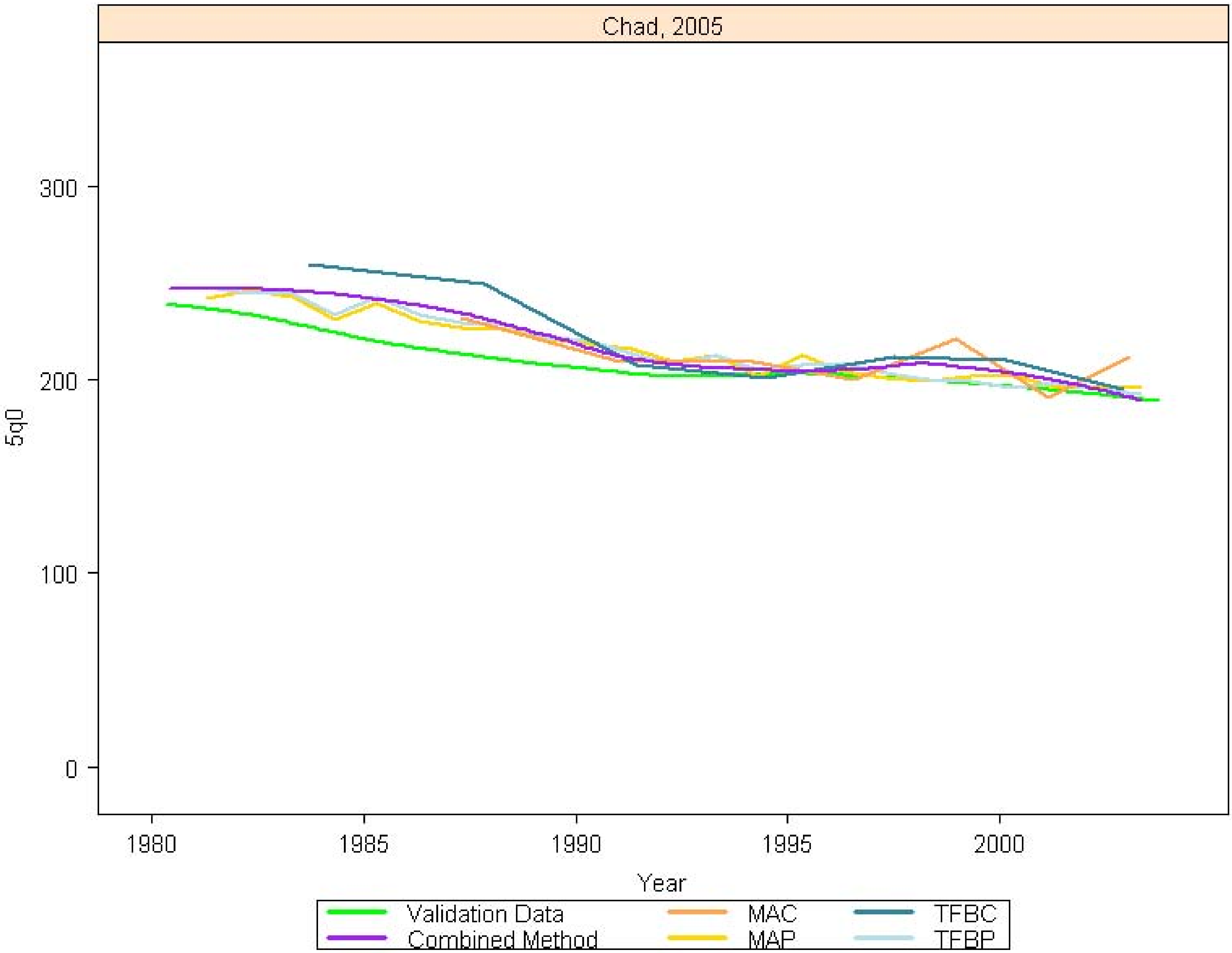

Chad, 2005
300
200
100
0
5q0
1980
1985
1990
1995
2000
Year
Validation Data
Combined Method
MAC
MAP
TFBC
TFBP

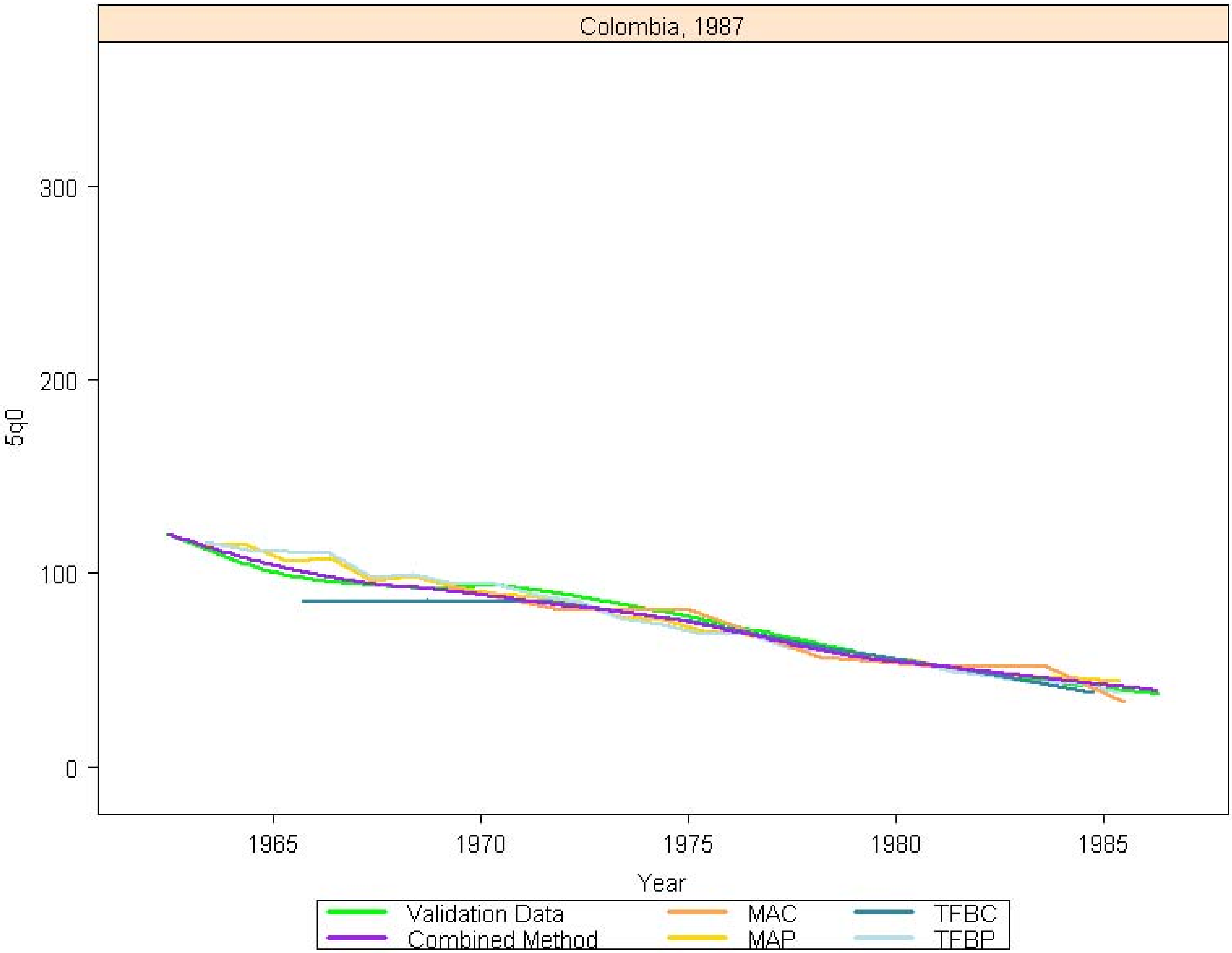
Colombia, 1987
300
200
100
0
5q0
1965
1970
1975
1980
1985
Year
Validation Data
Combined Method
MAC
MAP
TFBC
TFBP

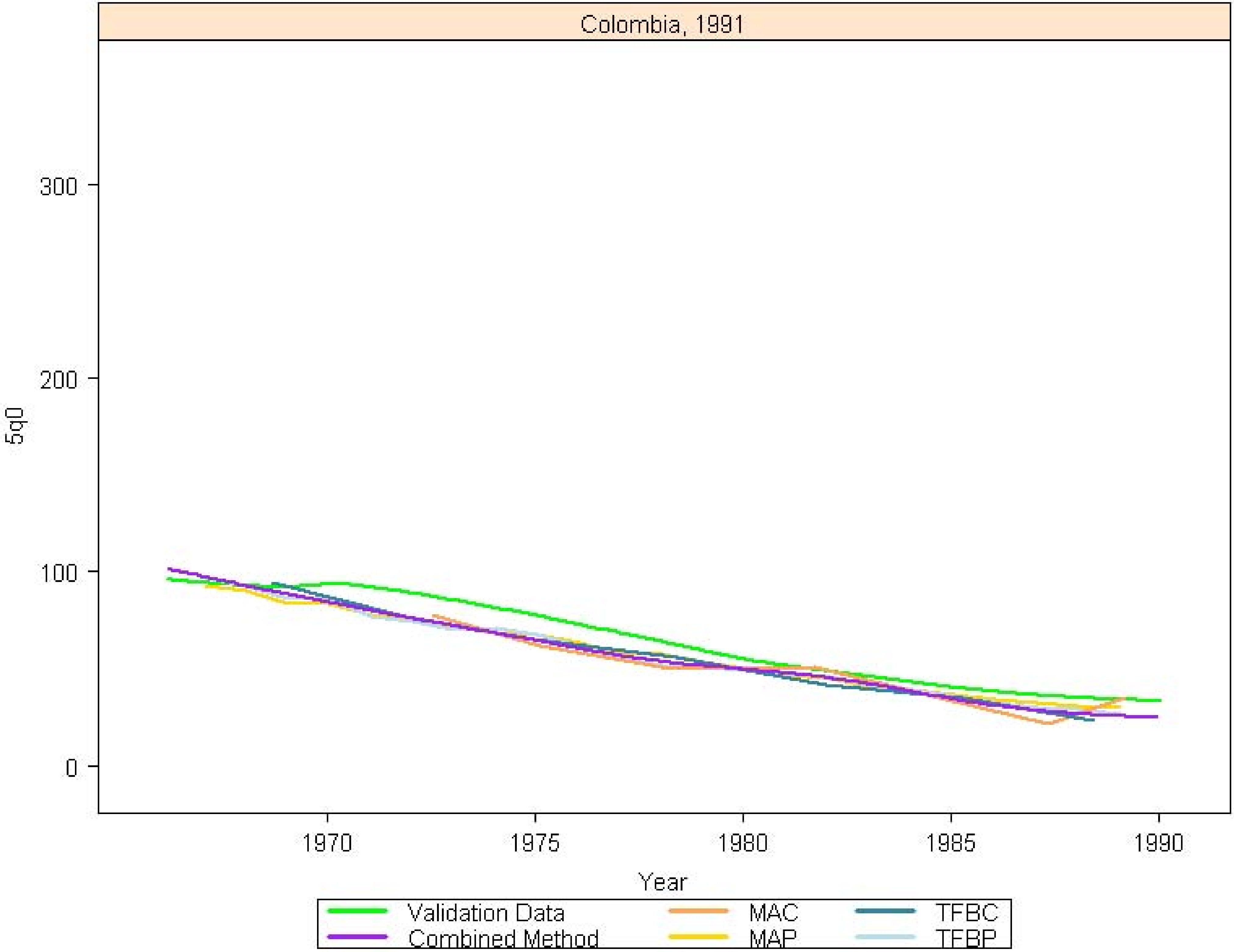
Colombia, 1991
5q0
300
200
100
0
1970
1975
1980
1985
1990
Year
Validation Data
Combined Method
MAC
MAP
TFBC
TFBP

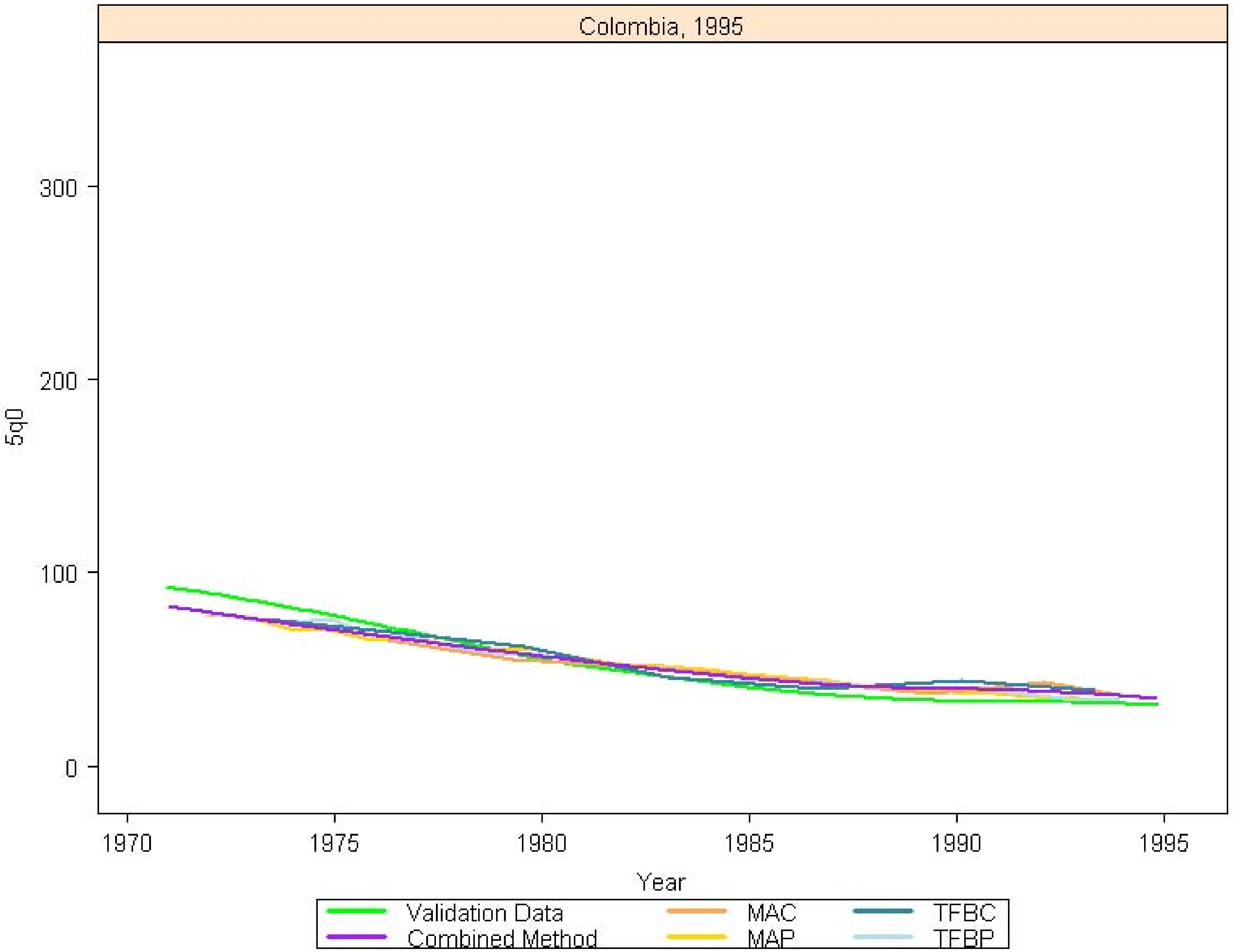

Colombia, 1995
300
200
100
0
5q0
1970
1975
1980
1985
1990
1995
Year
Validation Data
Combined Method
MAC
MAP
TFBC
TFBP

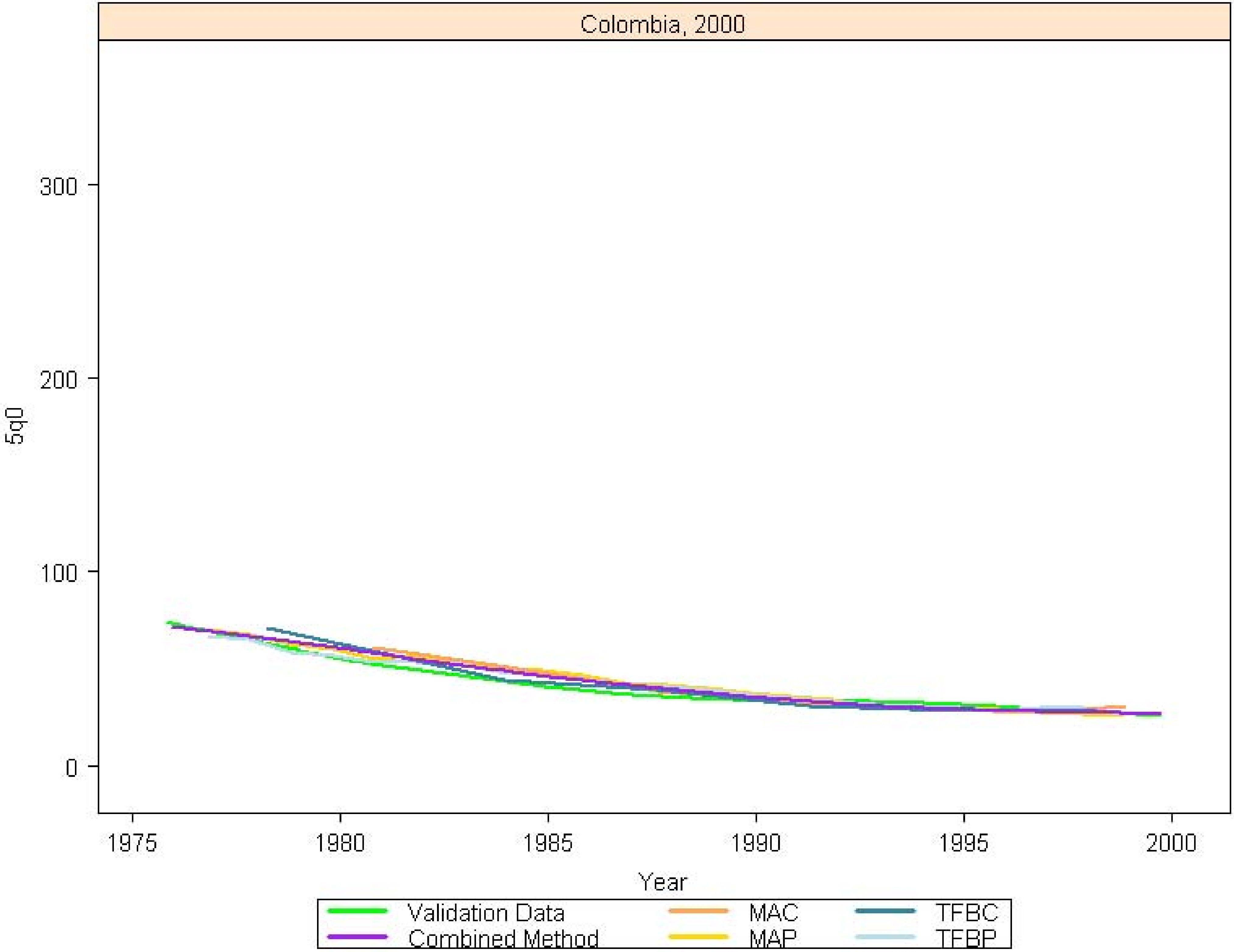
Colombia, 2000
300
200
100
0
5q0
1975
1980
1985
1990
1995
2000
Year
Validation Data
Combined Method
MAC
MAP
TFBC
TFBP

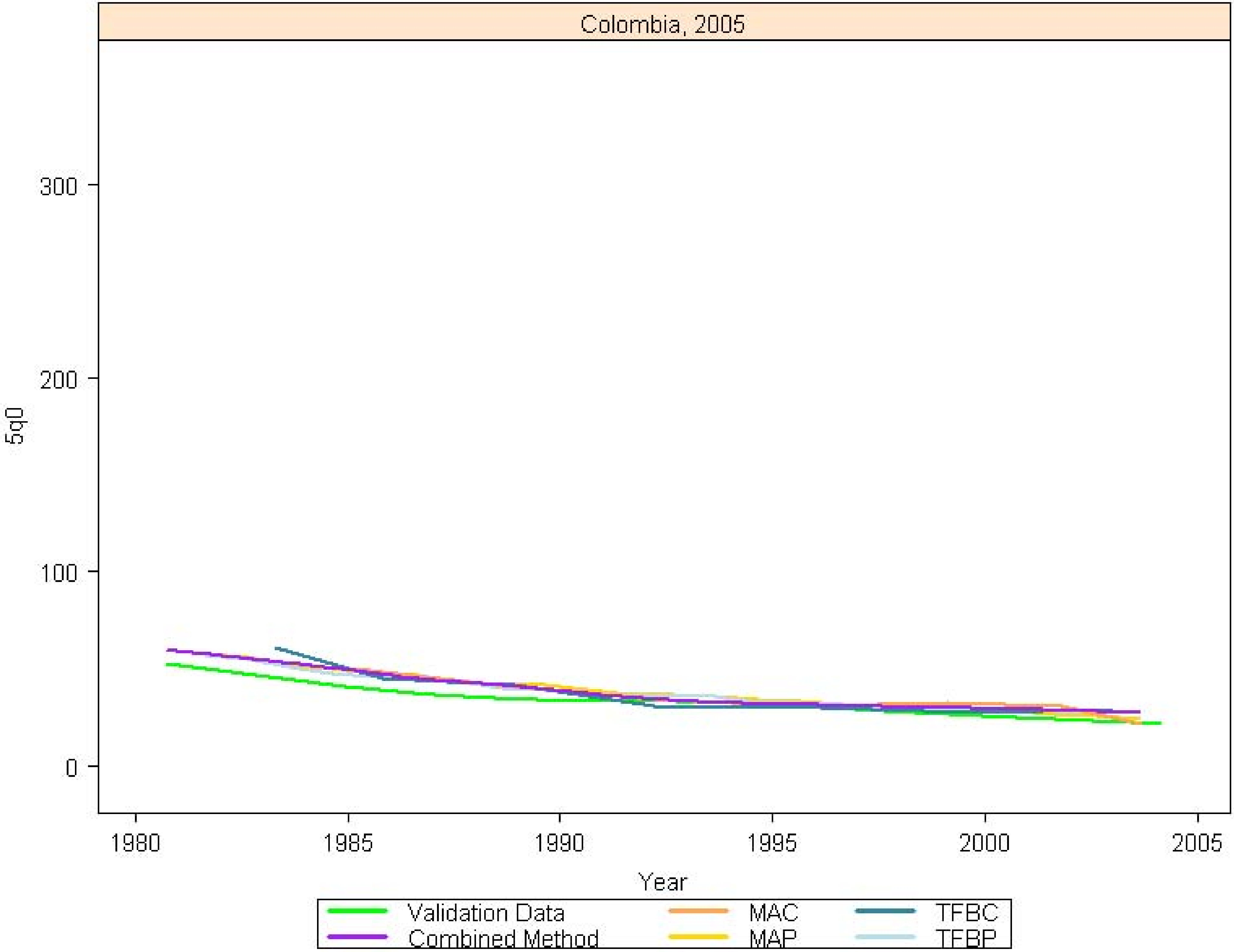
Colombia, 2005
5q0
300
200
100
0
1980
1985
1990
1995
2000
2005
Year
Validation Data
Combined Method
MAC
MAP
TFBC
TFBP

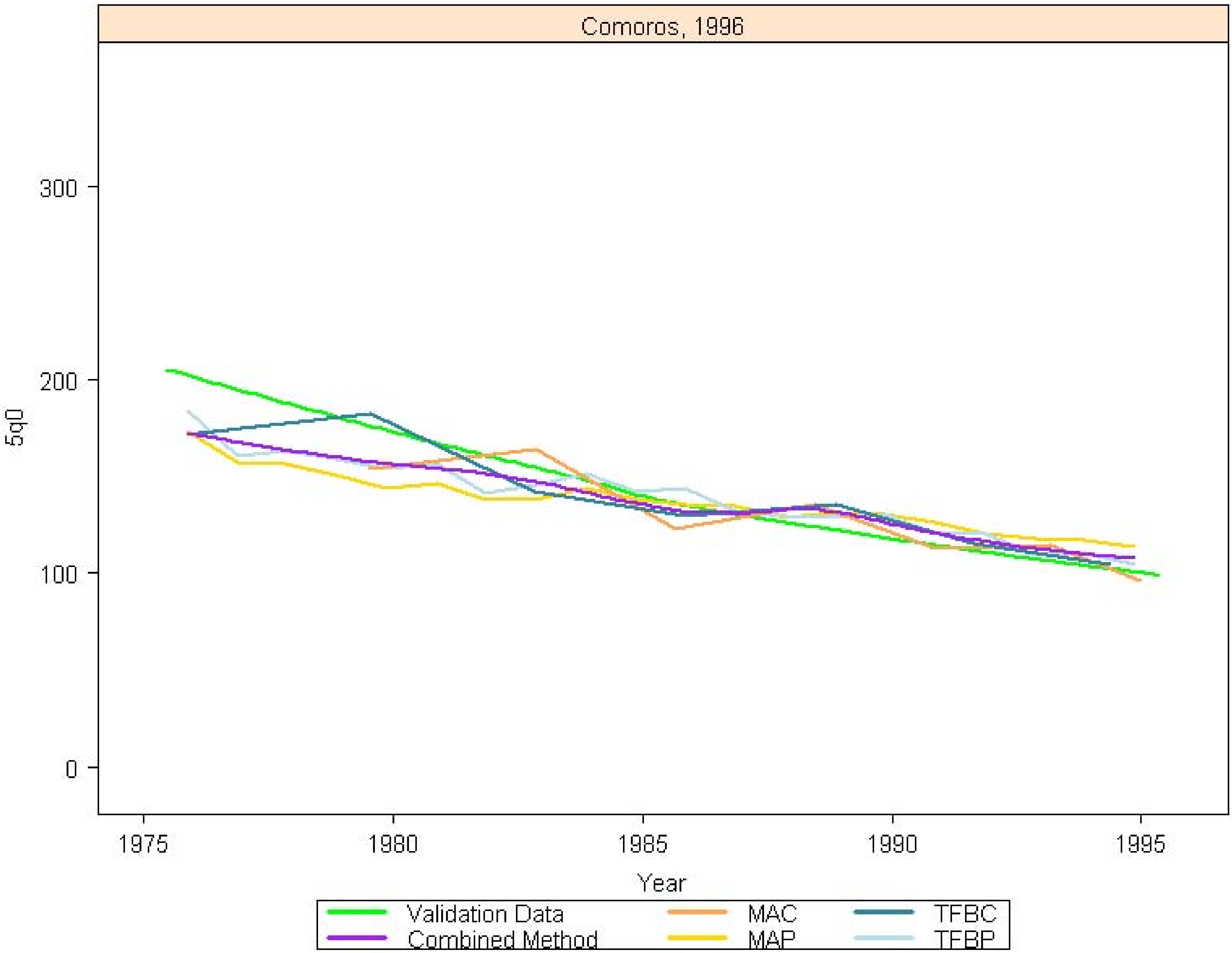

Comoros, 1996
5q0
0
100
200
300
1975
1980
1985
1990
1995
Year
Validation Data
Combined Method
MAC
MAP
TFBC
TFBP

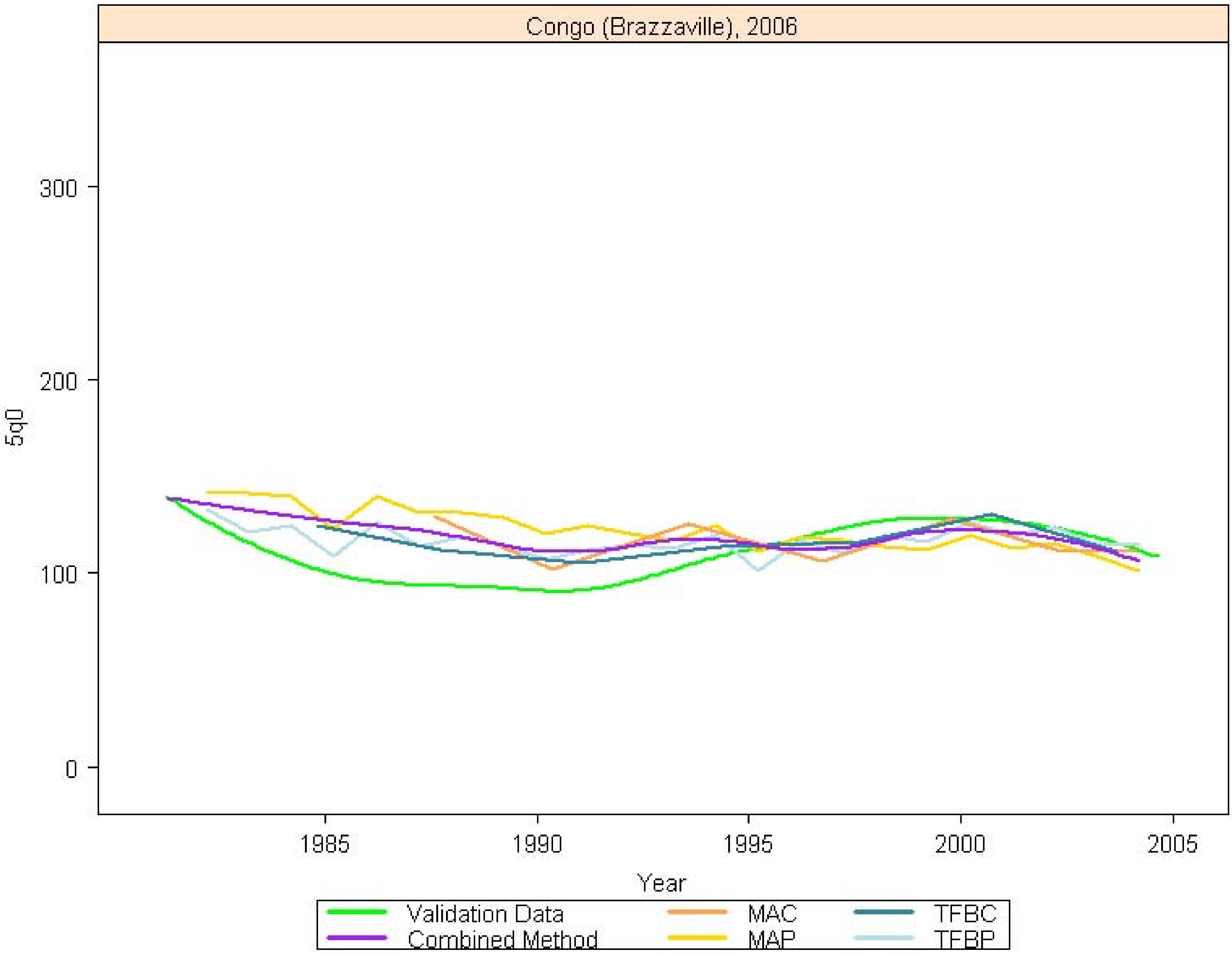
Congo (Brazzaville), 2006
5q0
300
200
100
0
1985
1990
1995
2000
2005
Year
Validation Data
Combined Method
MAC
MAP
TFBC
TFBP

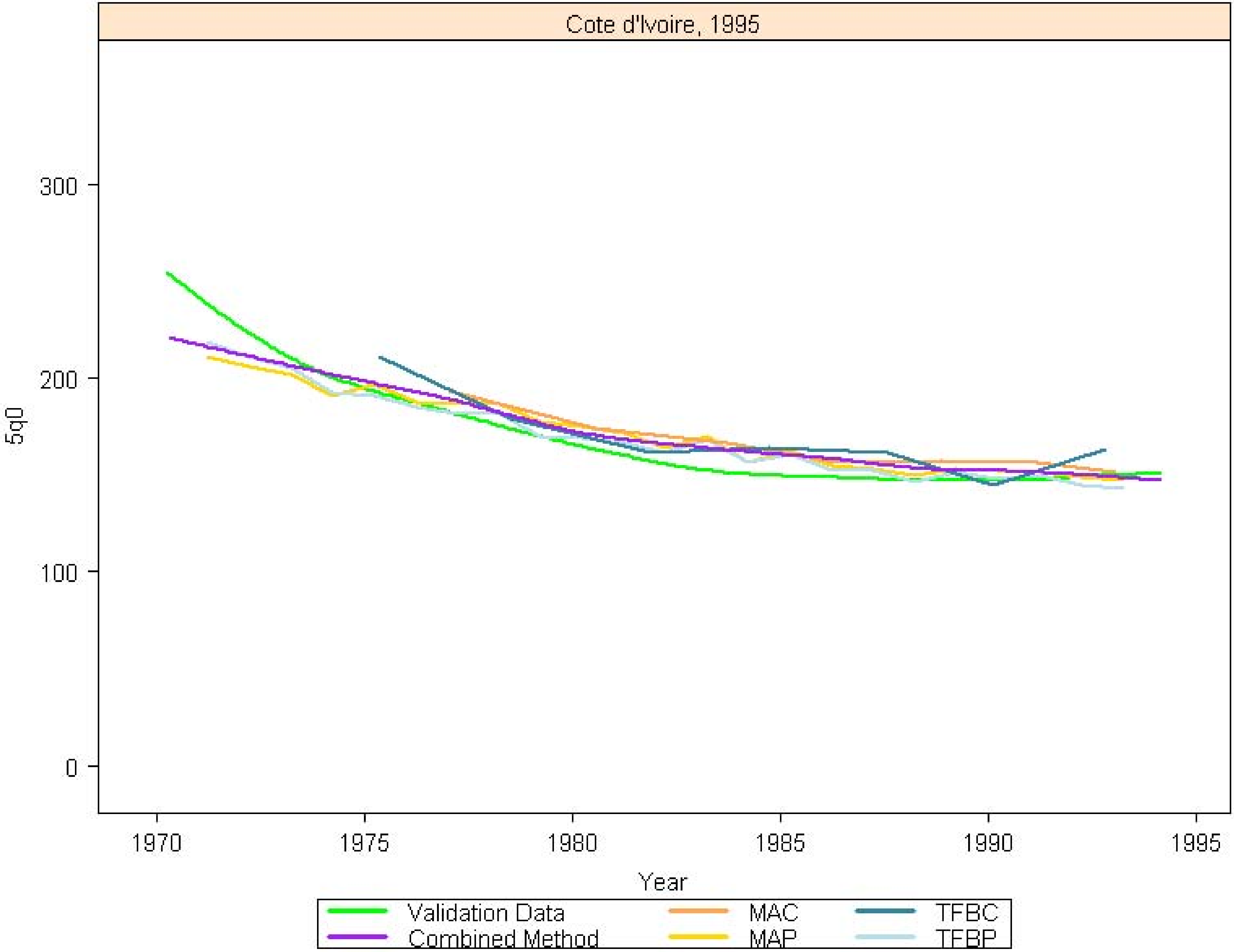
Cote d'Ivoire, 1995
300
200
100
0
5q0
1970
1975
1980
1985
1990
1995
Year
Validation Data
Combined Method
MAC
MAP
TFBC
TFBP

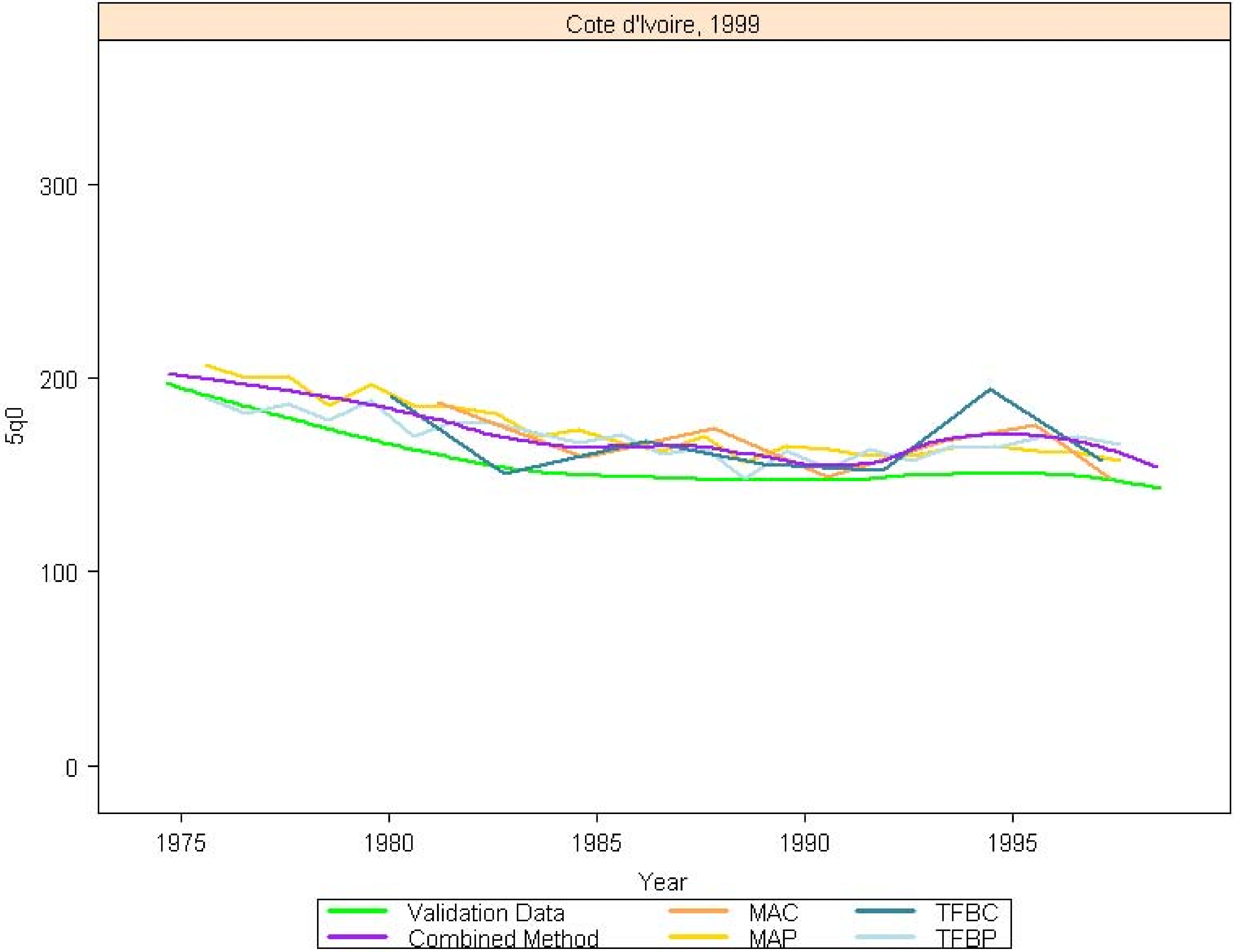
Cote d'Ivoire, 1999
300
200
100
0
5q0
1975
1980
1985
1990
1995
Year
Validation Data
Combined Method
MAC
MAP
TFBC
TFBP

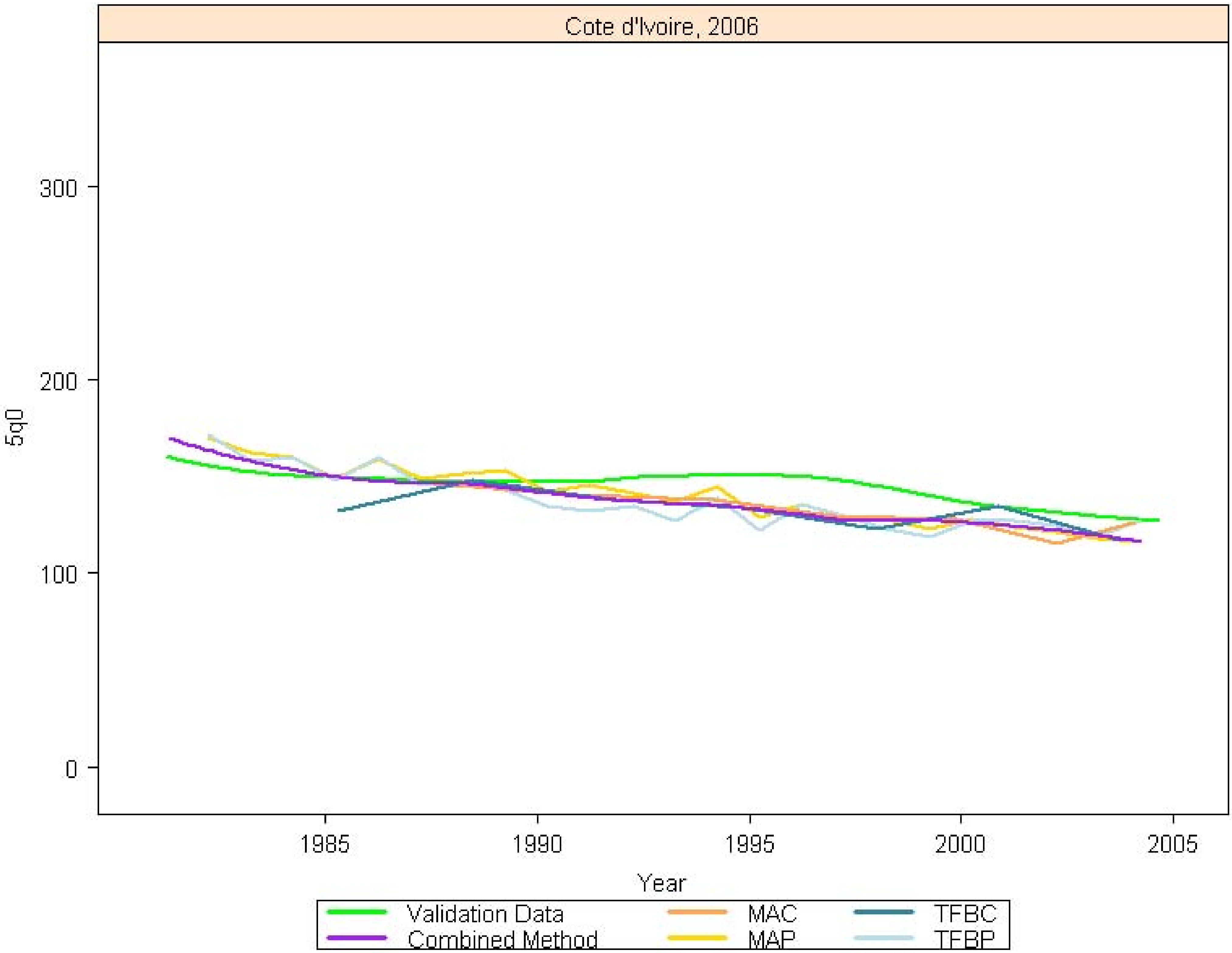

Cote d'Ivoire, 2006
300
200
100
0
5q0
1985
1990
1995
2000
2005
Year
Validation Data
Combined Method
MAC
MAP
TFBC
TFBP

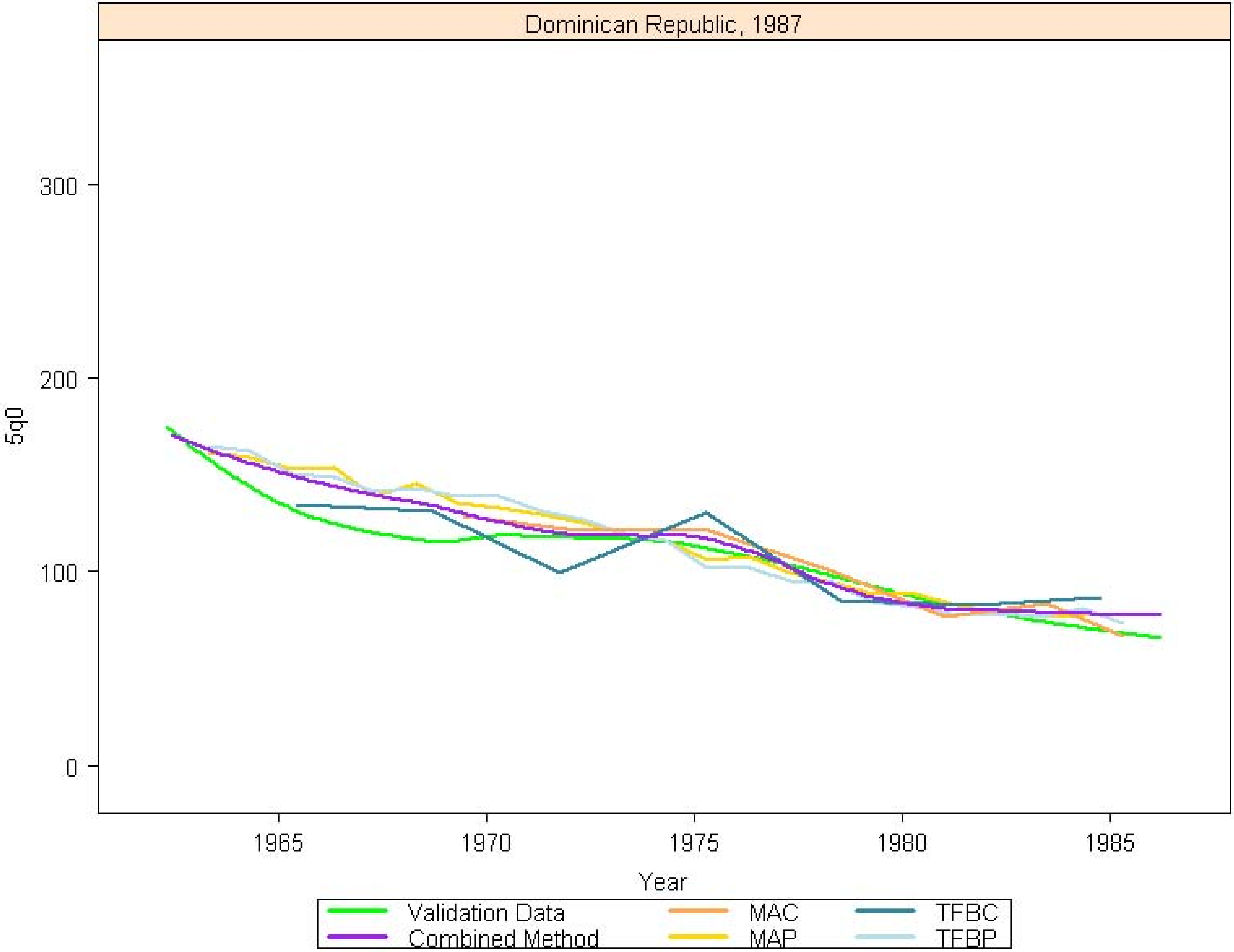
Dominican Republic, 1987
300
200
100
0
5q0
1965
1970
1975
1980
1985
Year
Validation Data
Combined Method
MAC
MAP
TFBC
TFBP

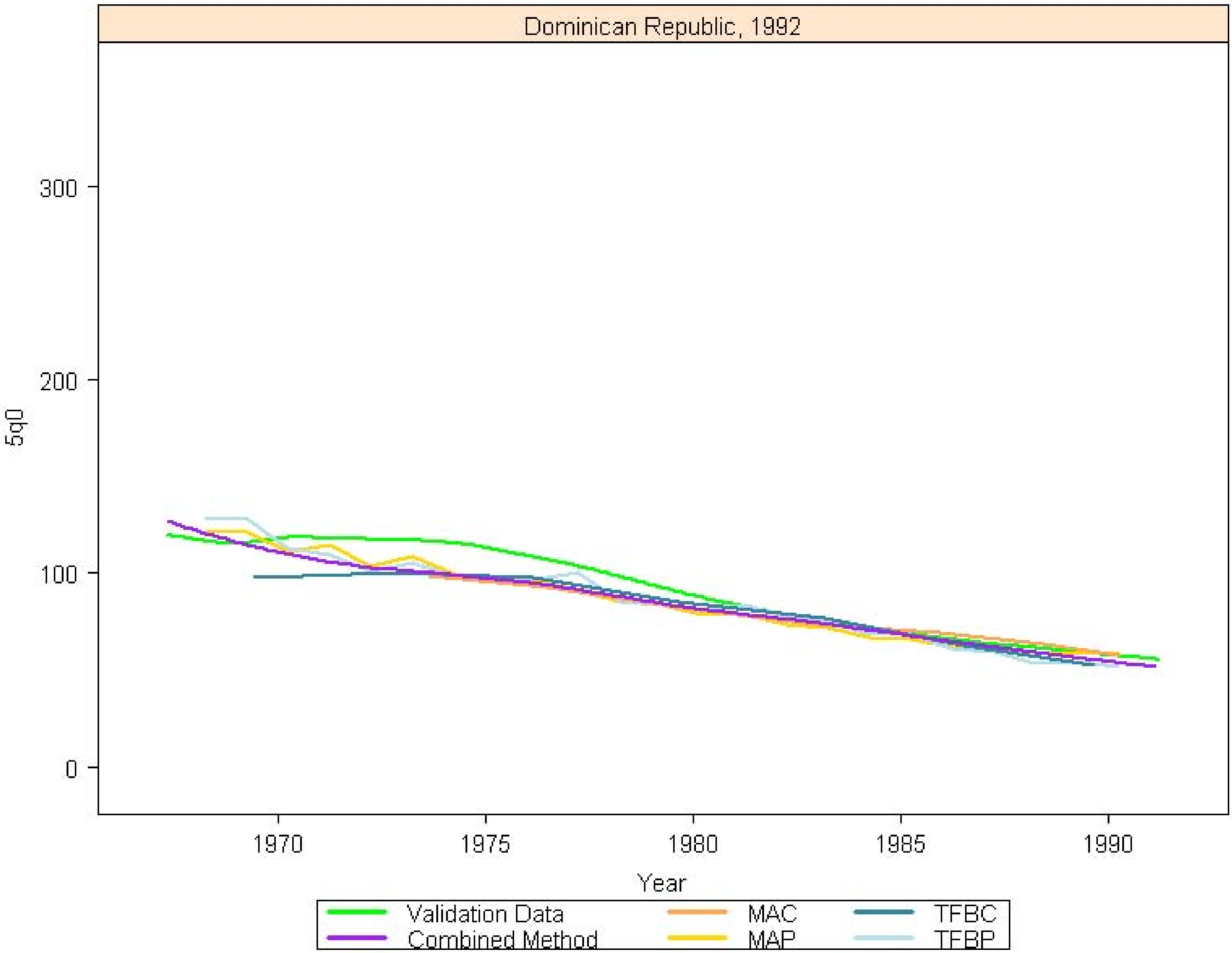

Dominican Republic, 1992
300
200
100
0
5q0
1970
1975
1980
1985
1990
Year
Validation Data
Combined Method
MAC
MAP
TFBC
TFBP

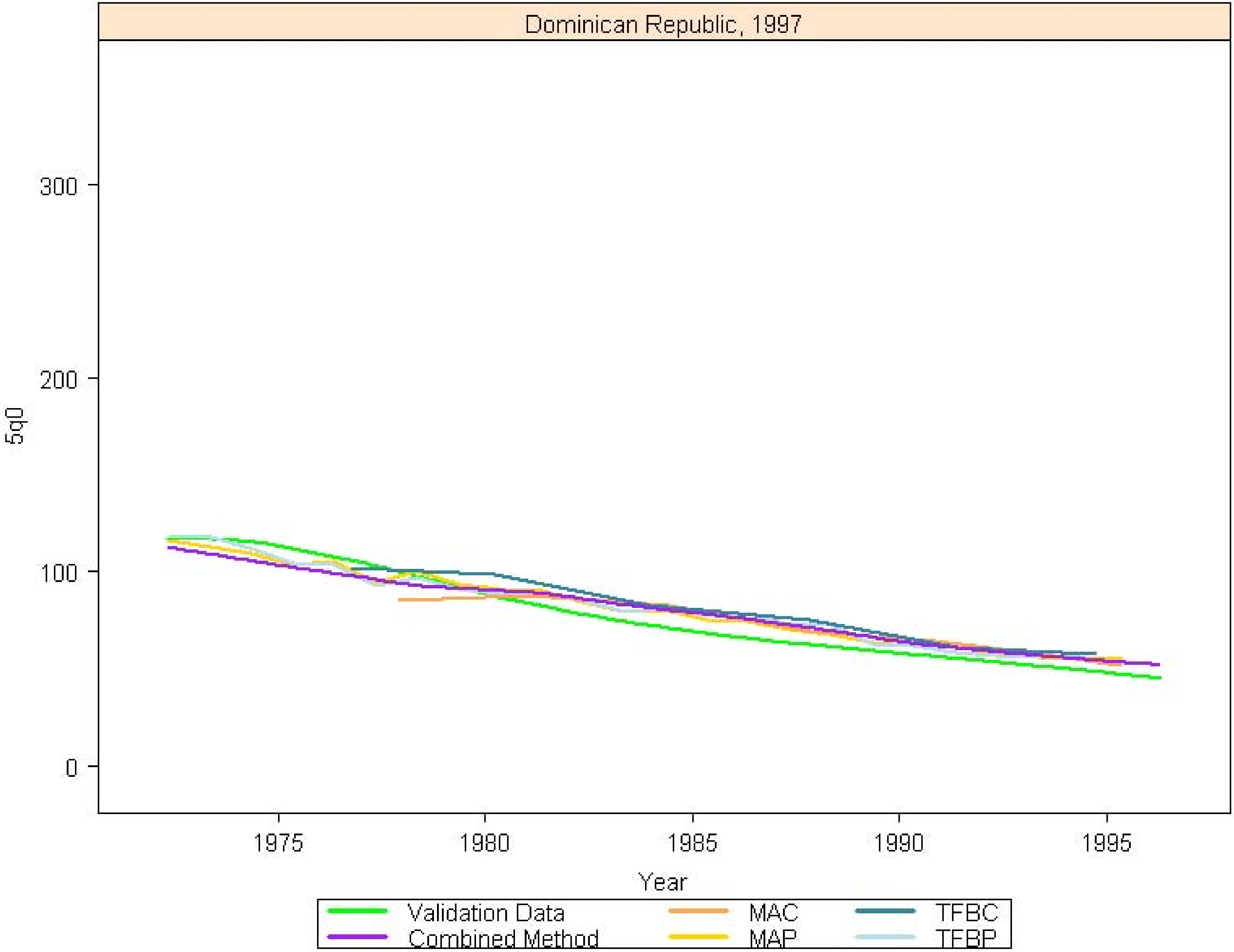

Dominican Republic, 1997
5q0
300
200
100
0
1975
1980
1985
1990
1995
Year
Validation Data
Combined Method
MAC
MAP
TFBC
TFBP

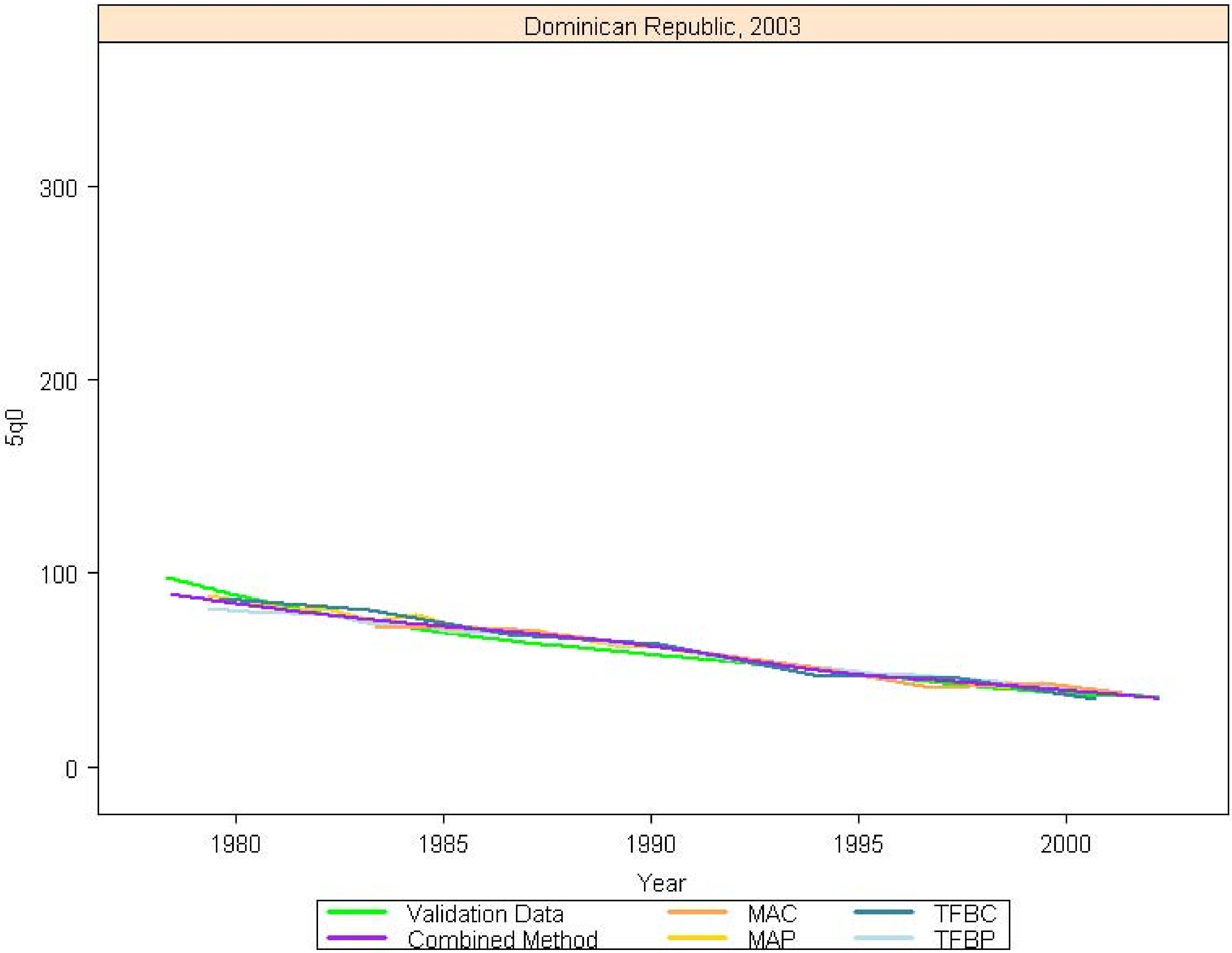

Dominican Republic, 2003
300
200
100
0
5q0
1980
1985
1990
1995
2000
Year
Validation Data
Combined Method
MAC
MAP
TFBC
TFBP

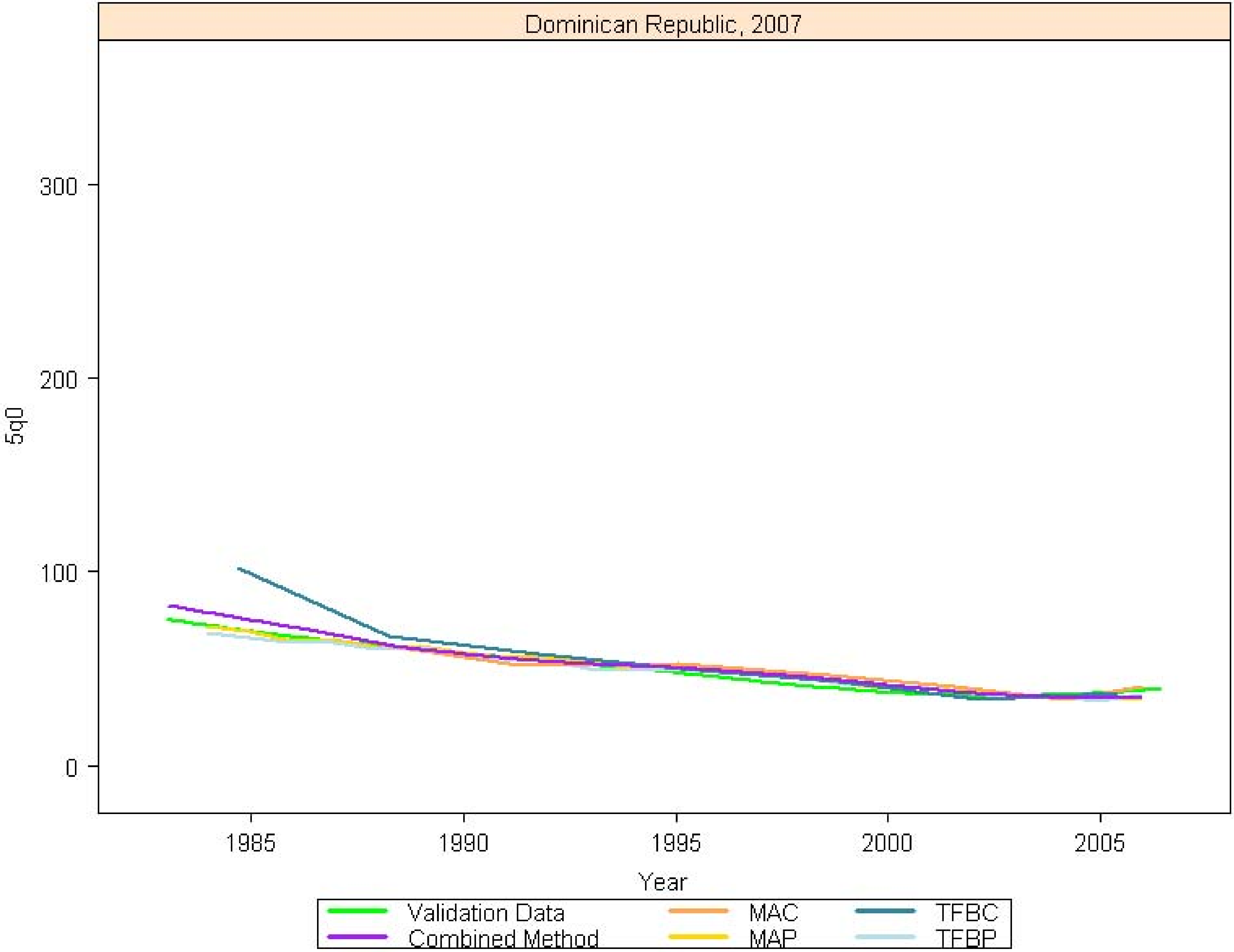

Dominican Republic, 2007
5q0
300
200
100
0
1985
1990
1995
2000
2005
Year
Validation Data
Combined Method
MAC
MAP
TFBC
TFBP

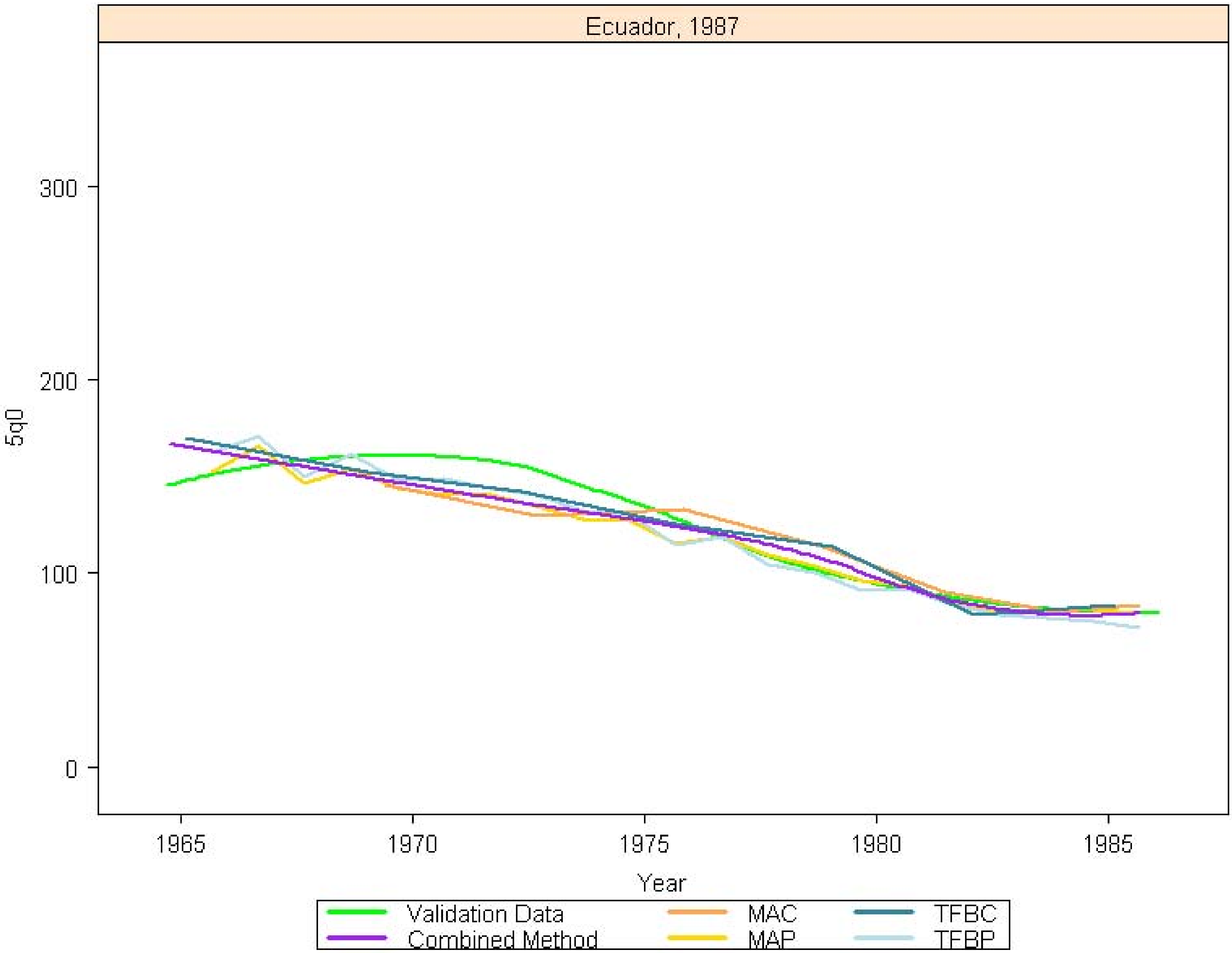

Ecuador, 1987
300
200
100
0
5q0
1965
1970
1975
1980
1985
Year
Validation Data
Combined Method
MAC
MAP
TFBC
TFBP

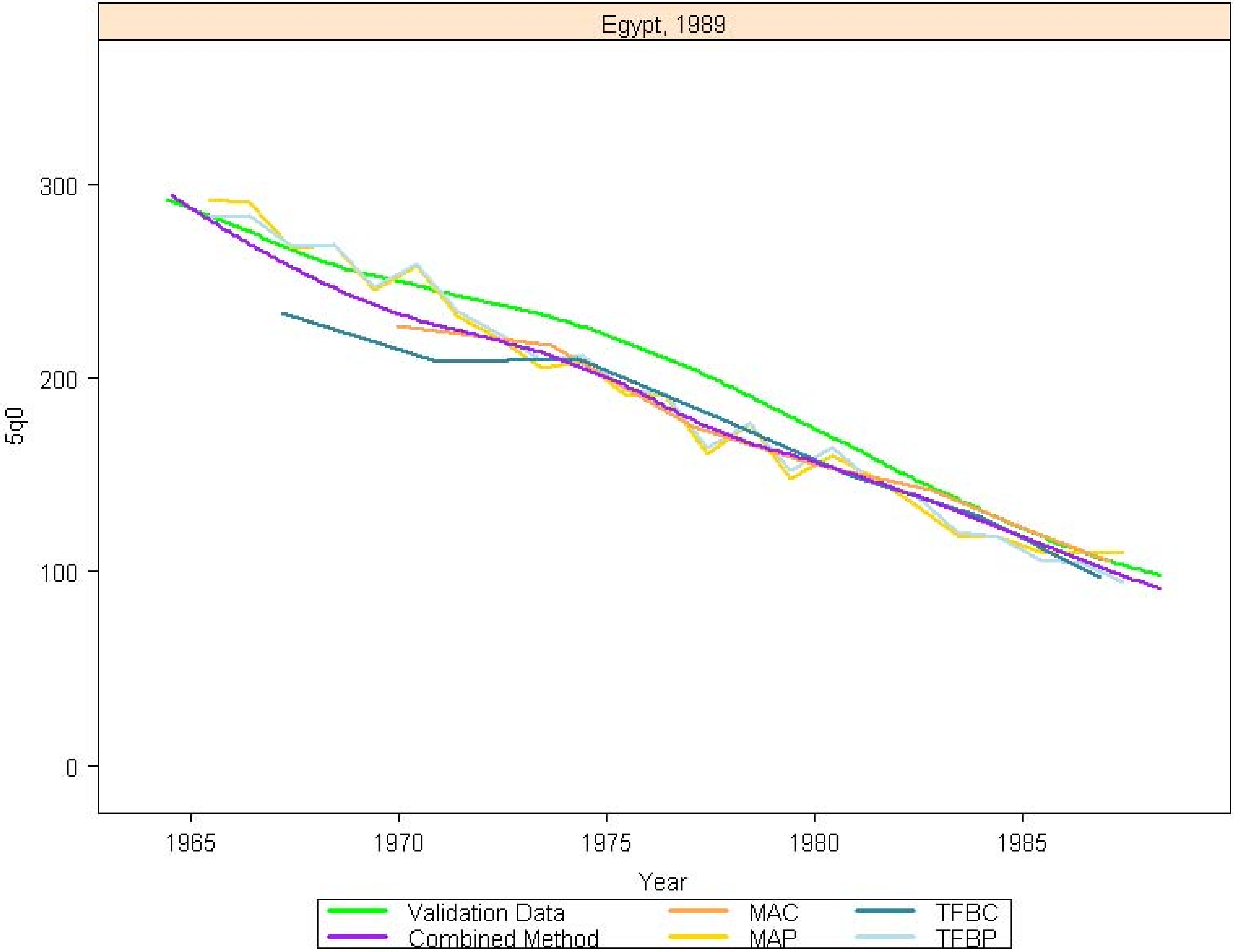

Egypt, 1989
300
200
100
0
5q0
1965
1970
1975
1980
1985
Year
Validation Data
Combined Method
MAC
MAP
TFBC
TFBP

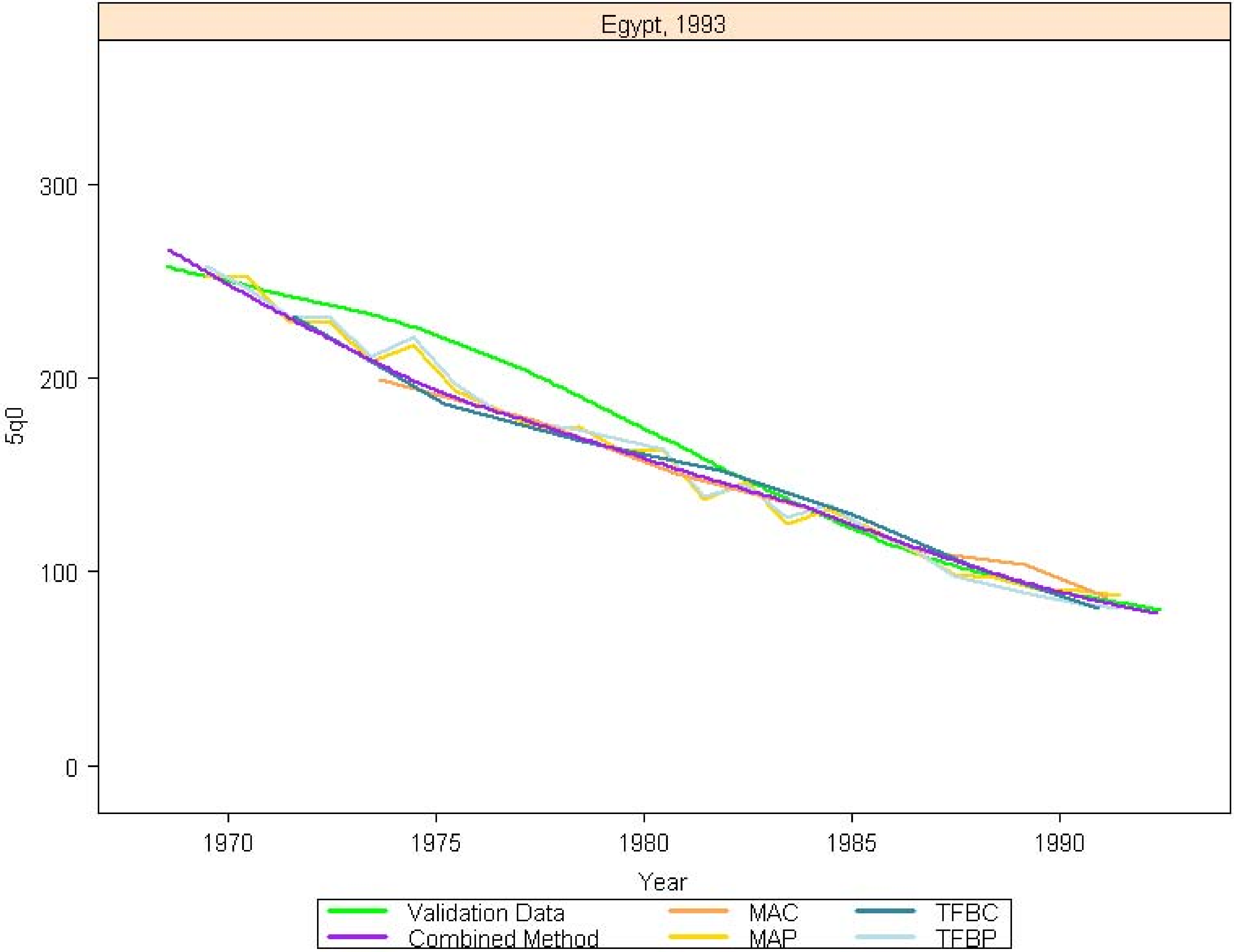
Egypt, 1993
5q0
0
100
200
300
1970
1975
1980
1985
1990
Year
Validation Data
Combined Method
MAC
MAP
TFBC
TFBP

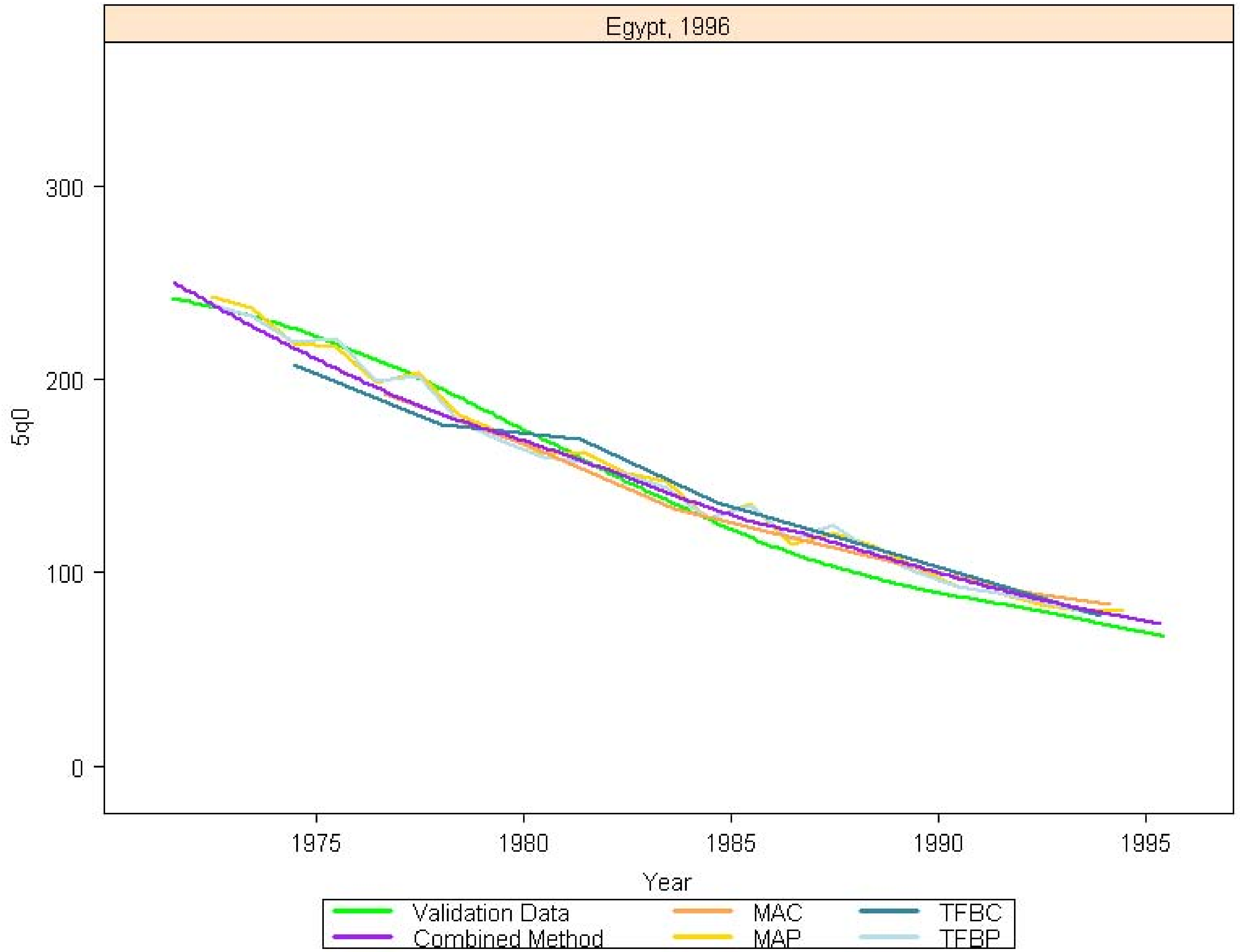

Egypt, 1996
5q0
300
200
100
0
1975
1980
1985
1990
1995
Year
Validation Data
Combined Method
MAC
MAP
TFBC
TFBP

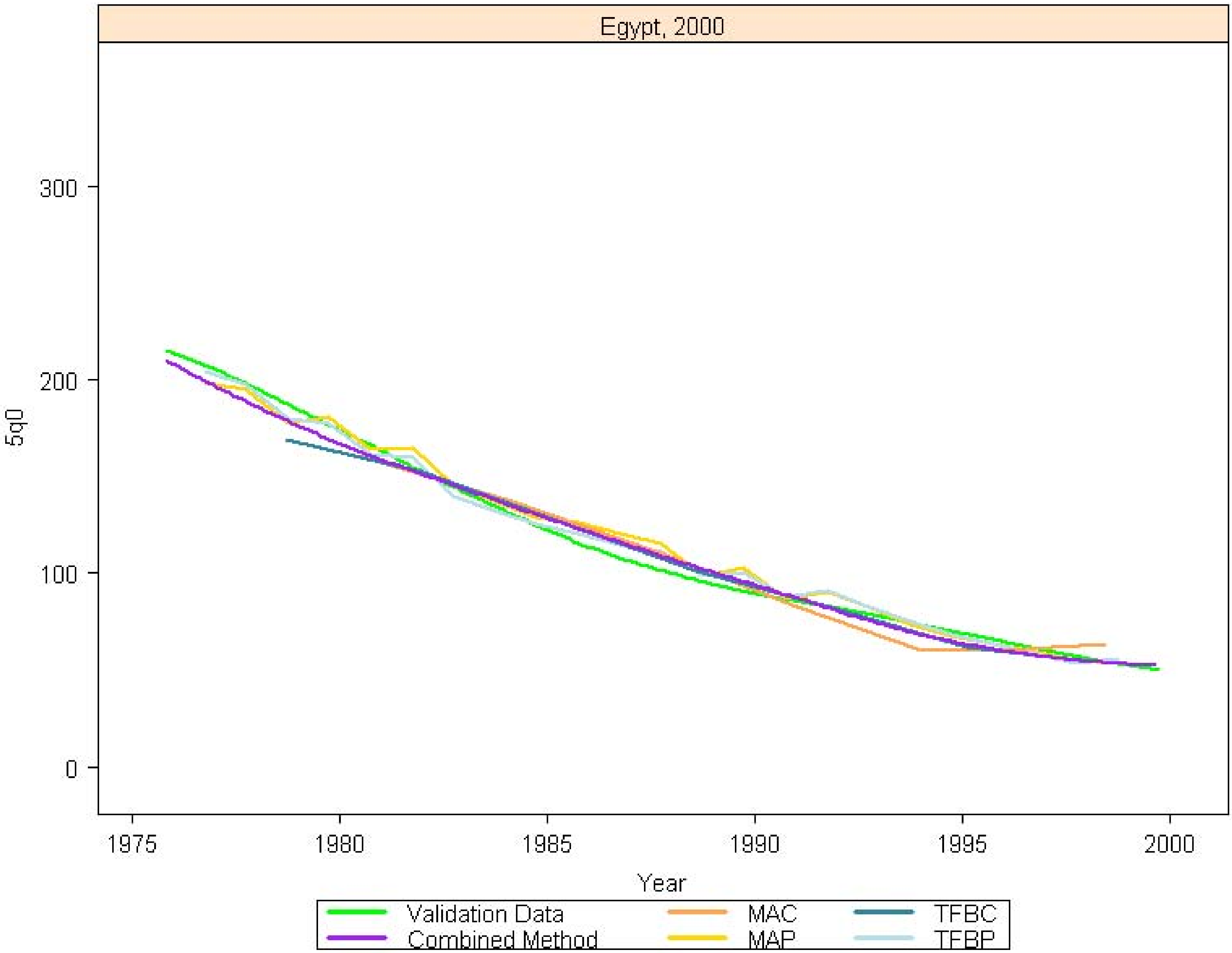
Egypt, 2000
5q0
300
200
100
0
1975
1980
1985
1990
1995
2000
Year
Validation Data
Combined Method
MAC
MAP
TFBC
TFBP

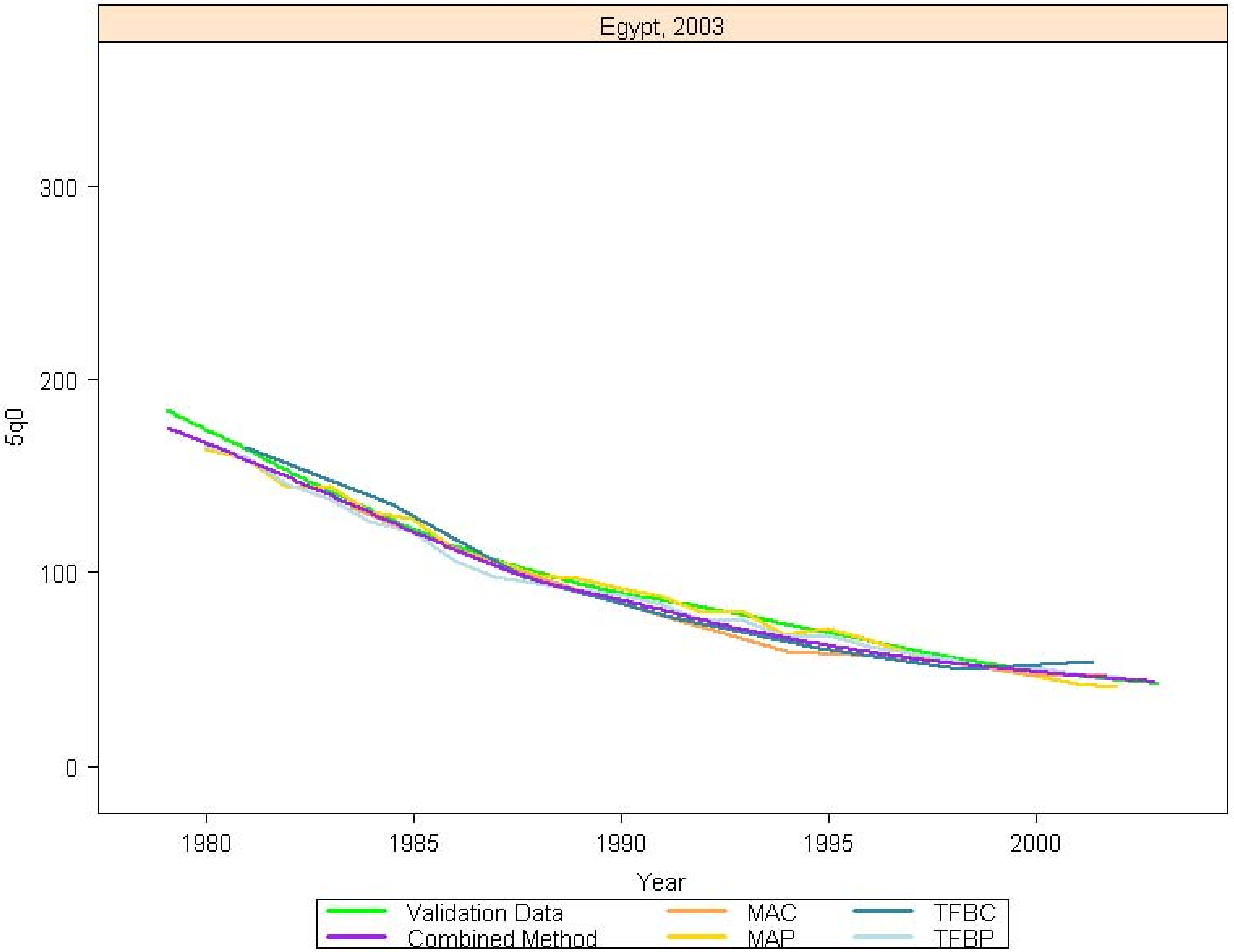

Egypt, 2003
300
200
100
0
5q0
1980
1985
1990
1995
2000
Year
Validation Data
Combined Method
MAC
MAP
TFBC
TFBP

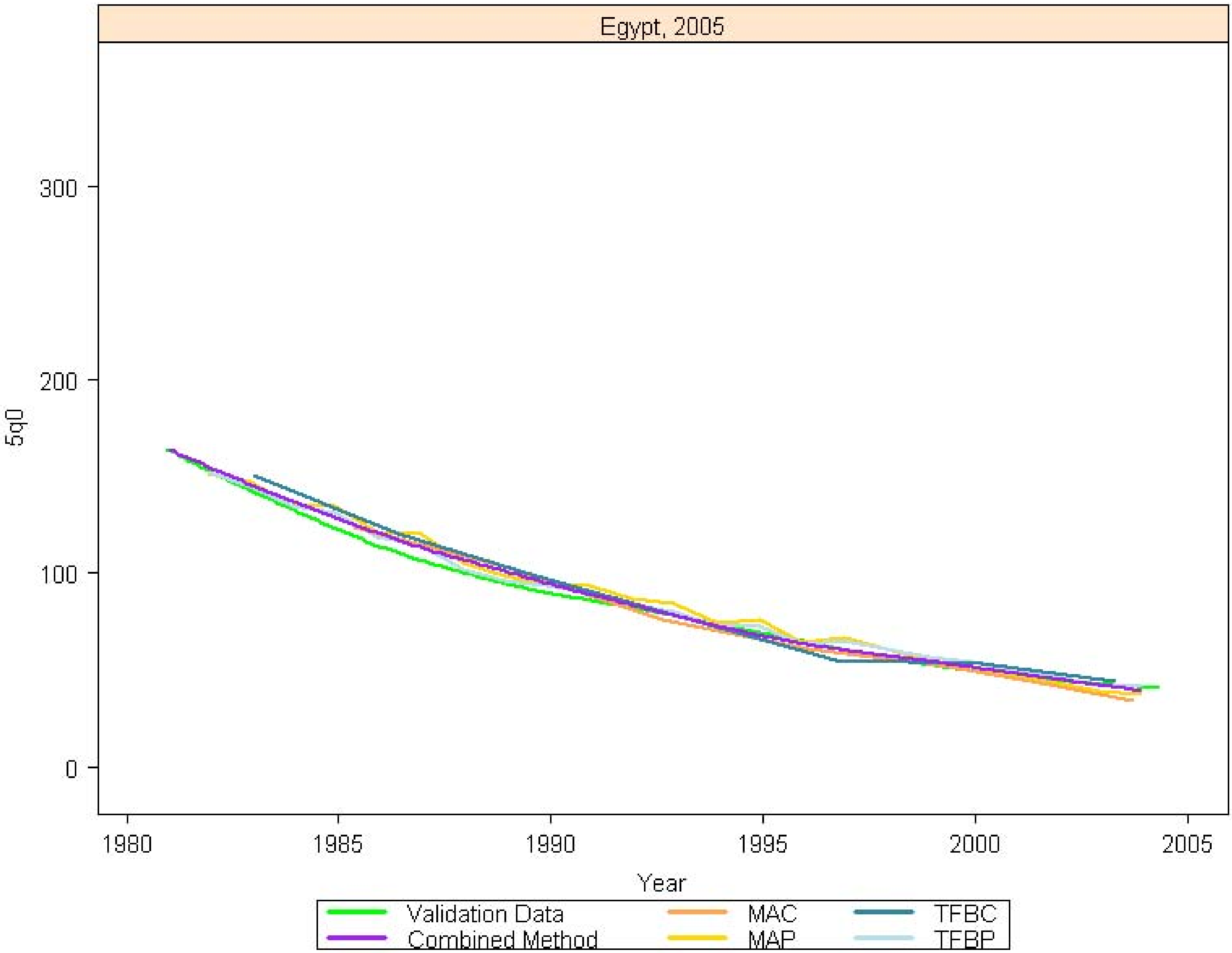
Egypt, 2005
300
200
100
0
5q0
1980
1985
1990
1995
2000
2005
Year
Validation Data
Combined Method
MAC
MAP
TFBC
TFBP

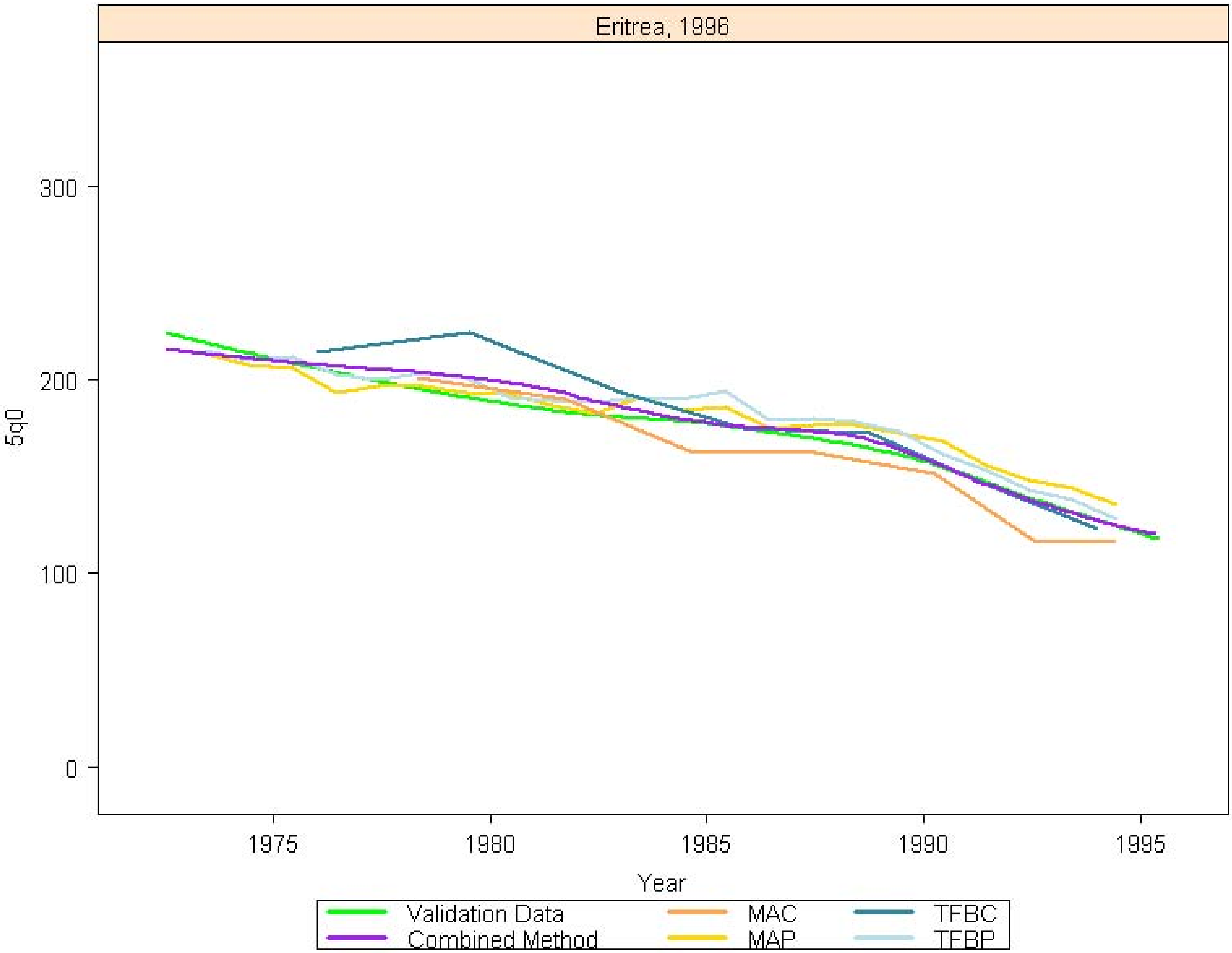

Eritrea, 1996
300
200
100
0
5q0
1975
1980
1985
1990
1995
Year
Validation Data
Combined Method
MAC
MAP
TFBC
TFBP

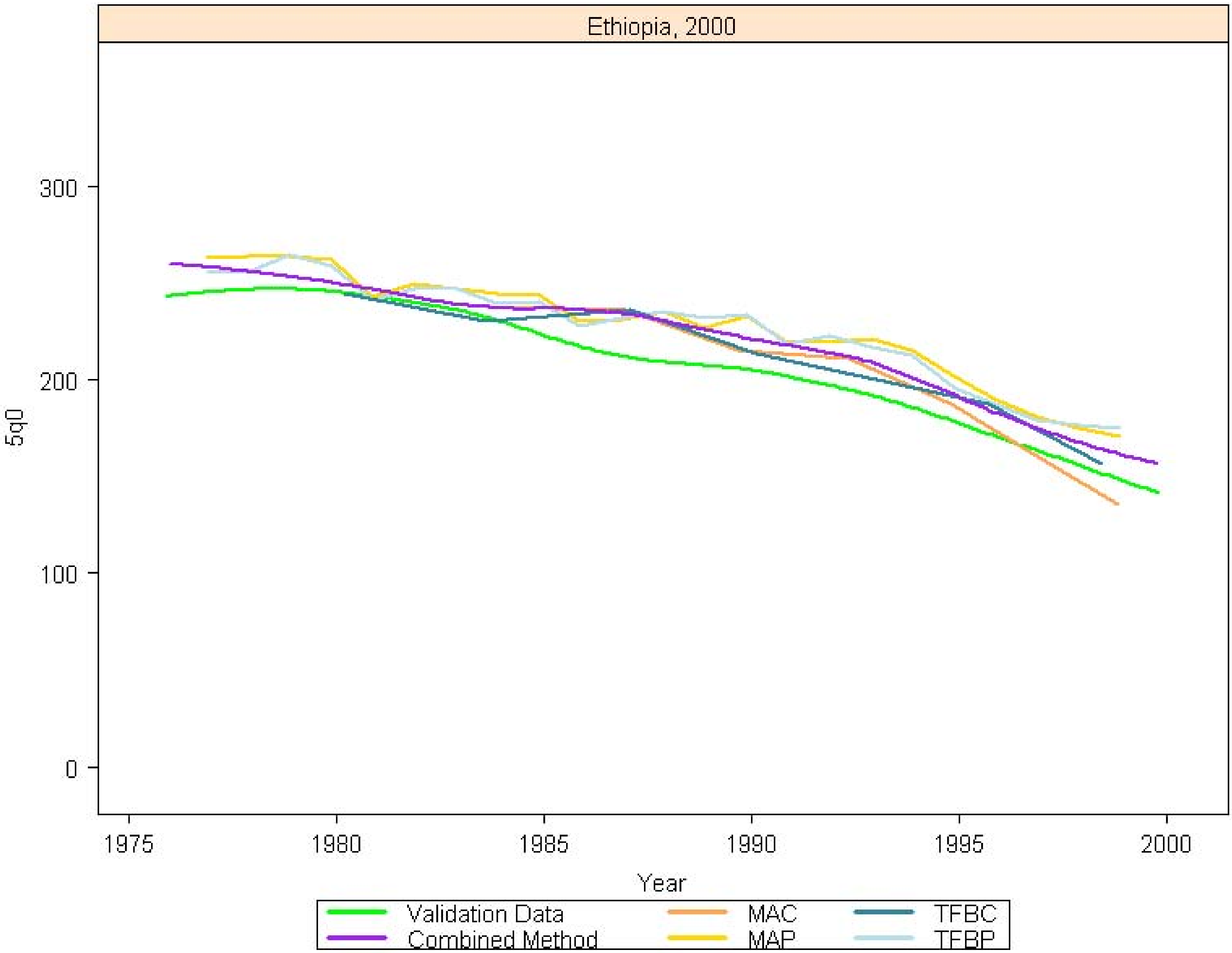
Ethiopia, 2000
300
200
100
0
5q0
1975
1980
1985
1990
1995
2000
Year
Validation Data
Combined Method
MAC
MAP
TFBC
TFBP

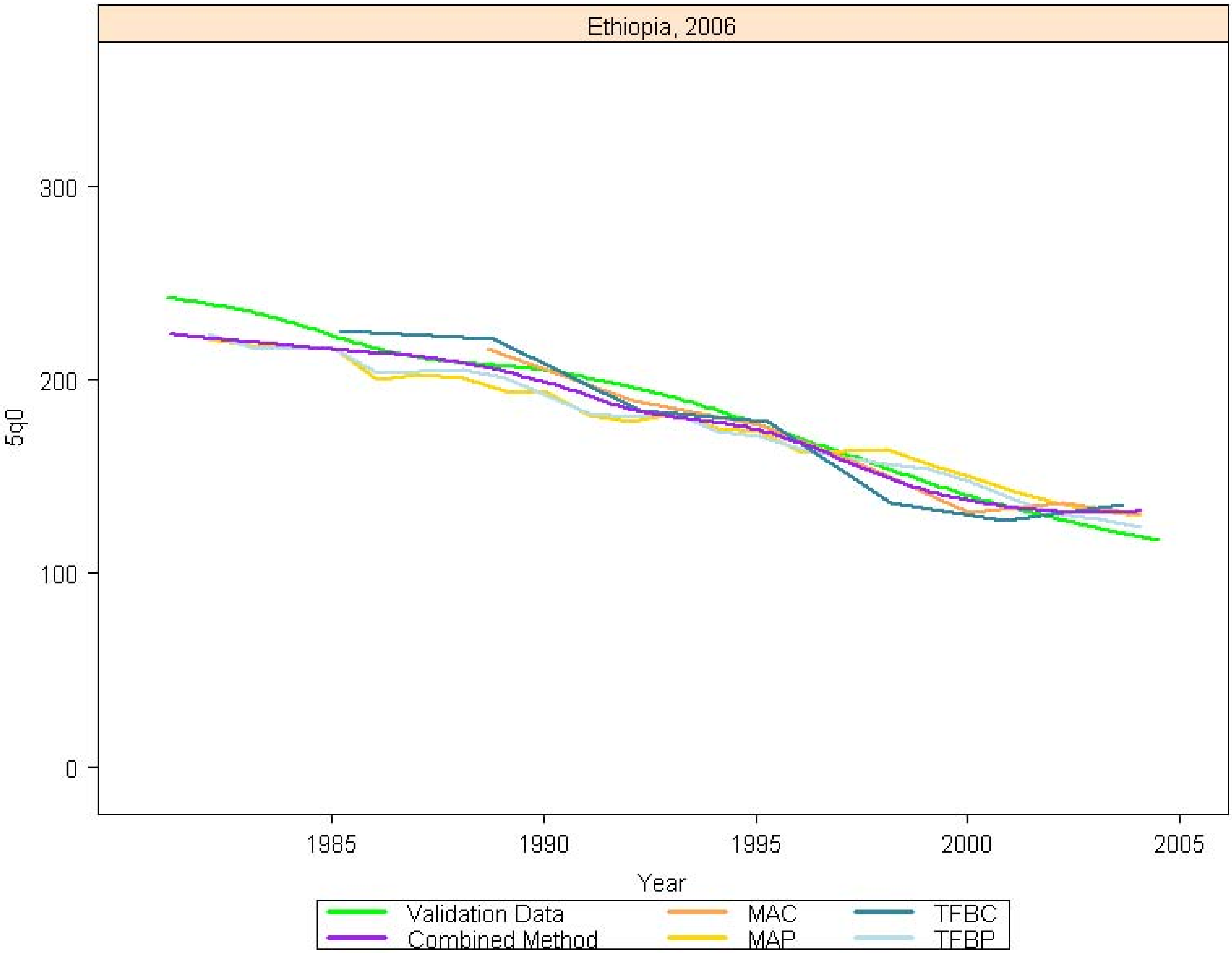

Ethiopia, 2006
5q0
300
200
100
0
1985
1990
1995
2000
2005
Year
Validation Data
Combined Method
MAC
MAP
TFBC
TFBP

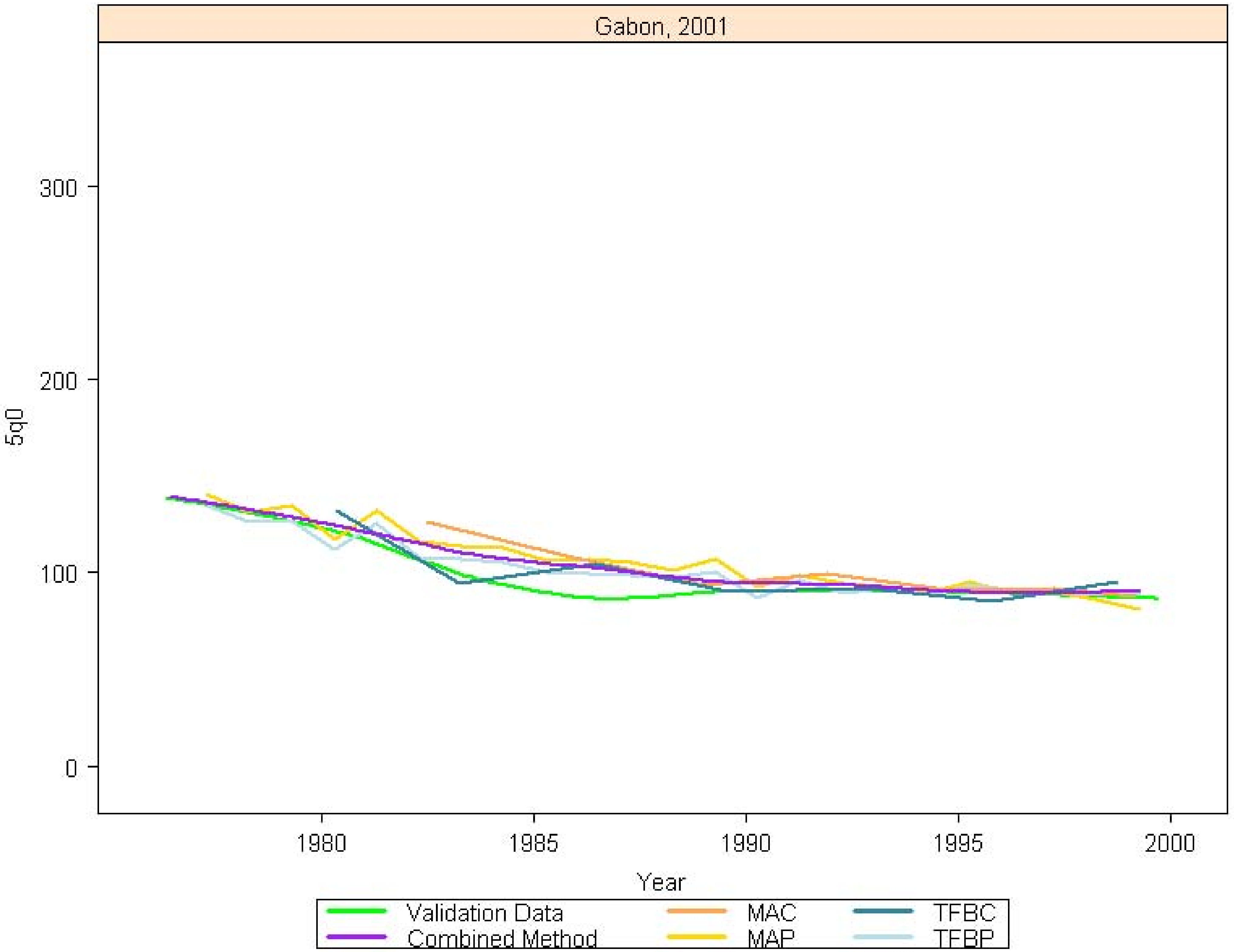
Gabon, 2001
5q0
300
200
100
0
1980
1985
1990
1995
2000
Year
Validation Data
Combined Method
MAC
MAP
TFBC
TFBP

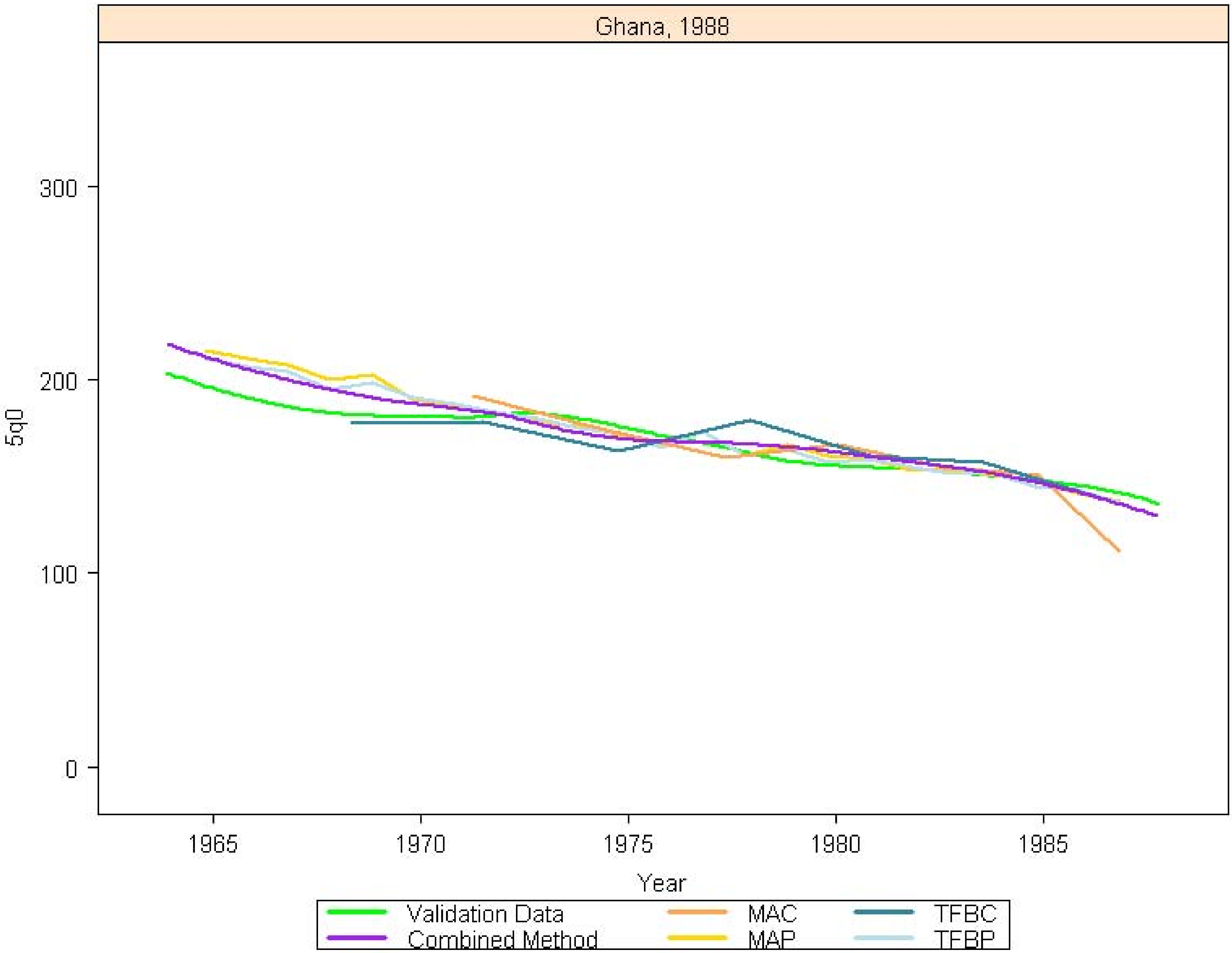

Ghana, 1988
300
200
100
0
5q0
1965
1970
1975
1980
1985
Year
Validation Data
Combined Method
MAC
MAP
TFBC
TFBP

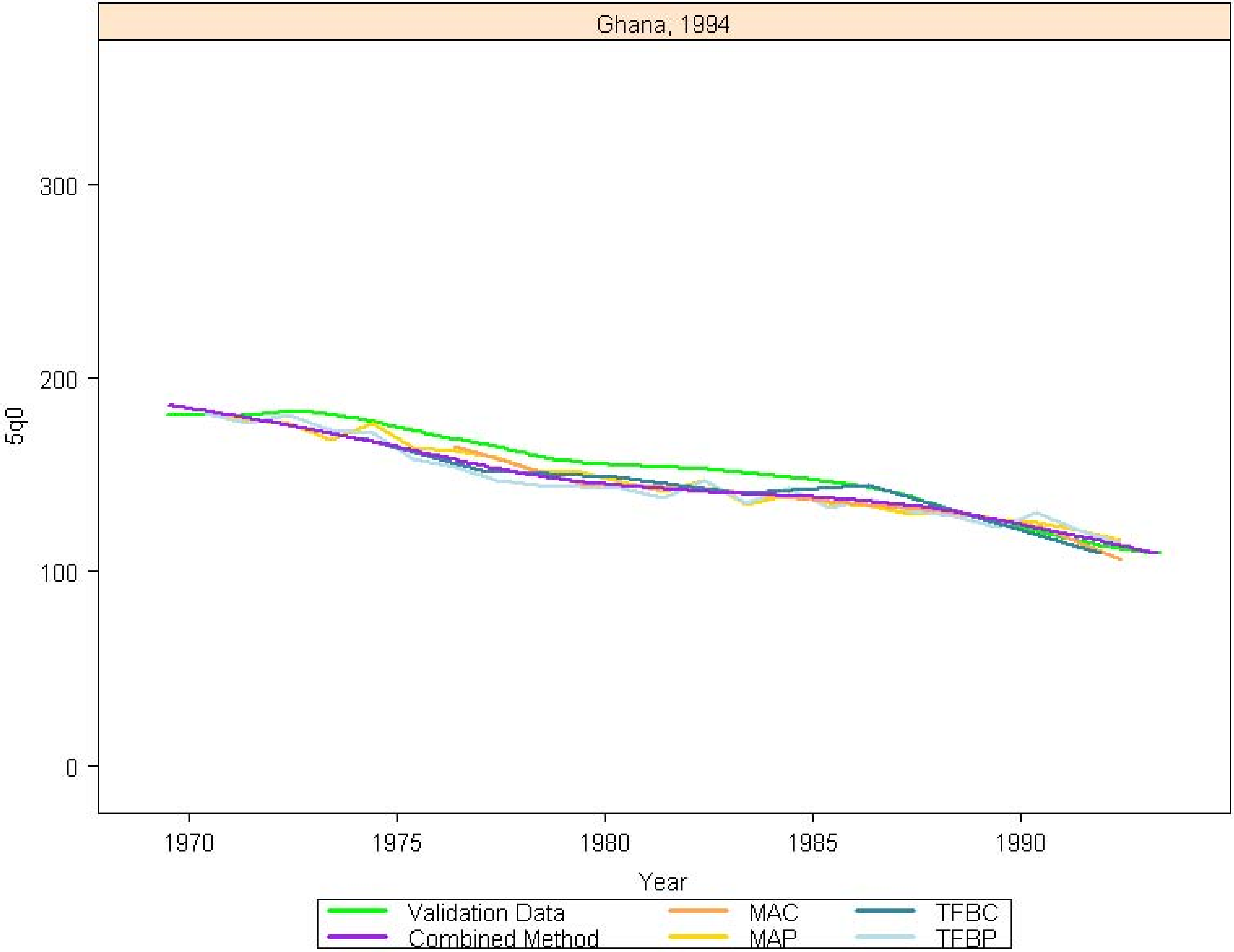

Ghana, 1994
300
200
100
0
5q0
1970
1975
1980
1985
1990
Year
Validation Data
Combined Method
MAC
MAP
TFBC
TFBP

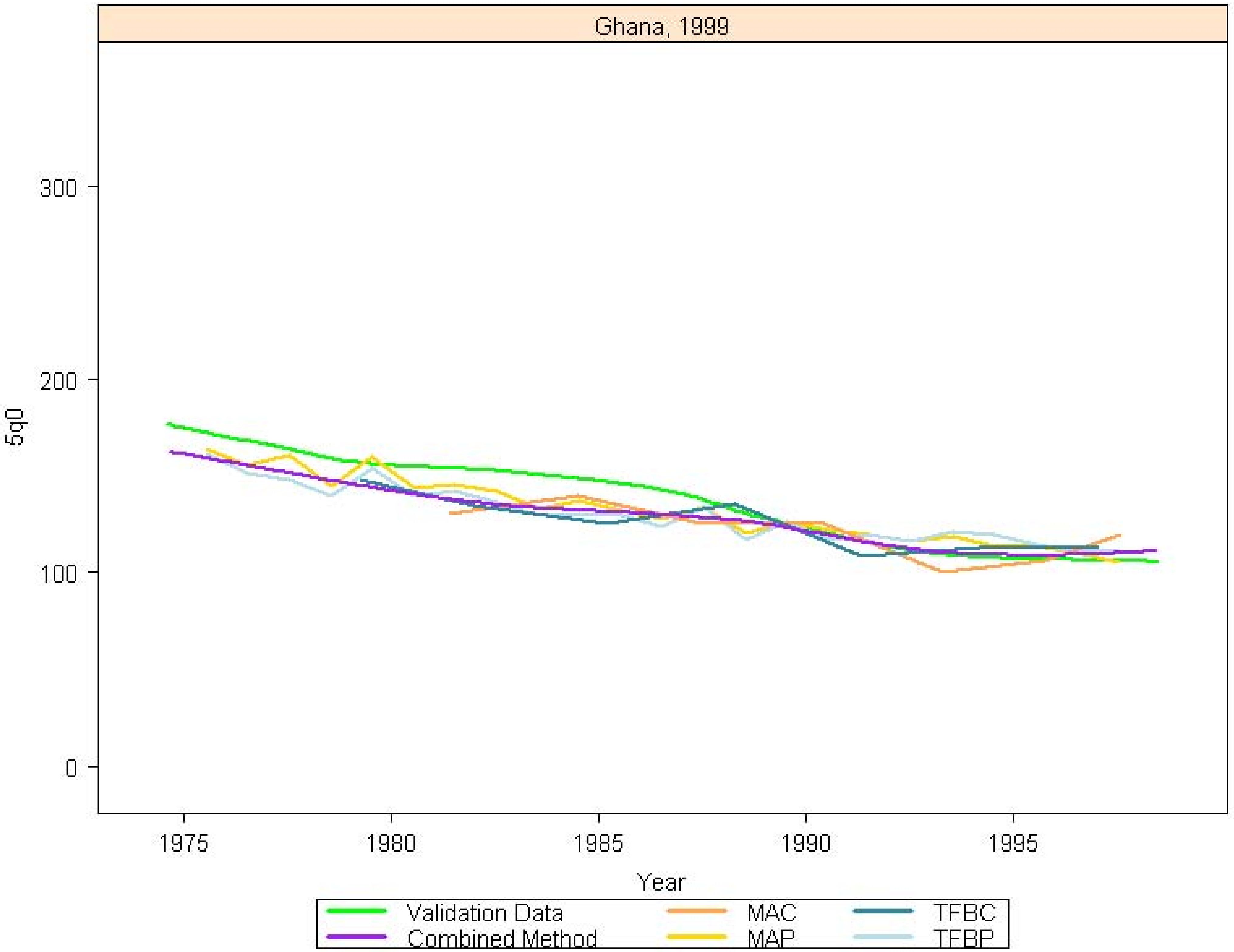
Ghana, 1999
5q0
300
200
100
0
1975
1980
1985
1990
1995
Year
Validation Data
Combined Method
MAC
MAP
TFBC
TFBP

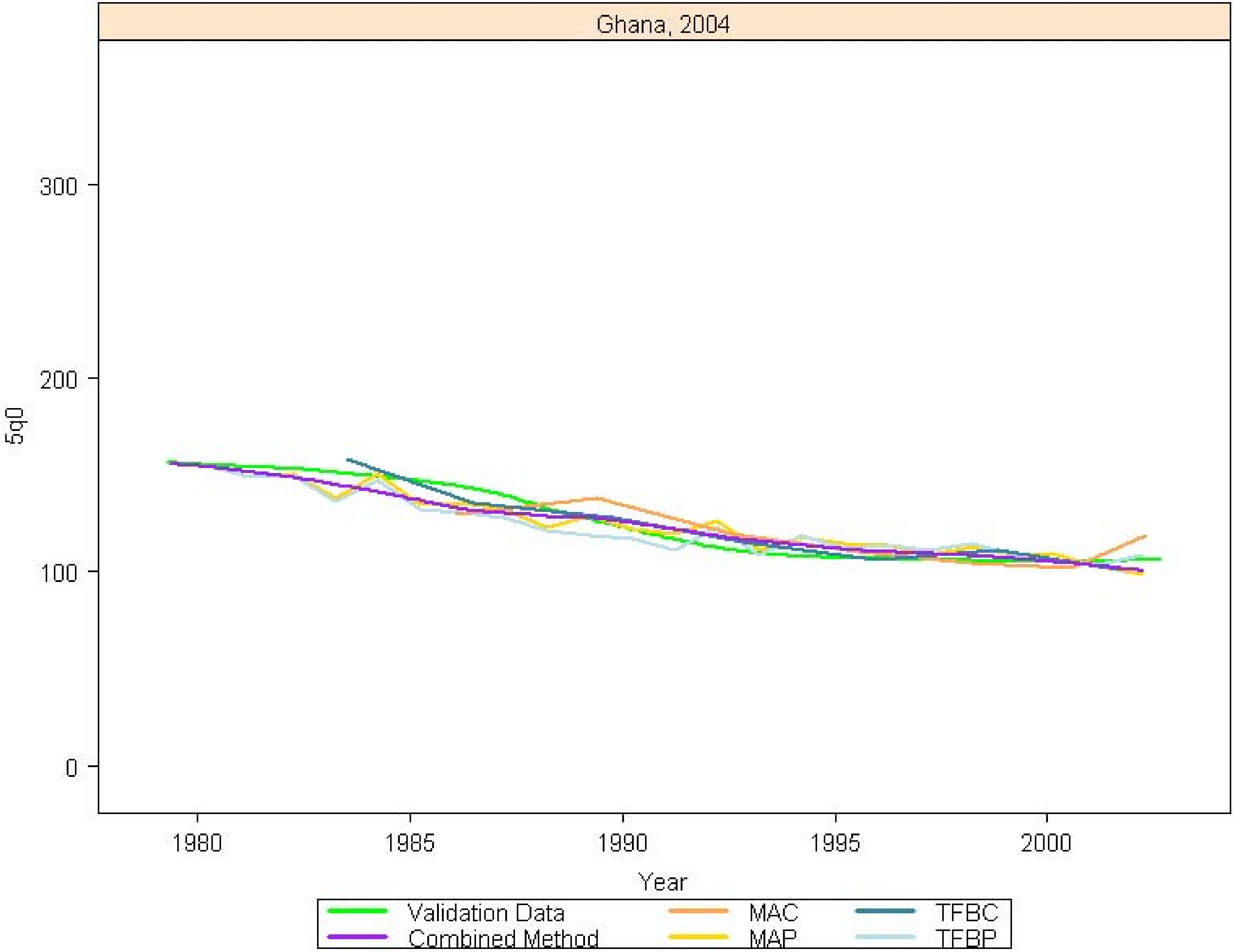
Ghana, 2004
300
200
100
0
5q0
1980
1985
1990
1995
2000
Year
Validation Data
Combined Method
MAC
MAP
TFBC
TFBP

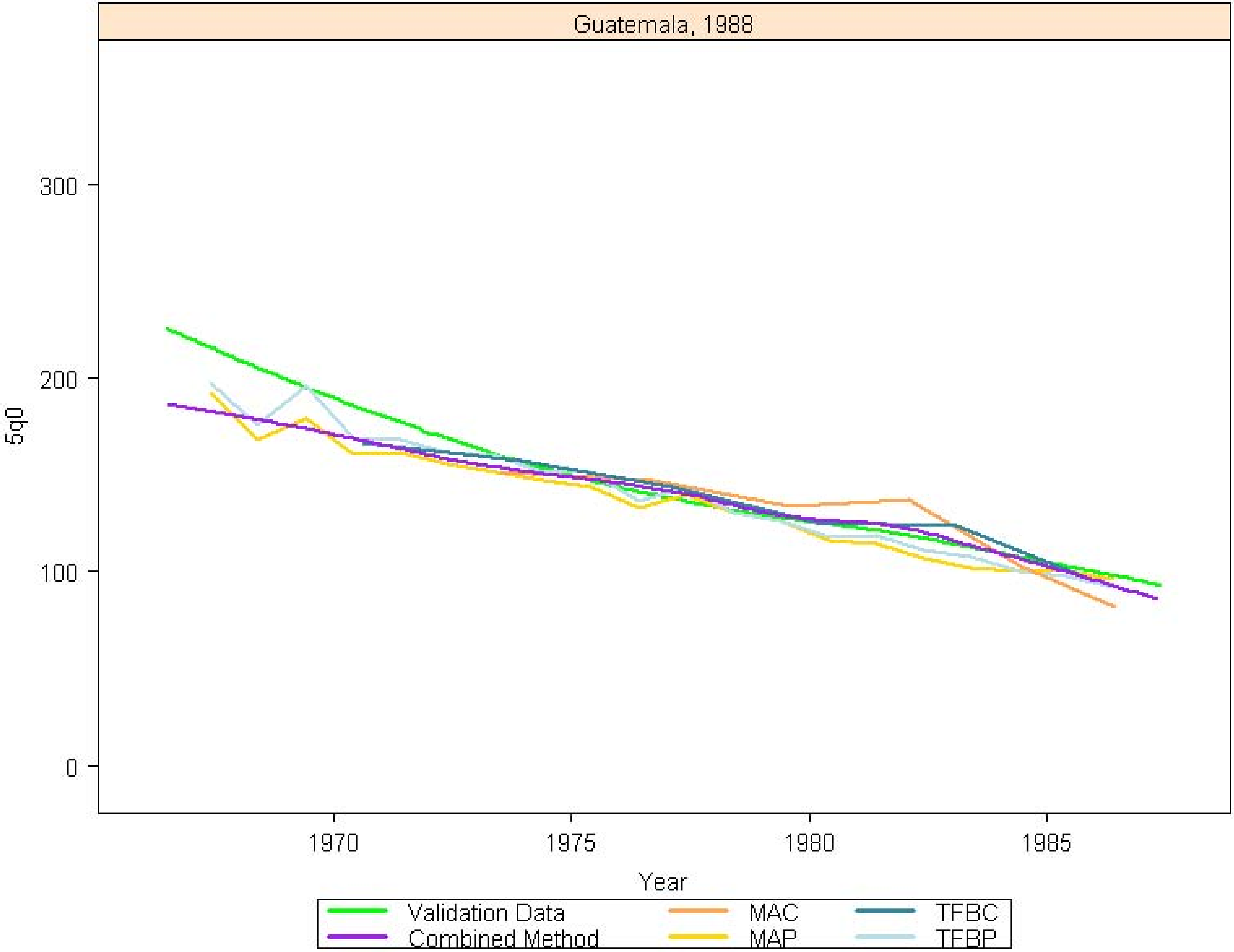
Guatemala, 1988
300
200
100
0
5q0
1970
1975
1980
1985
Year
Validation Data
Combined Method
MAC
MAP
TFBC
TFBP

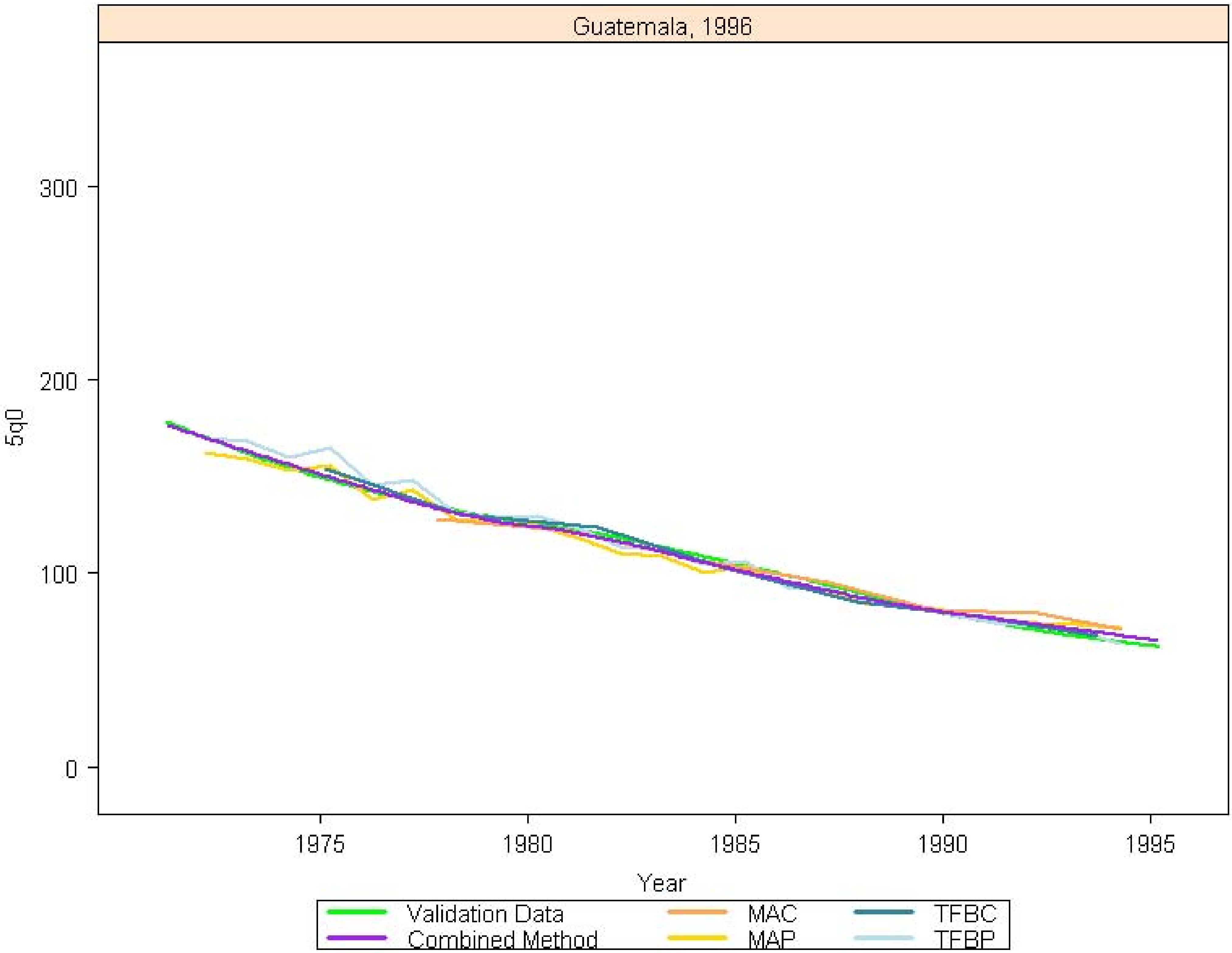
Guatemala, 1996
5q0
0
100
200
300
1975
1980
1985
1990
1995
Year
Validation Data
Combined Method
MAC
MAP
TFBC
TFBP

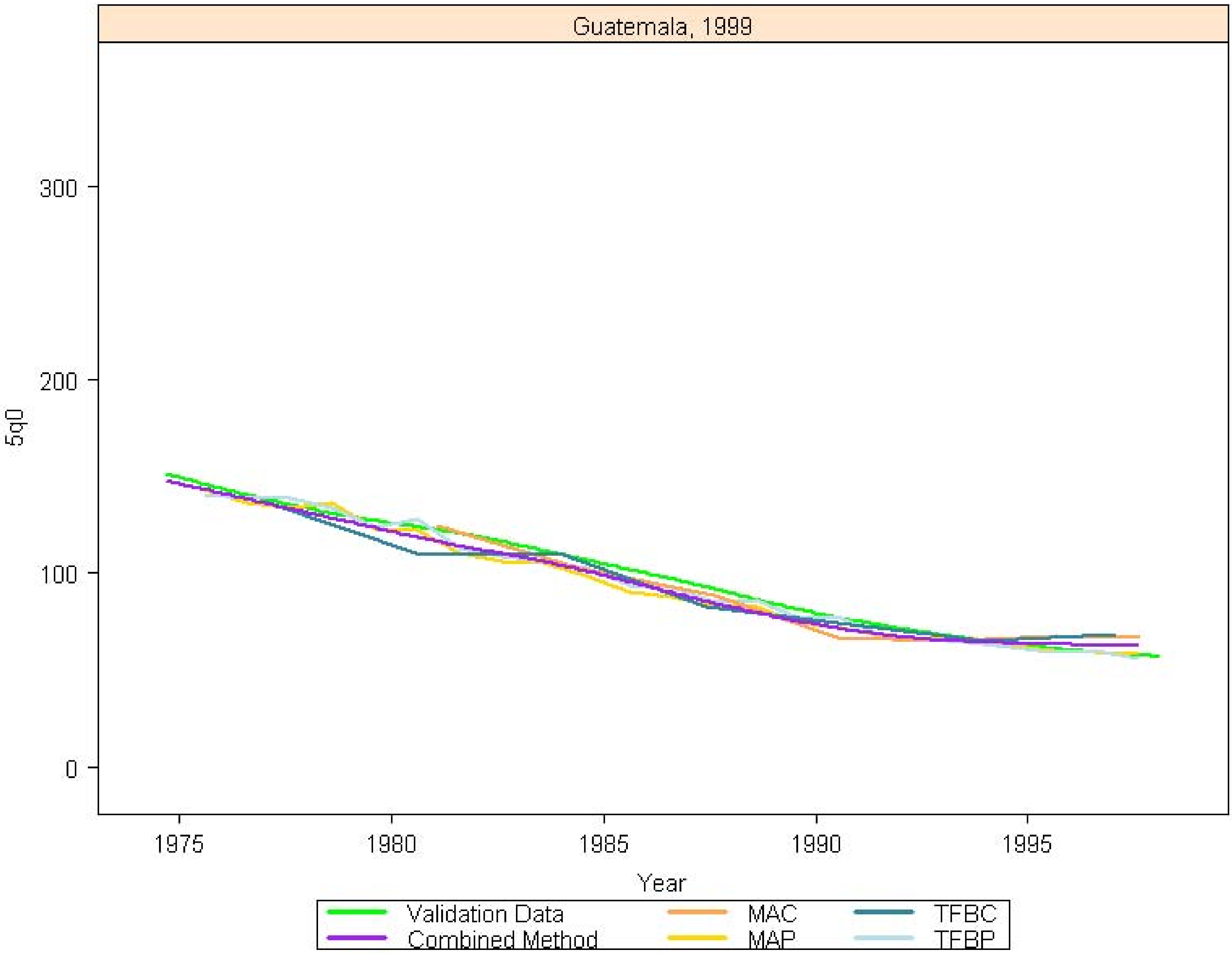
Guatemala, 1999
300
200
100
0
5q0
1975
1980
1985
1990
1995
Year
Validation Data
Combined Method
MAC
MAP
TFBC
TFBP

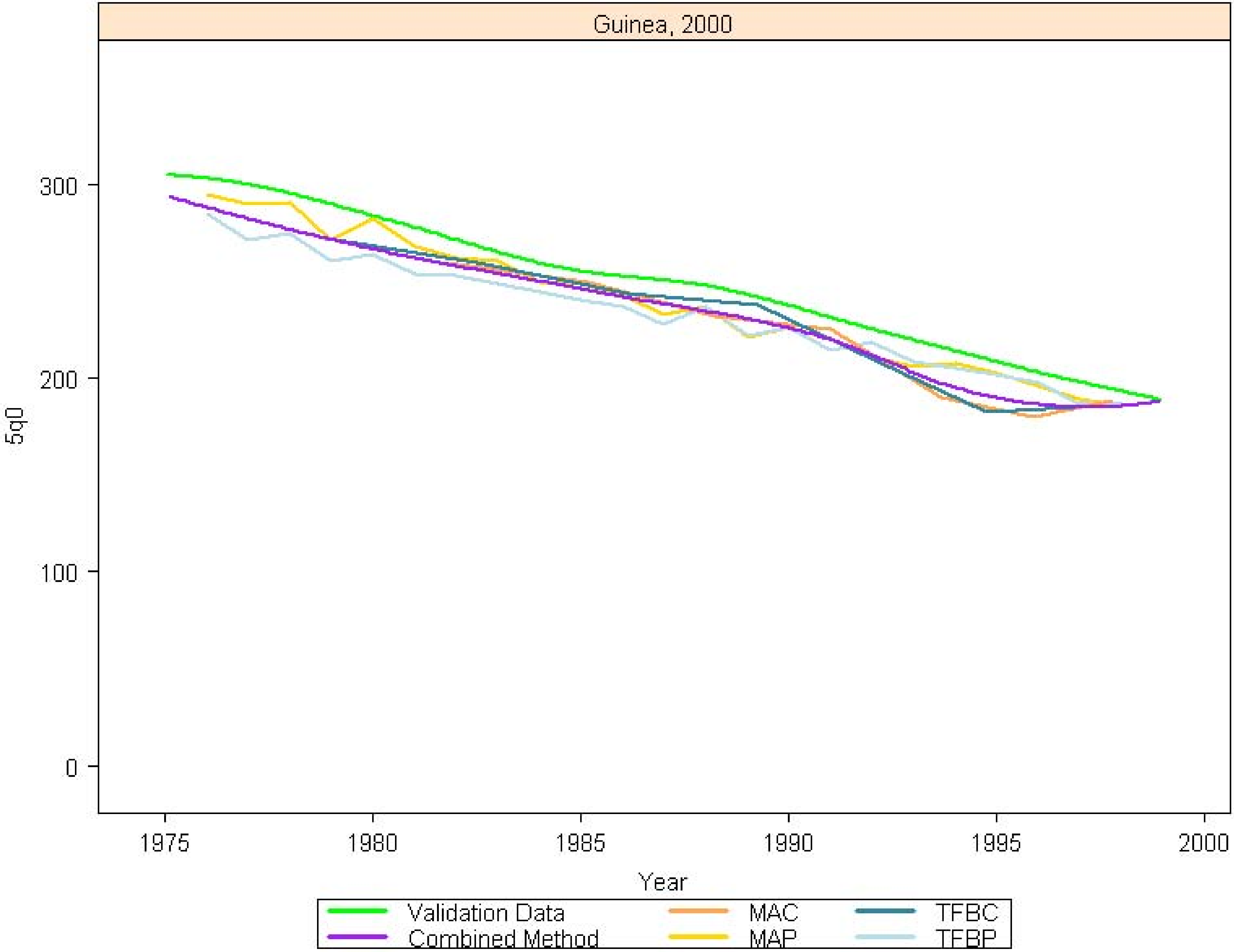

Guinea, 2000
5q0
300
200
100
0
1975
1980
1985
1990
1995
2000
Year
Validation Data
Combined Method
MAC
MAP
TFBC
TFBP

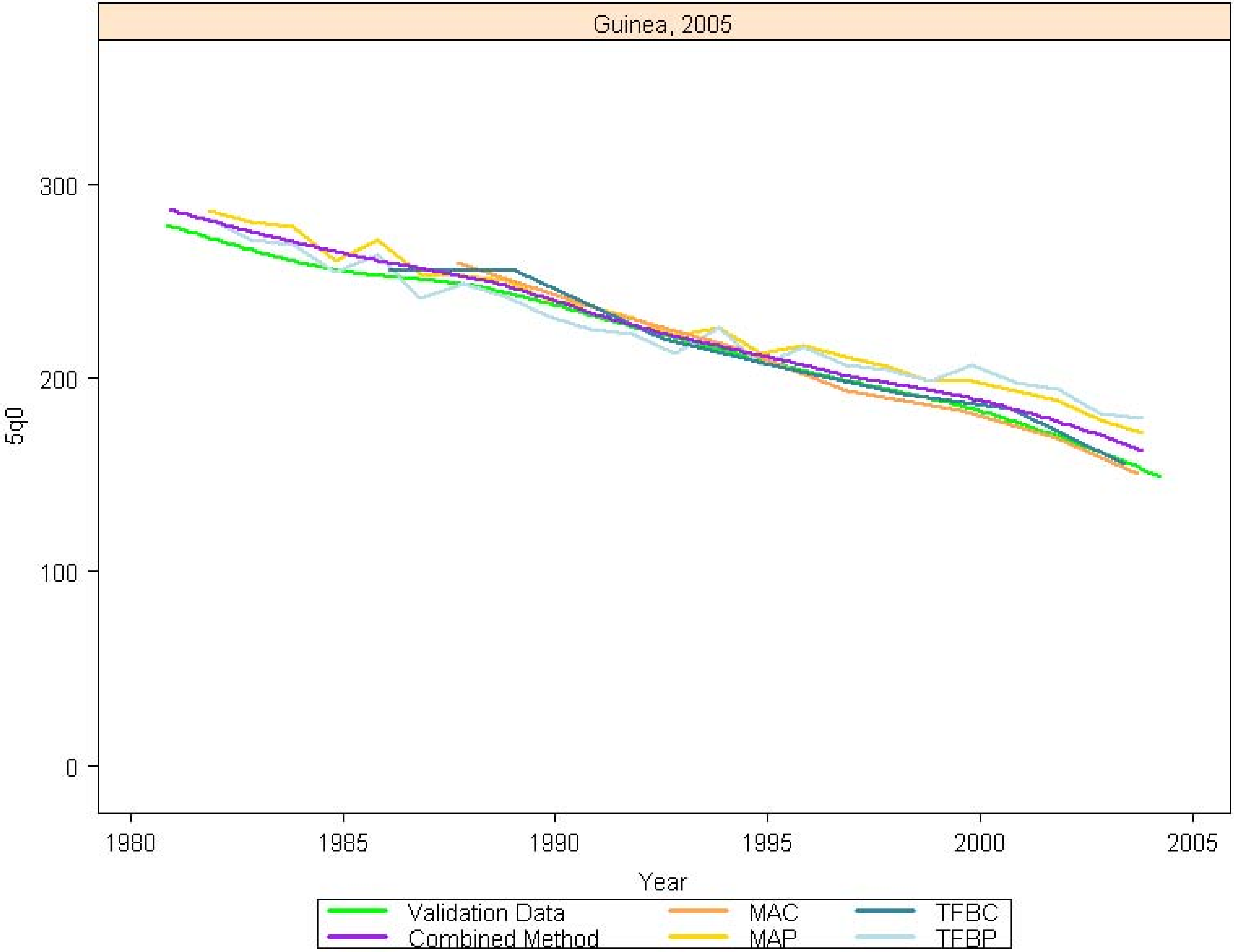

Guinea, 2005
300
200
100
0
5q0
1980
1985
1990
1995
2000
2005
Year
Validation Data
Combined Method
MAC
MAP
TFBC
TFBP

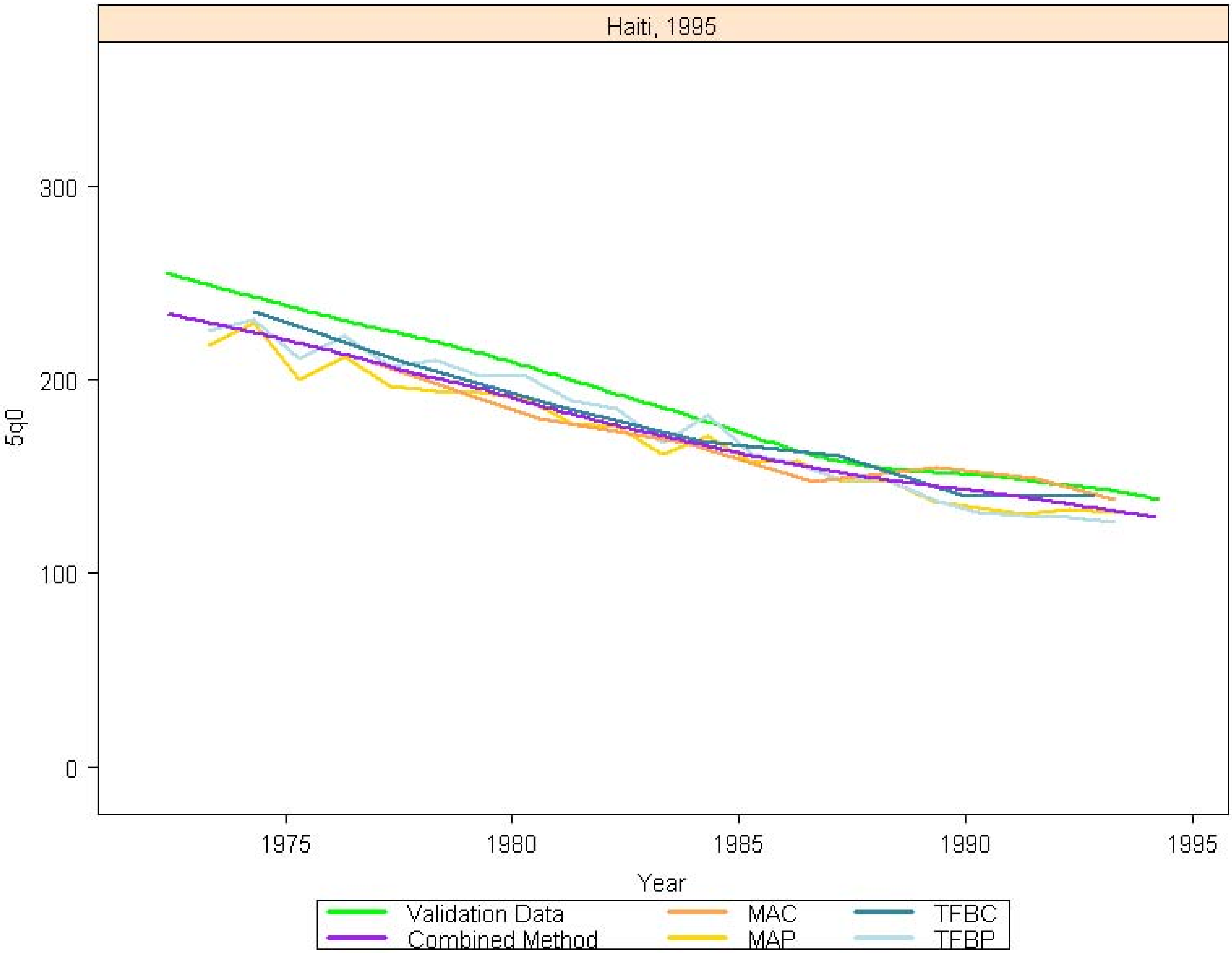
Haiti, 1995
300
200
100
0
5q0
1975
1980
1985
1990
1995
Year
Validation Data
Combined Method
MAC
MAP
TFBC
TFBP

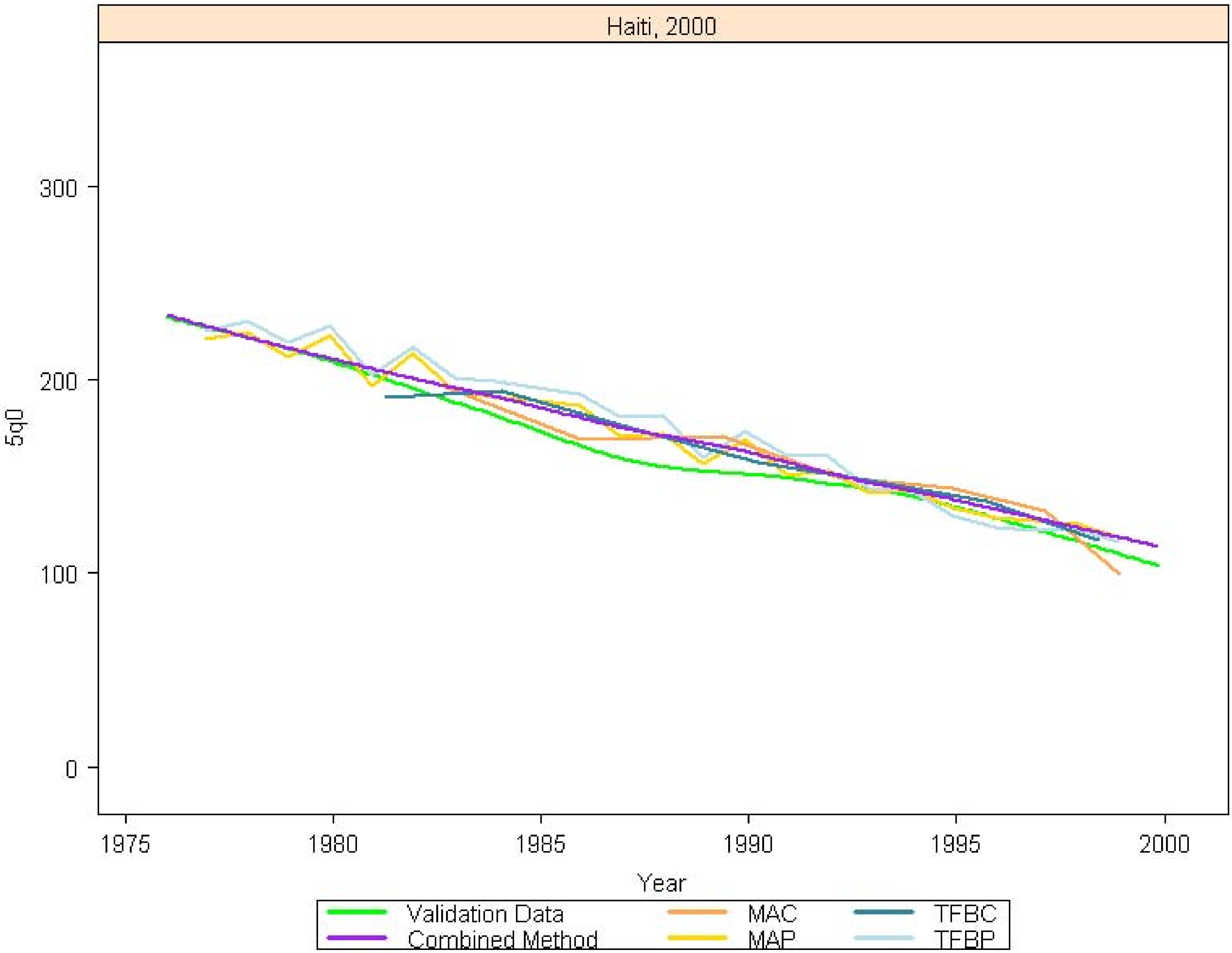

Haiti, 2000
300
200
100
0
5q0
1975
1980
1985
1990
1995
2000
Year
Validation Data
Combined Method
MAC
MAP
TFBC
TFBP

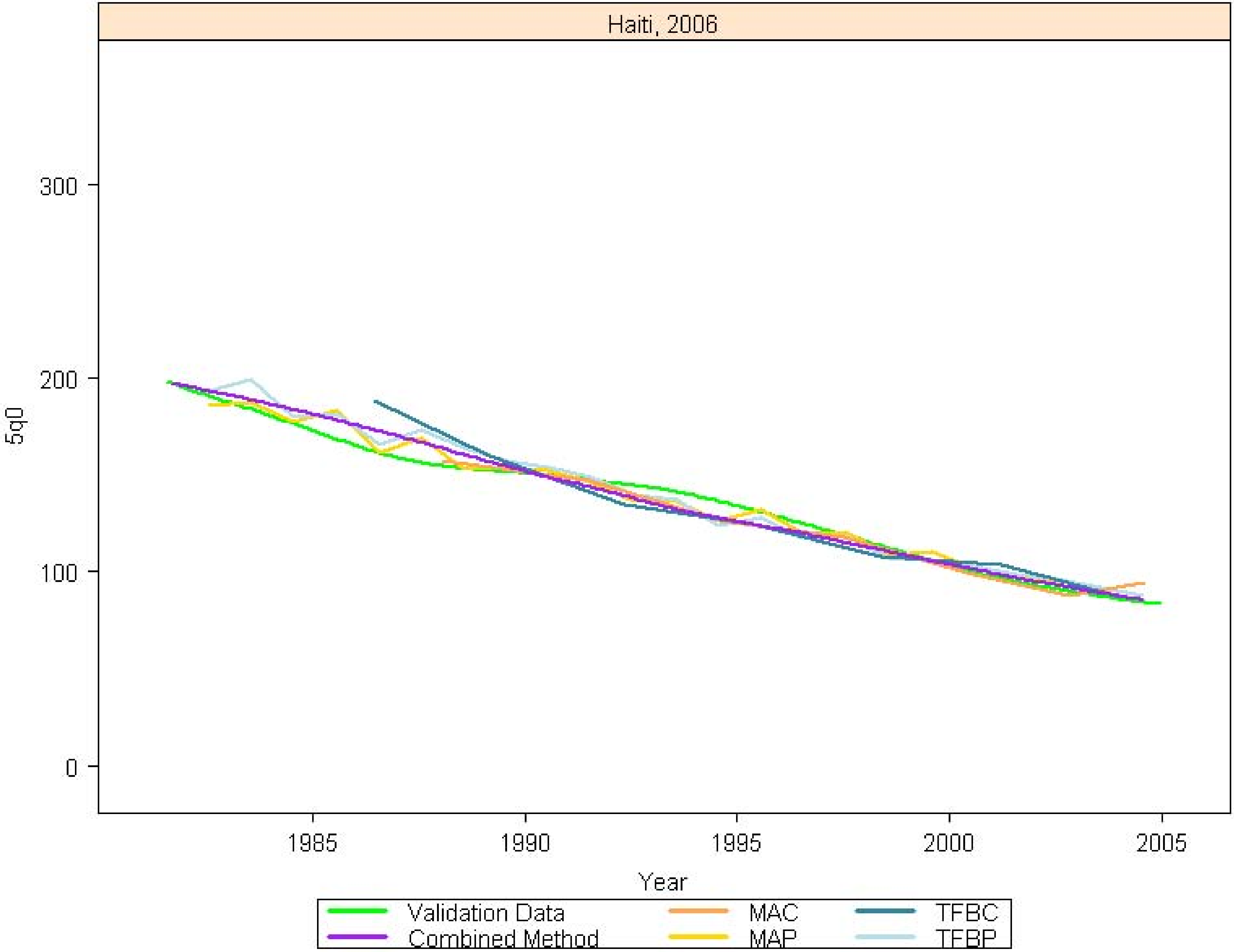

Haiti, 2006
300
200
100
0
5q0
1985
1990
1995
2000
2005
Year
Validation Data
Combined Method
MAC
MAP
TFBC
TFBP

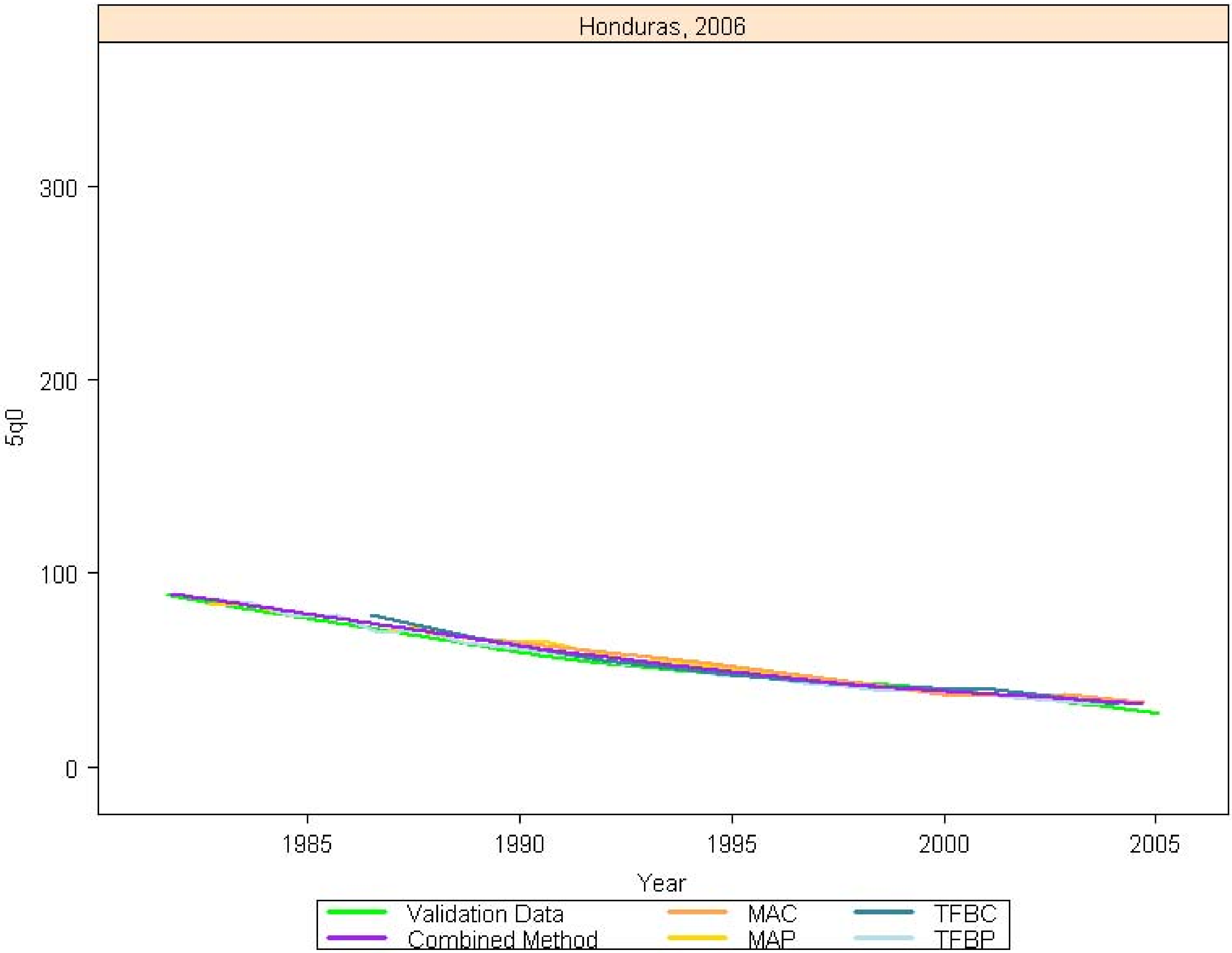

Honduras, 2006
5q0
300
200
100
0
1985
1990
1995
2000
2005
Year
Validation Data
Combined Method
MAC
MAP
TFBC
TFBP

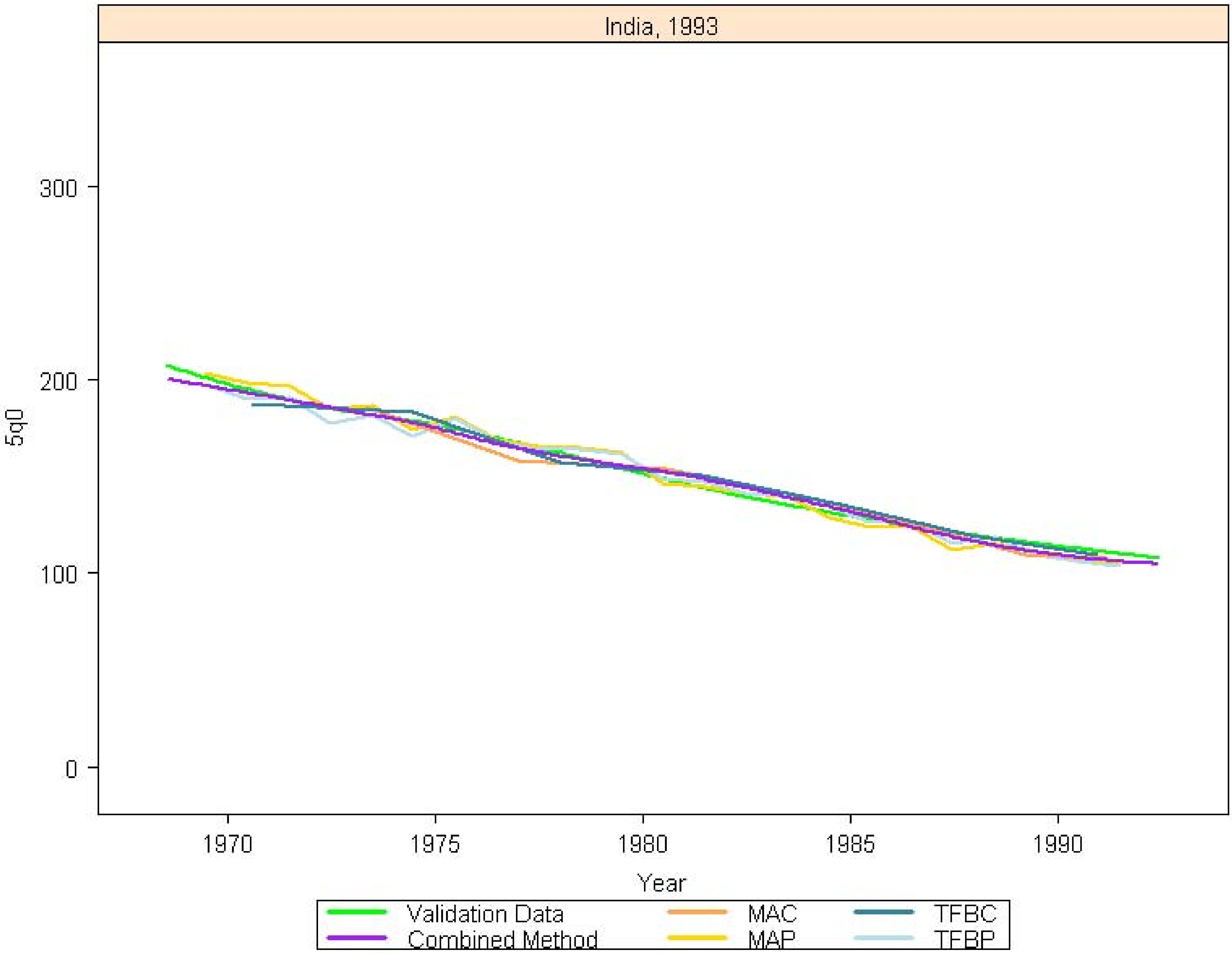
India, 1993
5q0
300
200
100
0
1970
1975
1980
1985
1990
Year
Validation Data
Combined Method
MAC
MAP
TFBC
TFBP

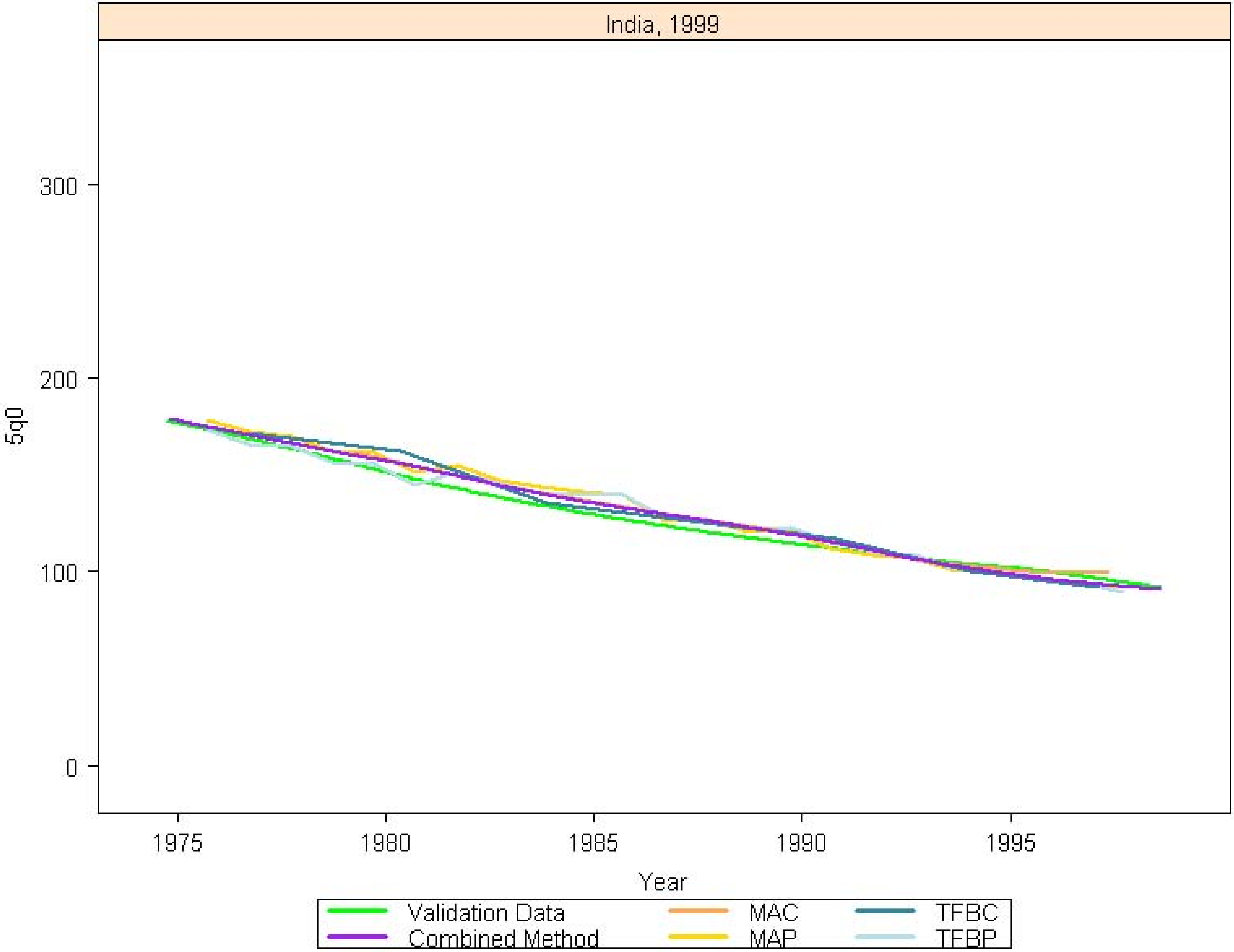

India, 1999
5q0
300
200
100
0
1975
1980
1985
1990
1995
Year
Validation Data
Combined Method
MAC
MAP
TFBC
TFBP

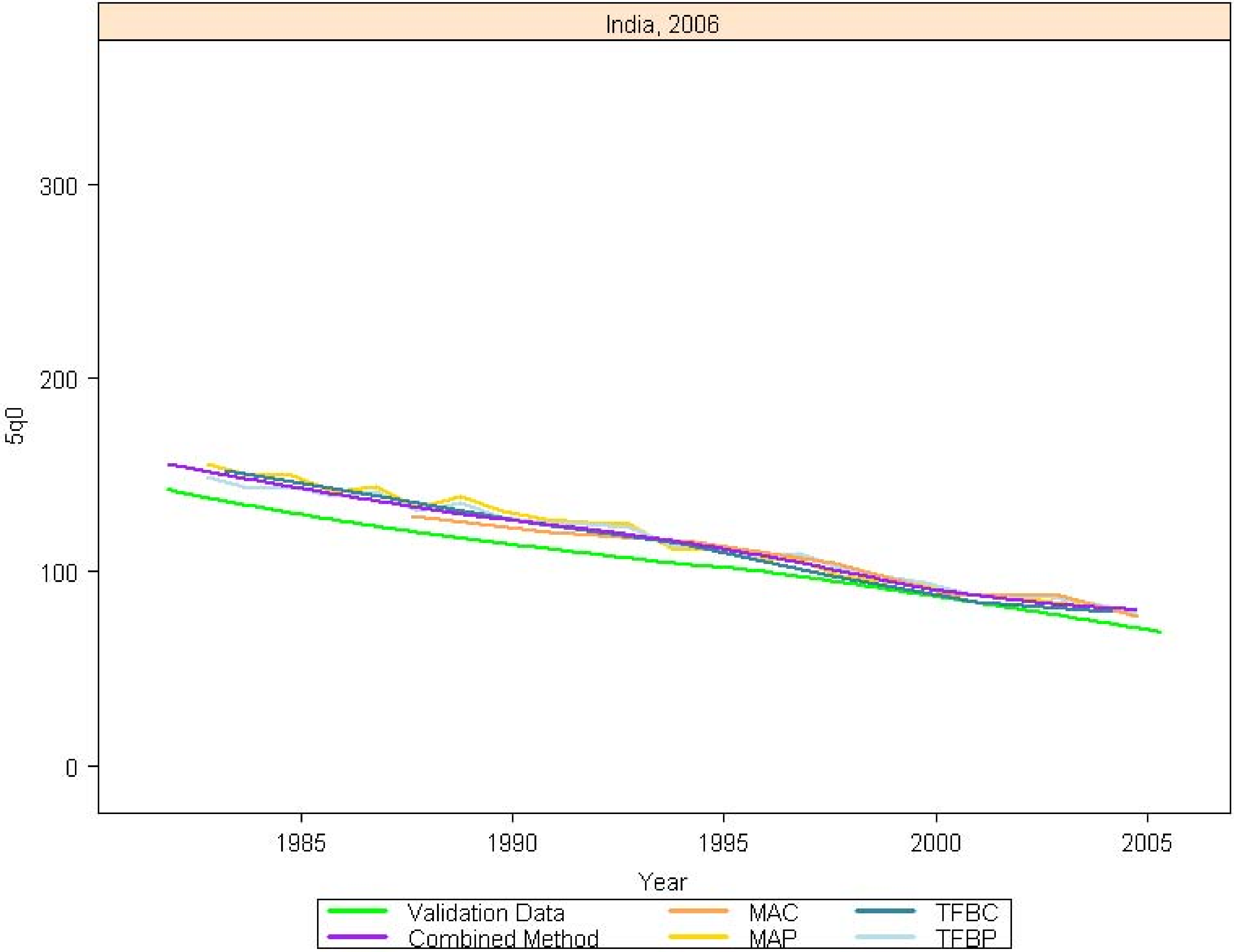

India, 2006
300
200
100
0
5q0
1985
1990
1995
2000
2005
Year
Validation Data
Combined Method
MAC
MAP
TFBC
TFBP

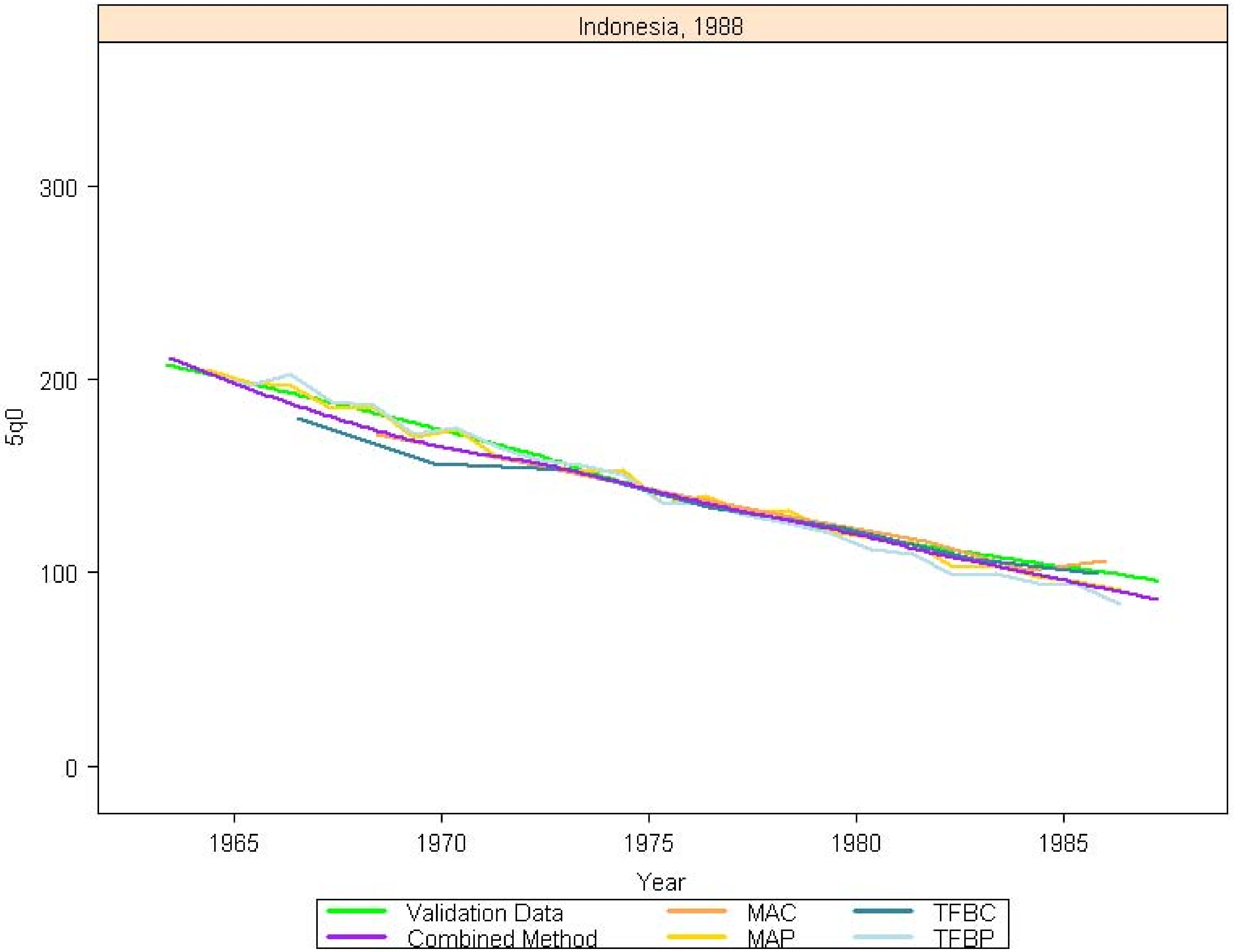
Indonesia, 1988
300
200
100
0
5q0
1965
1970
1975
1980
1985
Year
Validation Data
Combined Method
MAC
MAP
TFBC
TFBP

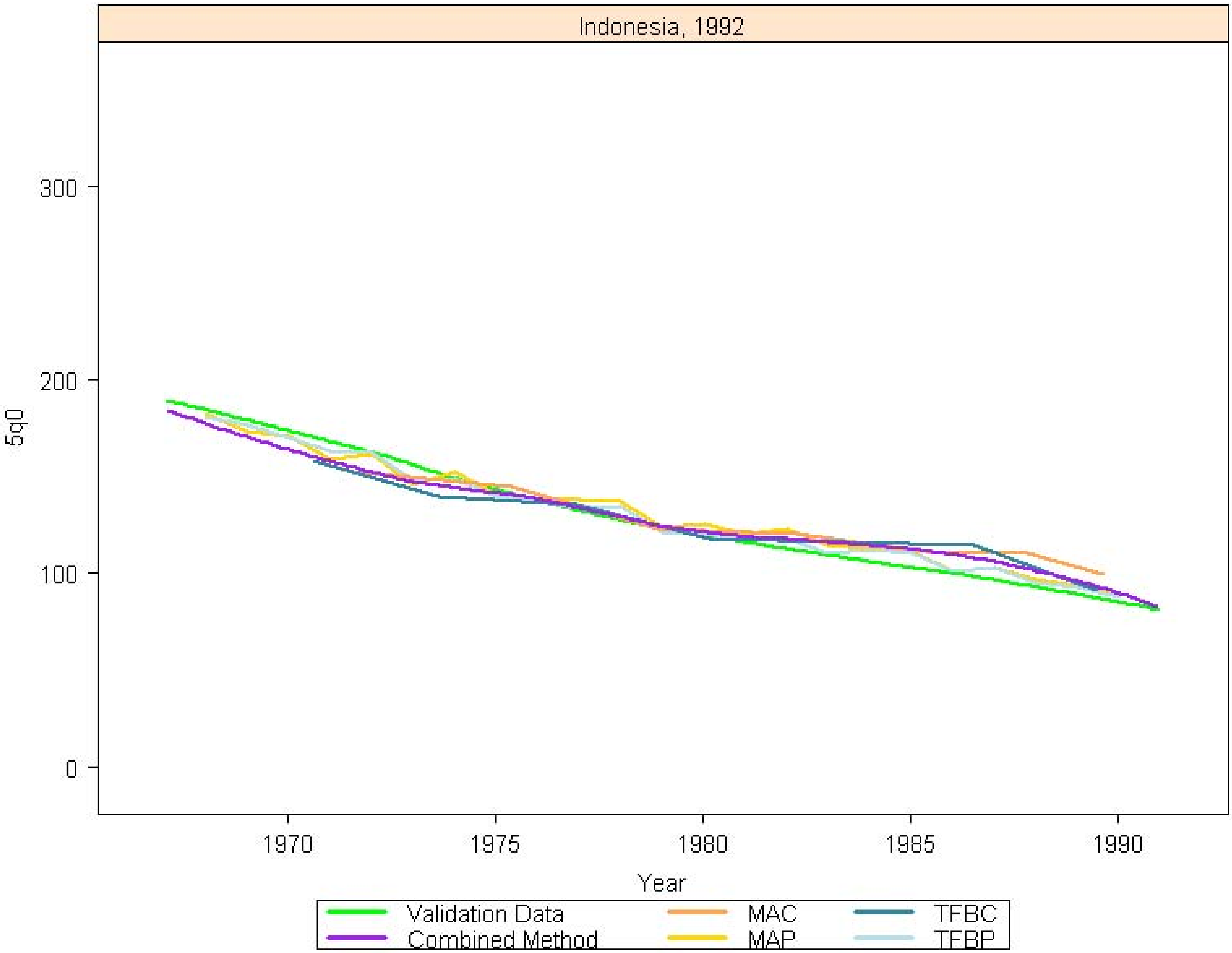
Indonesia, 1992
5q0
300
200
100
0
1970
1975
1980
1985
1990
Year
Validation Data
Combined Method
MAC
MAP
TFBC
TFBP

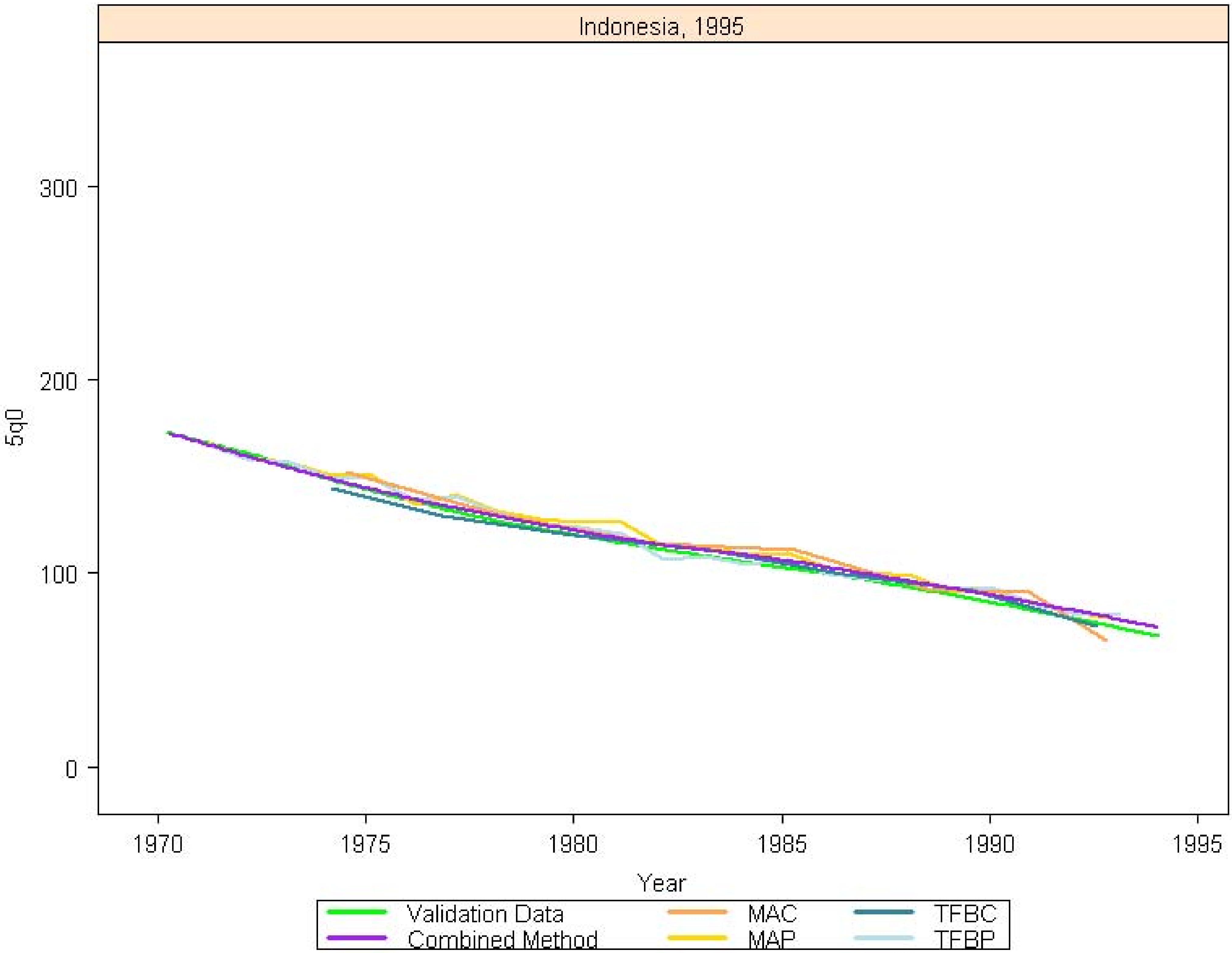

Indonesia, 1995
5q0
300
200
100
0
1970
1975
1980
1985
1990
1995
Year
Validation Data
Combined Method
MAC
MAP
TFBC
TFBP

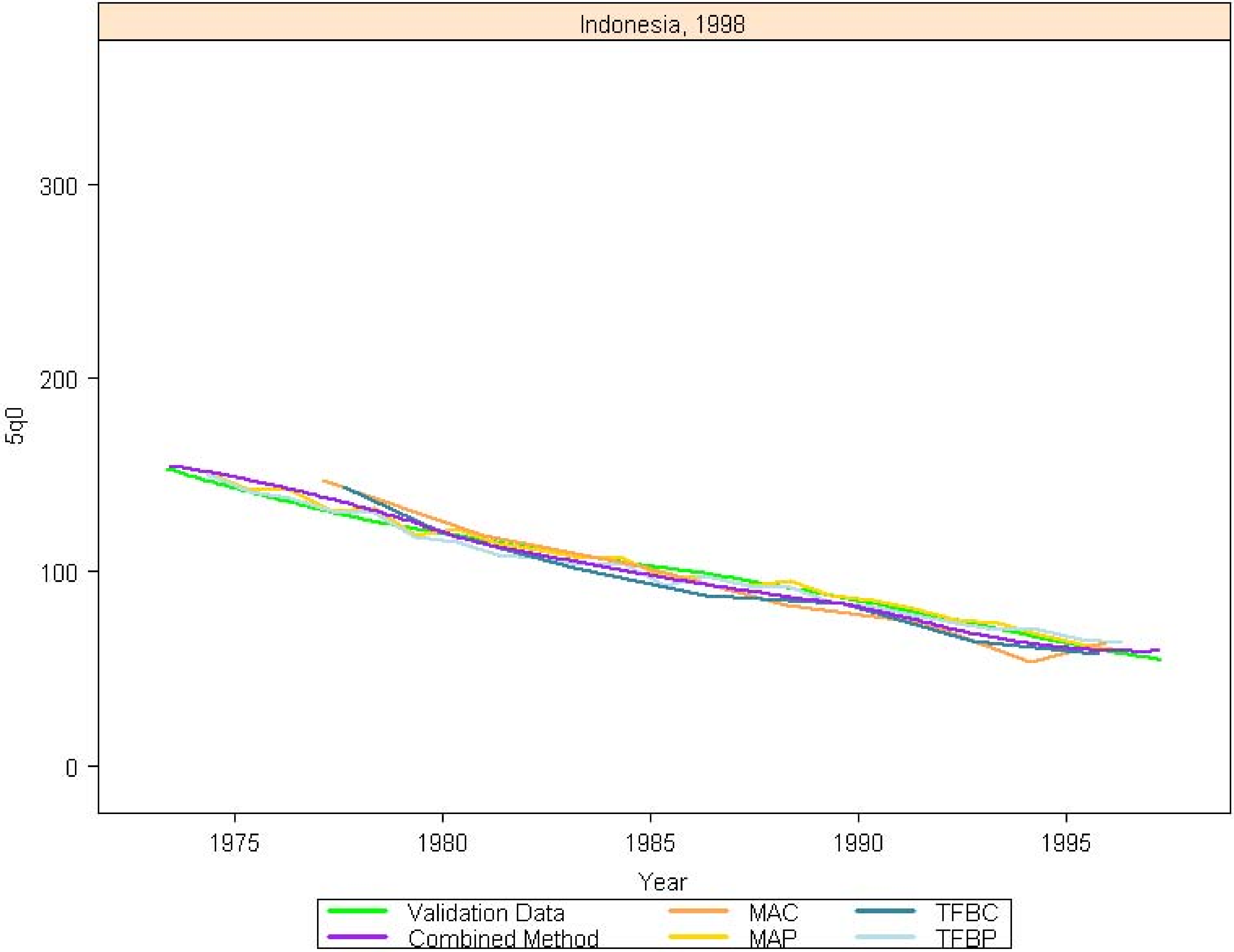

Indonesia, 1998
300
200
100
0
5q0
1975
1980
1985
1990
1995
Year
Validation Data
Combined Method
MAC
MAP
TFBC
TFBP

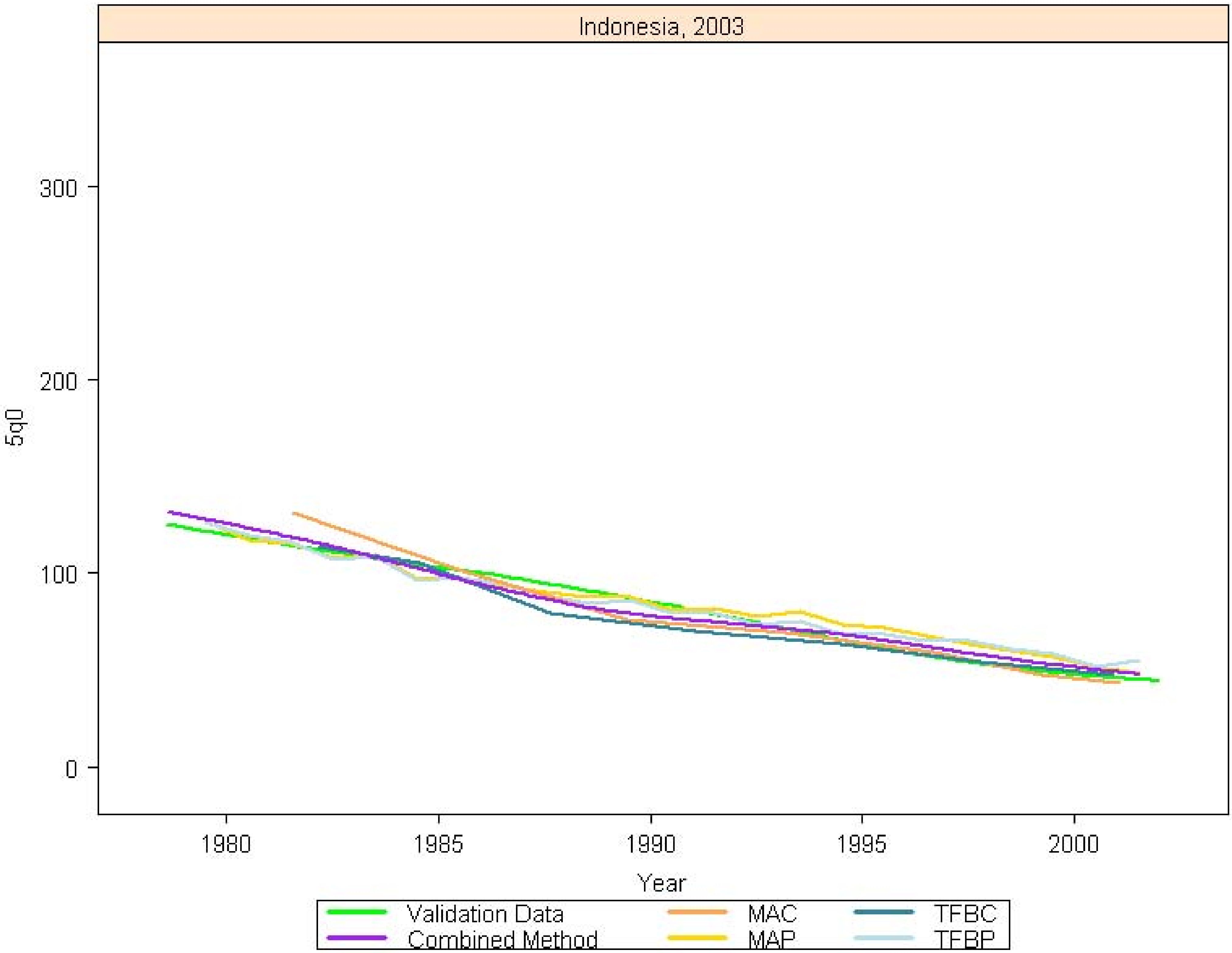
Indonesia, 2003
300
200
100
0
5q0
1980
1985
1990
1995
2000
Year
Validation Data
Combined Method
MAC
MAP
TFBC
TFBP

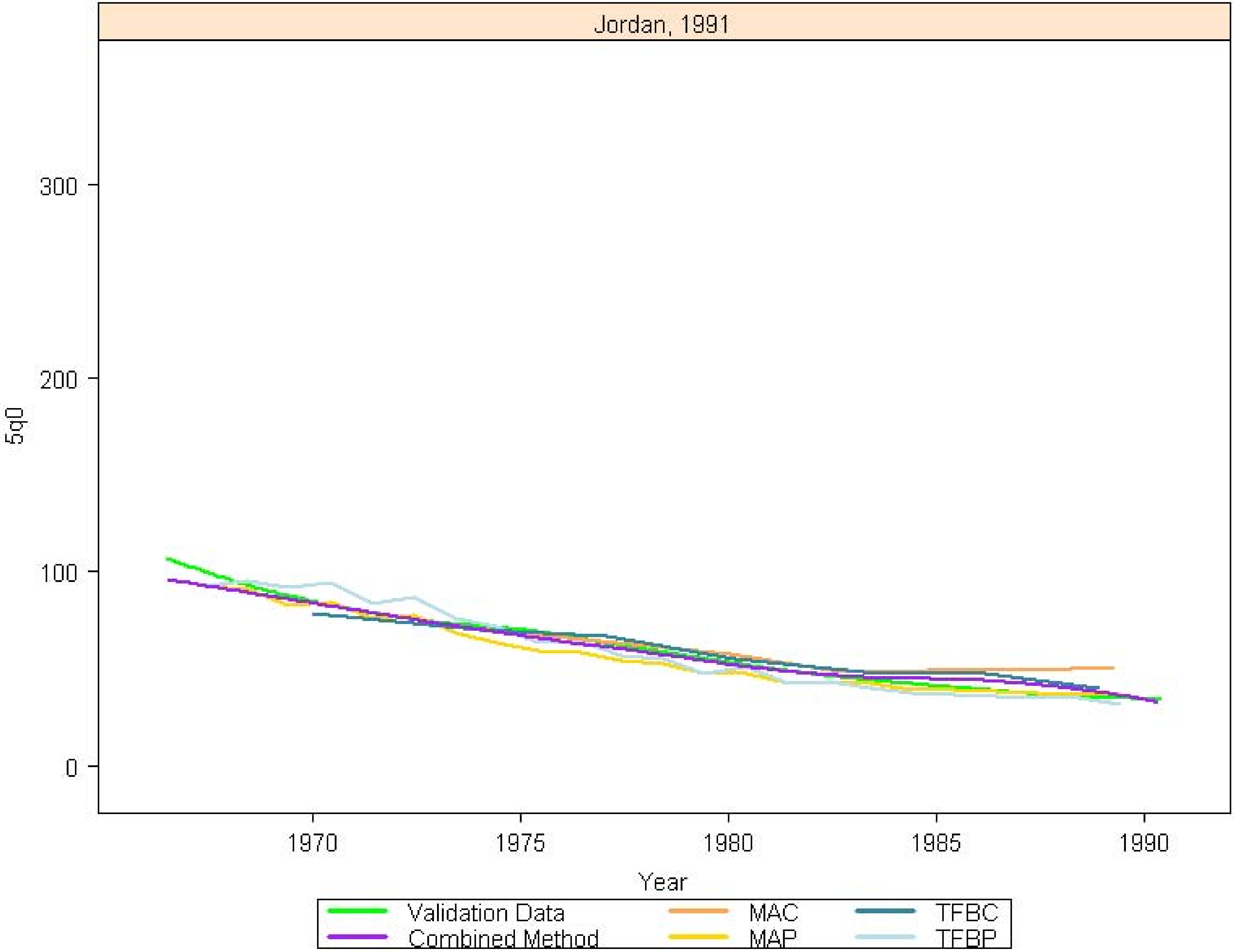

Jordan, 1991
300
200
100
0
5q0
1970
1975
1980
1985
1990
Year
Validation Data
Combined Method
MAC
MAP
TFBC
TFBP

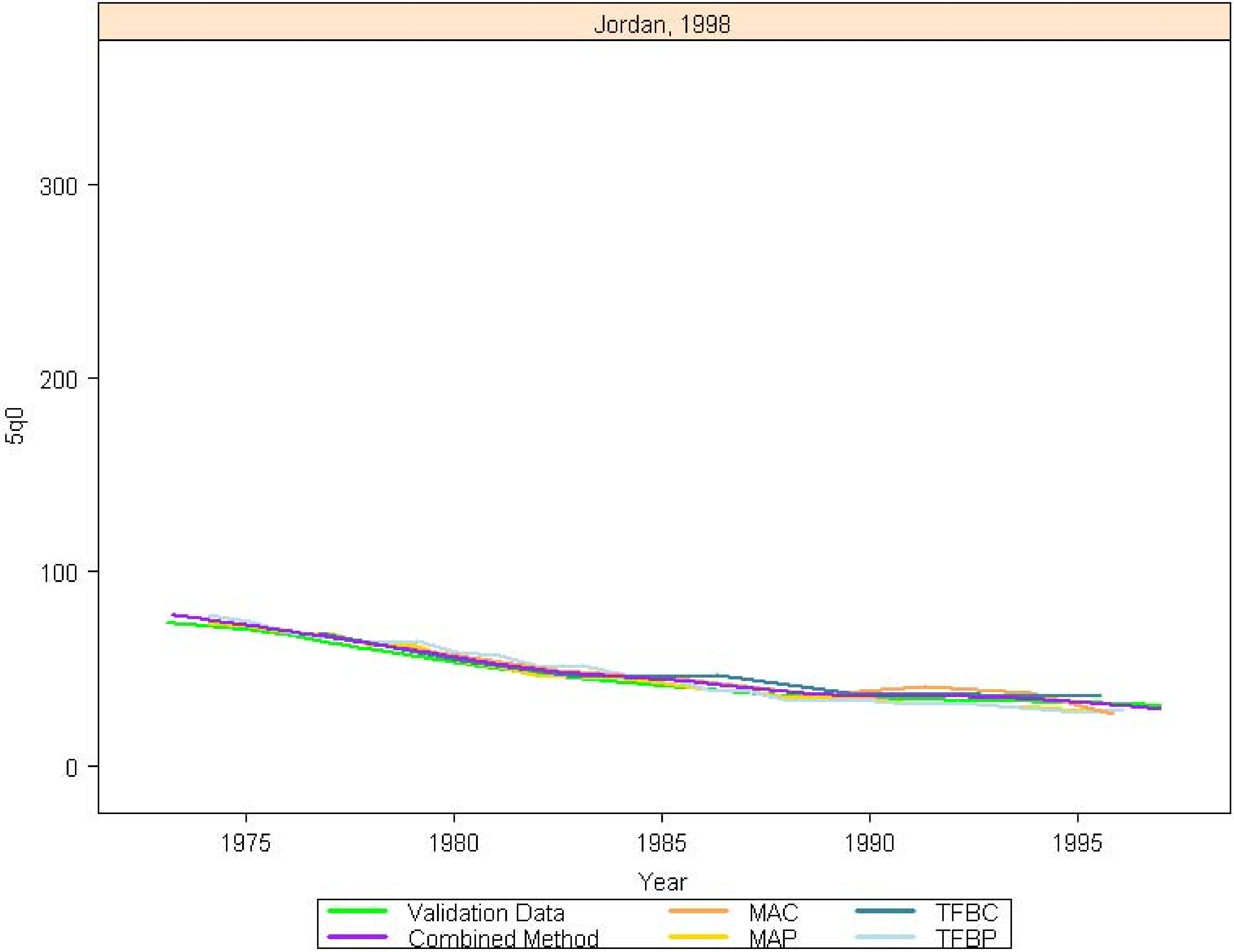
Jordan, 1998
300
200
100
0
5q0
1975
1980
1985
1990
1995
Year
Validation Data
Combined Method
MAC
MAP
TFBC
TFBP

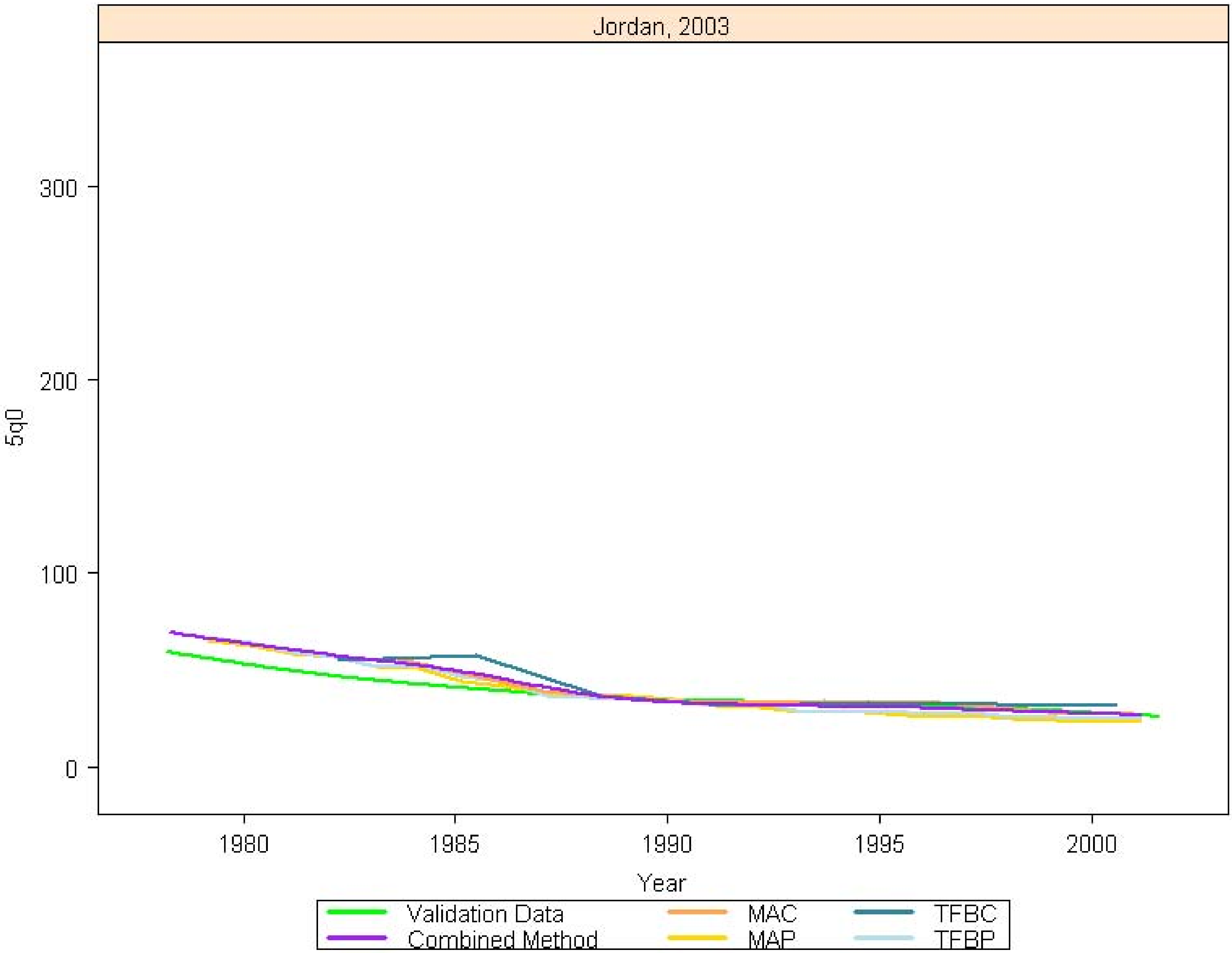
Jordan, 2003
300
200
100
0
5q0
1980
1985
1990
1995
2000
Year
Validation Data
Combined Method
MAC
MAP
TFBC
TFBP

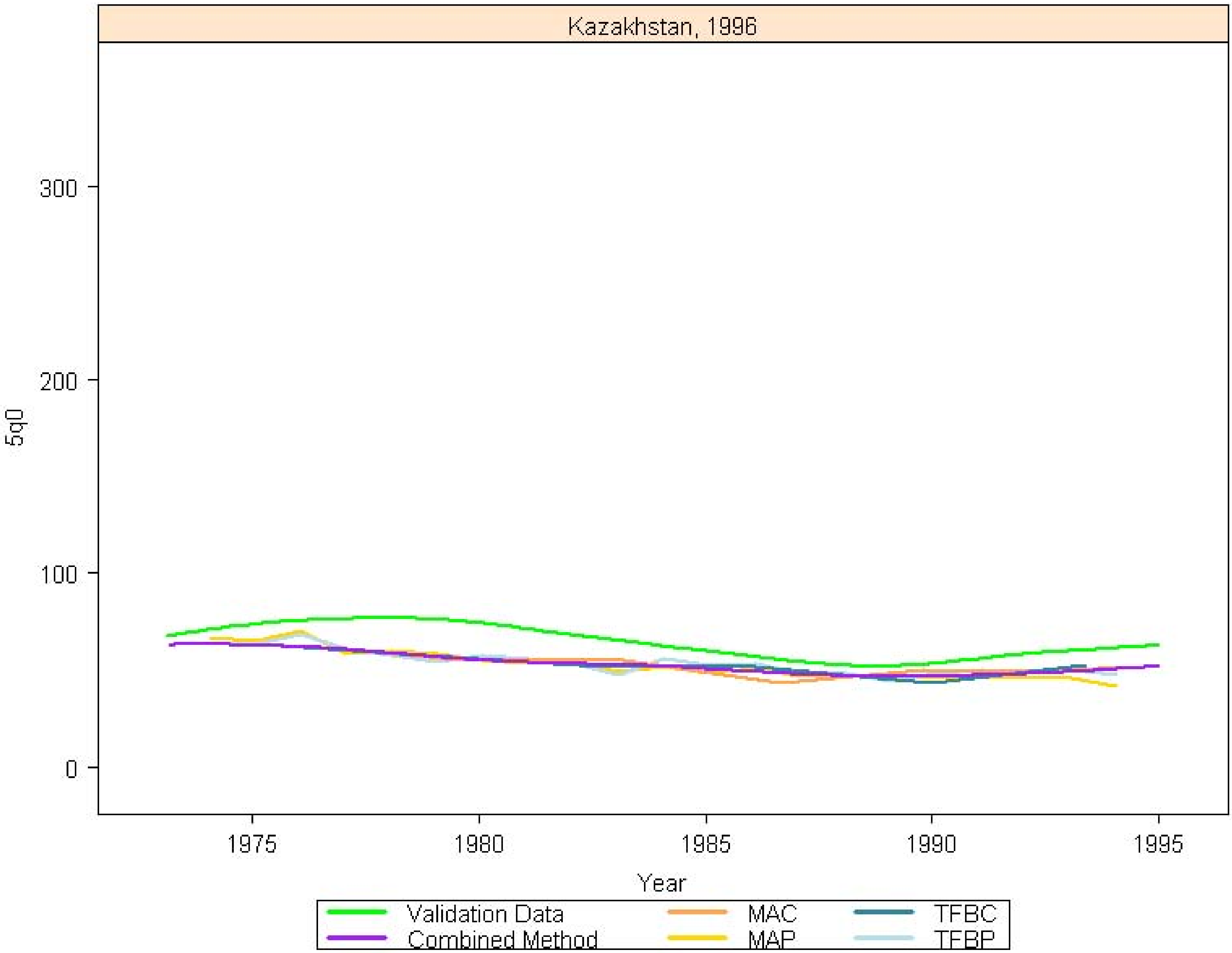
Kazakhstan, 1996
5q0
300
200
100
0
1975
1980
1985
1990
1995
Year
Validation Data
Combined Method
MAC
MAP
TFBC
TFBP

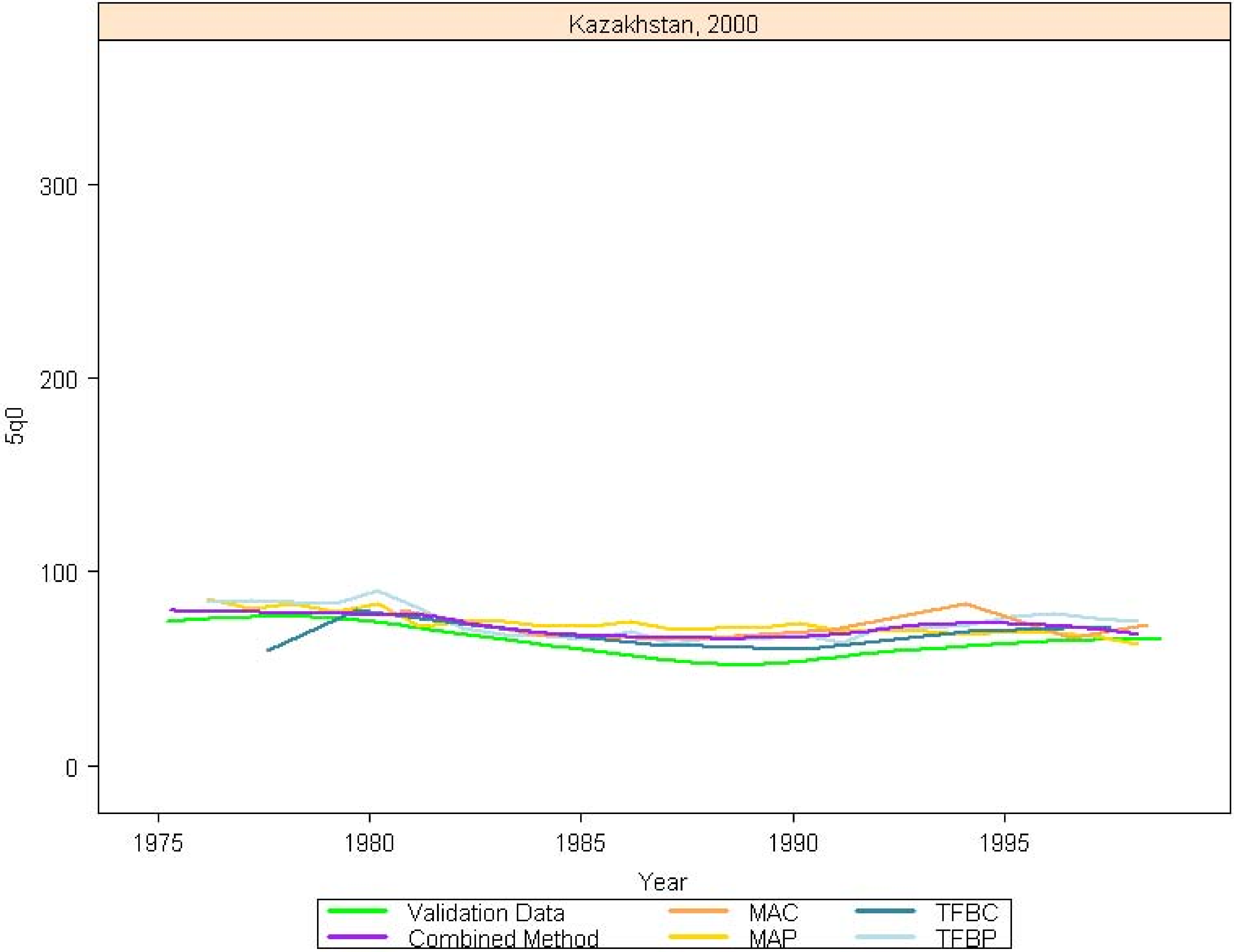
Kazakhstan, 2000
300
200
100
0
5q0
1975
1980
1985
1990
1995
Year
Validation Data
Combined Method
MAC
MAP
TFBC
TFBP

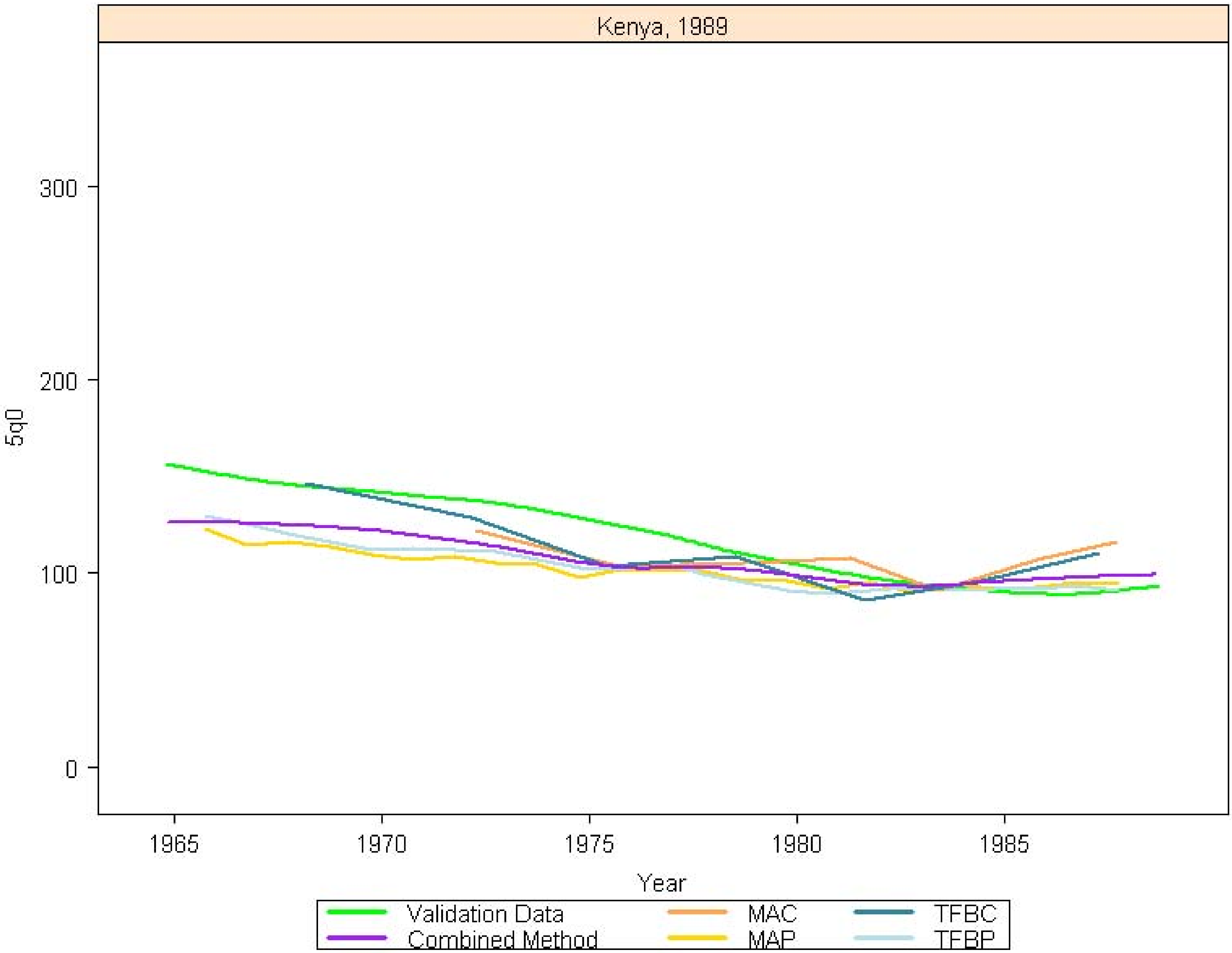
Kenya, 1989
5q0
300
200
100
0
1965
1970
1975
1980
1985
Year
Validation Data
Combined Method
MAC
MAP
TFBC
TFBP

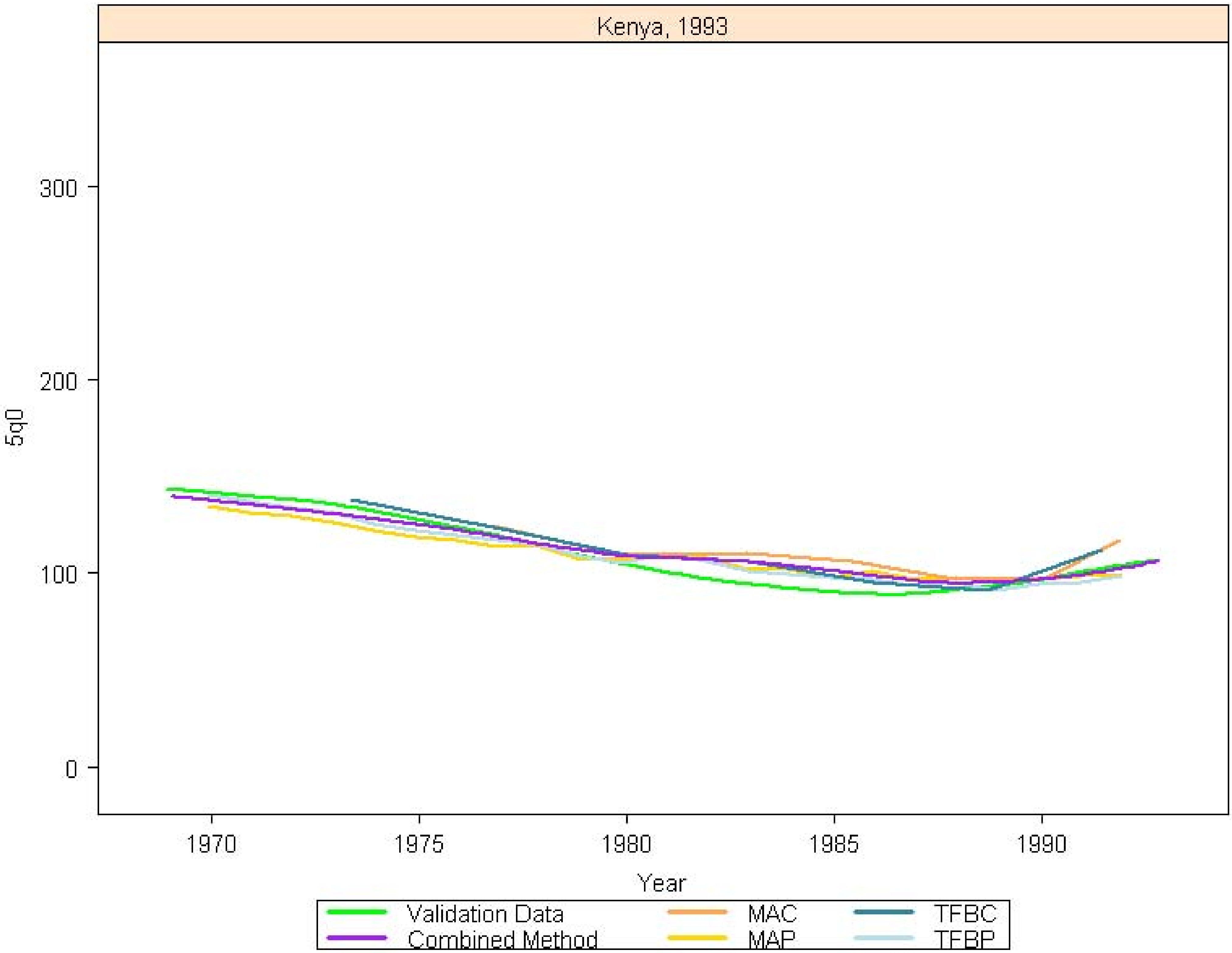
Kenya, 1993
5q0
300
200
100
0
1970
1975
1980
1985
1990
Year
Validation Data
Combined Method
MAC
MAP
TFBC
TFBP

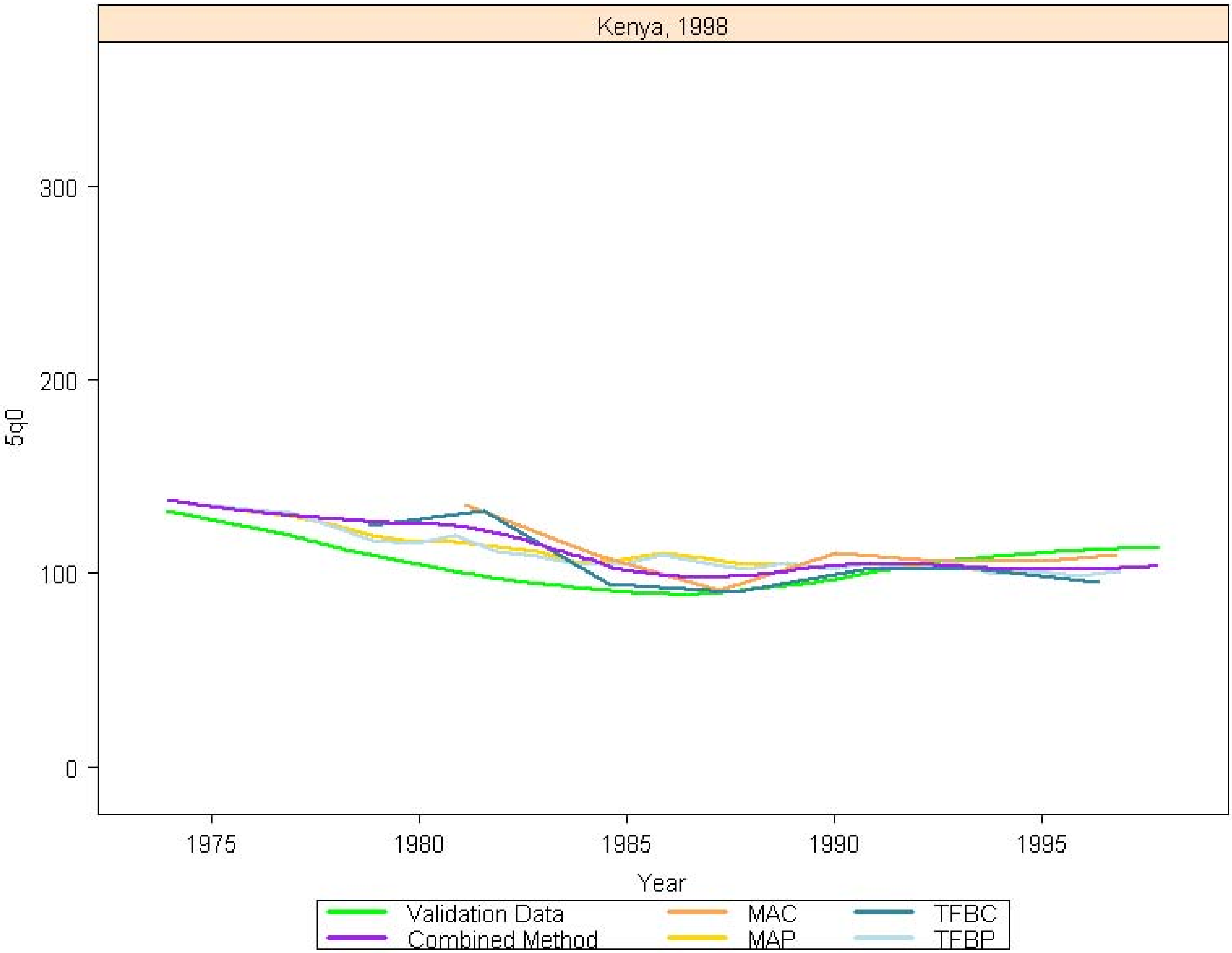

Kenya, 1998
300
200
100
0
5q0
1975
1980
1985
1990
1995
Year
Validation Data
Combined Method
MAC
MAP
TFBC
TFBP

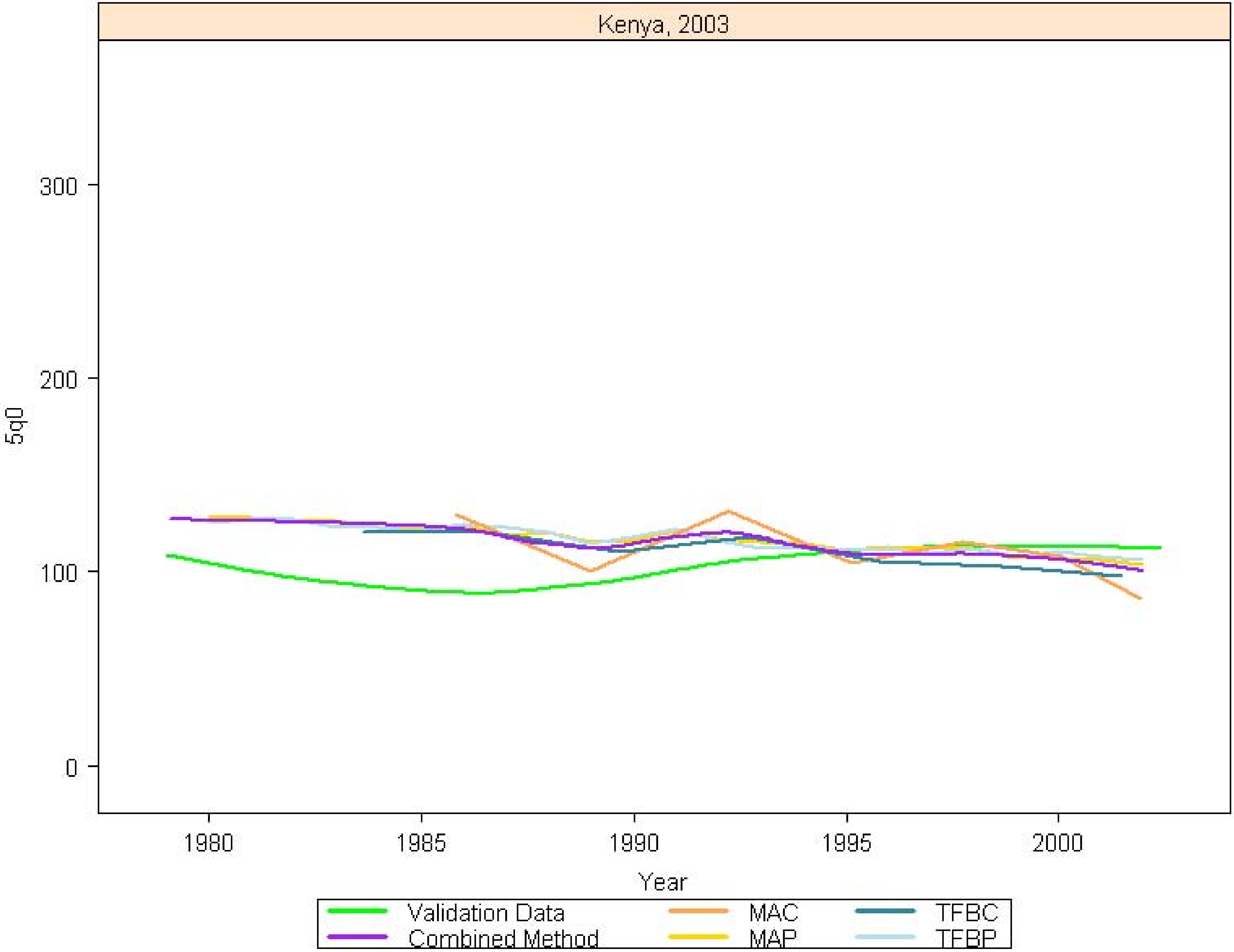
Kenya, 2003
5q0
300
200
100
0
1980
1985
1990
1995
2000
Year
Validation Data
Combined Method
MAC
MAP
TFBC
TFBP

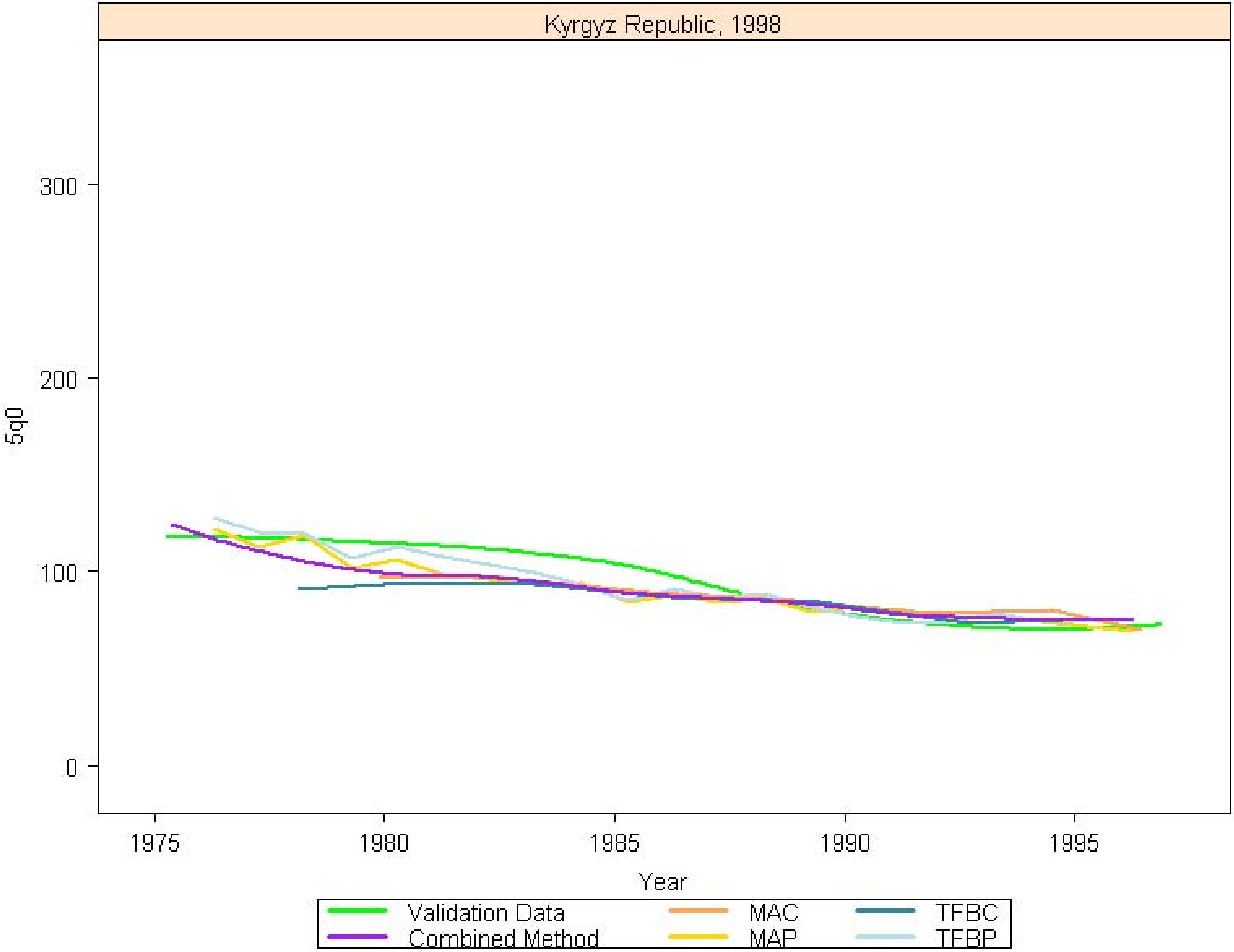
Kyrgyz Republic, 1998
300
200
100
0
5q0
1975
1980
1985
1990
1995
Year
Validation Data
Combined Method
MAC
MAP
TFBC
TFBP

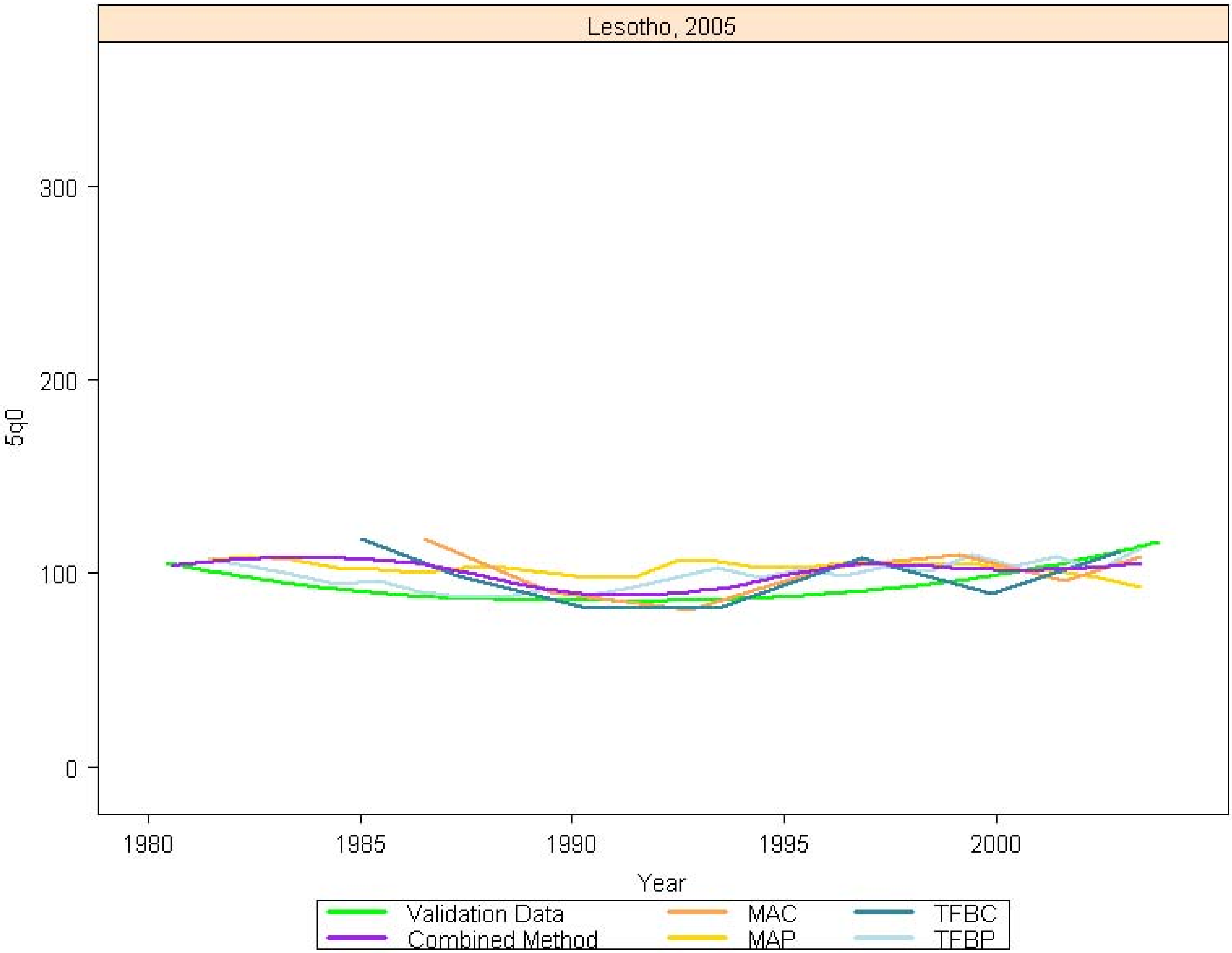

Lesotho, 2005
300
200
100
0
5q0
1980
1985
1990
1995
2000
Year
Validation Data
Combined Method
MAC
MAP
TFBC
TFBP

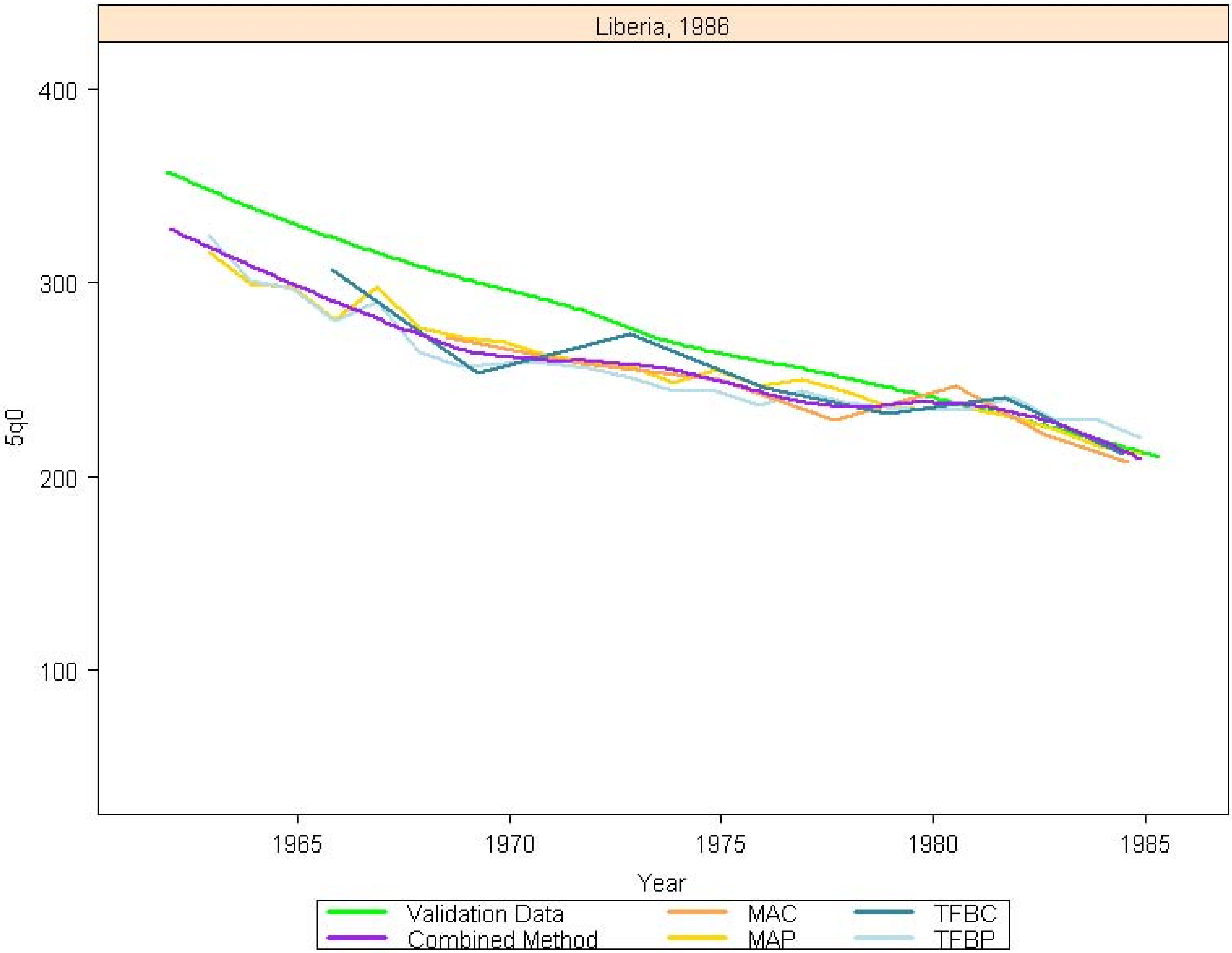

Liberia, 1986
400
300
200
100
5q0
1965
1970
1975
1980
1985
Year
Validation Data
Combined Method
MAC
MAP
TFBC
TFBP

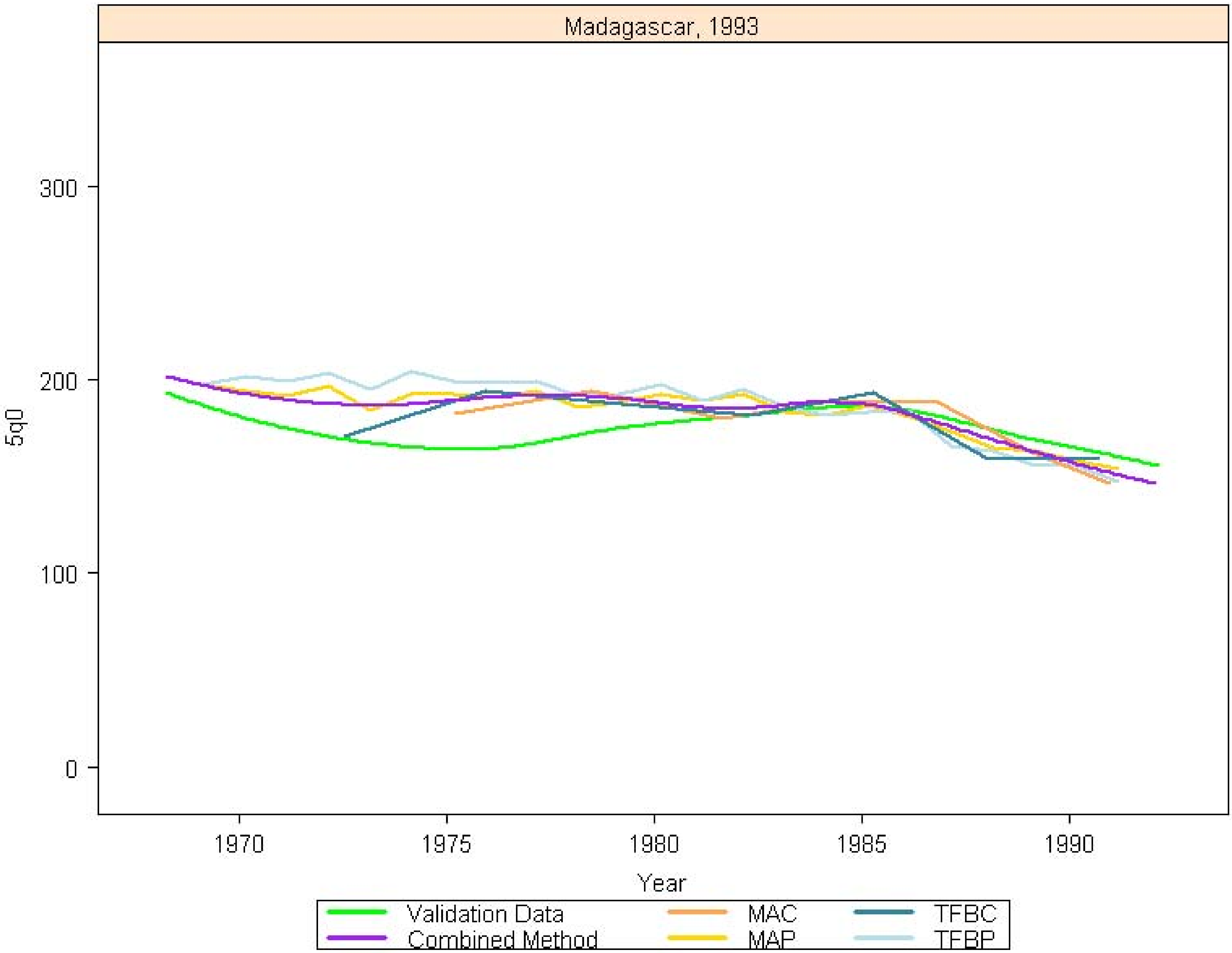
Madagascar, 1993
300
200
100
0
5q0
1970
1975
1980
1985
1990
Year
Validation Data
Combined Method
MAC
MAP
TFBC
TFBP

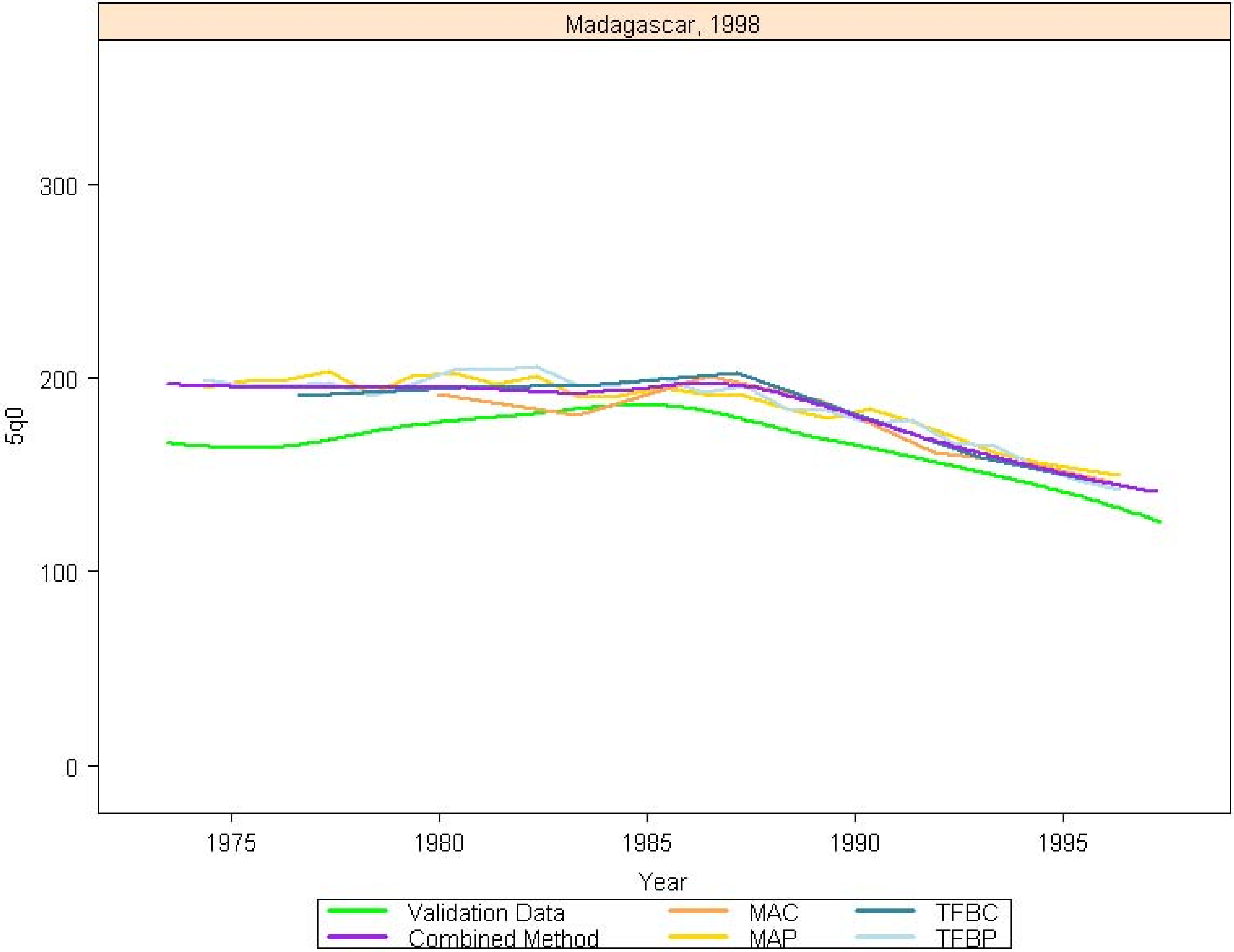
Madagascar, 1998
300
200
100
0
5q0
1975
1980
1985
1990
1995
Year
Validation Data
Combined Method
MAC
MAP
TFBC
TFBP

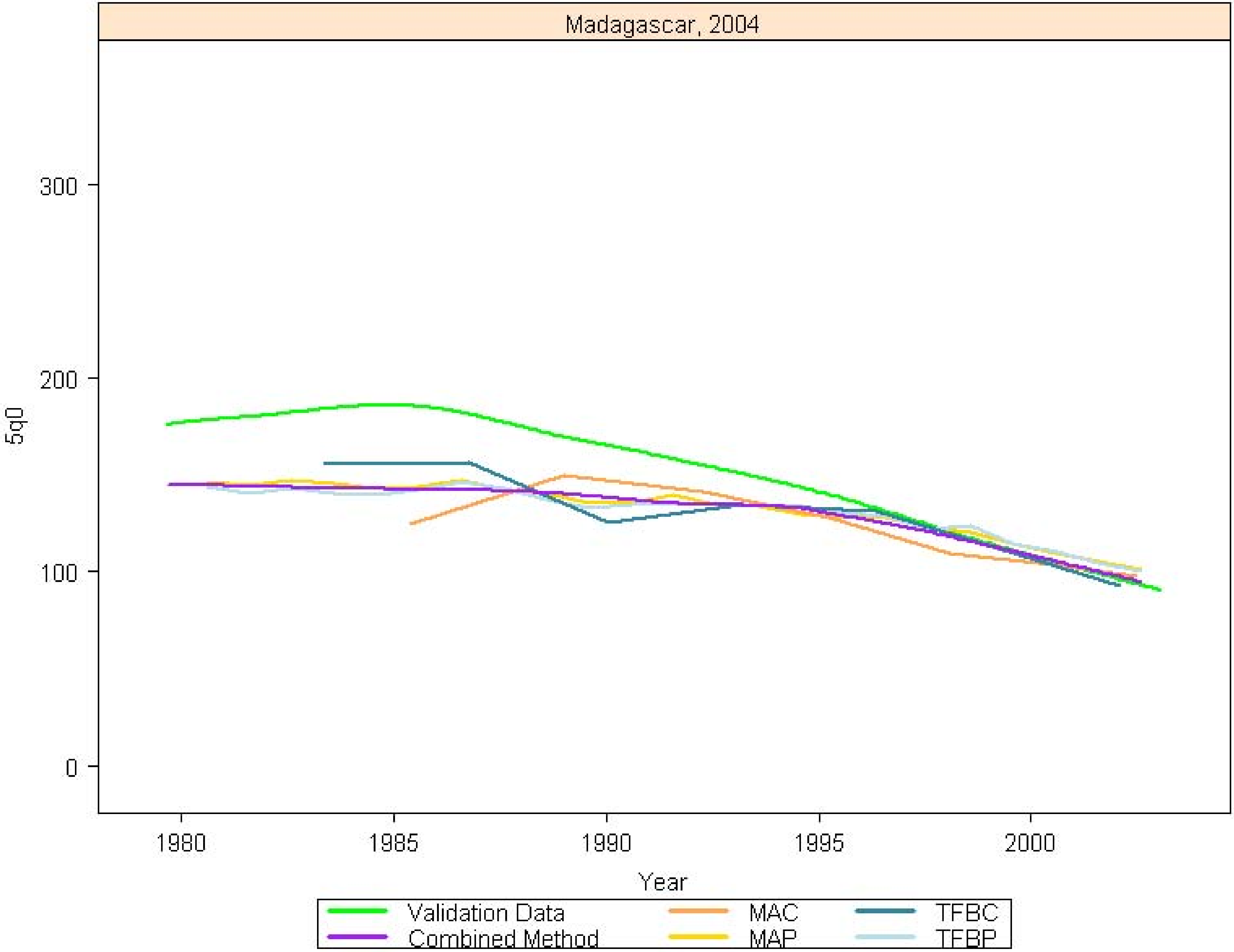

Madagascar, 2004
300
200
100
0
5q0
1980
1985
1990
1995
2000
Year
Validation Data
Combined Method
MAC
MAP
TFBC
TFBP

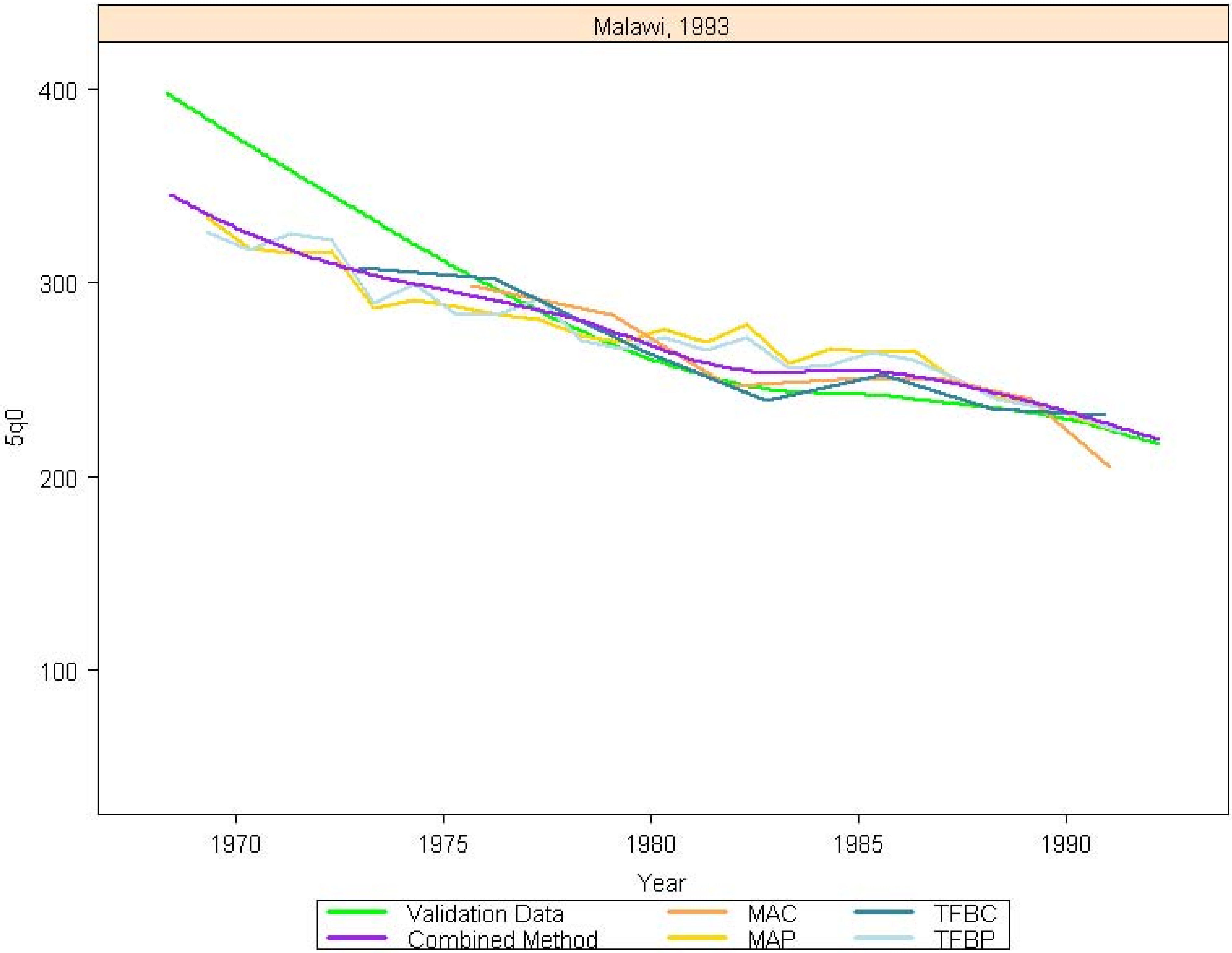
Malawi, 1993
400
300
200
100
5q0
1970
1975
1980
1985
1990
Year
Validation Data
Combined Method
MAC
MAP
TFBC
TFBP

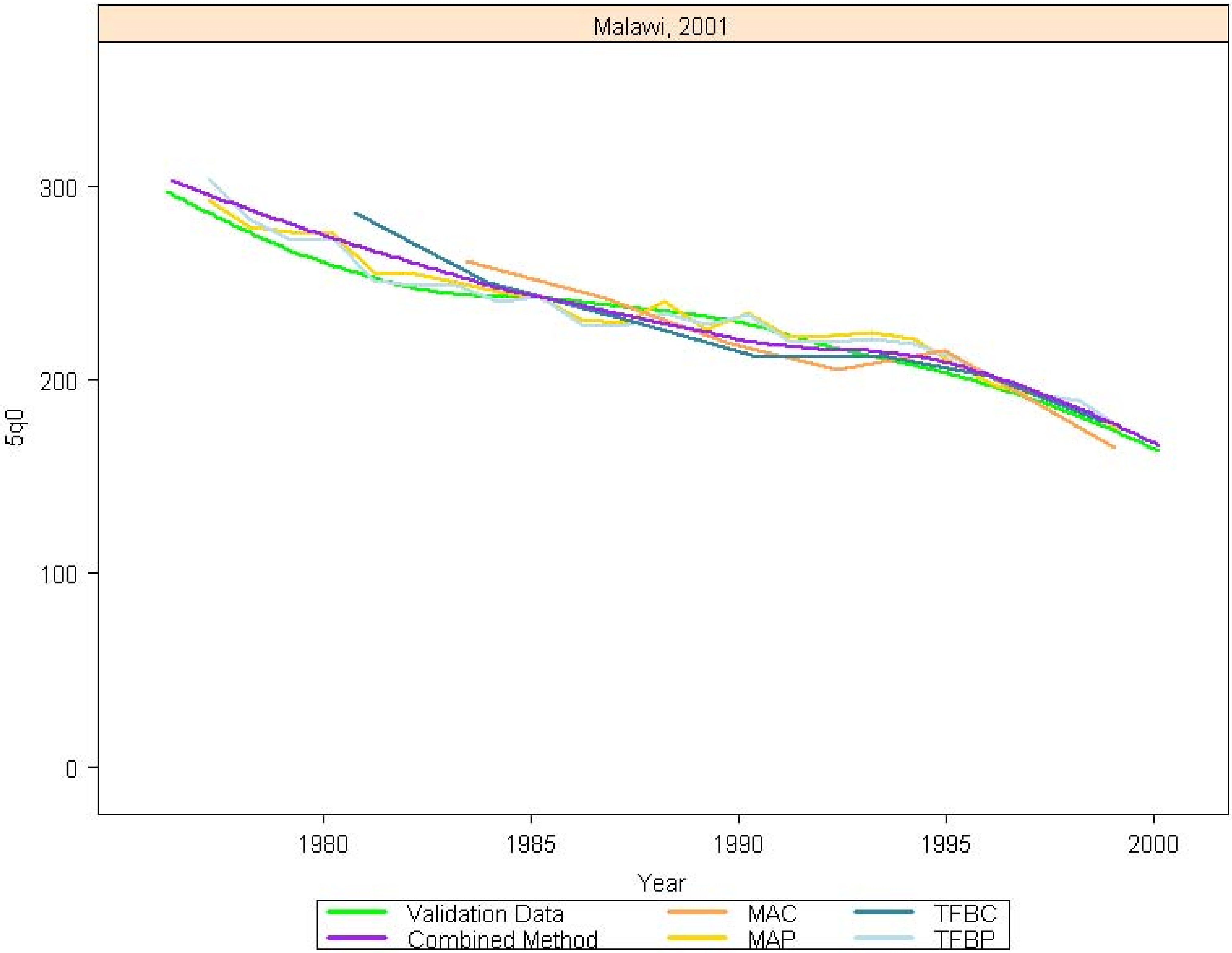
Malawi, 2001
300
200
100
0
5q0
1980
1985
1990
1995
2000
Year
Validation Data
Combined Method
MAC
MAP
TFBC
TFBP

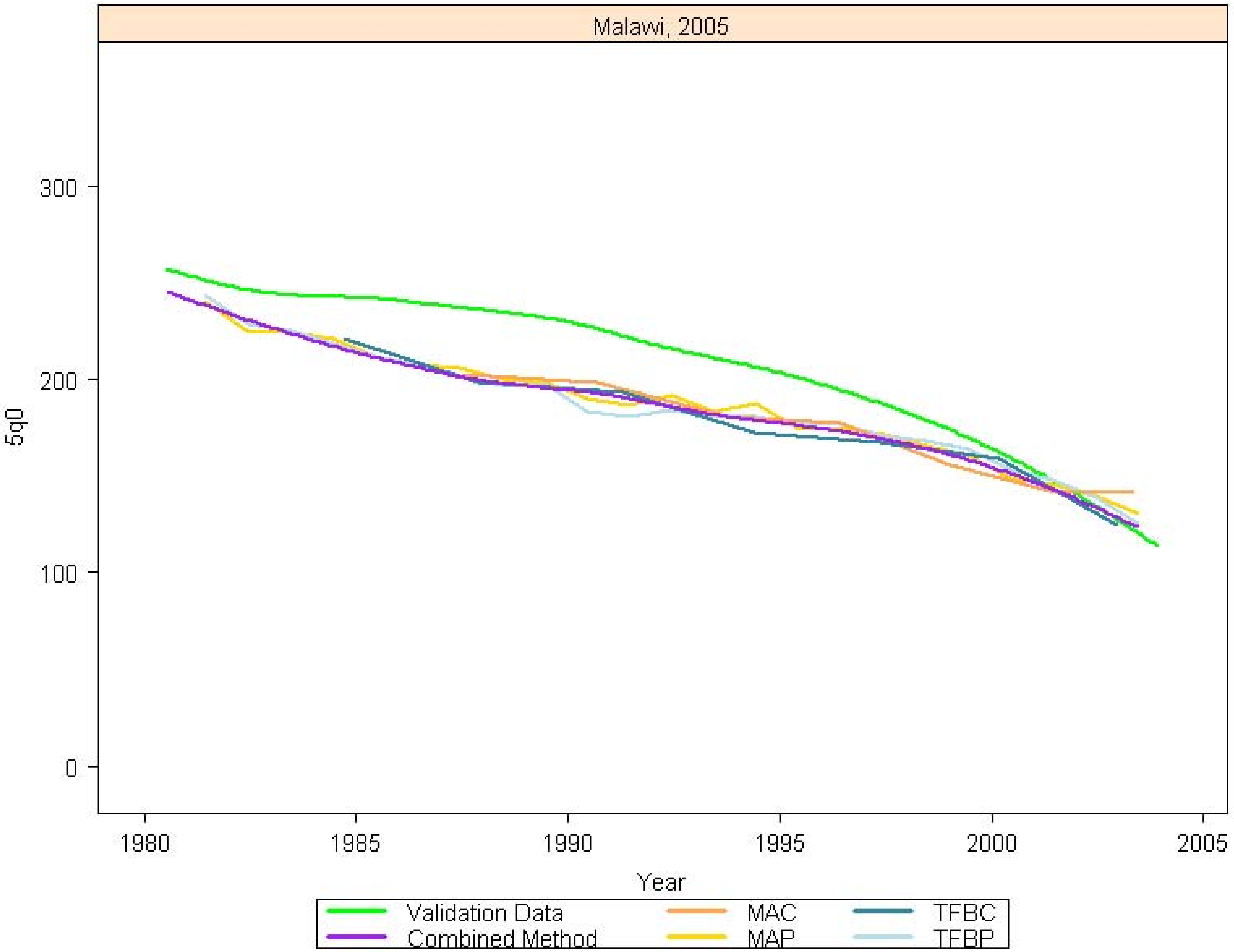

Malawi, 2005
300
200
100
0
5q0
1980
1985
1990
1995
2000
2005
Year
Validation Data
Combined Method
MAC
MAP
TFBC
TFBP

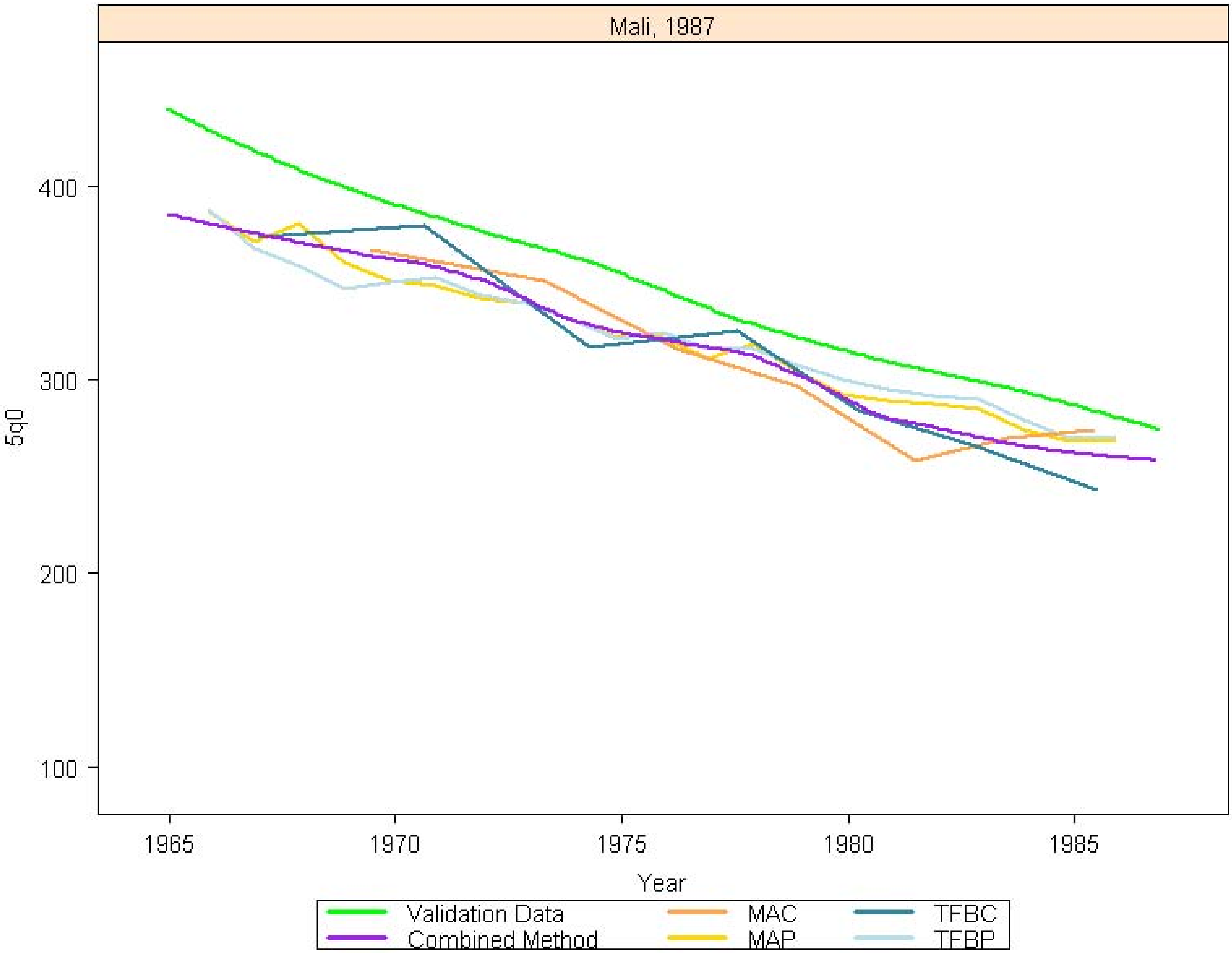
Mali, 1987
400
300
200
100
5q0
1965
1970
1975
1980
1985
Year
Validation Data
Combined Method
MAC
MAP
TFBC
TFBP

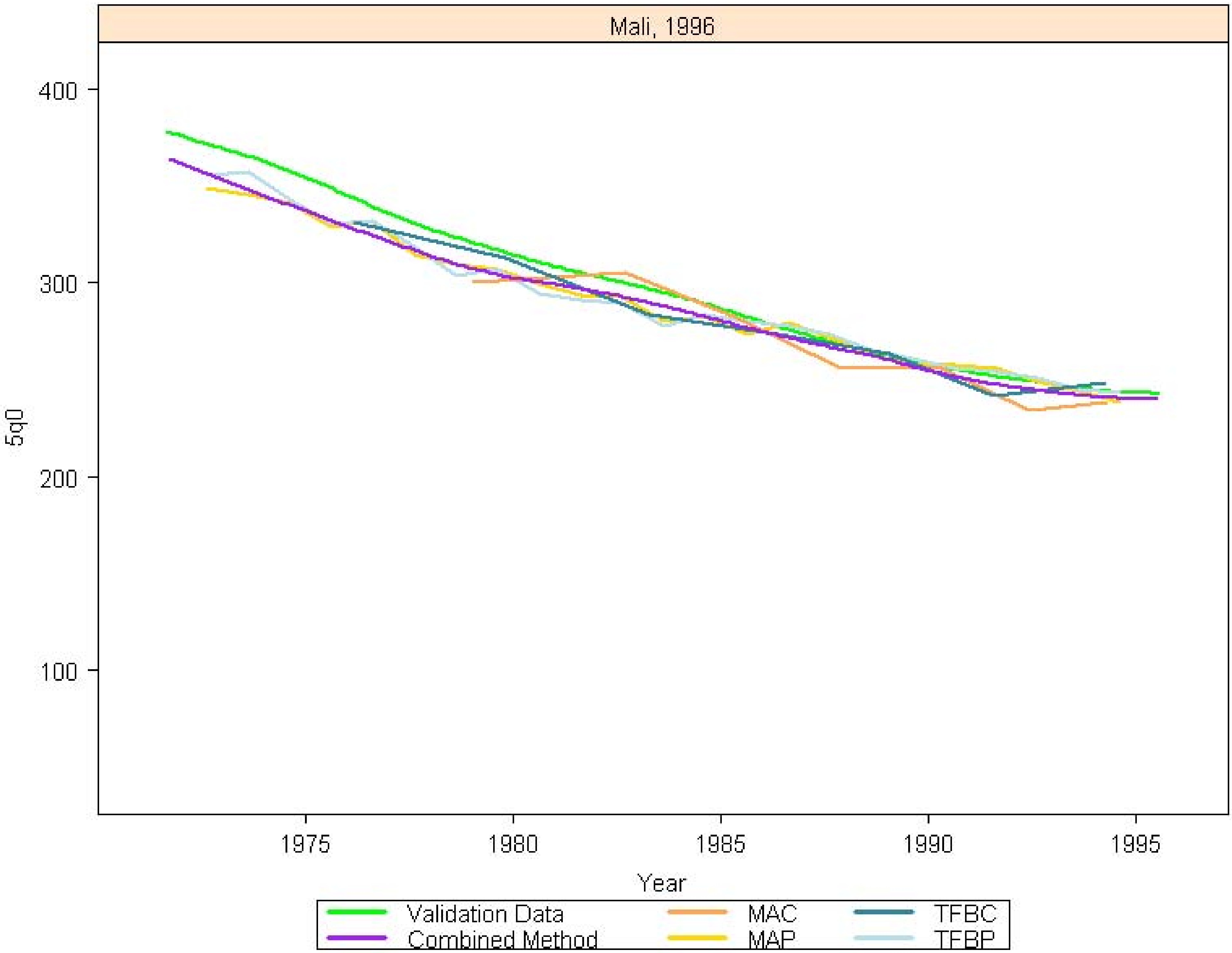

Mali, 1996
400
300
200
100
5q0
1975
1980
1985
1990
1995
Year
Validation Data
Combined Method
MAC
MAP
TFBC
TFBP

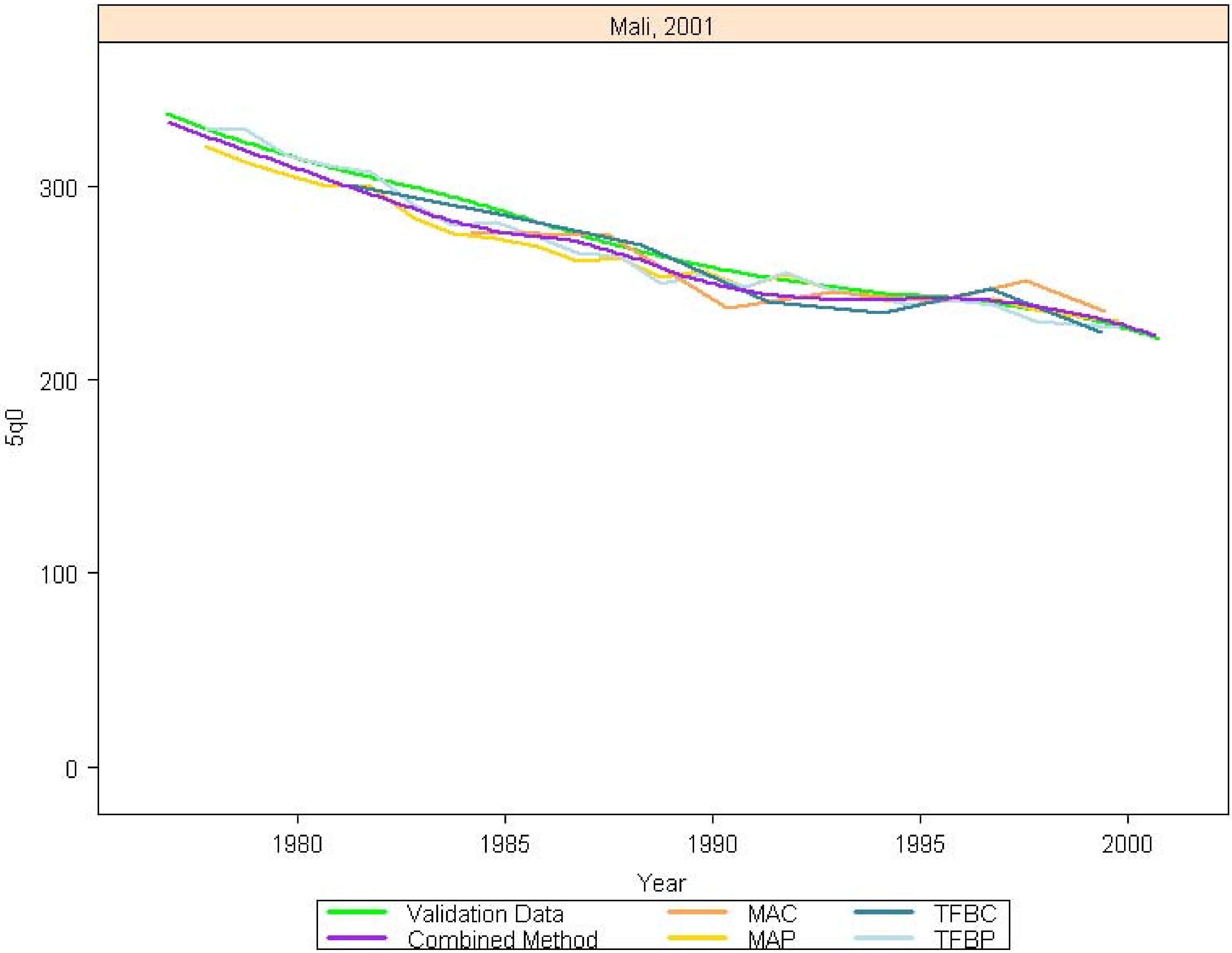
Mali, 2001
5q0
0
100
200
300
1980
1985
1990
1995
2000
Year
Validation Data
Combined Method
MAC
MAP
TFBC
TFBP

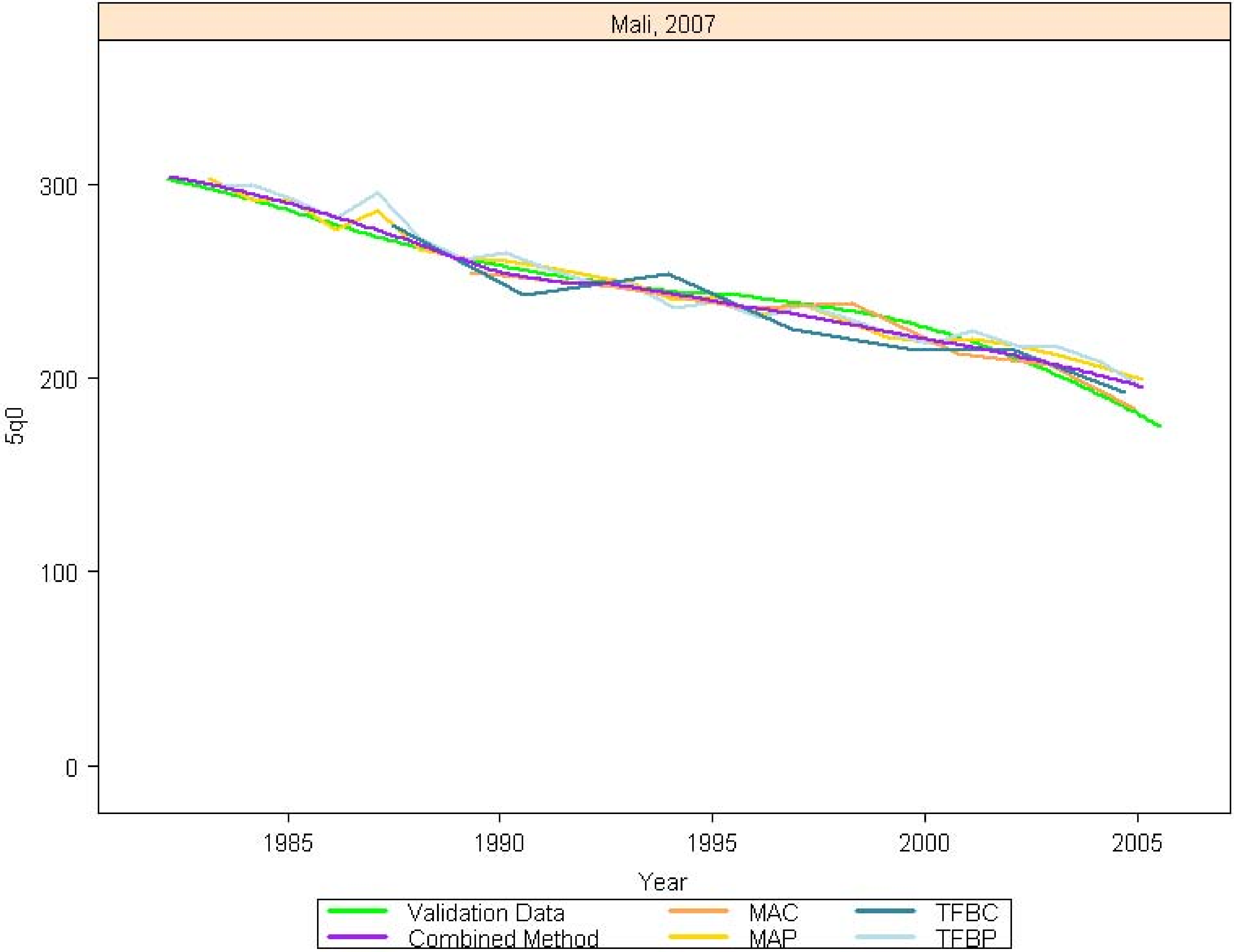
Mali, 2007
300
200
100
0
5q0
1985
1990
1995
2000
2005
Year
Validation Data
Combined Method
MAC
MAP
TFBC
TFBP

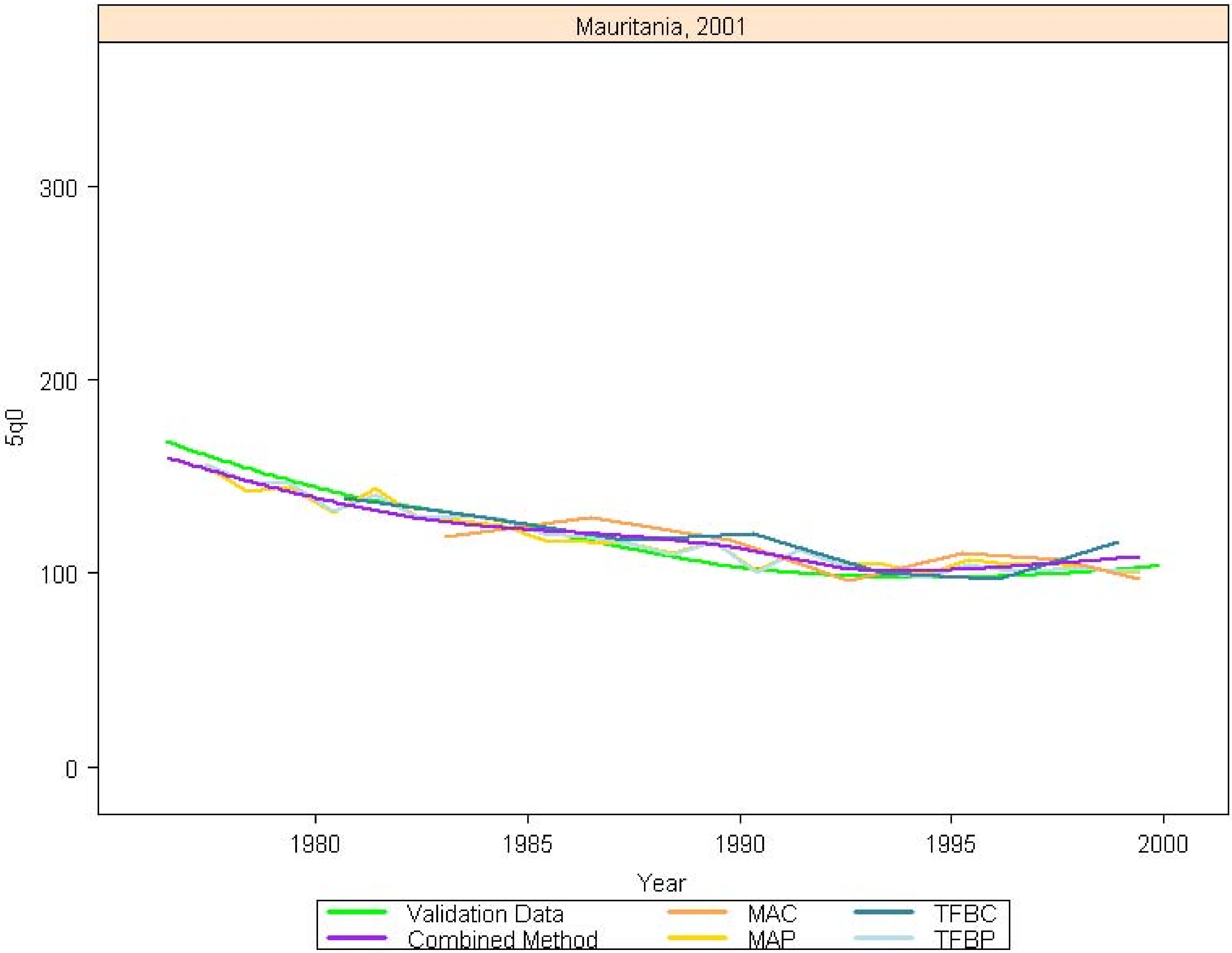

Mauritania, 2001
300
200
100
0
5q0
1980
1985
1990
1995
2000
Year
Validation Data
Combined Method
MAC
MAP
TFBC
TFBP

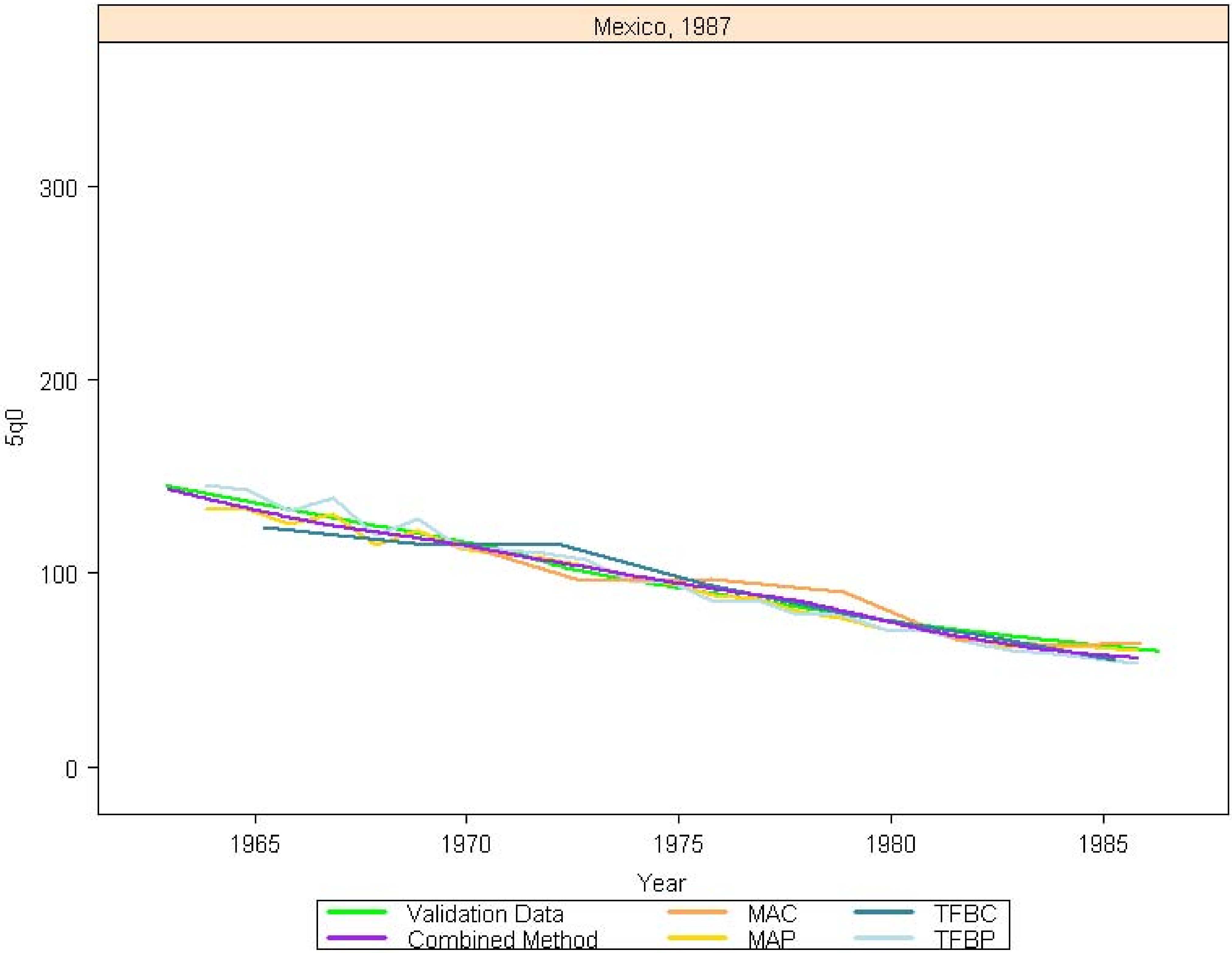

Mexico, 1987
300
200
100
0
5q0
1965
1970
1975
1980
1985
Year
Validation Data
Combined Method
MAC
MAP
TFBC
TFBP

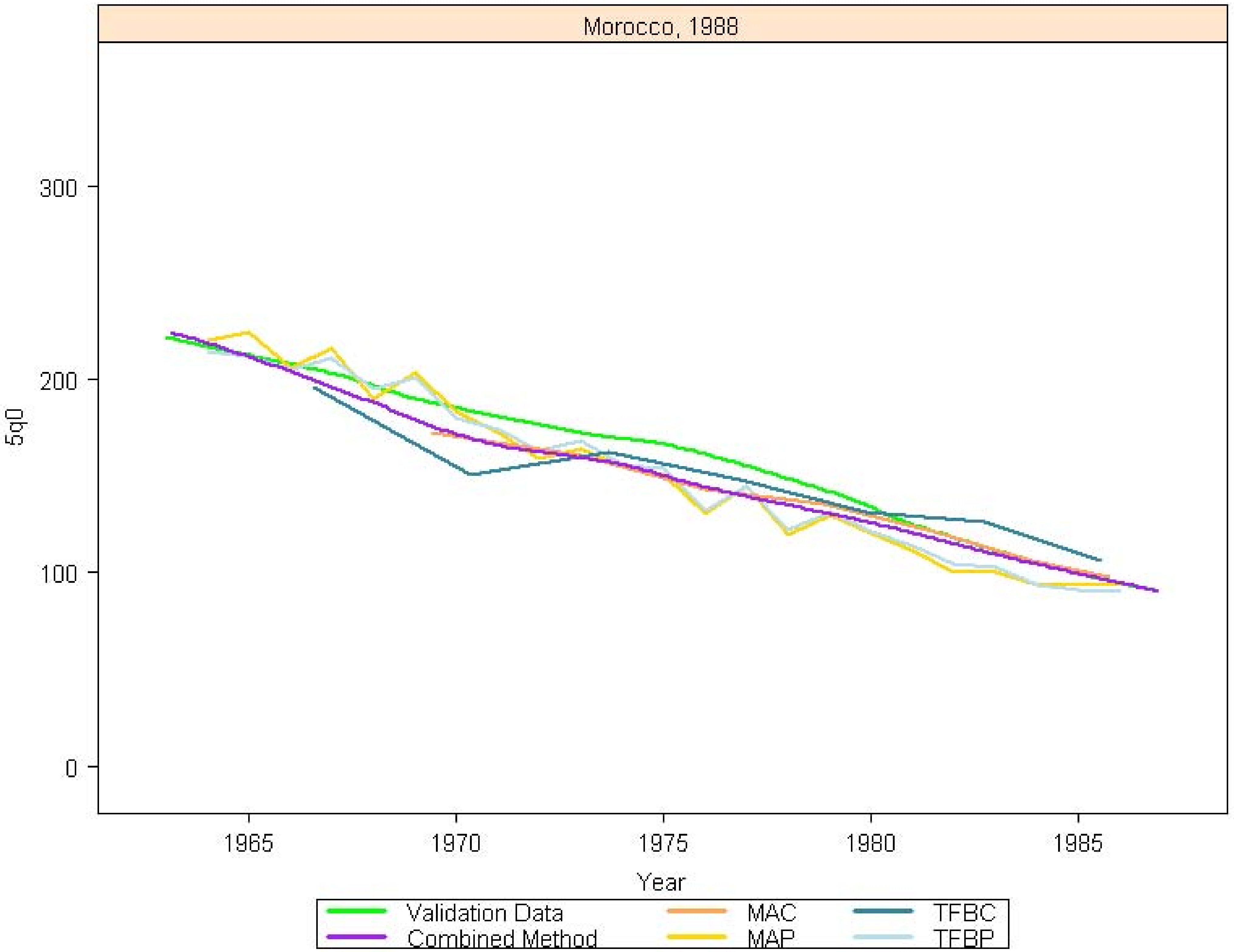
Morocco, 1988
5q0
0
100
200
300
1965
1970
1975
1980
1985
Year
Validation Data
Combined Method
MAC
MAP
TFBC
TFBP

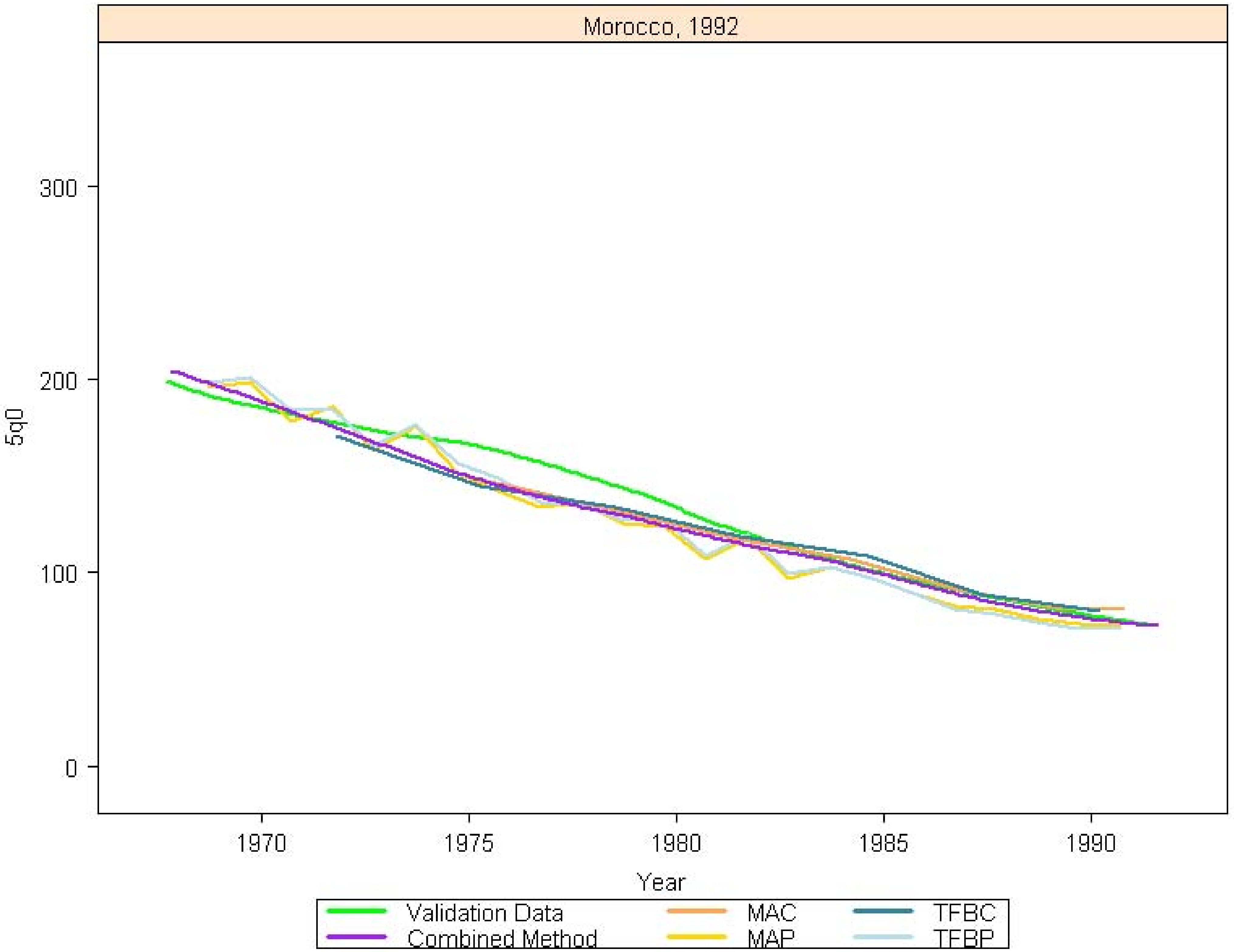

Morocco, 1992
5q0
300
200
100
0
1970
1975
1980
1985
1990
Year
Validation Data
Combined Method
MAC
MAP
TFBC
TFBP

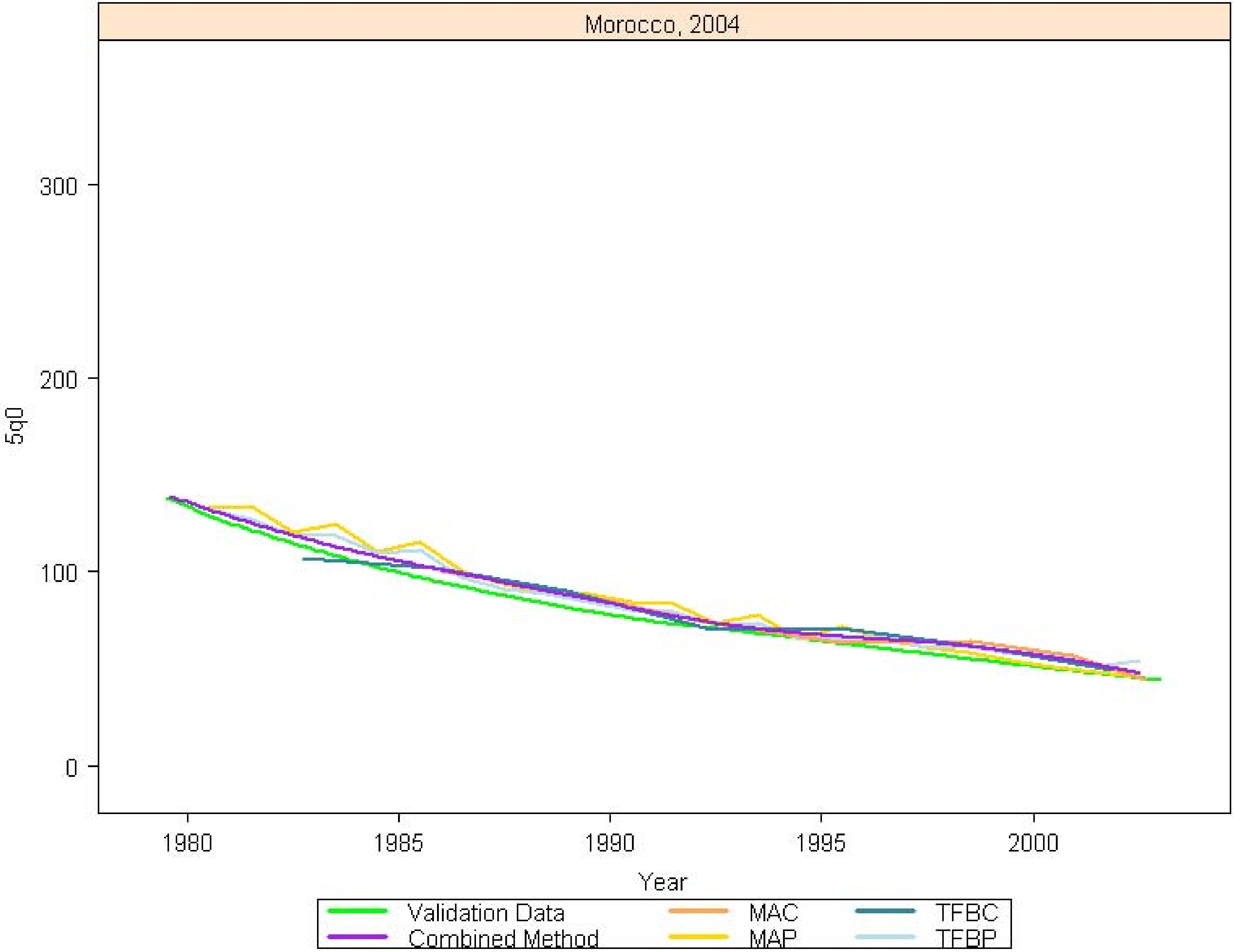

Morocco, 2004
300
200
100
0
5q0
1980
1985
1990
1995
2000
Year
Validation Data
Combined Method
MAC
MAP
TFBC
TFBP

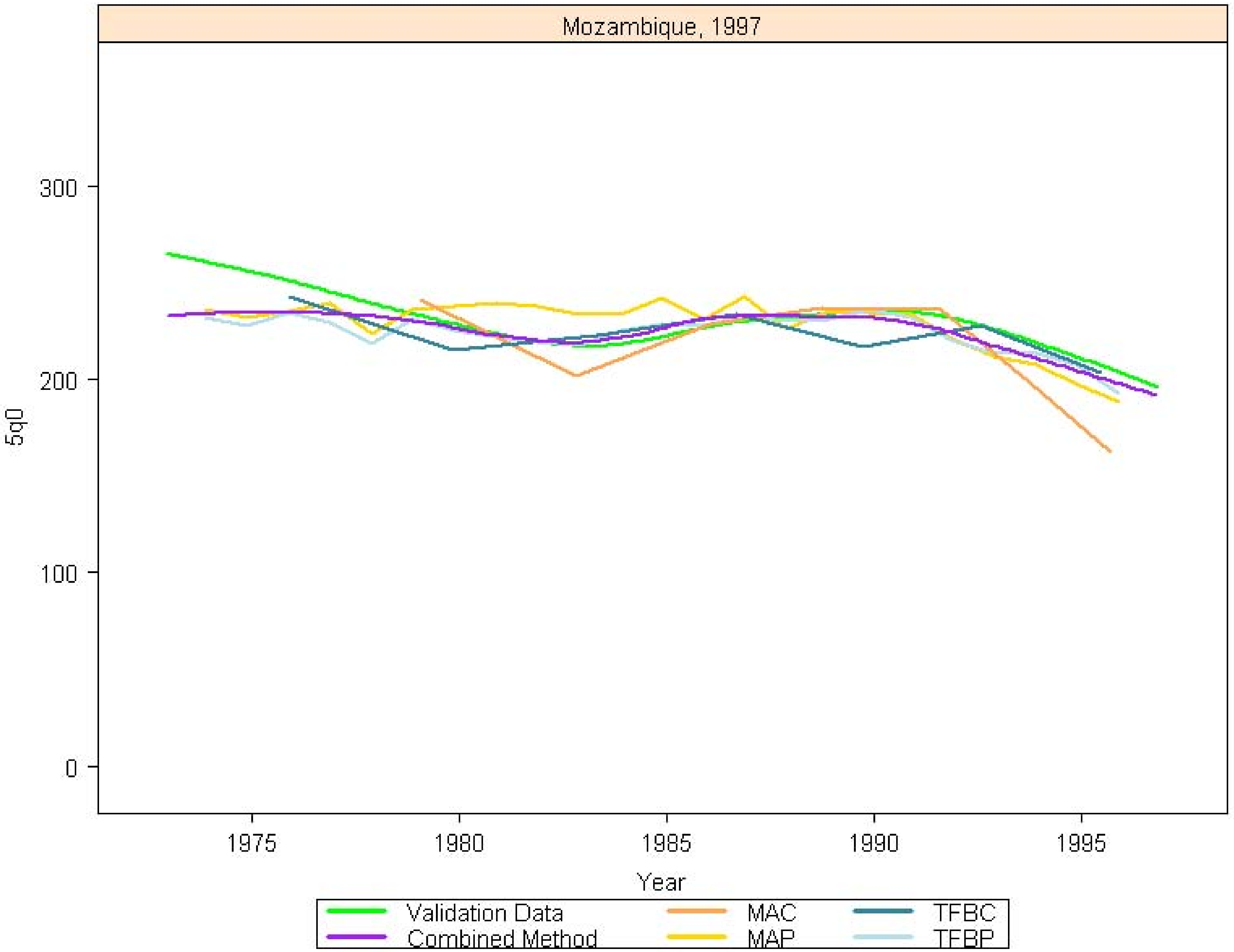
Mozambique, 1997
300
200
100
0
5q0
1975
1980
1985
1990
1995
Year
Validation Data
Combined Method
MAC
MAP
TFBC
TFBP

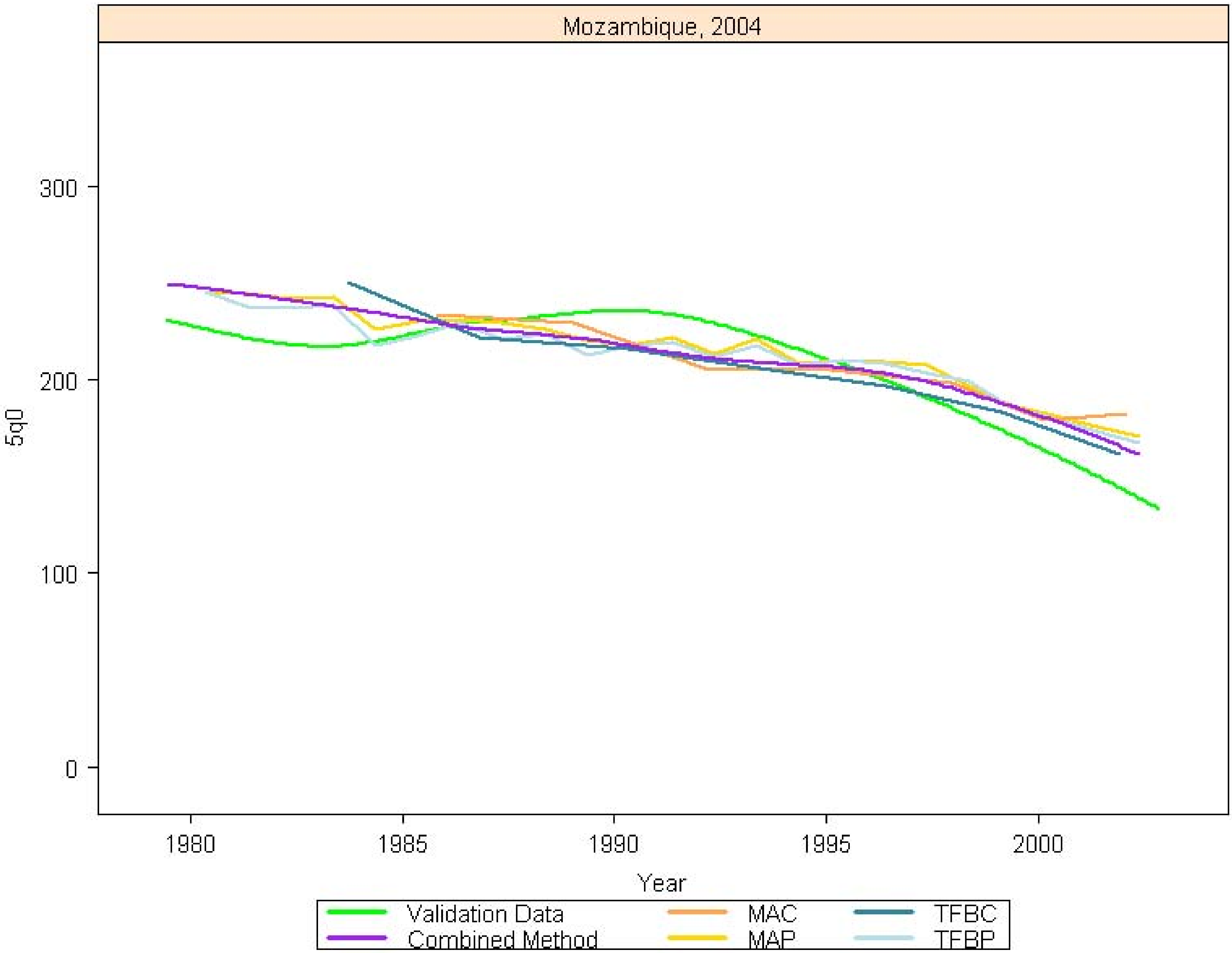

Mozambique, 2004
300
200
100
0
5q0
1980
1985
1990
1995
2000
Year
Validation Data
Combined Method
MAC
MAP
TFBC
TFBP

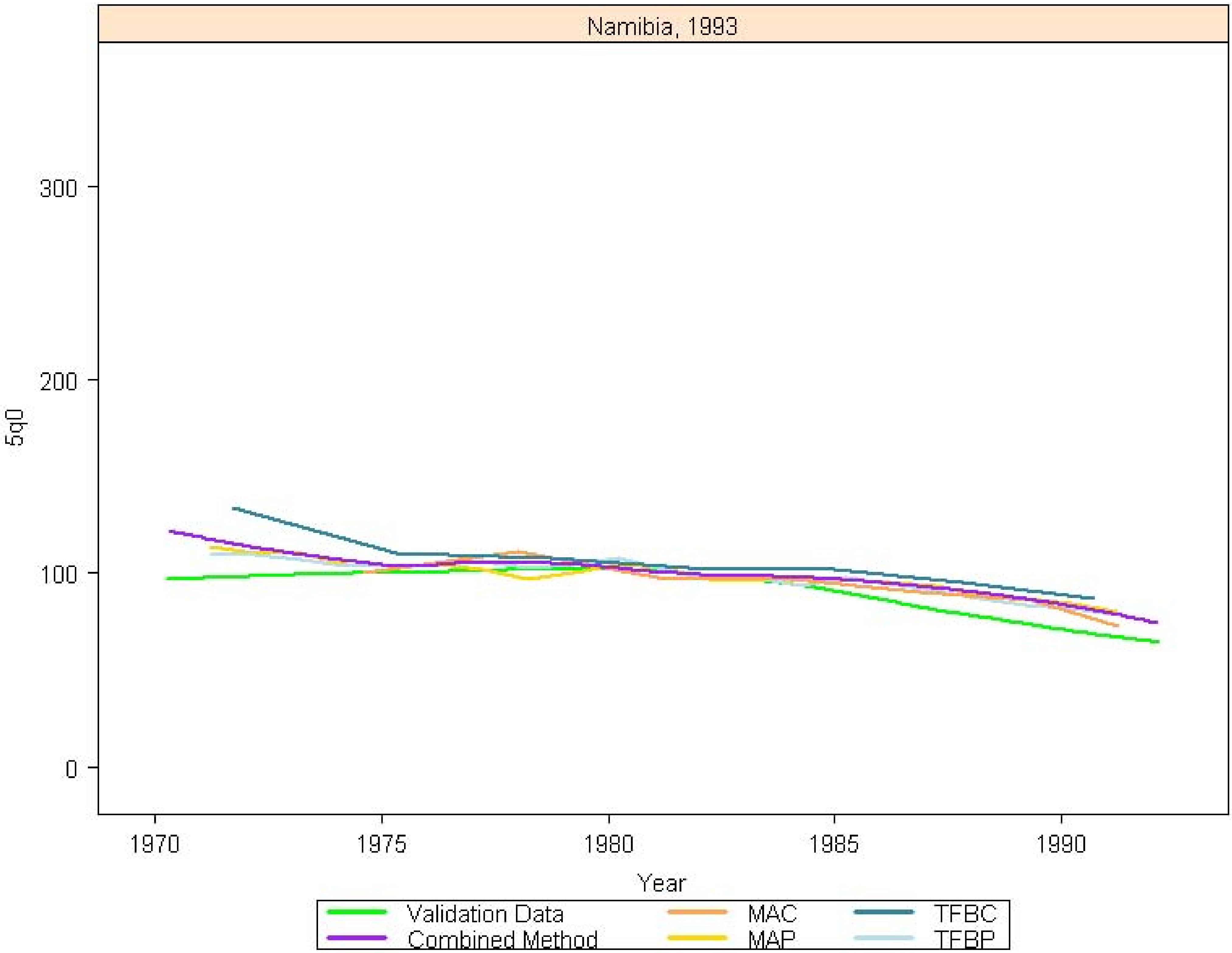

Namibia, 1993
5q0
300
200
100
0
1970
1975
1980
1985
1990
Year
Validation Data
Combined Method
MAC
MAP
TFBC
TFBP

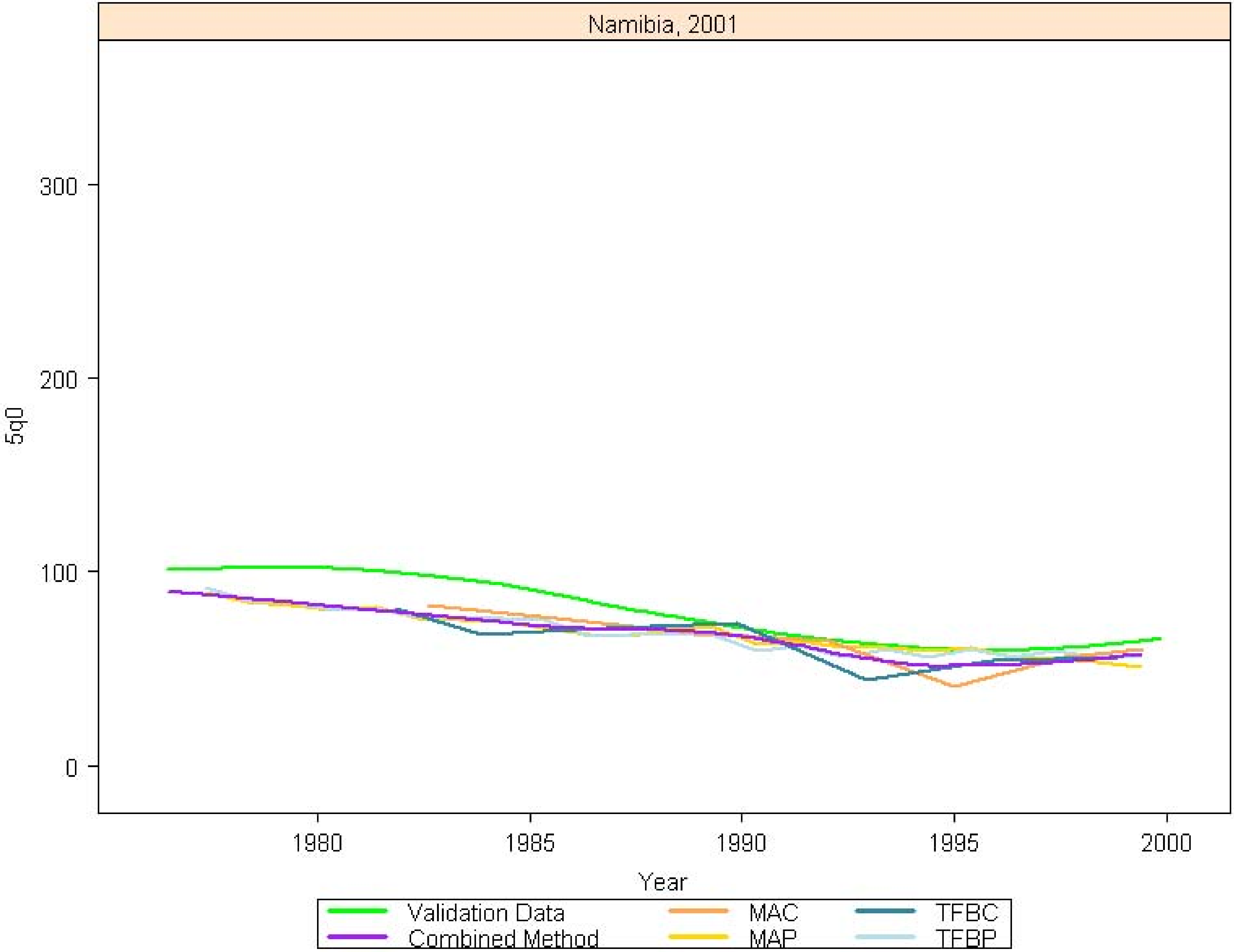
Namibia, 2001
300
200
100
0
5q0
1980
1985
1990
1995
2000
Year
Validation Data
Combined Method
MAC
MAP
TFBC
TFBP

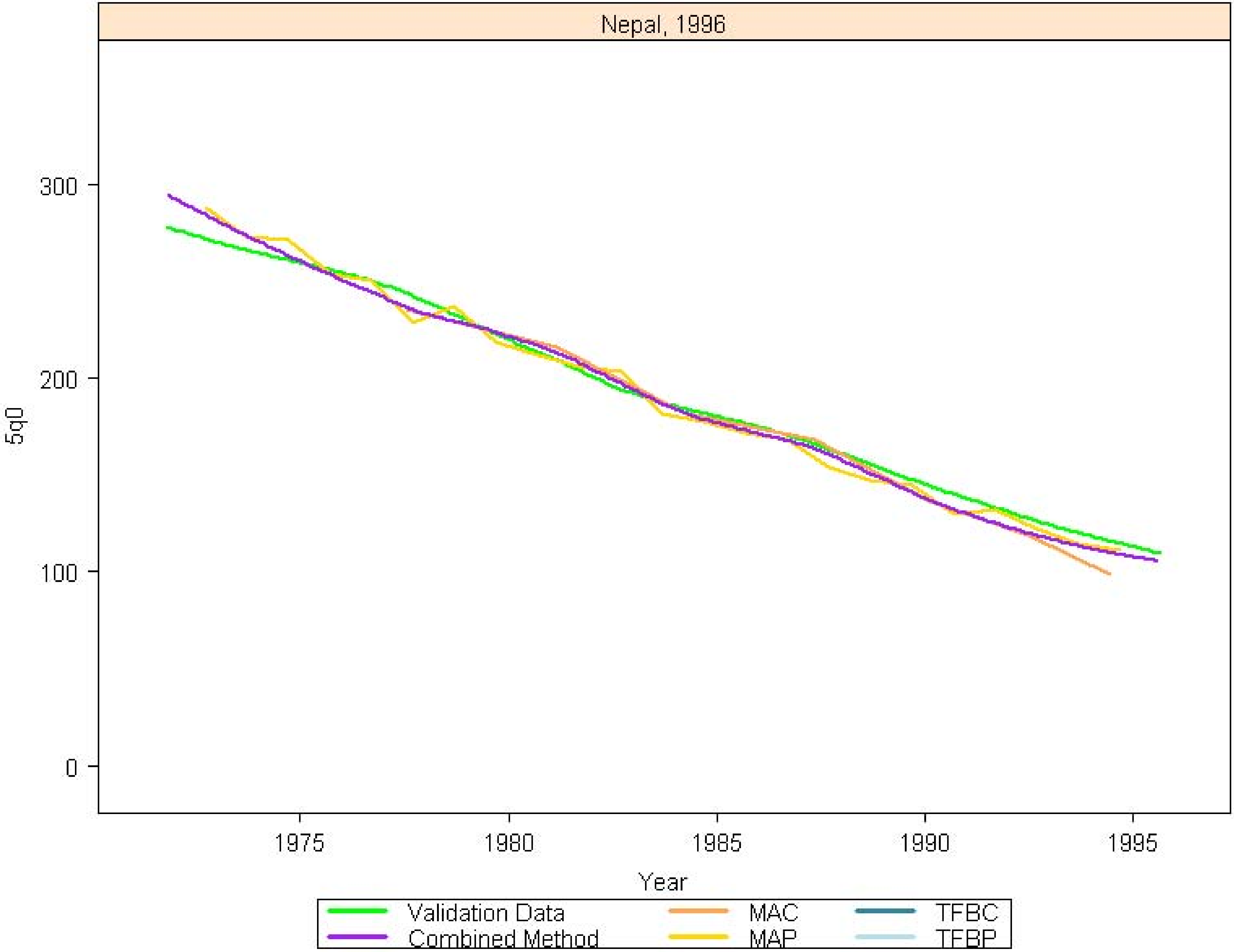
Nepal, 1996
300
200
100
0
5q0
1975
1980
1985
1990
1995
Year
Validation Data
Combined Method
MAC
MAP
TFBC
TFBP

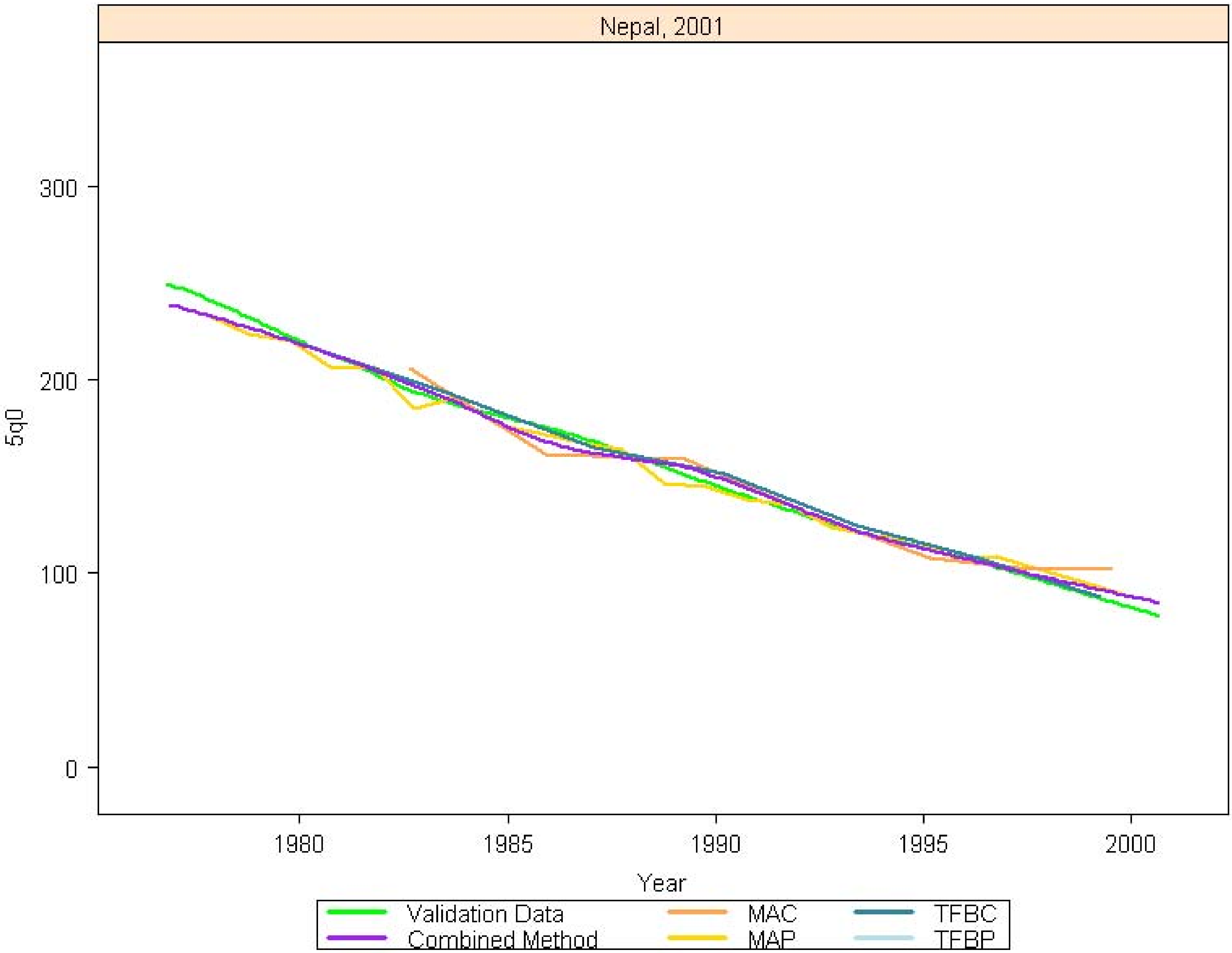

Nepal, 2001
300
200
100
0
5q0
1980
1985
1990
1995
2000
Year
Validation Data
Combined Method
MAC
MAP
TFBC
TFBP

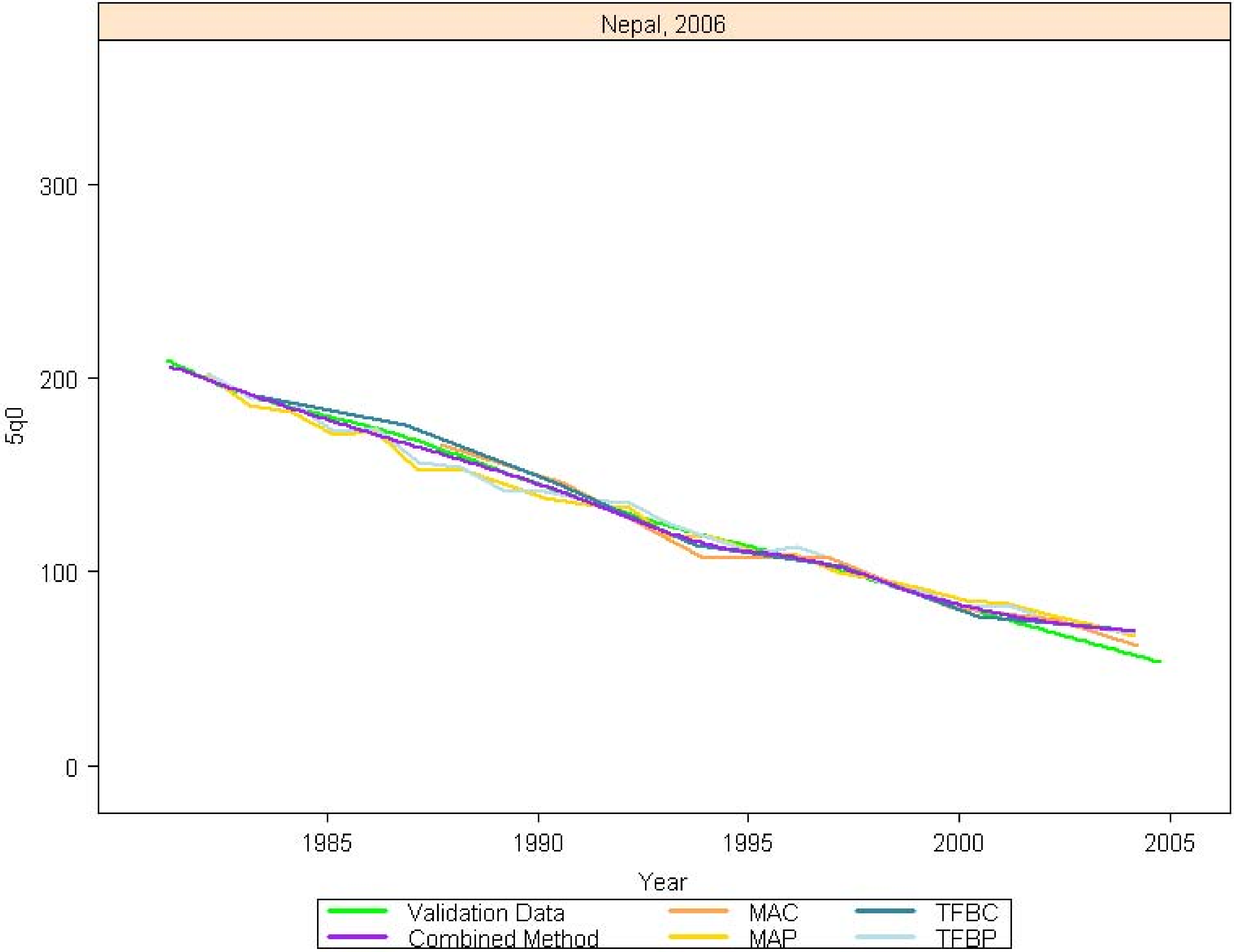

Nepal, 2006
300
200
100
0
5q0
1985
1990
1995
2000
2005
Year
Validation Data
Combined Method
MAC
MAP
TFBC
TFBP

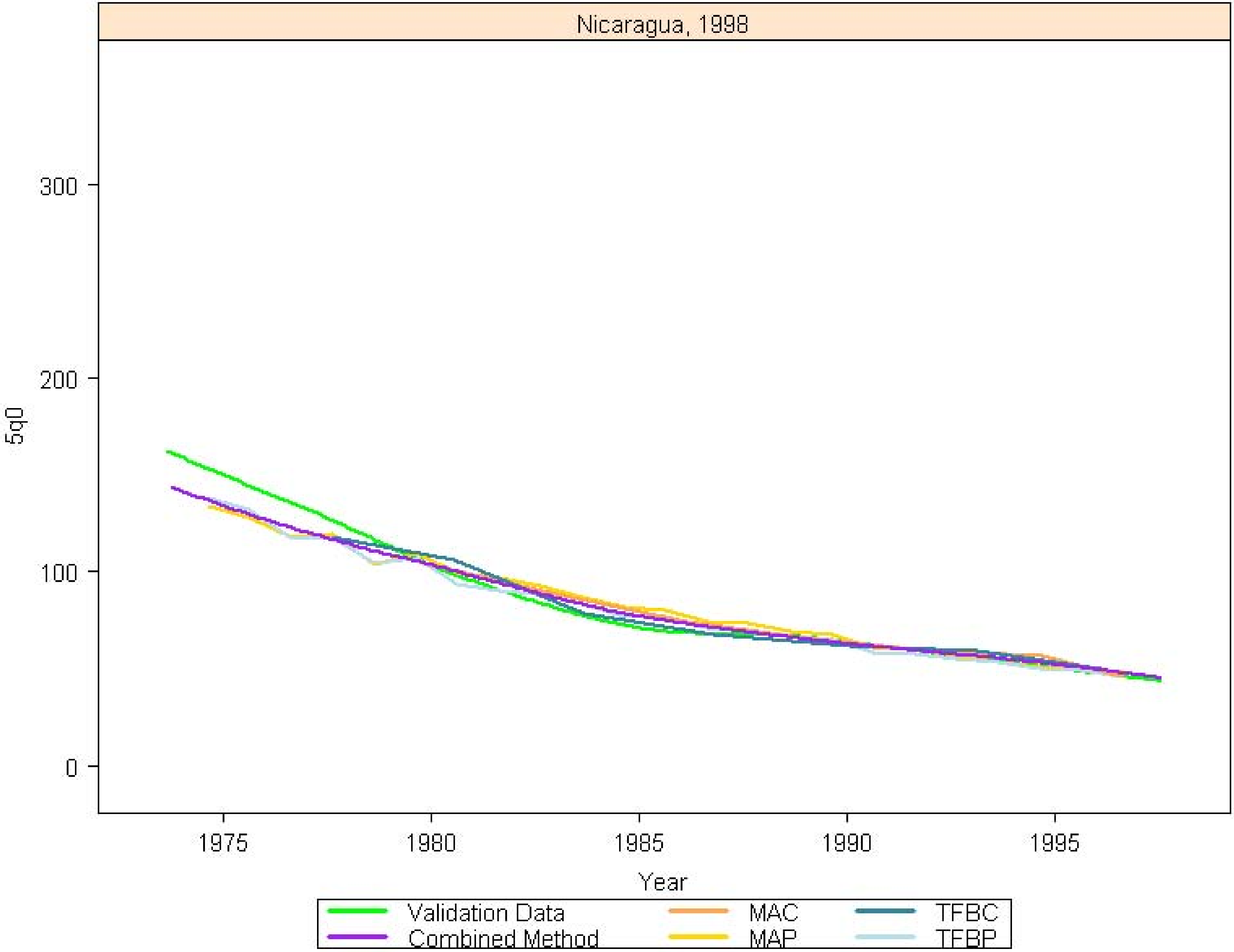

Nicaragua, 1998
5q0
300
200
100
0
1975
1980
1985
1990
1995
Year
Validation Data
Combined Method
MAC
MAP
TFBC
TFBP

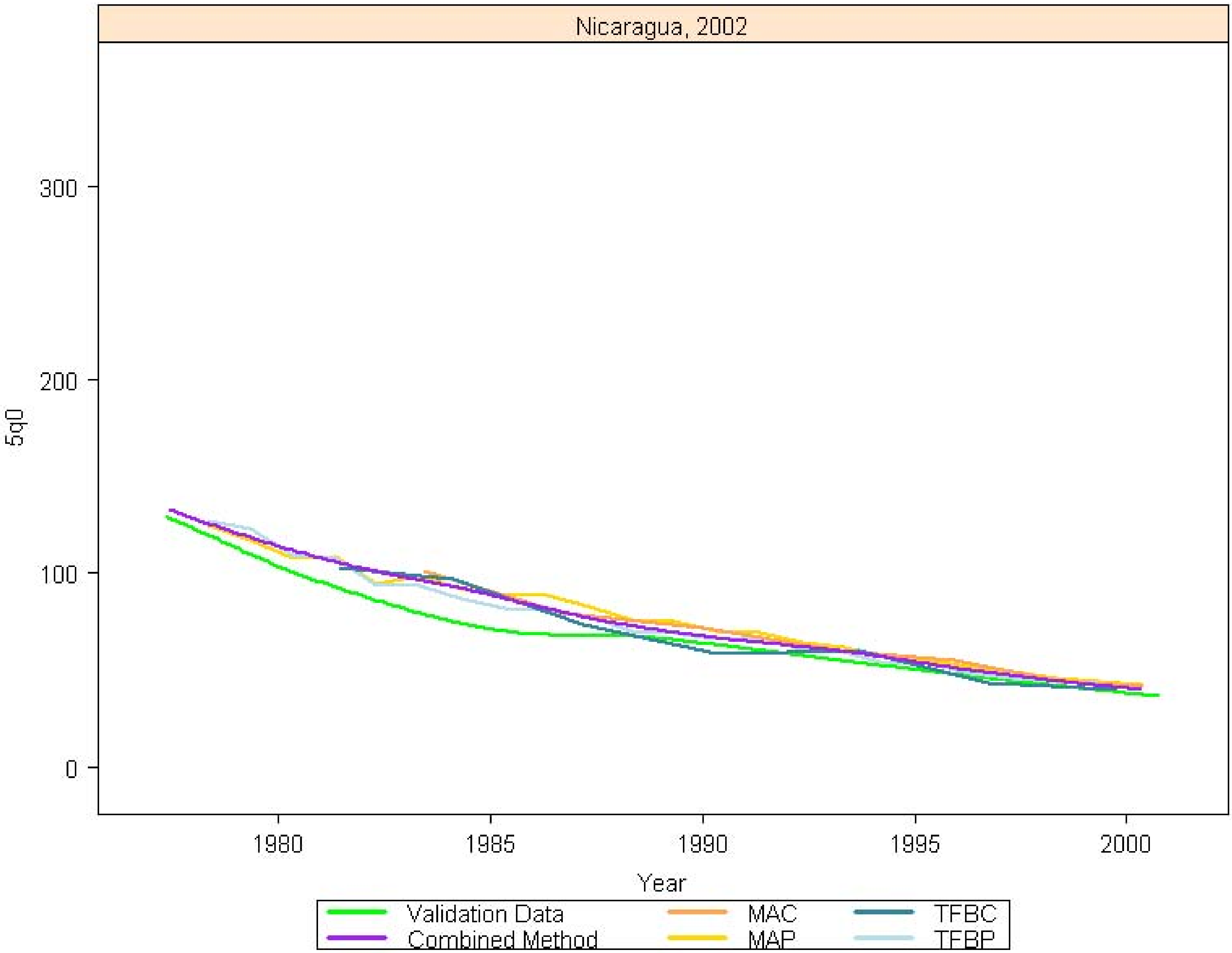

Nicaragua, 2002
300
200
100
0
5q0
1980
1985
1990
1995
2000
Year
Validation Data
Combined Method
MAC
MAP
TFBC
TFBP

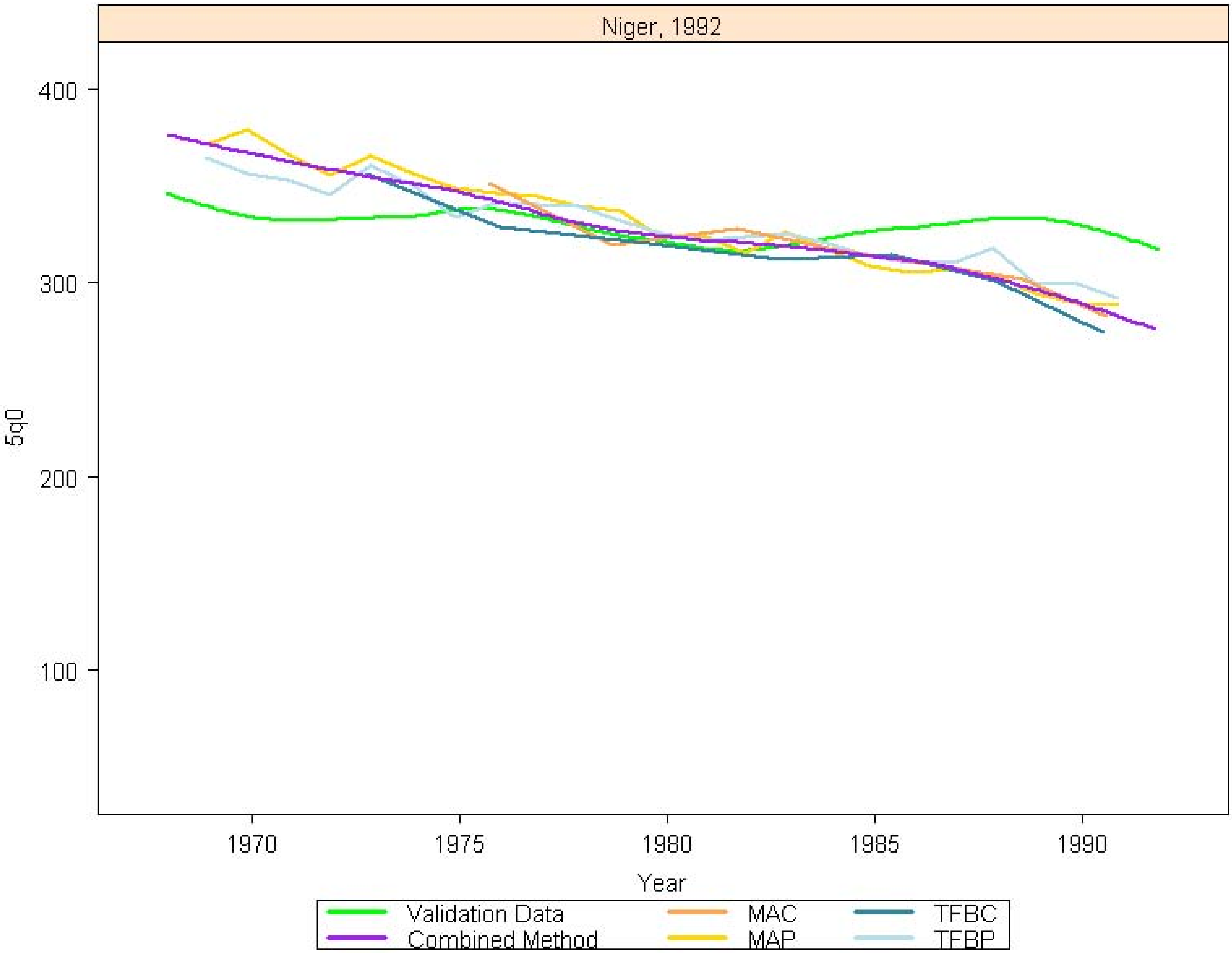
Niger, 1992
400
300
200
100
5q0
1970
1975
1980
1985
1990
Year
Validation Data
Combined Method
MAC
MAP
TFBC
TFBP

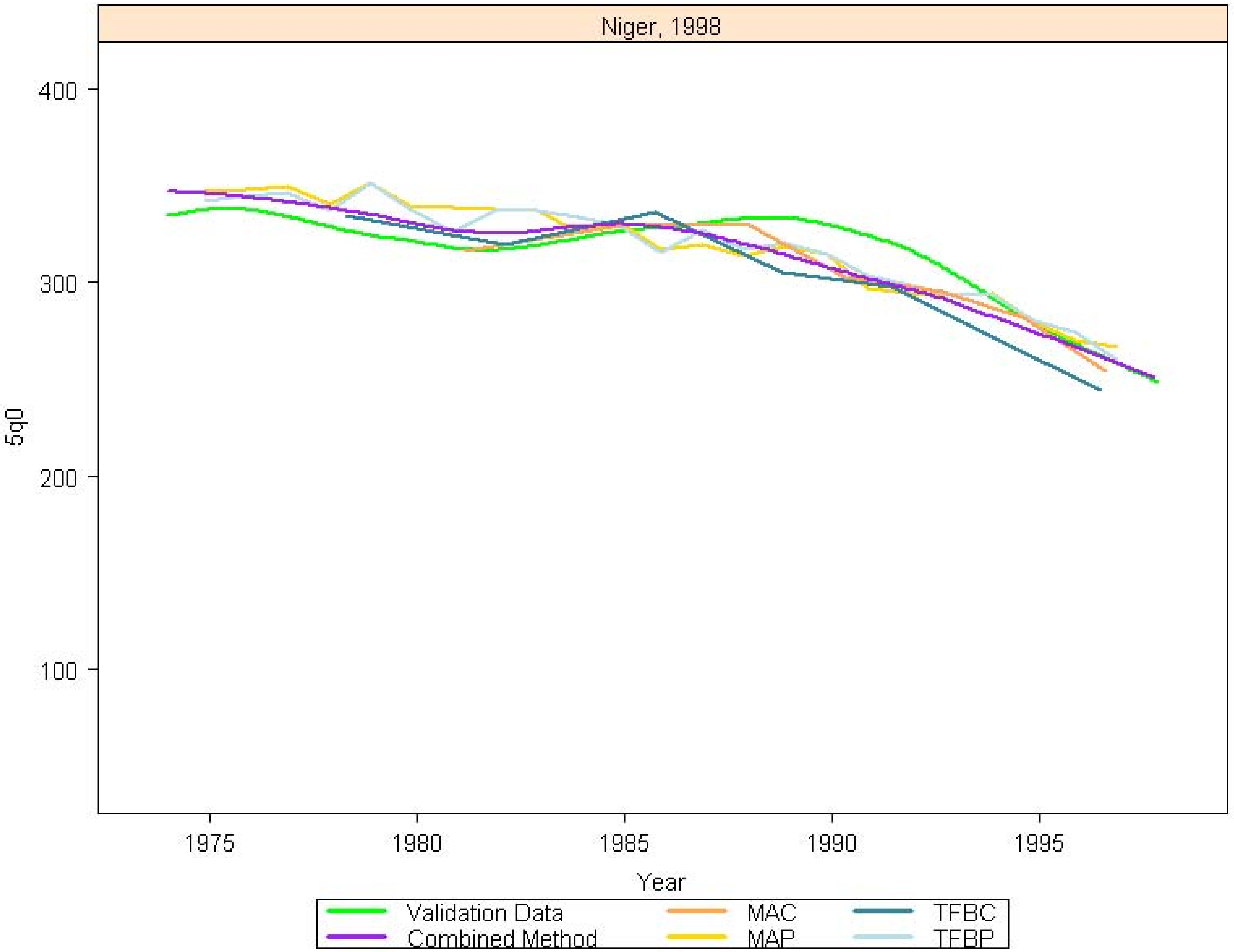

Niger, 1998
5q0
400
300
200
100
1975
1980
1985
1990
1995
Year
Validation Data
Combined Method
MAC
MAP
TFBC
TFBP

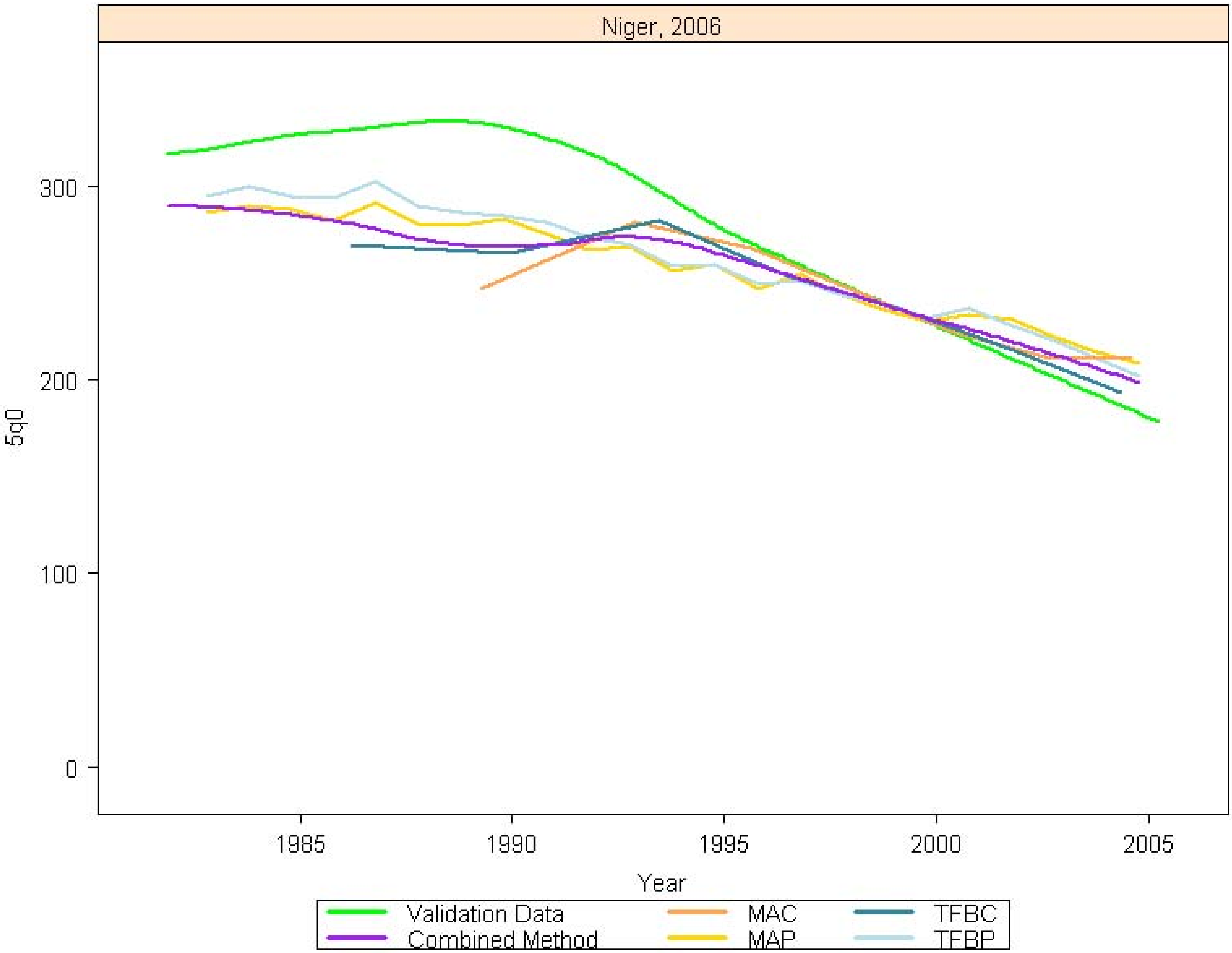

Niger, 2006
300
200
100
0
5q0
1985
1990
1995
2000
2005
Year
Validation Data
Combined Method
MAC
MAP
TFBC
TFBP

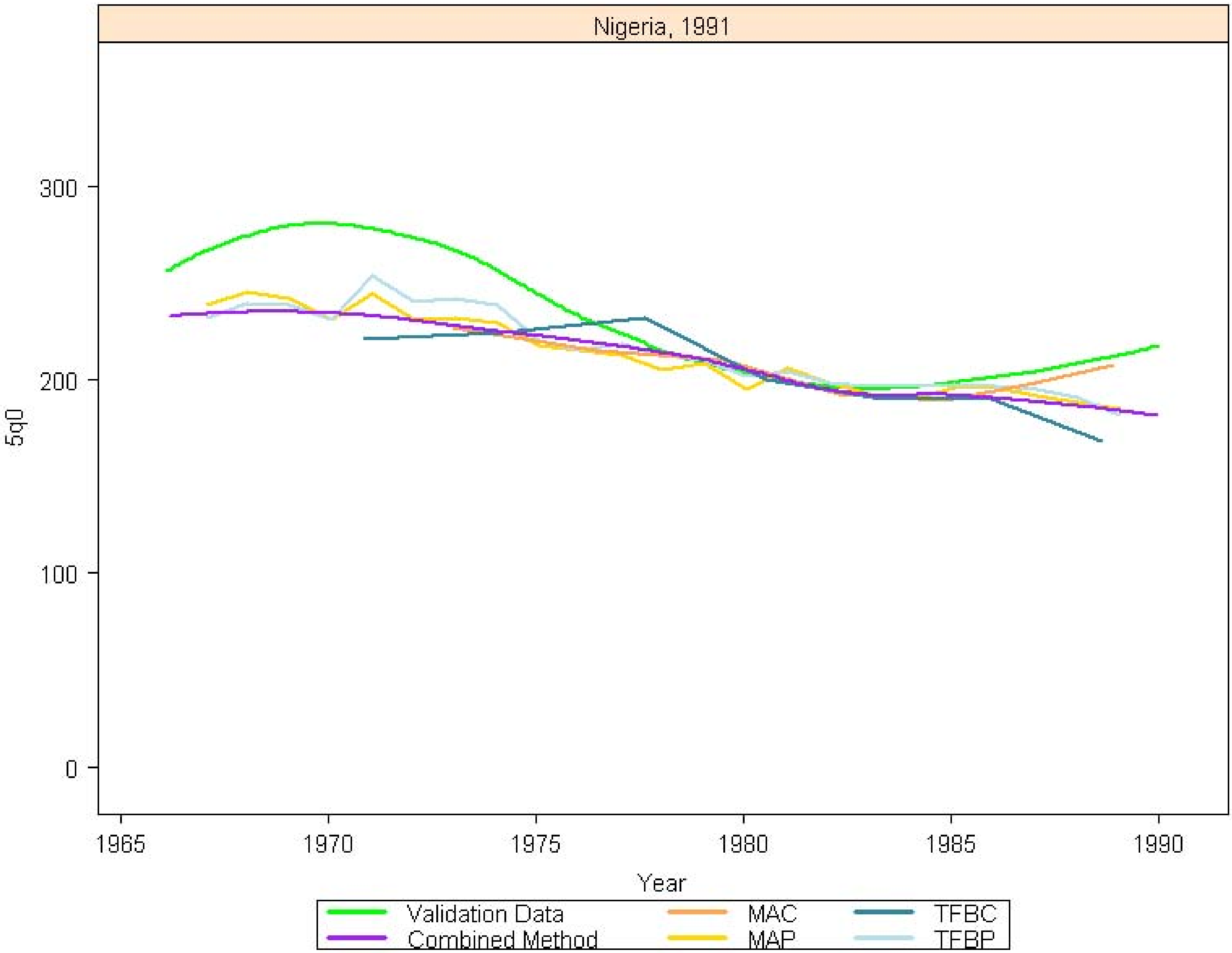

Nigeria, 1991
5q0
300
200
100
0
1965
1970
1975
1980
1985
1990
Year
Validation Data
Combined Method
MAC
MAP
TFBC
TFBP

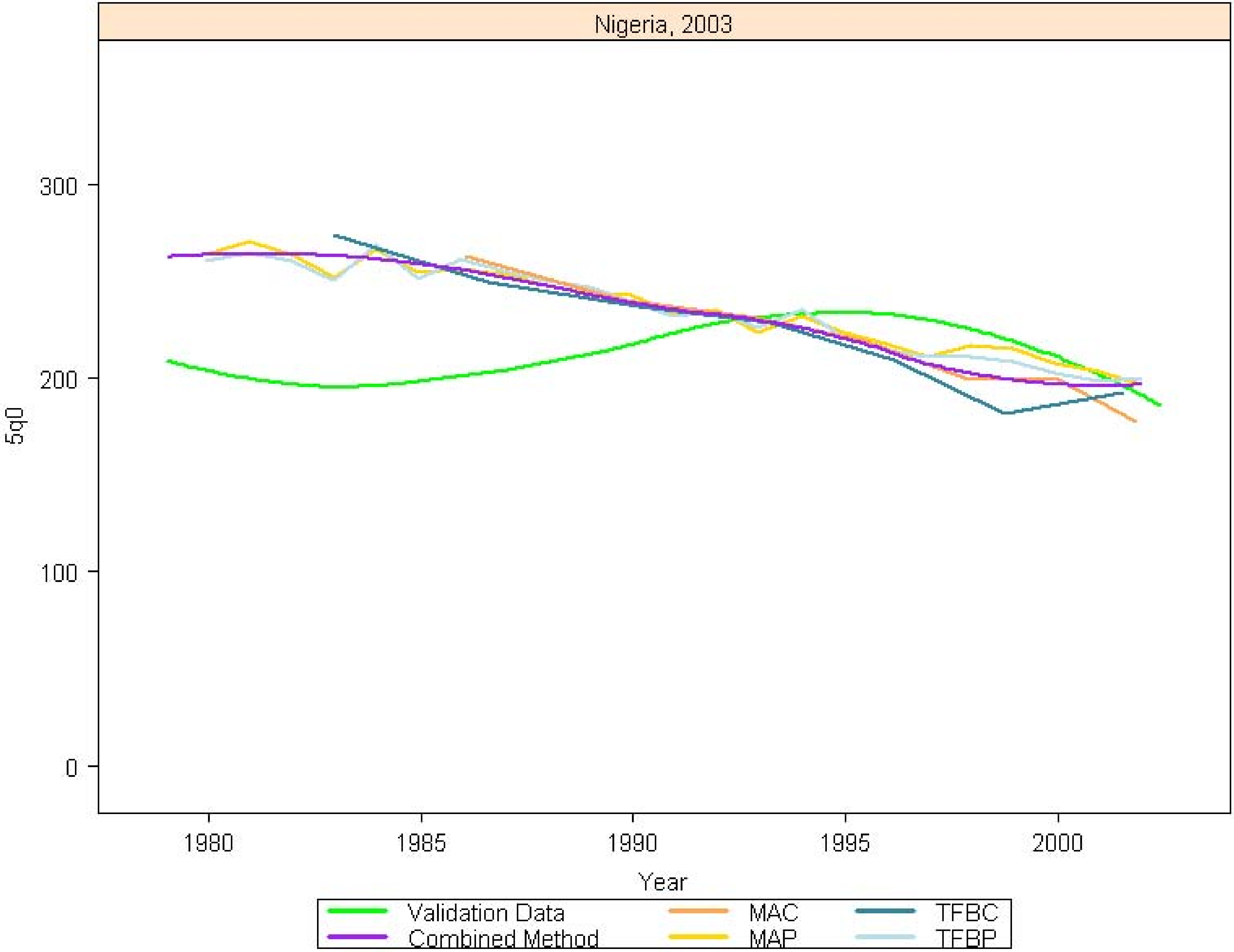
Nigeria, 2003
300
200
100
0
5q0
1980
1985
1990
1995
2000
Year
Validation Data
Combined Method
MAC
MAP
TFBC
TFBP

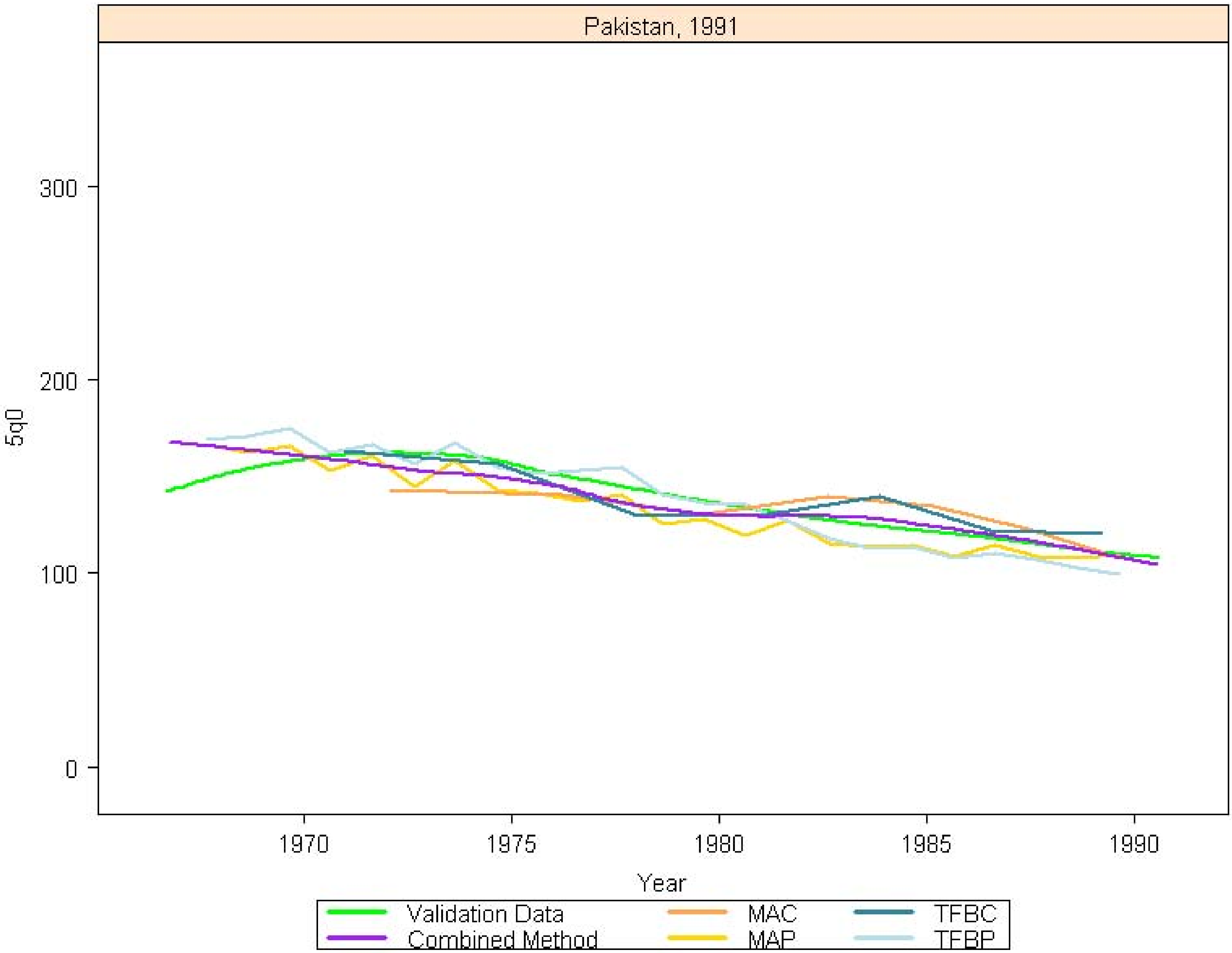
Pakistan, 1991
300
200
100
0
5q0
1970
1975
1980
1985
1990
Year
Validation Data
Combined Method
MAC
MAP
TFBC
TFBP

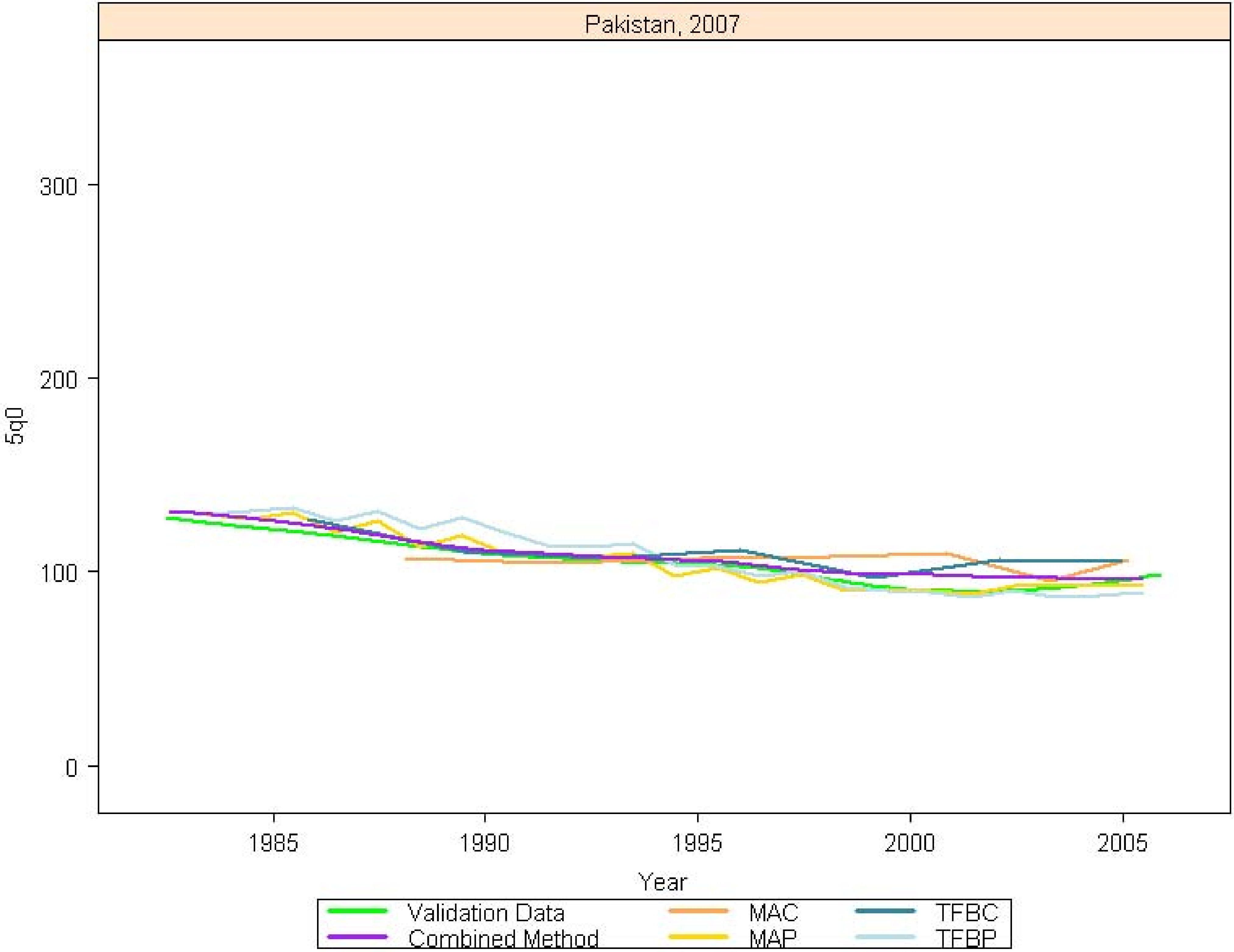
Pakistan, 2007
300
200
100
0
5q0
1985
1990
1995
2000
2005
Year
Validation Data
Combined Method
MAC
MAP
TFBC
TFBP

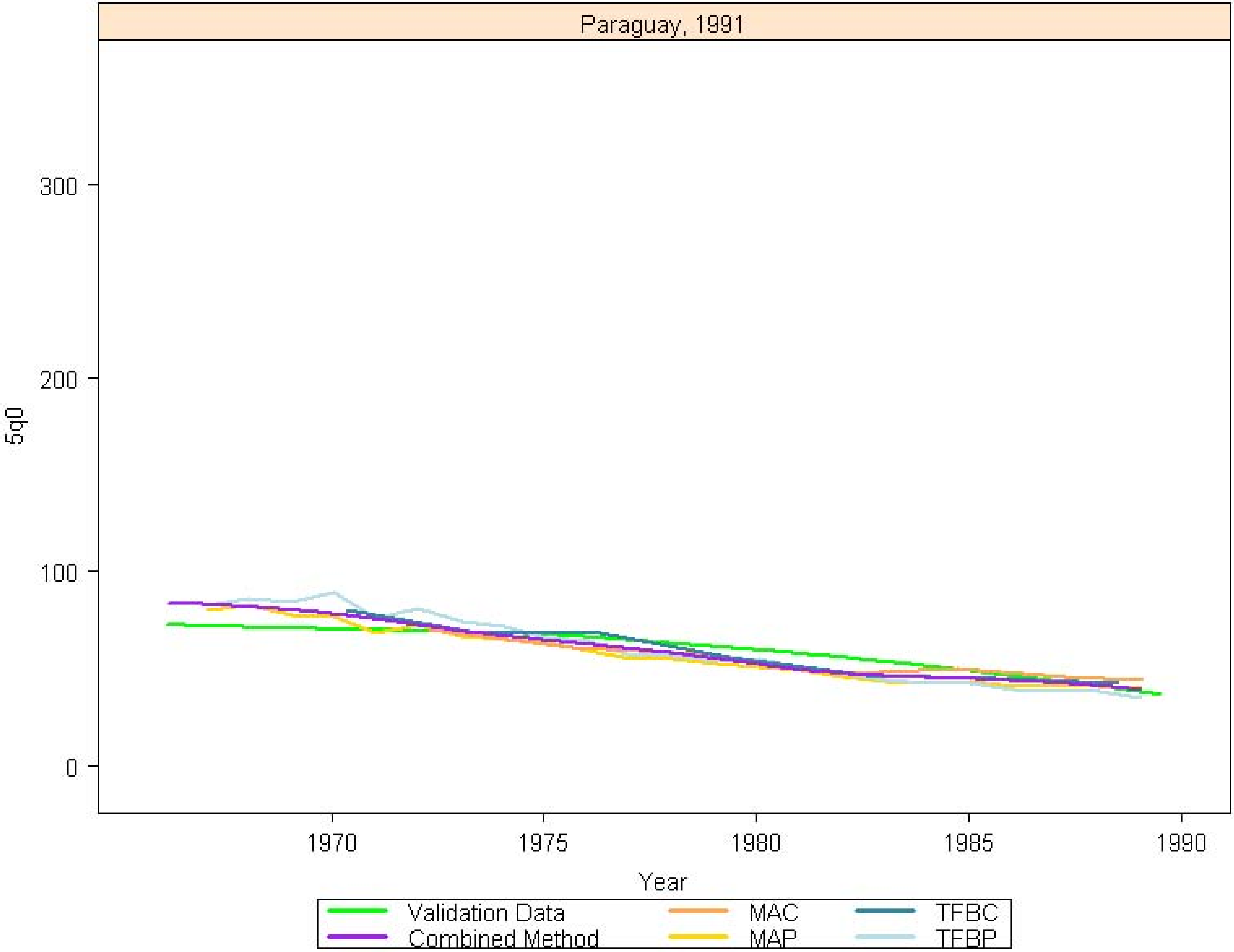

Paraguay, 1991
5q0
300
200
100
0
1970
1975
1980
1985
1990
Year
Validation Data
Combined Method
MAC
MAP
TFBC
TFBP

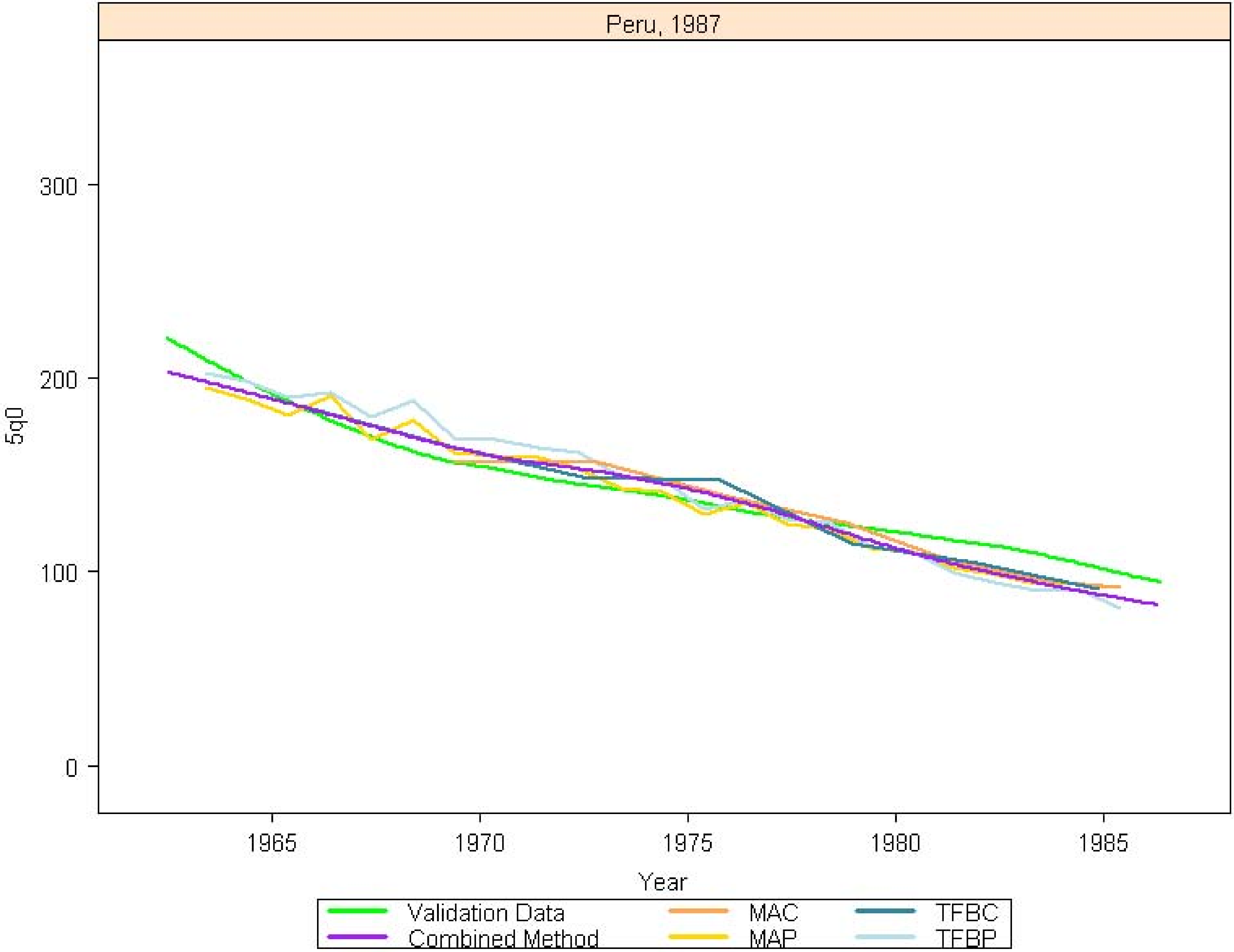
Peru, 1987
300
200
100
0
5q0
1965
1970
1975
1980
1985
Year
Validation Data
Combined Method
MAC
MAP
TFBC
TFBP

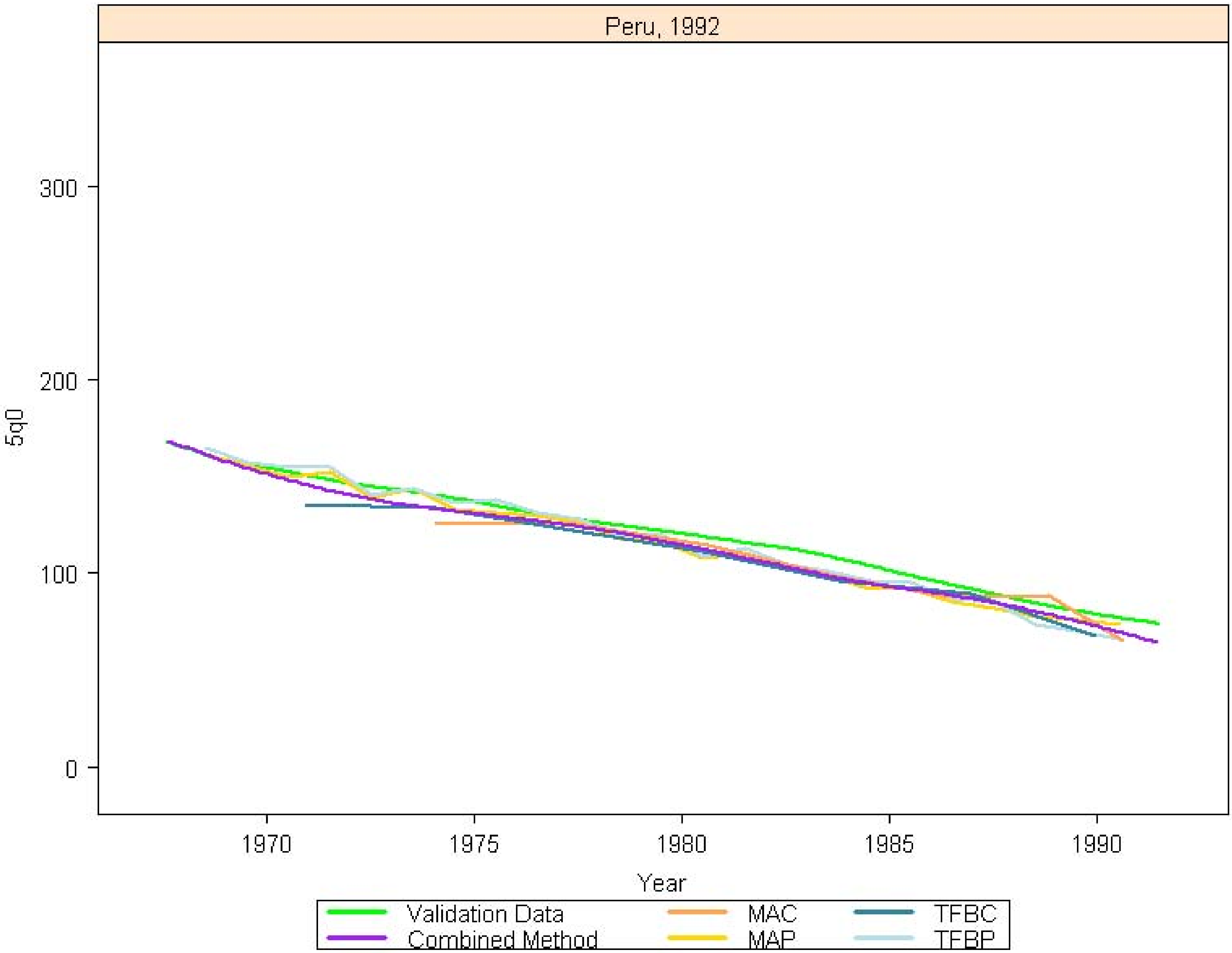
Peru, 1992
300
200
100
0
5q0
1970
1975
1980
1985
1990
Year
Validation Data
Combined Method
MAC
MAP
TFBC
TFBP

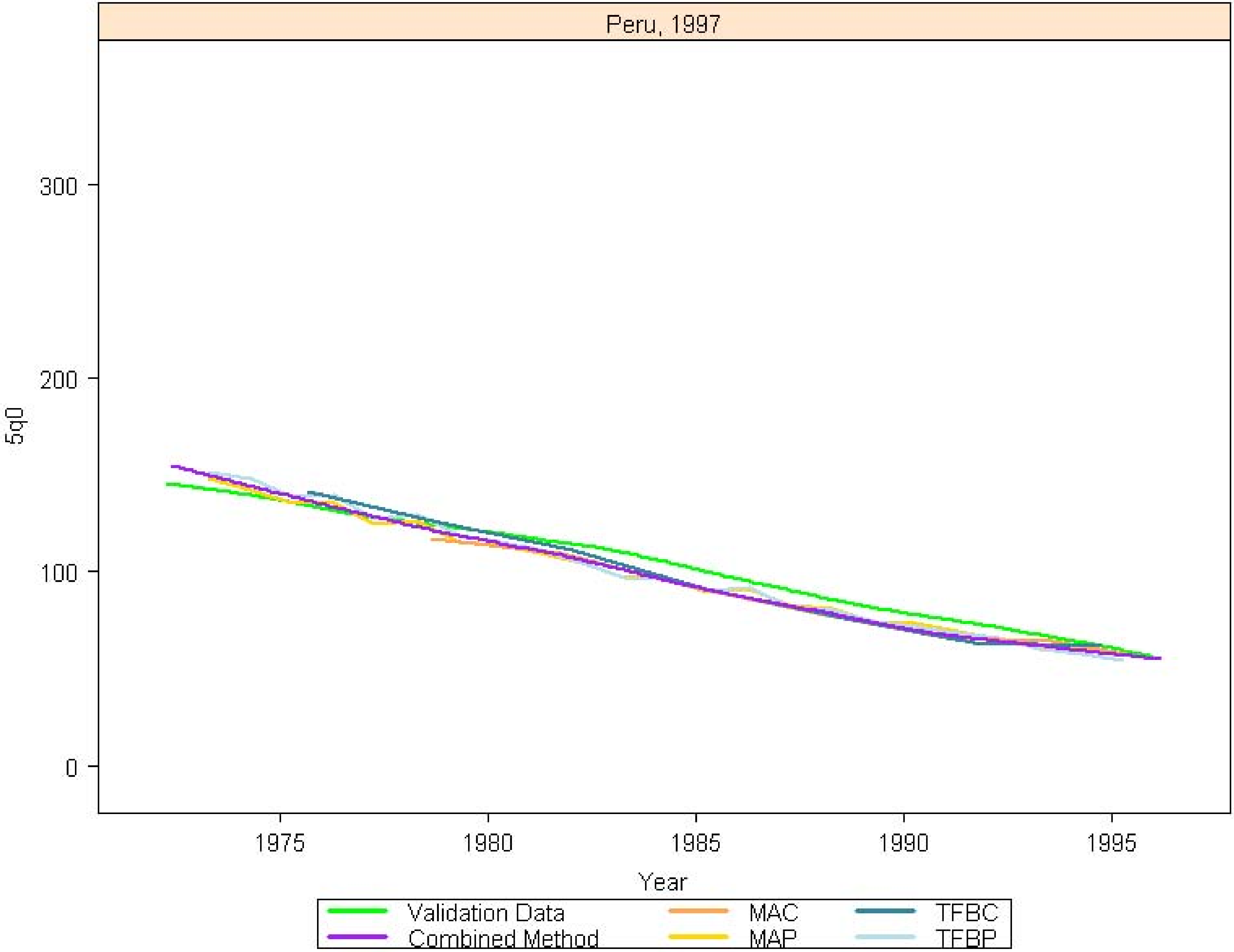
Peru, 1997
5q0
0
100
200
300
1975
1980
1985
1990
1995
Year
Validation Data
Combined Method
MAC
MAP
TFBC
TFBP

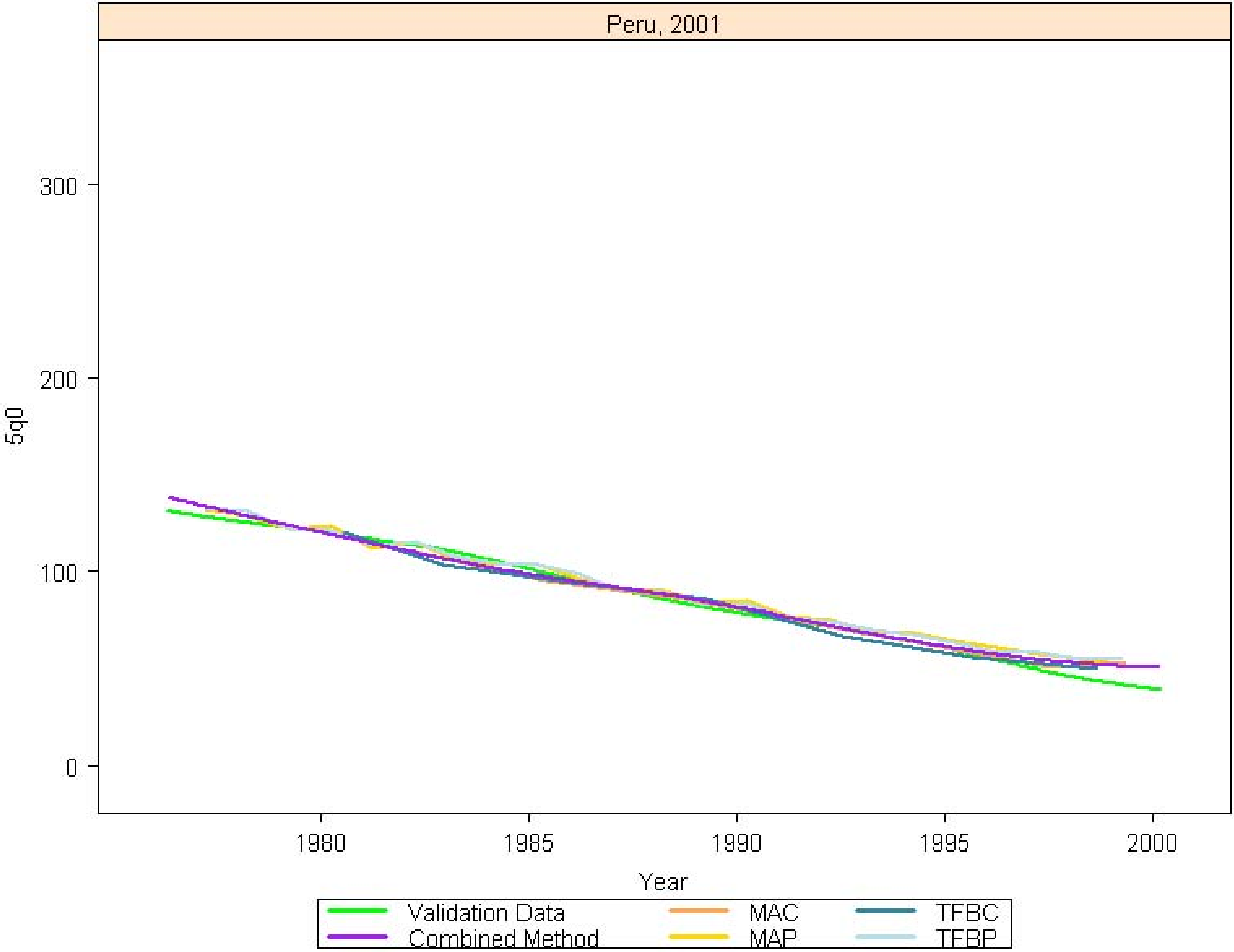

Peru, 2001
300
200
100
0
5q0
1980
1985
1990
1995
2000
Year
Validation Data
Combined Method
MAC
MAP
TFBC
TFBP

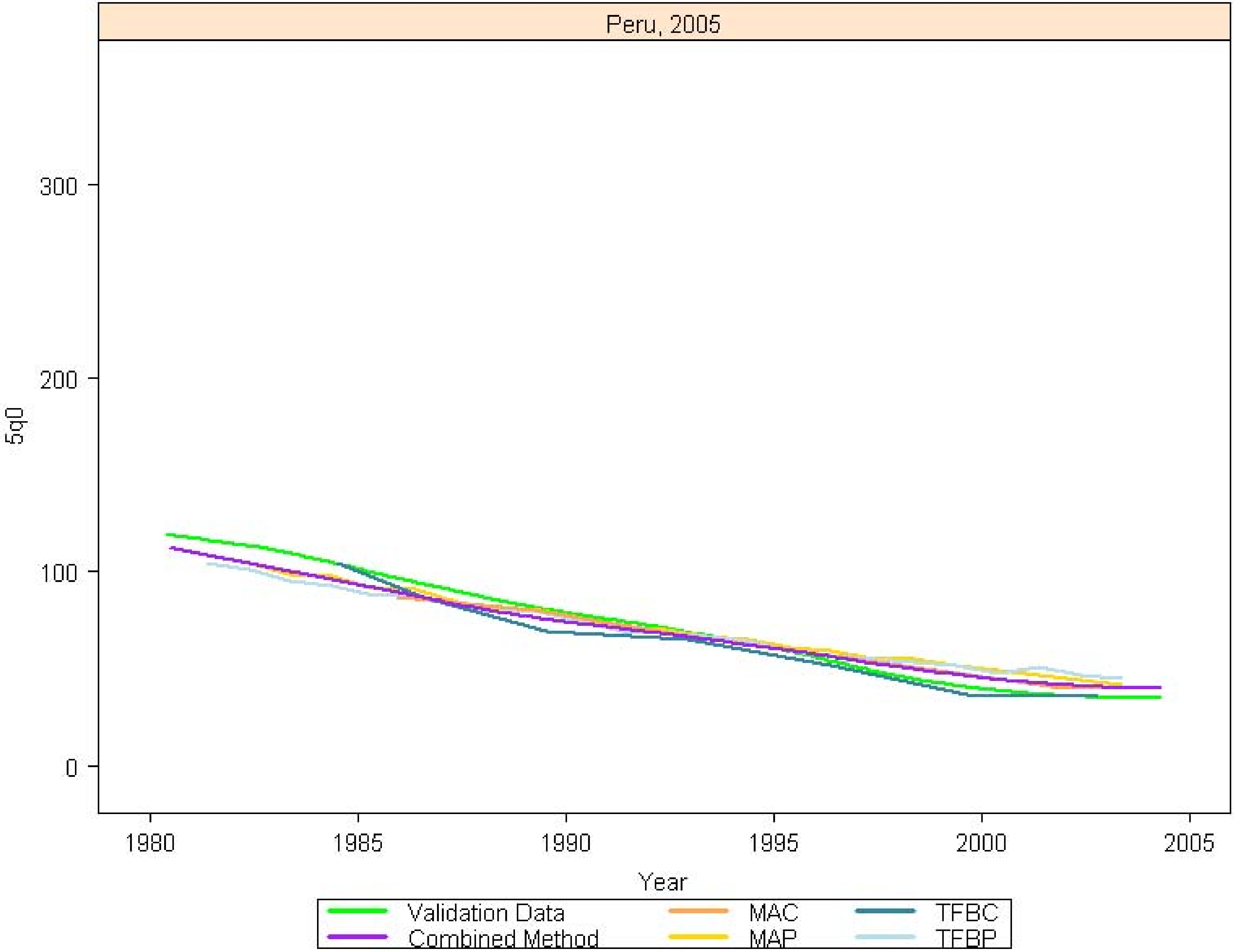
Peru, 2005
300
200
100
0
5q0
1980
1985
1990
1995
2000
2005
Year
Validation Data
Combined Method
MAC
MAP
TFBC
TFBP

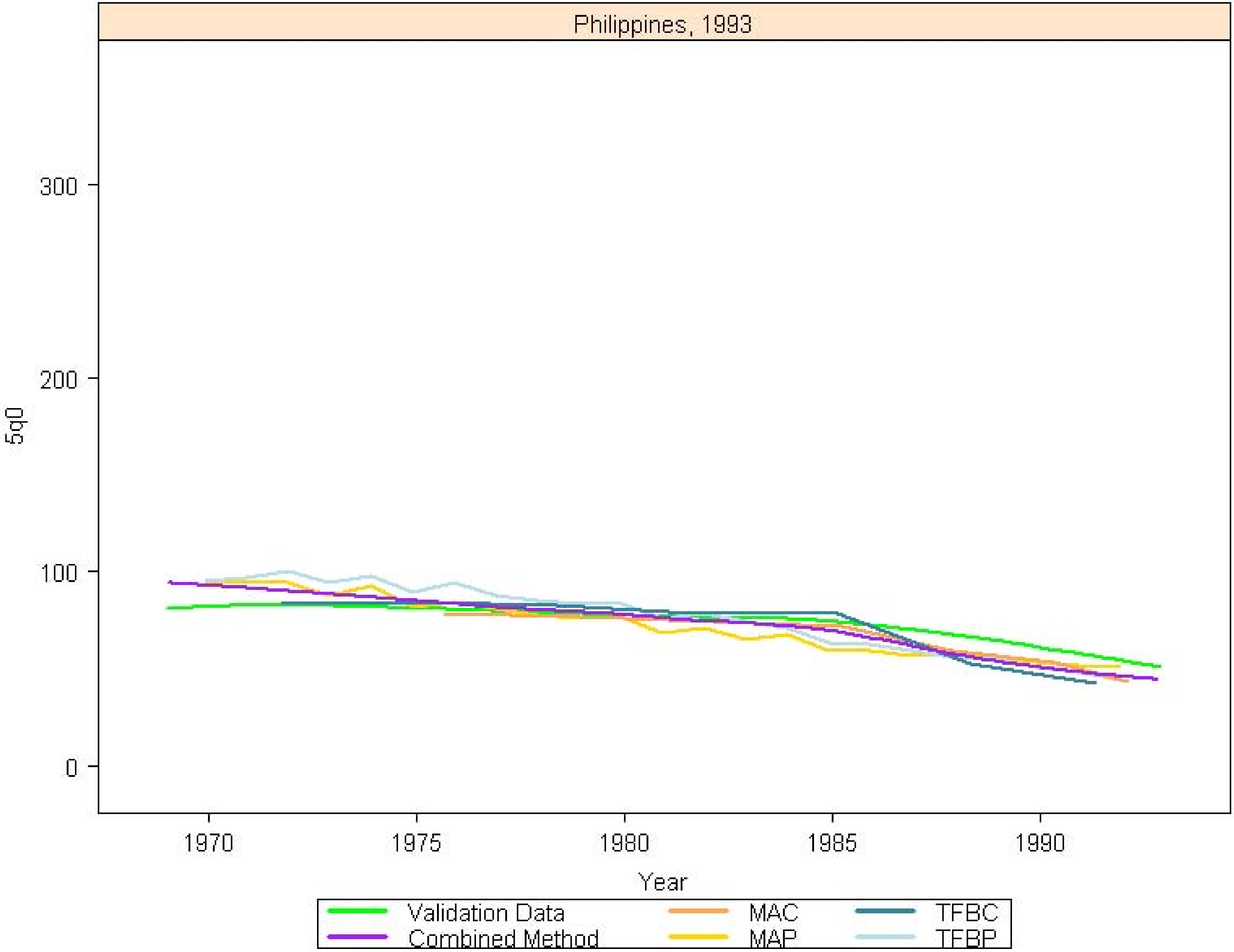
Philippines, 1993
5q0
0
100
200
300
1970
1975
1980
1985
1990
Year
Validation Data
Combined Method
MAC
MAP
TFBC
TFBP

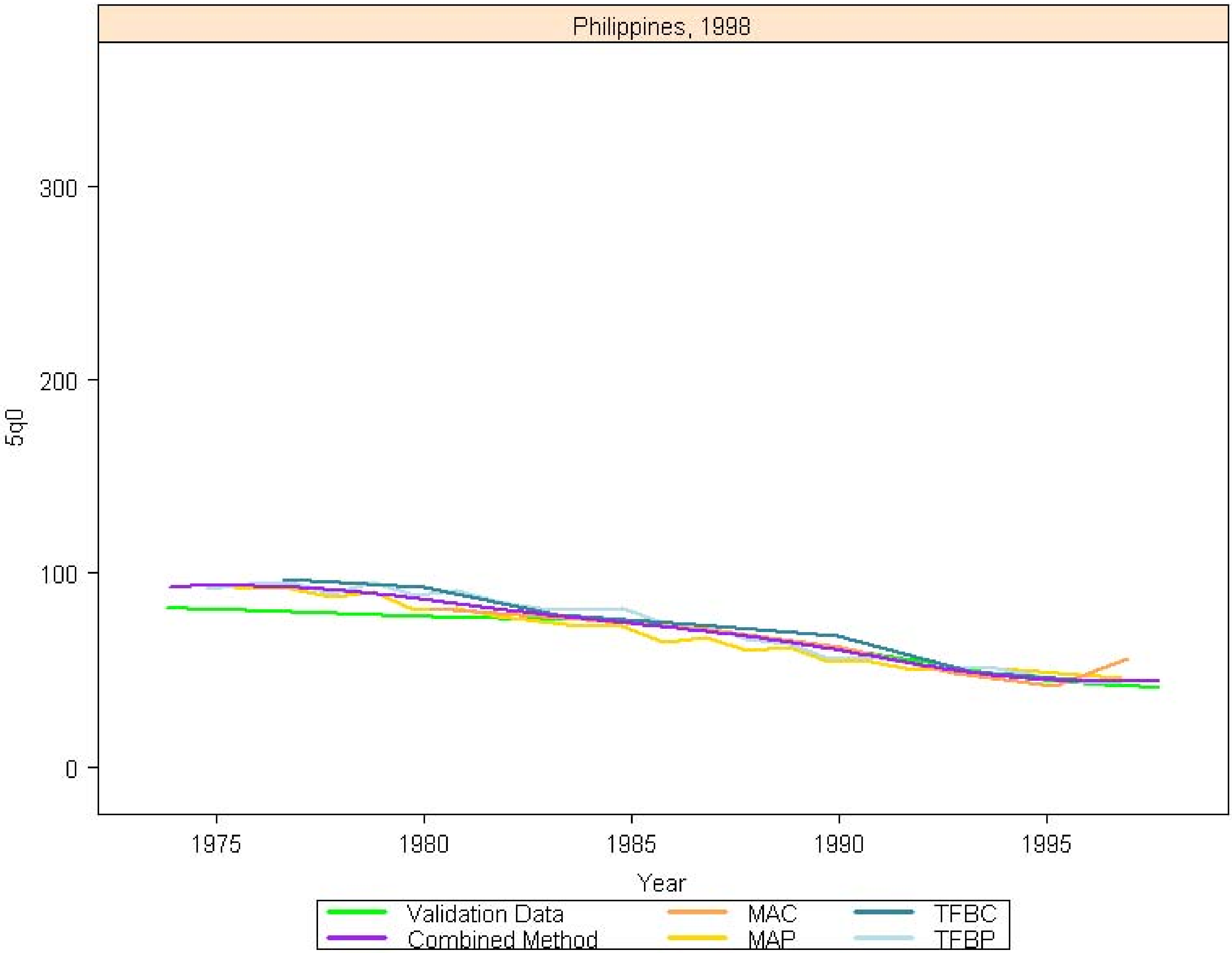

Philippines, 1998
5q0
0
100
200
300
1975
1980
1985
1990
1995
Year
Validation Data
Combined Method
MAC
MAP
TFBC
TFBP

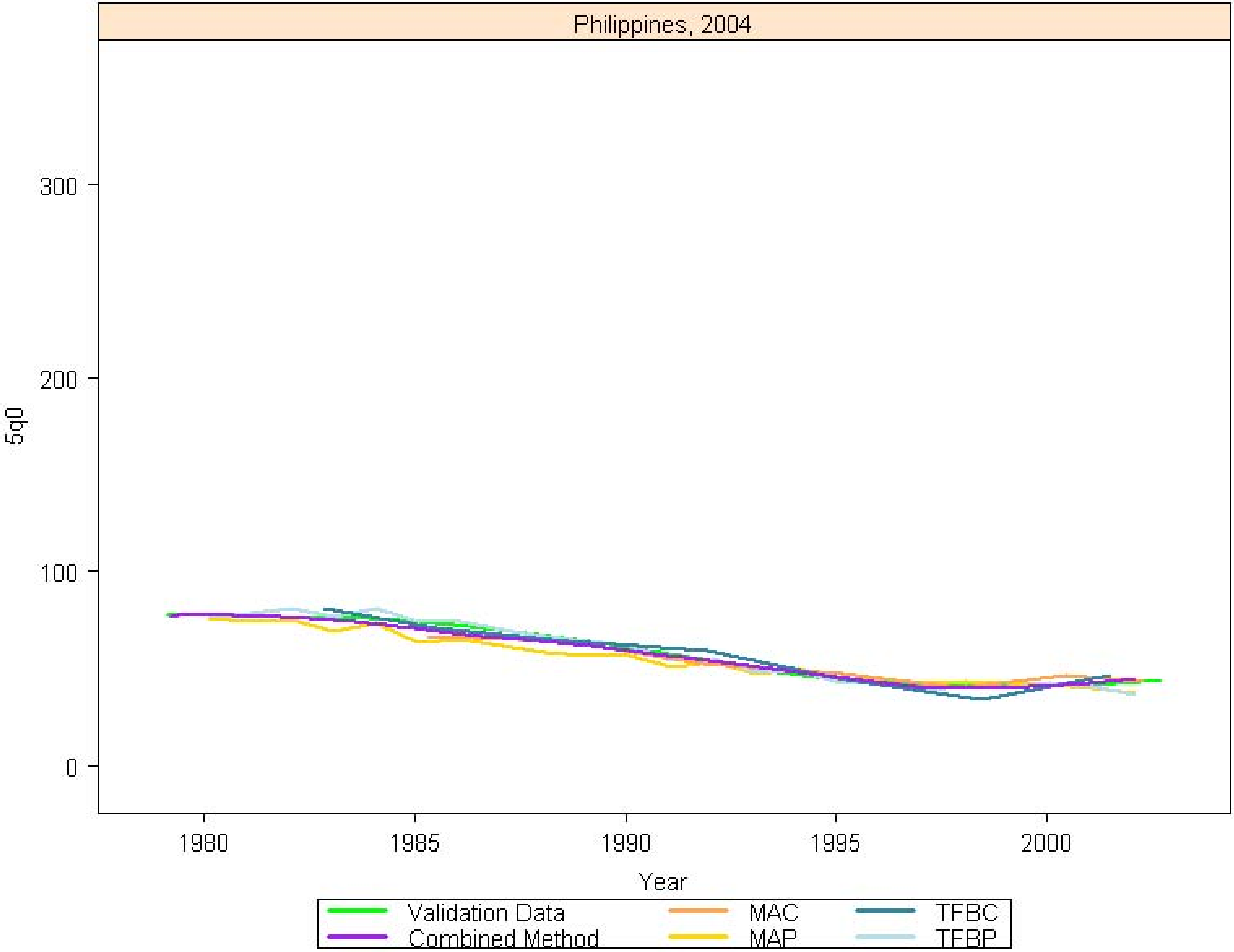

Philippines, 2004
300
200
100
0
5q0
1980
1985
1990
1995
2000
Year
Validation Data
Combined Method
MAC
MAP
TFBC
TFBP

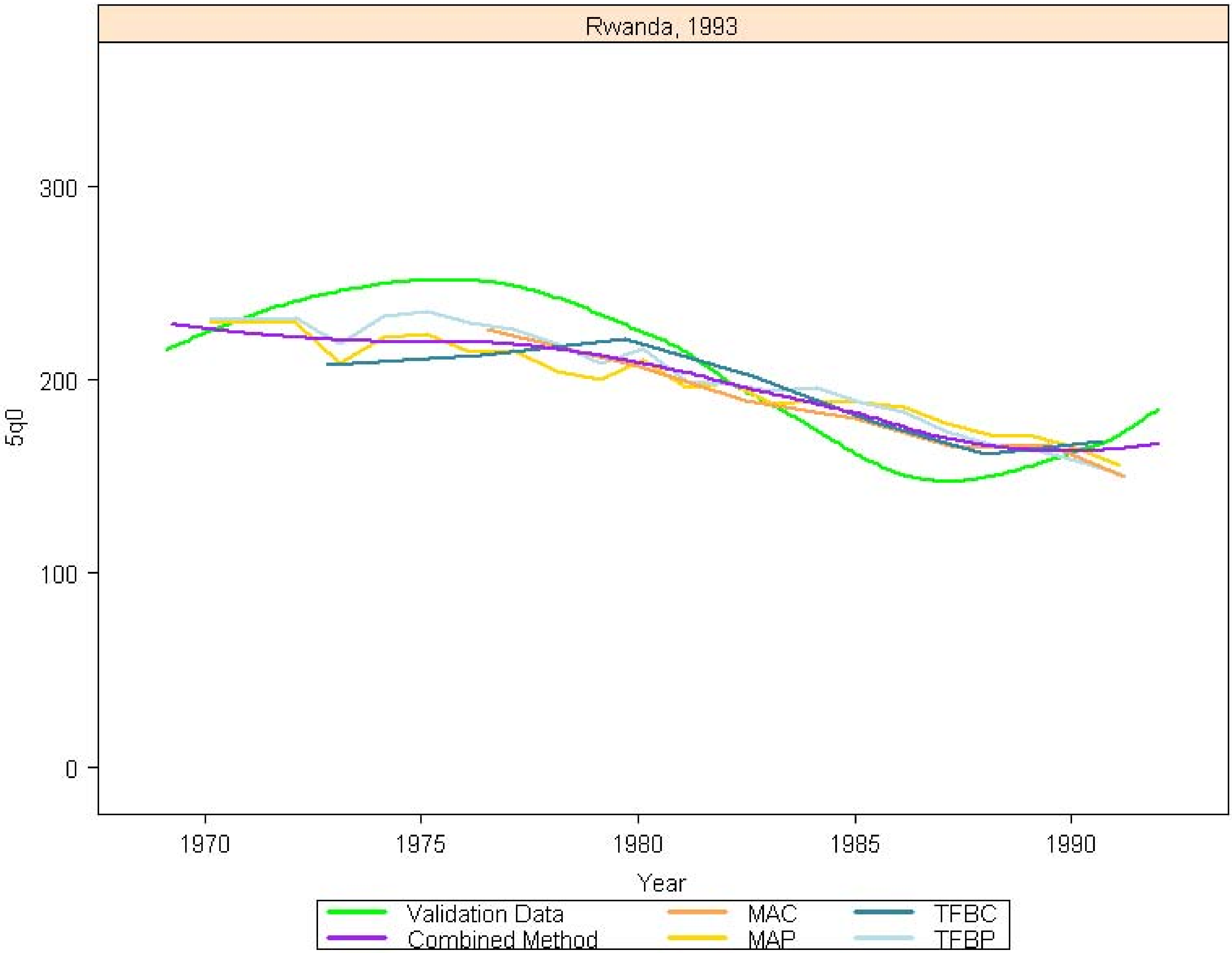
Rwanda, 1993
300
200
100
0
5q0
1970
1975
1980
1985
1990
Year
Validation Data
Combined Method
MAC
MAP
TFBC
TFBP

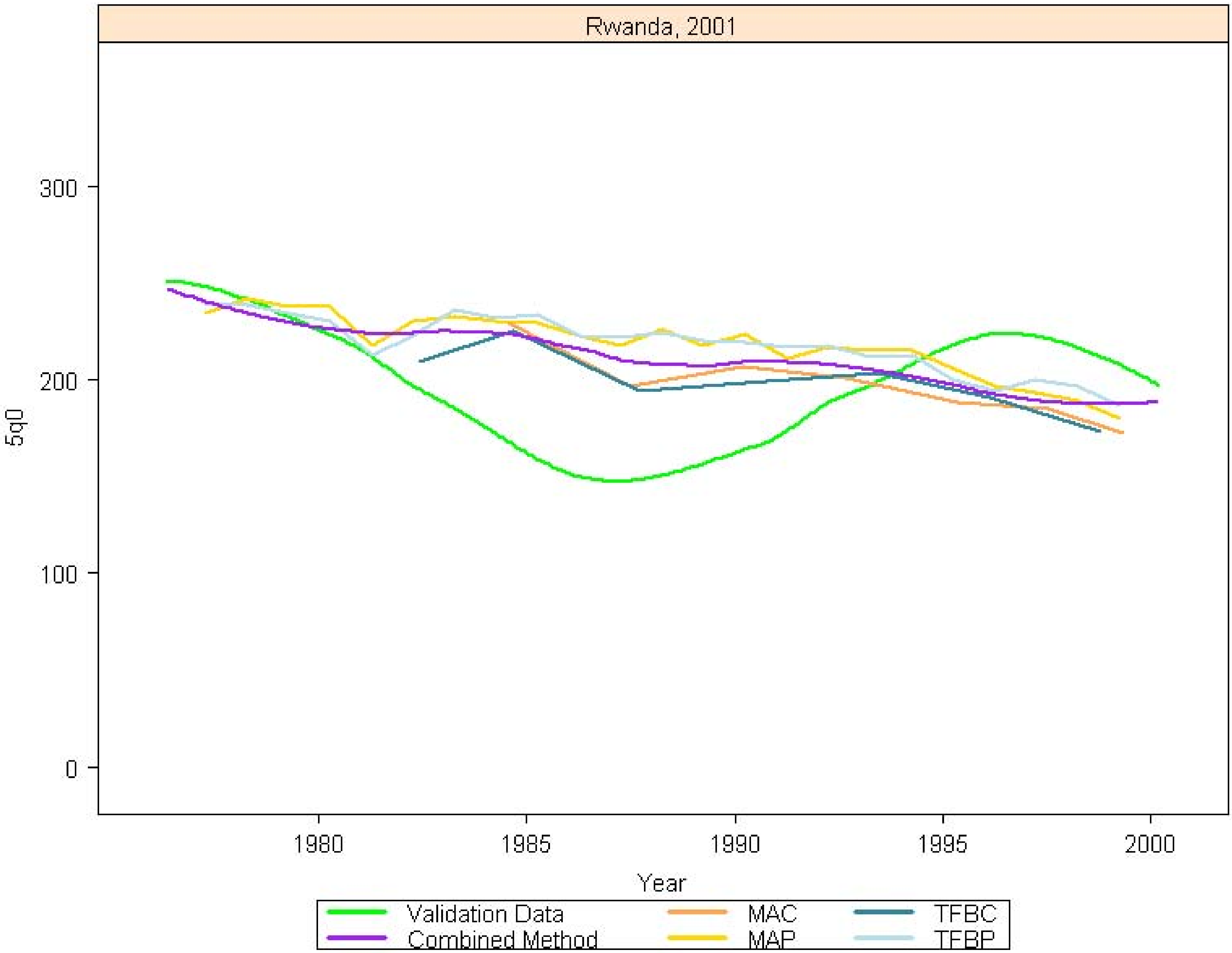
Rwanda, 2001
300
200
100
0
5q0
1980
1985
1990
1995
2000
Year
Validation Data
Combined Method
MAC
MAP
TFBC
TFBP

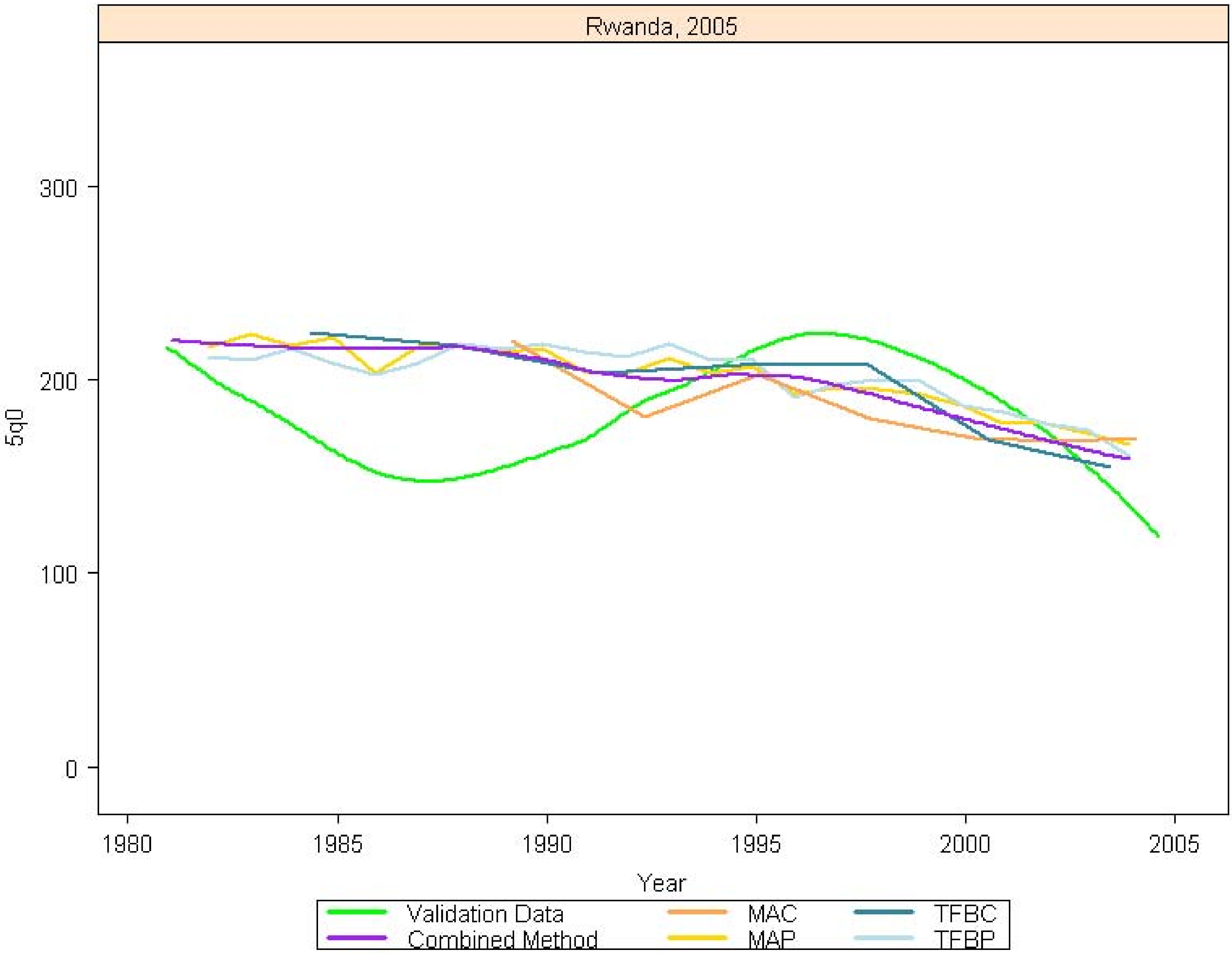

Rwanda, 2005
300
200
100
0
5q0
1980
1985
1990
1995
2000
2005
Year
Validation Data
Combined Method
MAC
MAP
TFBC
TFBP

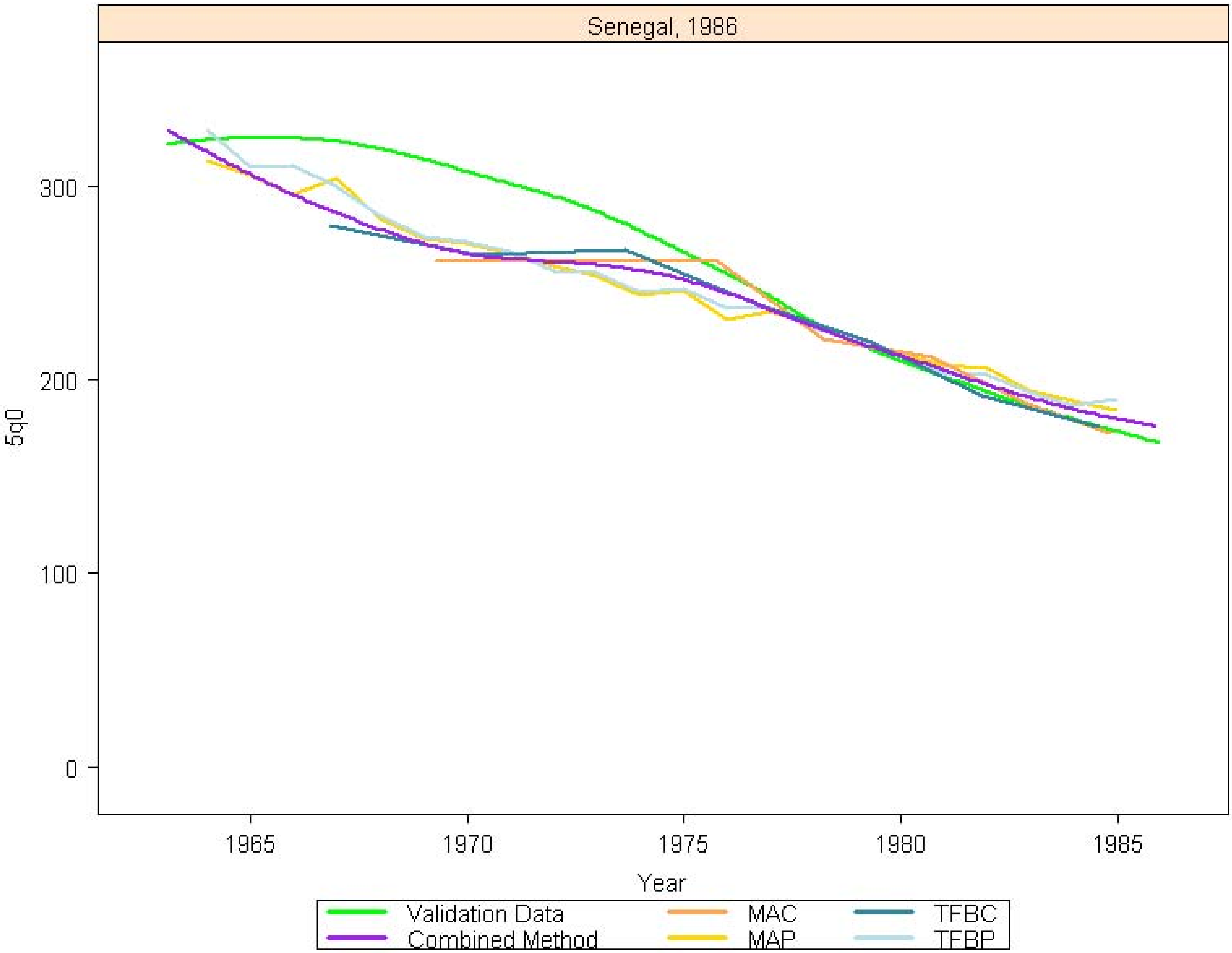
Senegal, 1986
5q0
300
200
100
0
1965
1970
1975
1980
1985
Year
Validation Data
Combined Method
MAC
MAP
TFBC
TFBP

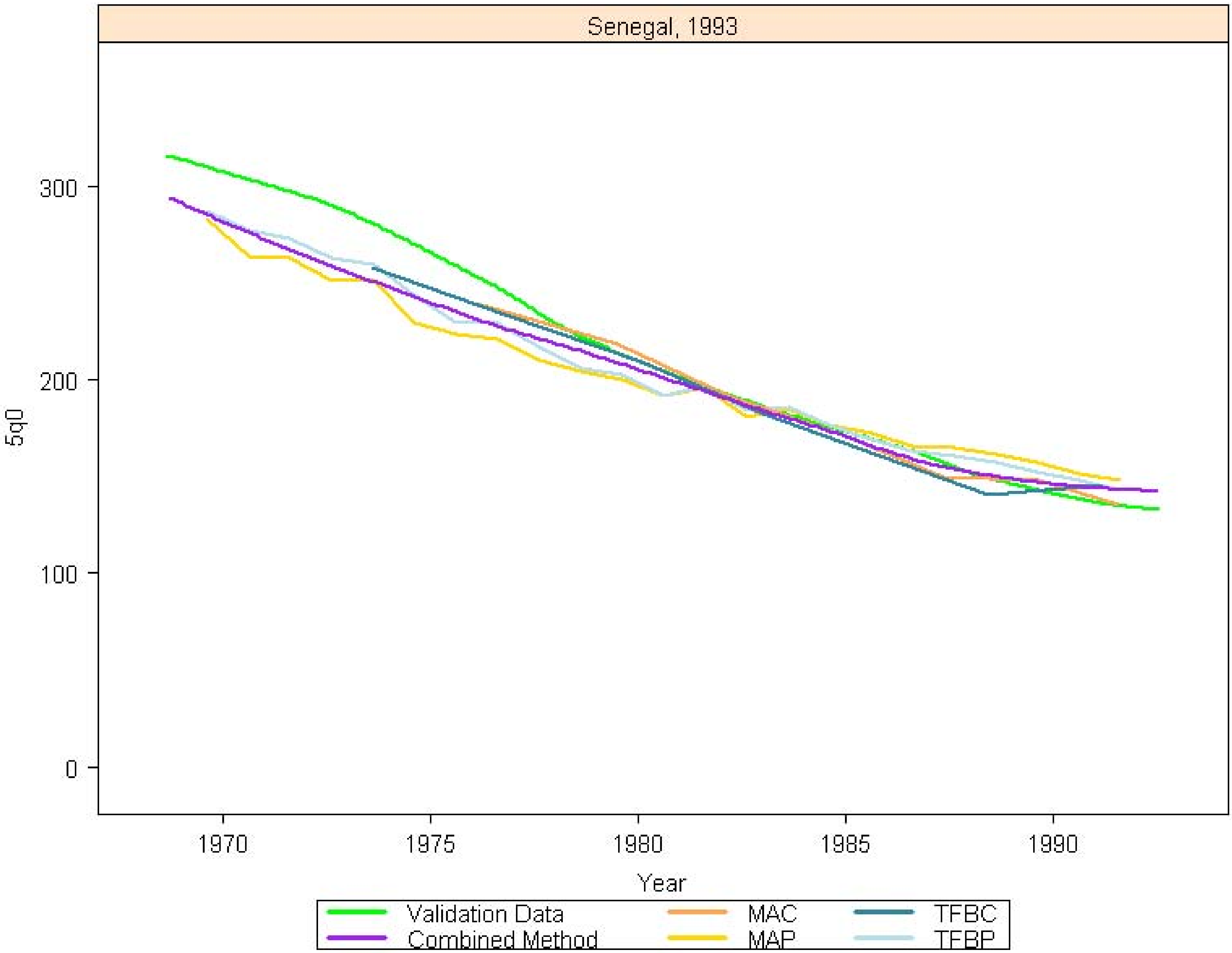

Senegal, 1993
5q0
300
200
100
0
1970
1975
1980
1985
1990
Year
Validation Data
Combined Method
MAC
MAP
TFBC
TFBP

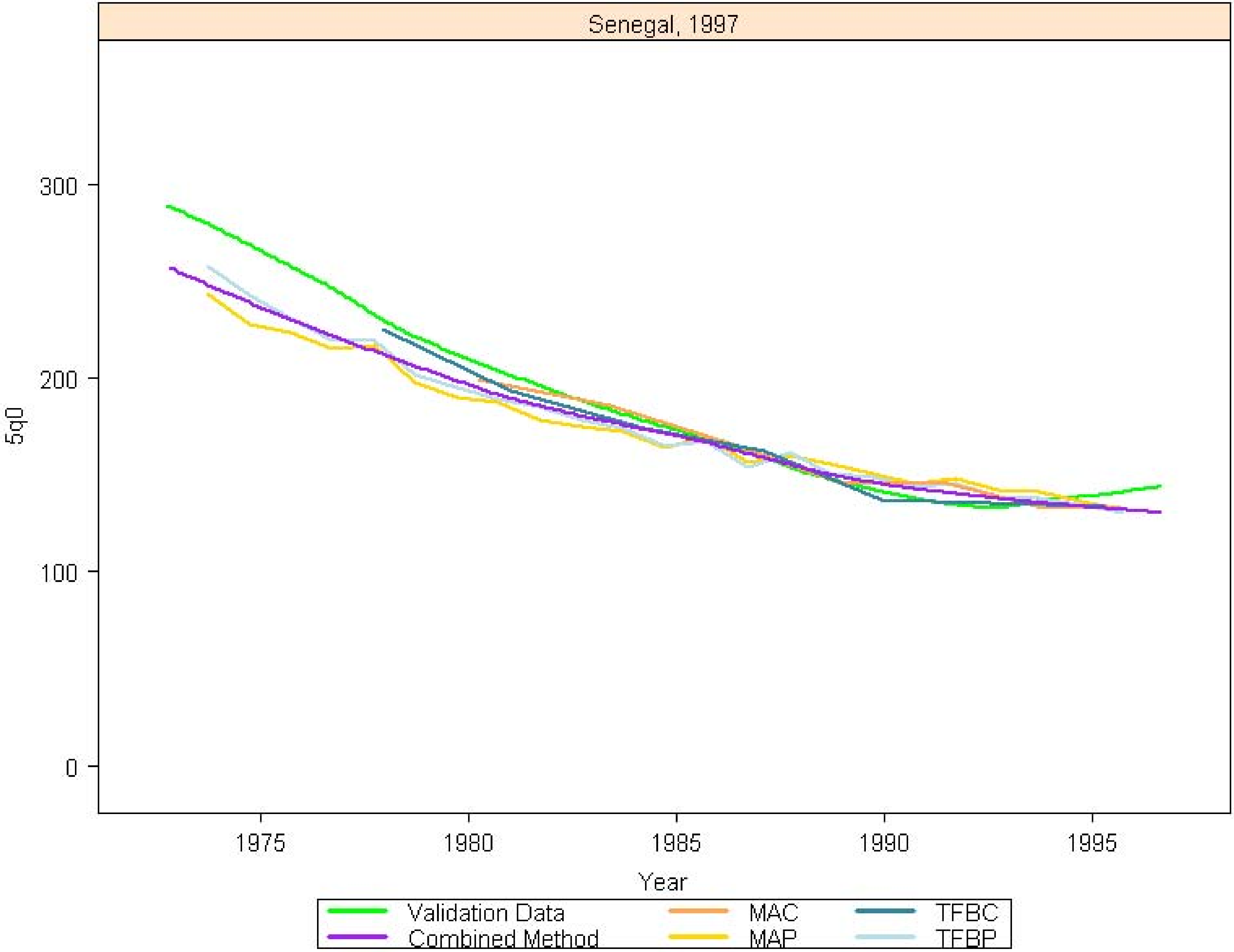

Senegal, 1997
300
200
100
0
5q0
1975
1980
1985
1990
1995
Year
Validation Data
Combined Method
MAC
MAP
TFBC
TFBP

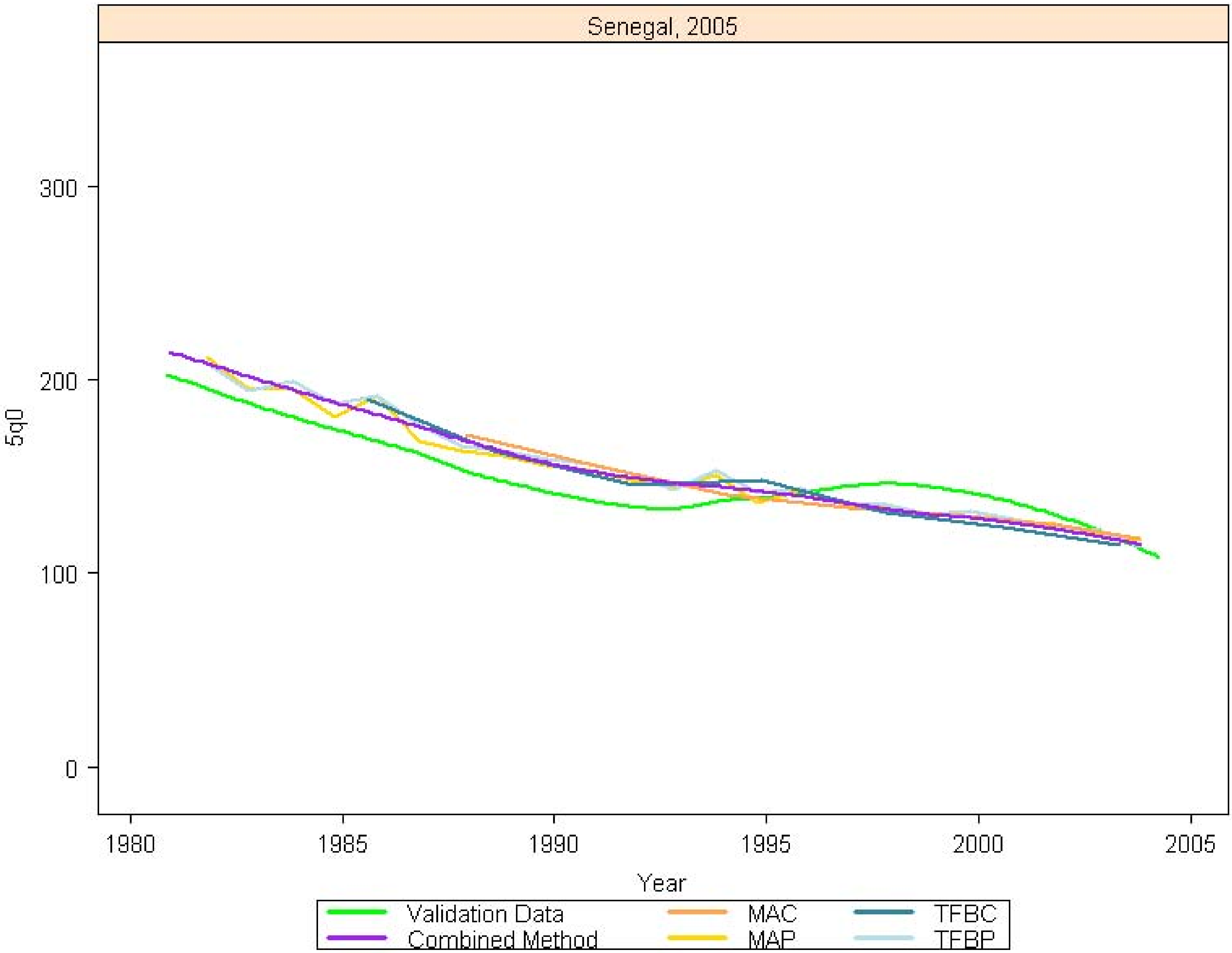
Senegal, 2005
5q0
300
200
100
0
1980
1985
1990
1995
2000
2005
Year
Validation Data
Combined Method
MAC
MAP
TFBC
TFBP

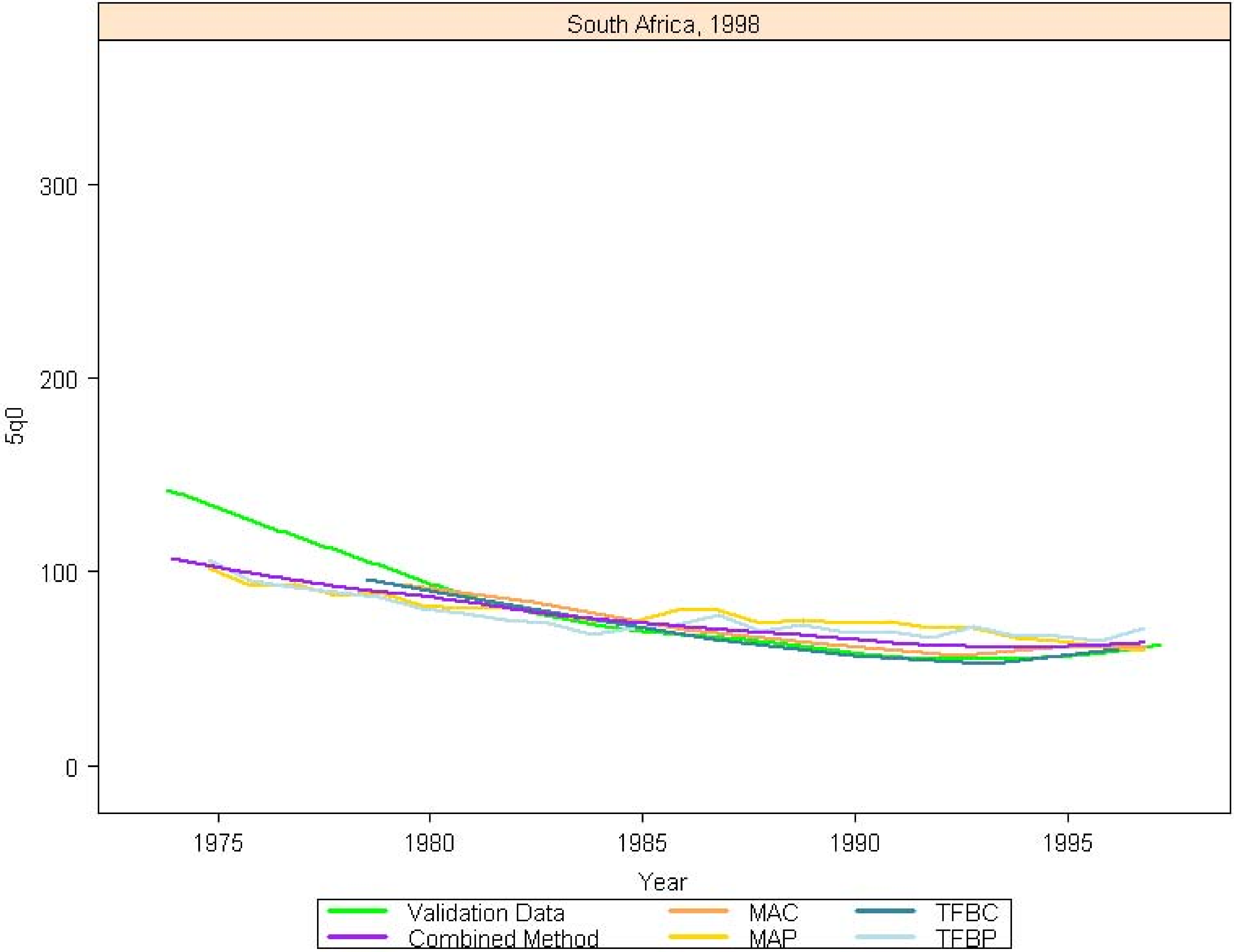
South Africa, 1998
300
200
100
0
5q0
1975
1980
1985
1990
1995
Year
Validation Data
Combined Method
MAC
MAP
TFBC
TFBP

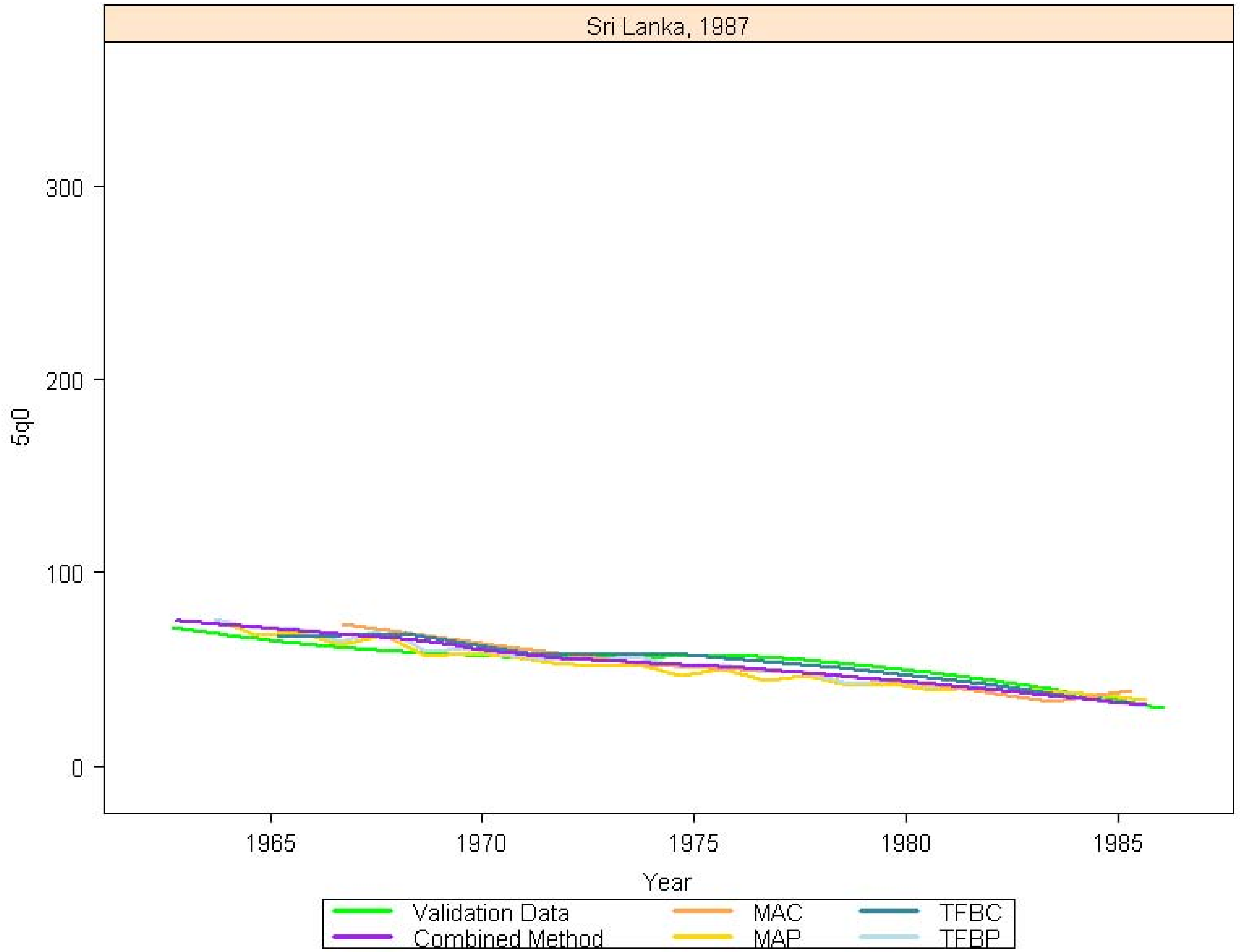

Sri Lanka, 1987
300
200
100
0
5q0
1965
1970
1975
1980
1985
Year
Validation Data
Combined Method
MAC
MAP
TFBC
TFBP

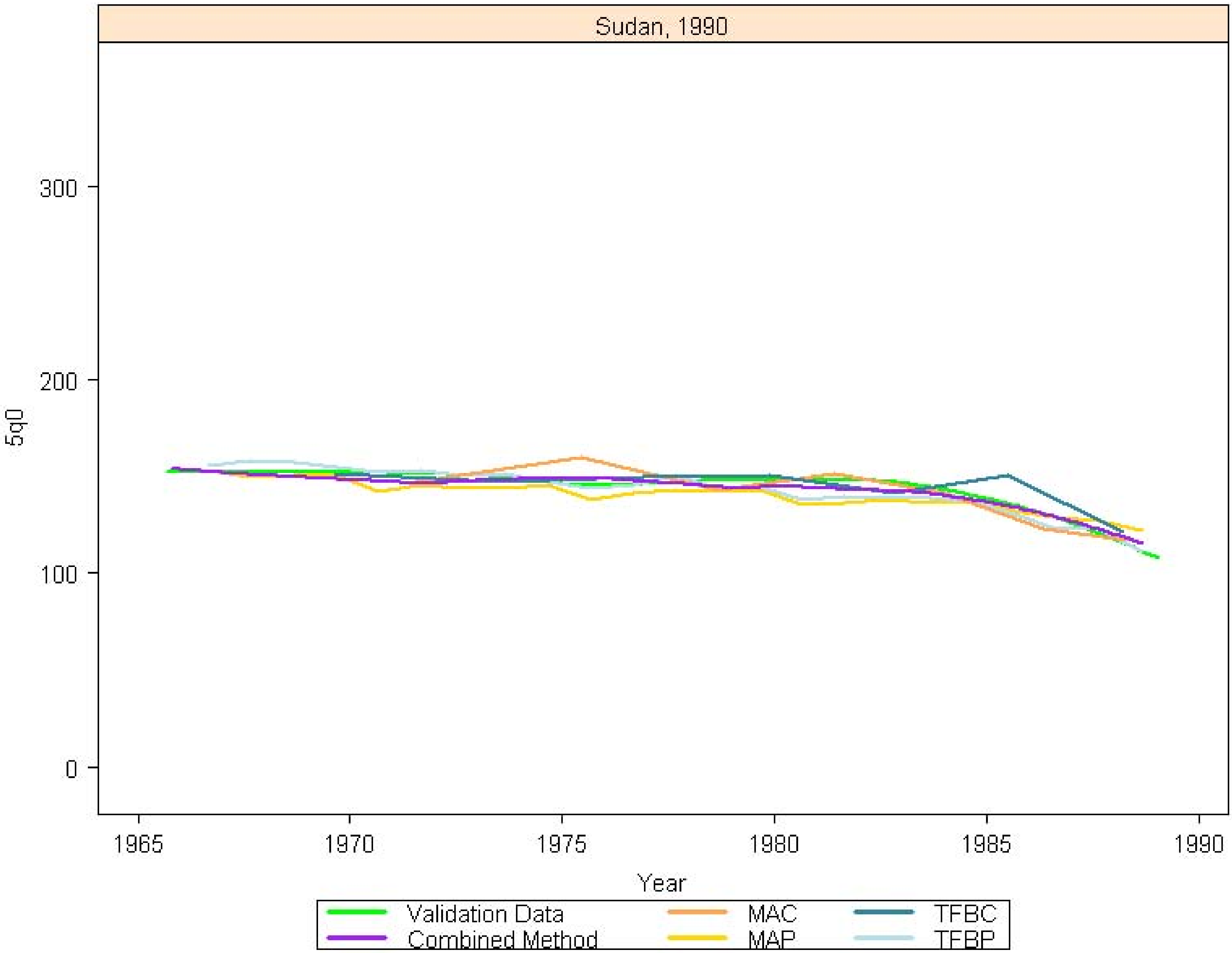
Sudan, 1990
300
200
100
0
5q0
1965
1970
1975
1980
1985
1990
Year
Validation Data
Combined Method
MAC
MAP
TFBC
TFBP

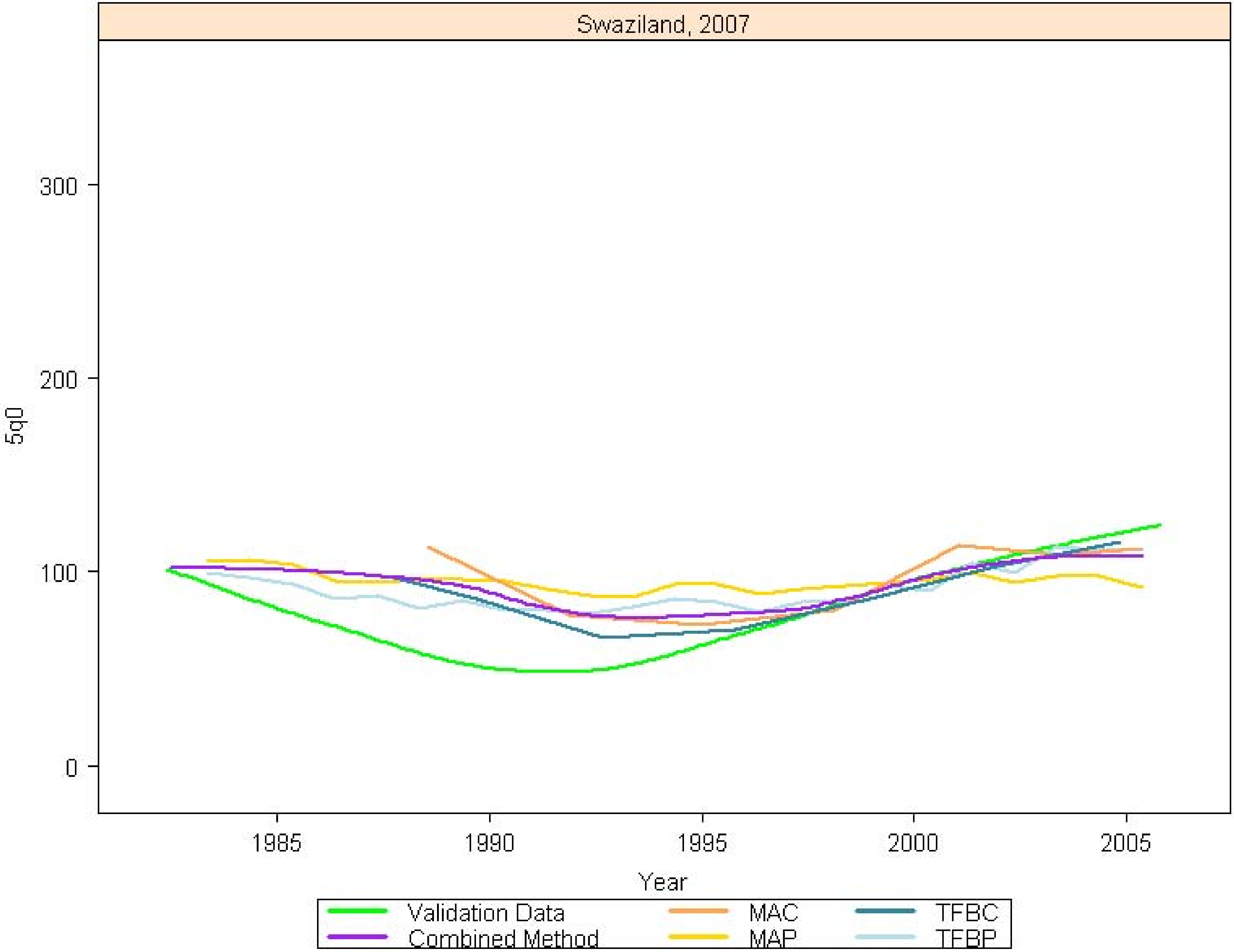

Swaziland, 2007
300
200
100
0
5q0
1985
1990
1995
2000
2005
Year
Validation Data
Combined Method
MAC
MAP
TFBC
TFBP

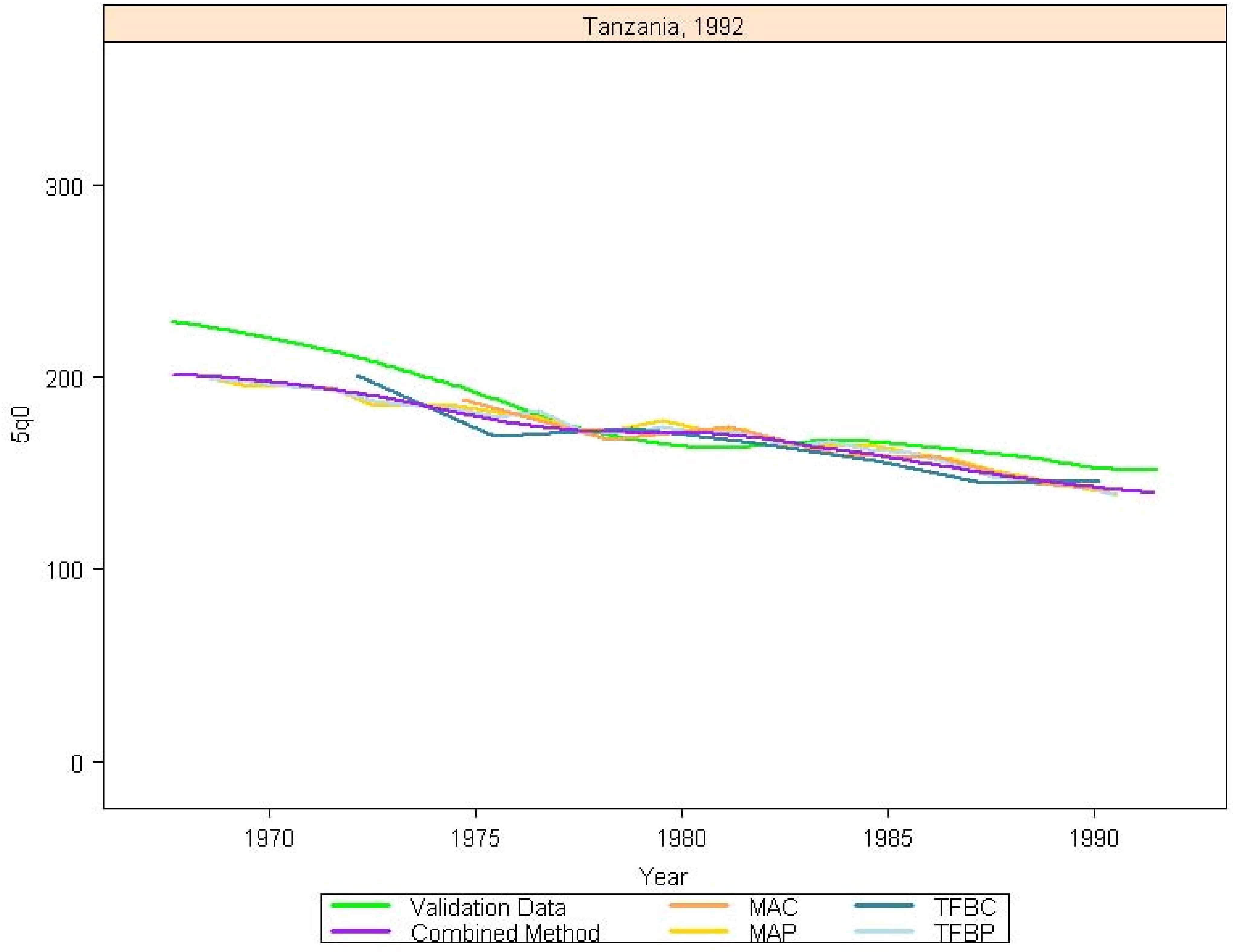
Tanzania, 1992
300
200
100
0
5q0
1970
1975
1980
1985
1990
Year
Validation Data
Combined Method
MAC
MAP
TFBC
TFBP

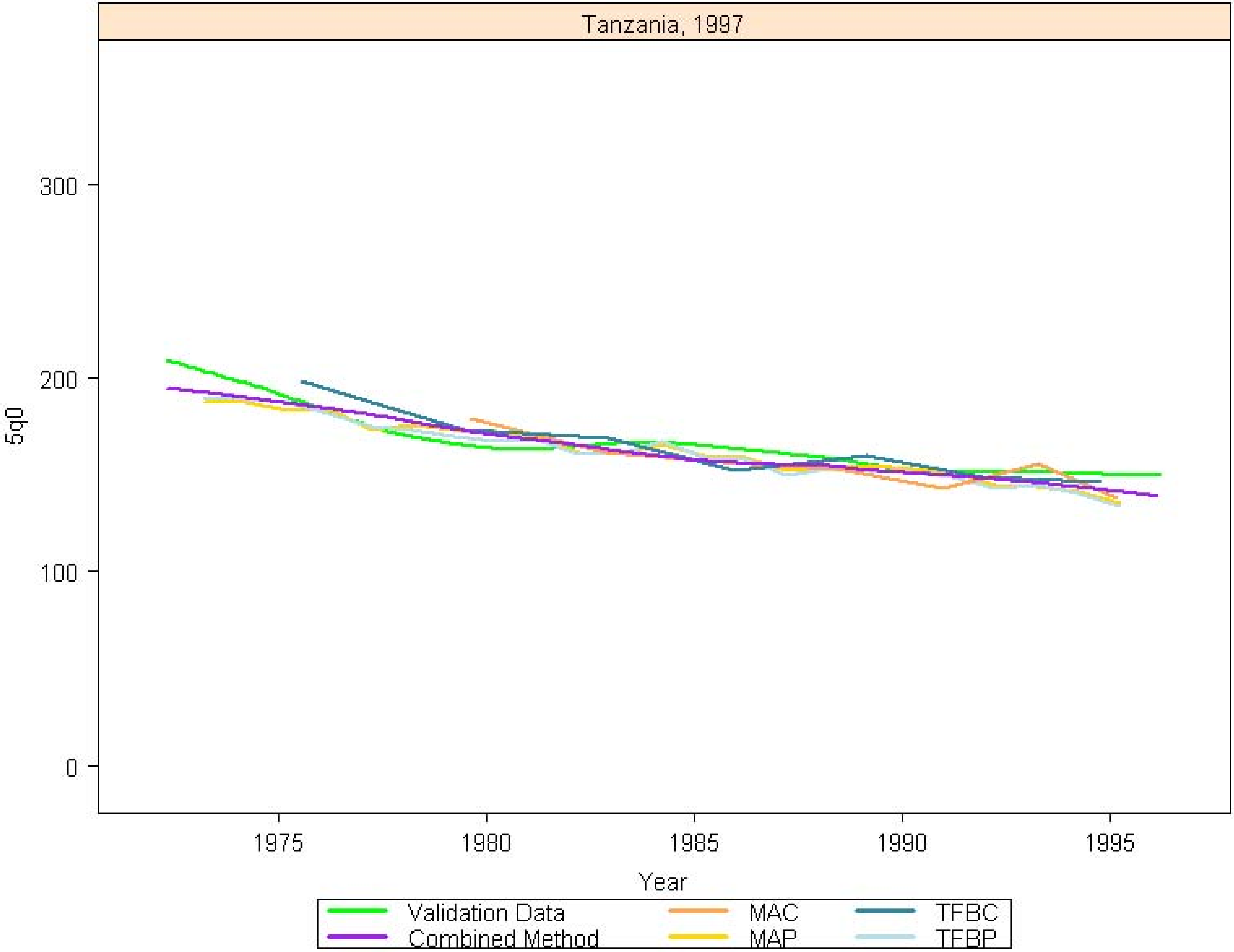

Tanzania, 1997
5q0
300
200
100
0
1975
1980
1985
1990
1995
Year
Validation Data
Combined Method
MAC
MAP
TFBC
TFBP

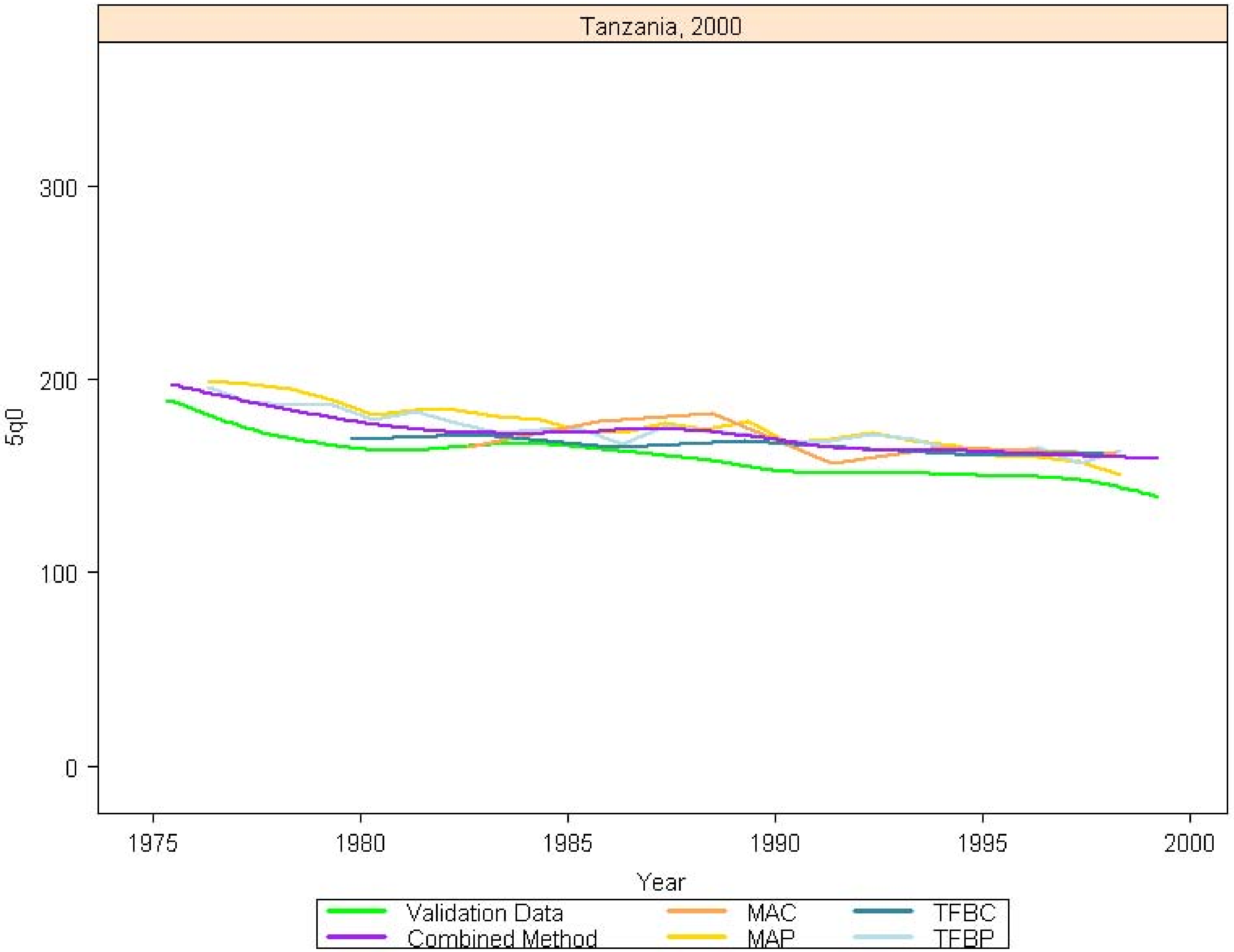
Tanzania, 2000
300
200
100
0
5q0
1975
1980
1985
1990
1995
2000
Year
Validation Data
Combined Method
MAC
MAP
TFBC
TFBP

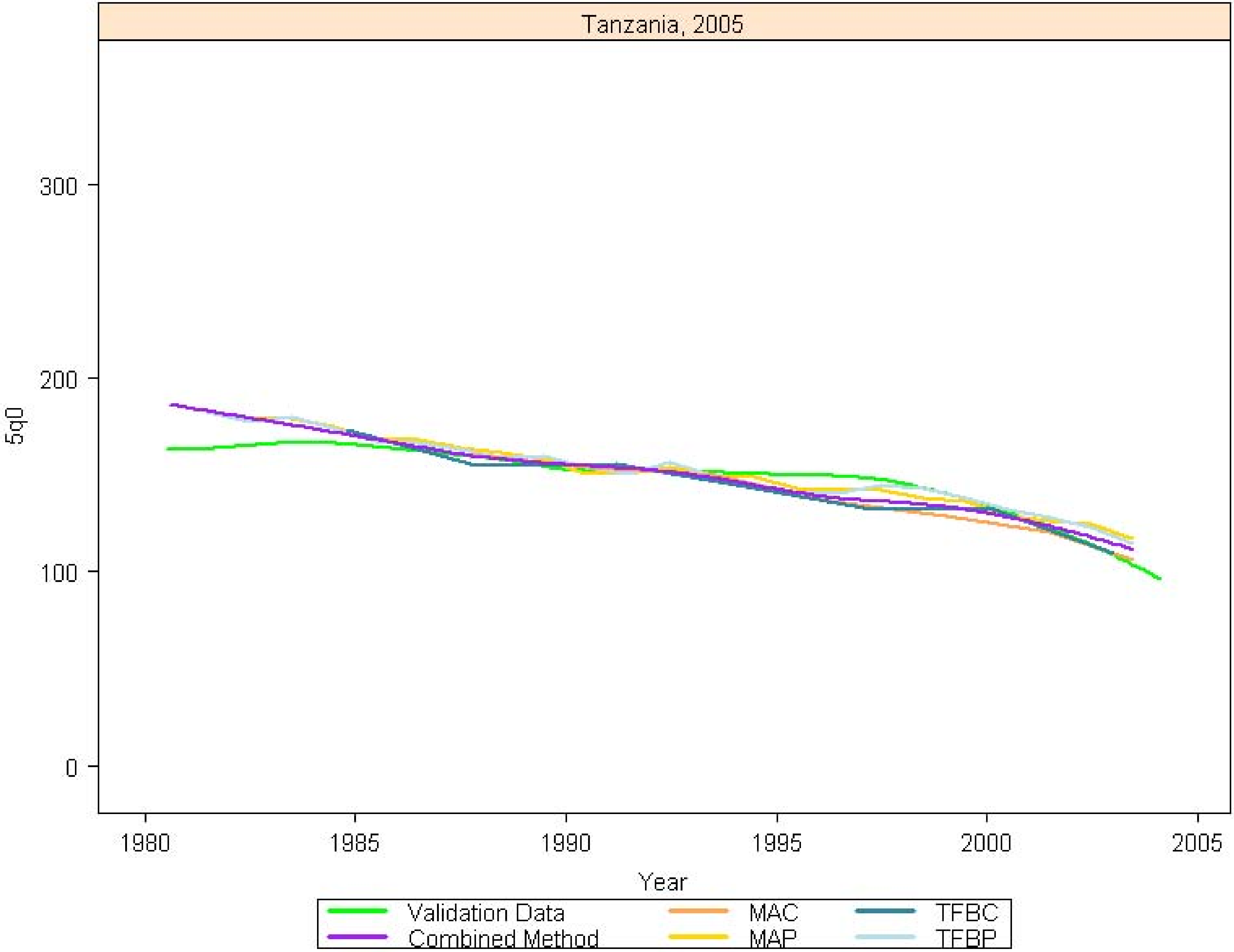

Tanzania, 2005
300
200
100
0
5q0
1980
1985
1990
1995
2000
2005
Year
Validation Data
Combined Method
MAC
MAP
TFBC
TFBP

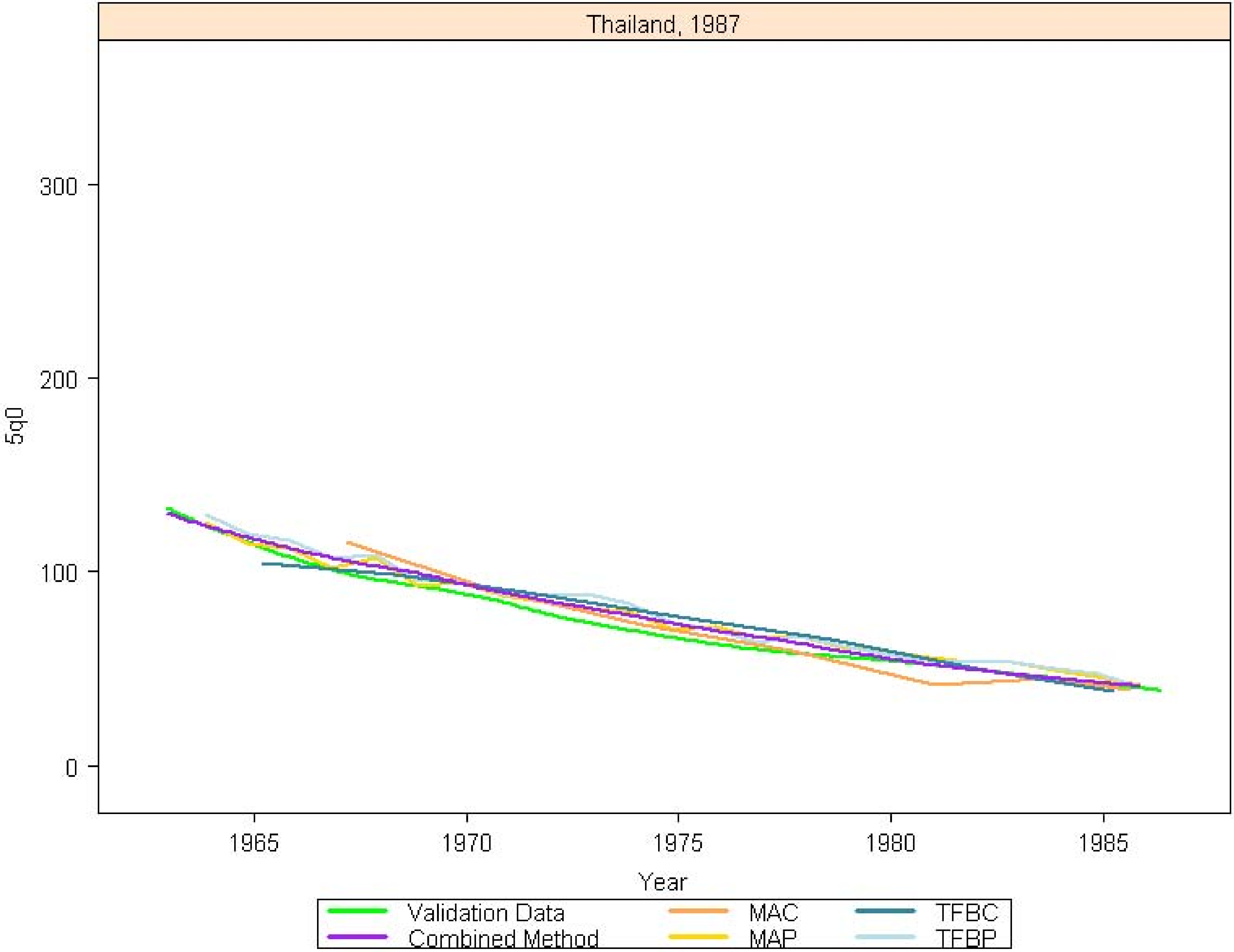

Thailand, 1987
300
200
100
0
5q0
1965
1970
1975
1980
1985
Year
Validation Data
Combined Method
MAC
MAP
TFBC
TFBP

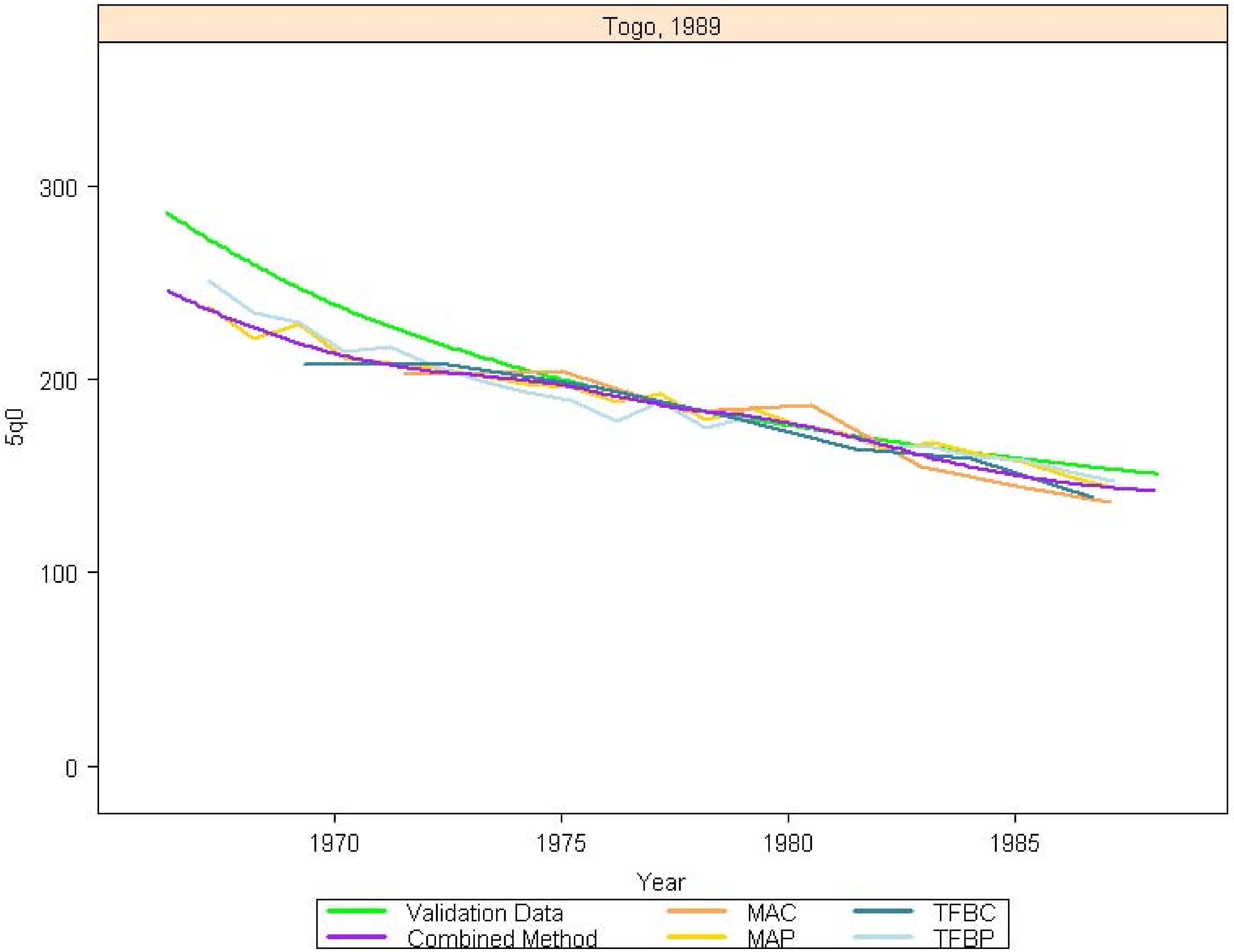

Togo, 1989
5q0
300
200
100
0
1970
1975
1980
1985
Year
Validation Data
Combined Method
MAC
MAP
TFBC
TFBP

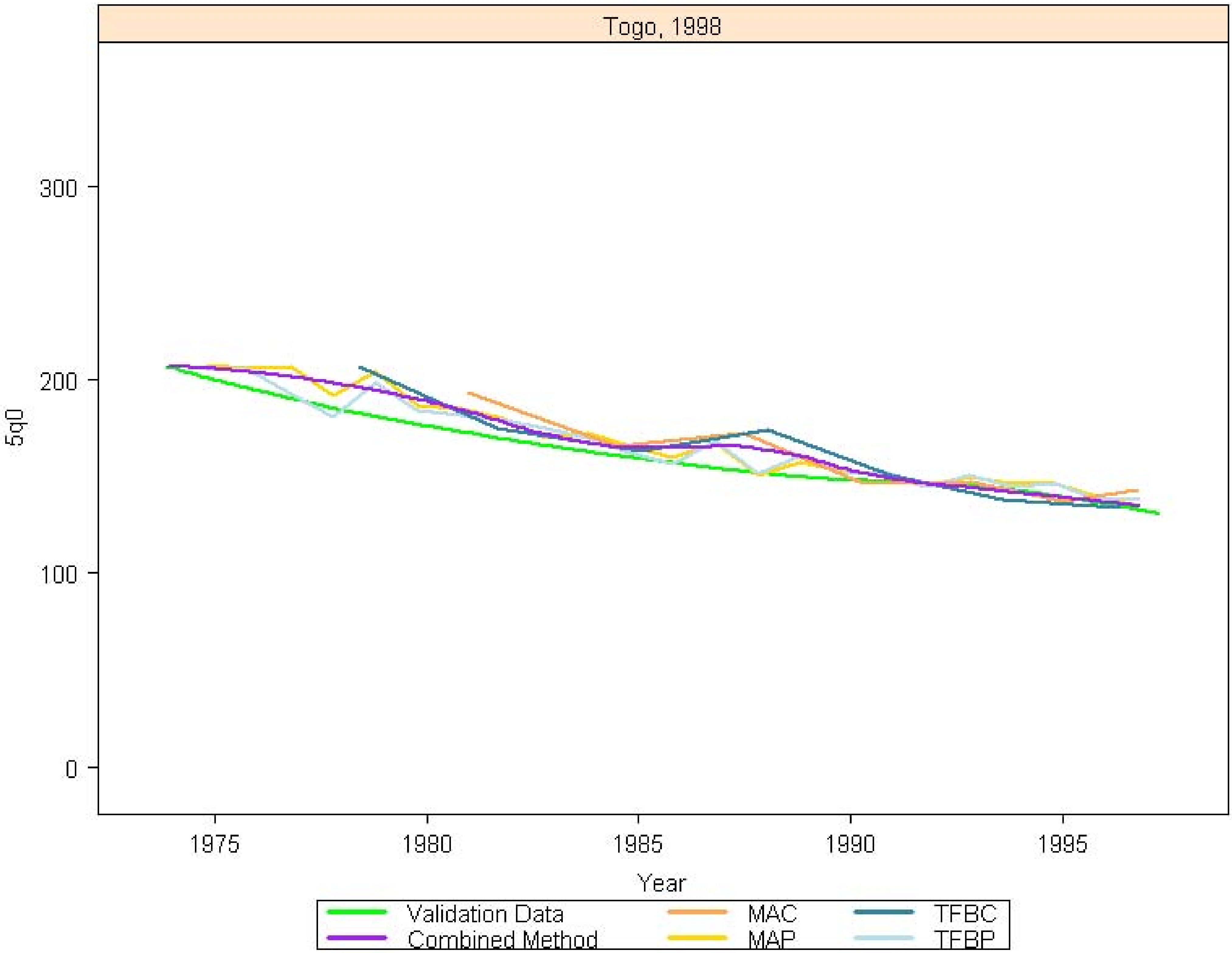

Togo, 1998
300
200
100
0
5q0
1975
1980
1985
1990
1995
Year
Validation Data
Combined Method
MAC
MAP
TFBC
TFBP

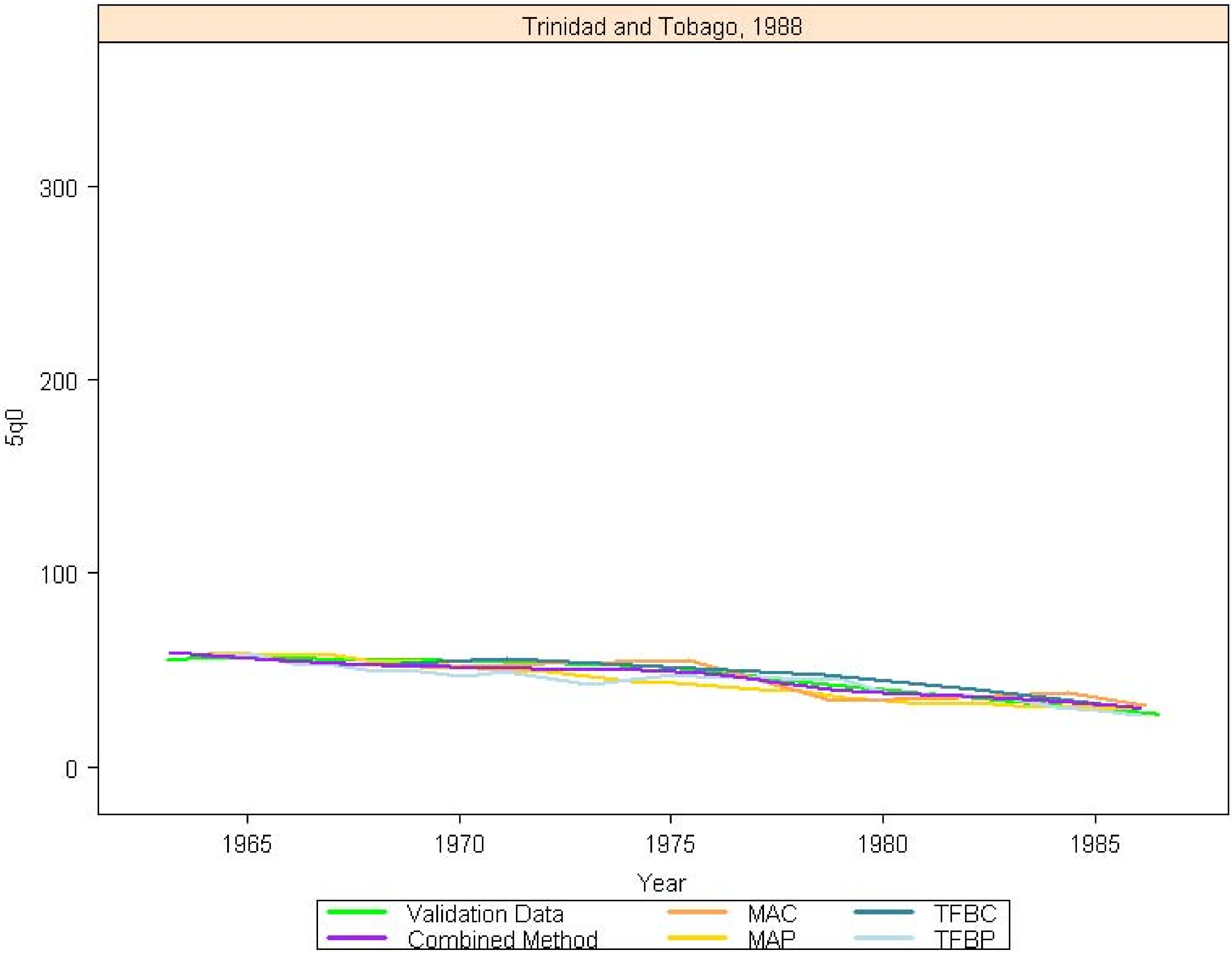

Trinidad and Tobago, 1988
300
200
100
0
5q0
1965
1970
1975
1980
1985
Year
Validation Data
Combined Method
MAC
MAP
TFBC
TFBP

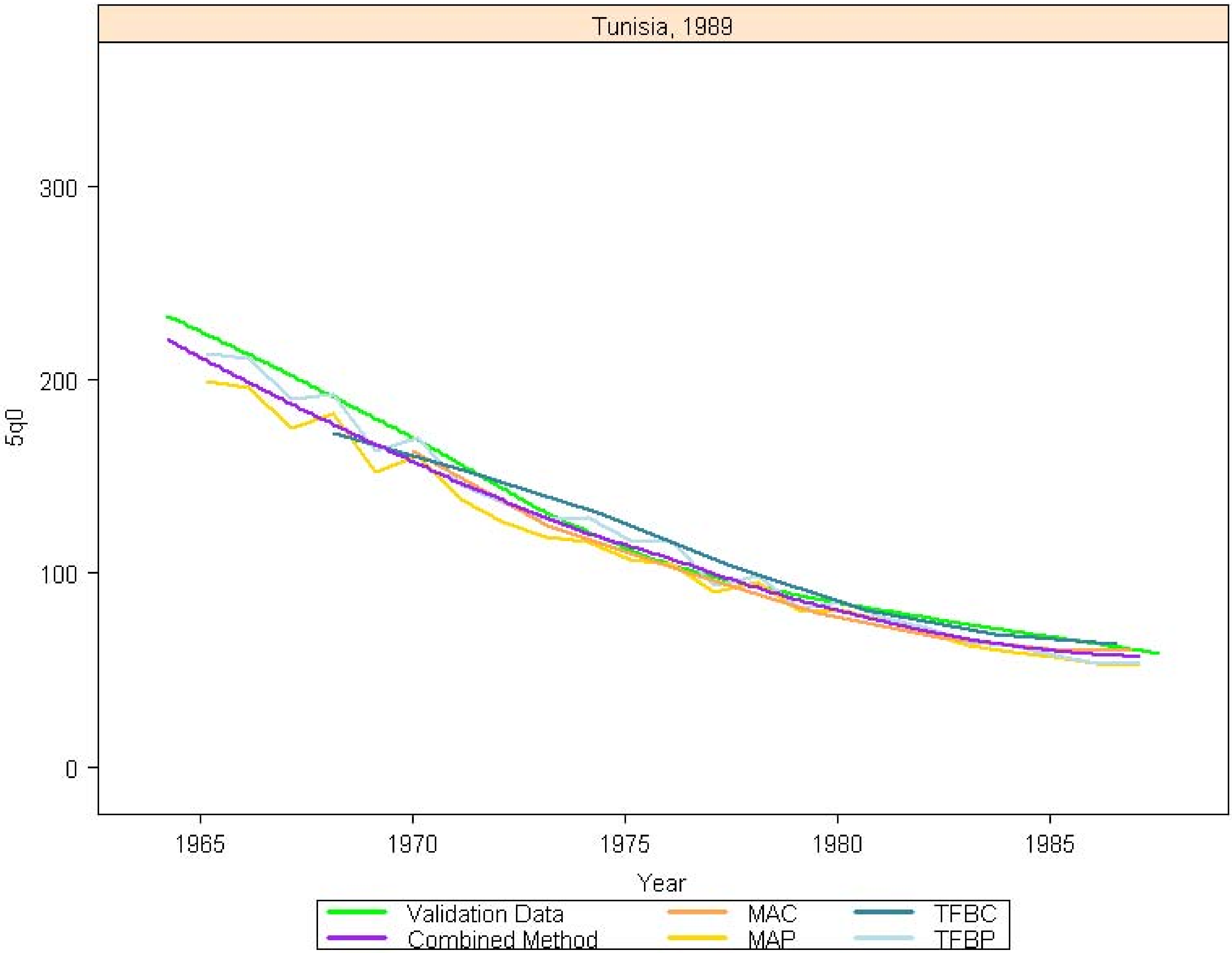

Tunisia, 1989
300
200
100
0
5q0
1965
1970
1975
1980
1985
Year
Validation Data
Combined Method
MAC
MAP
TFBC
TFBP

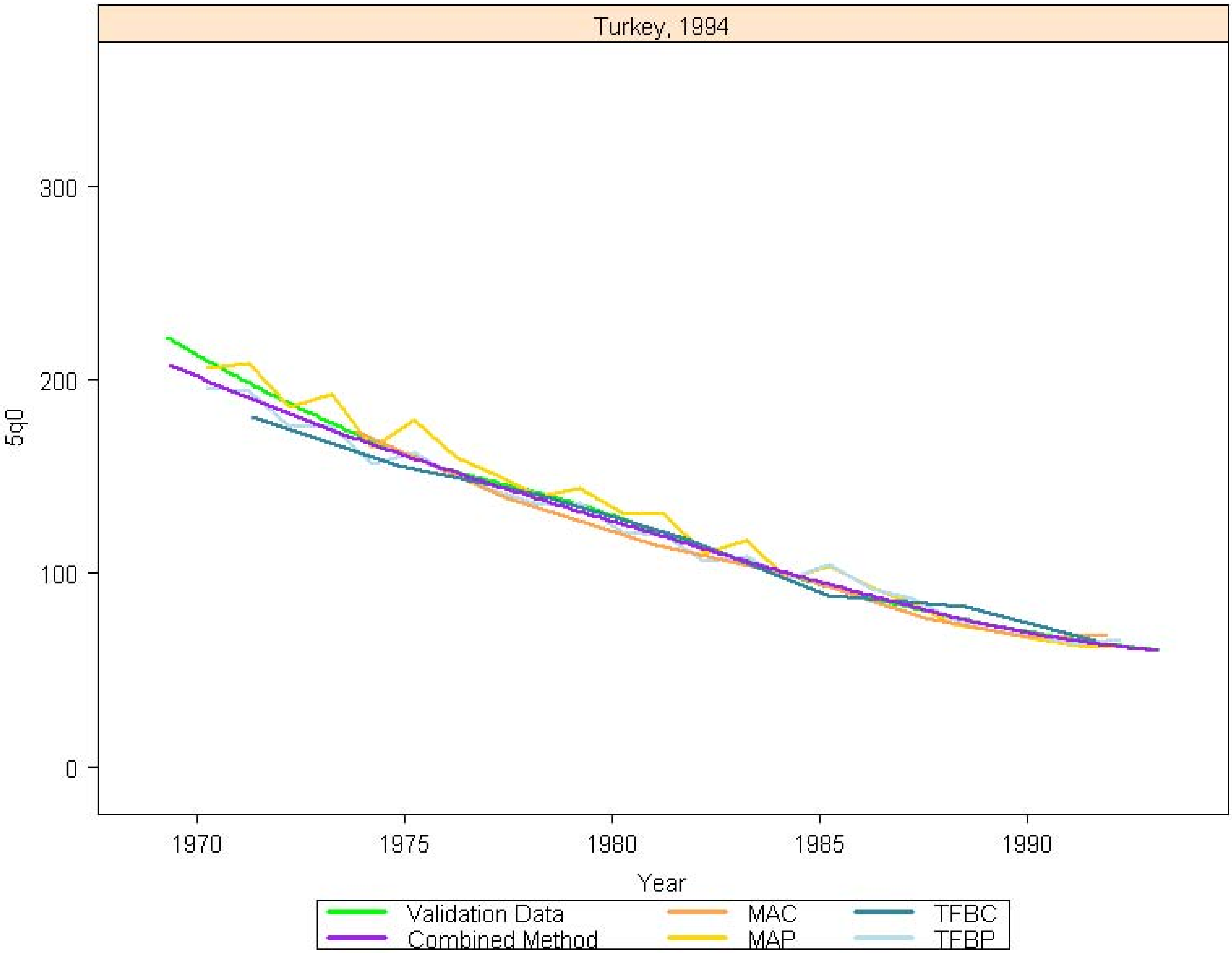

Turkey, 1994
300
200
100
0
5q0
1970
1975
1980
1985
1990
Year
Validation Data
Combined Method
MAC
MAP
TFBC
TFBP

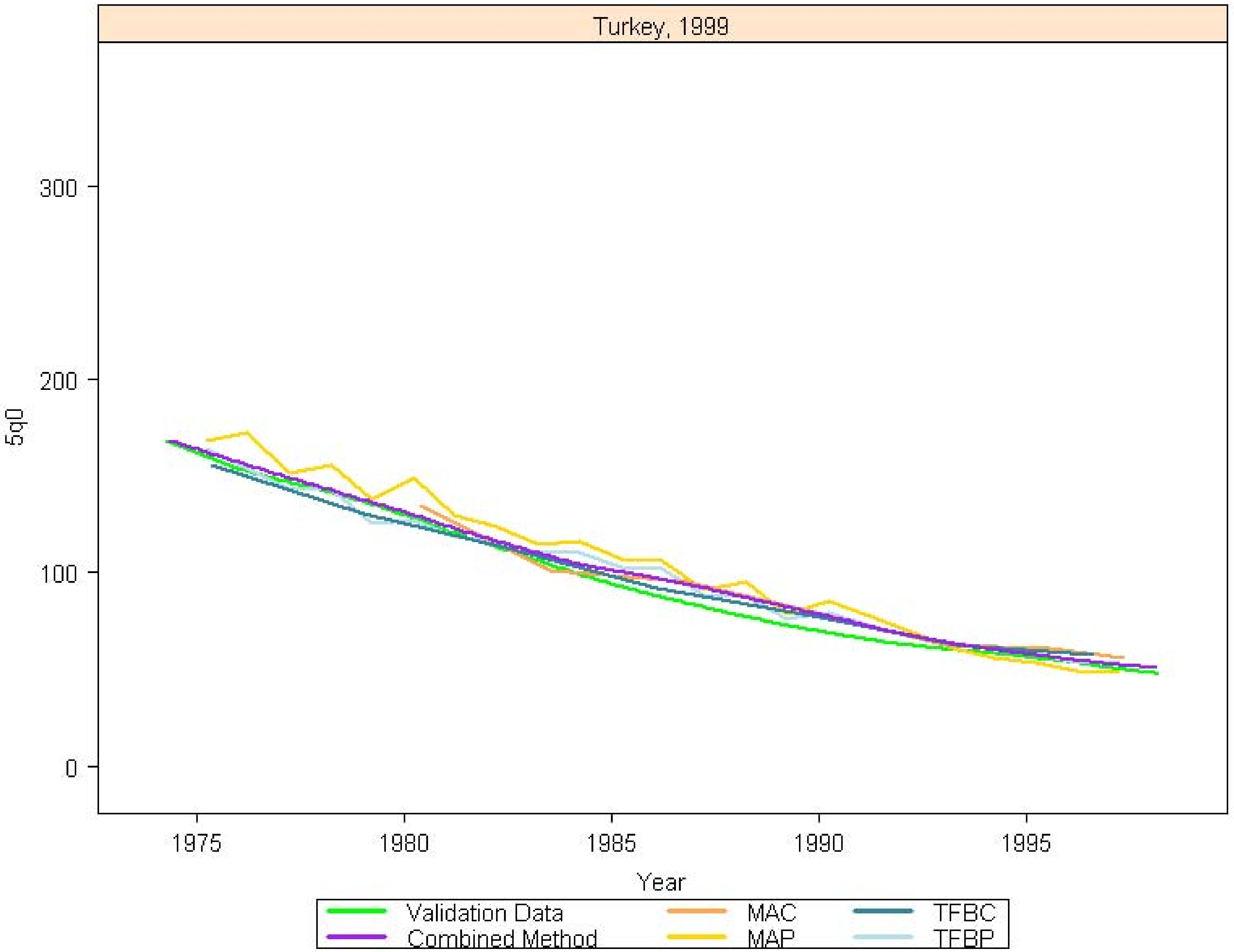

Turkey, 1999
300
200
100
0
5q0
1975
1980
1985
1990
1995
Year
Validation Data
Combined Method
MAC
MAP
TFBC
TFBP

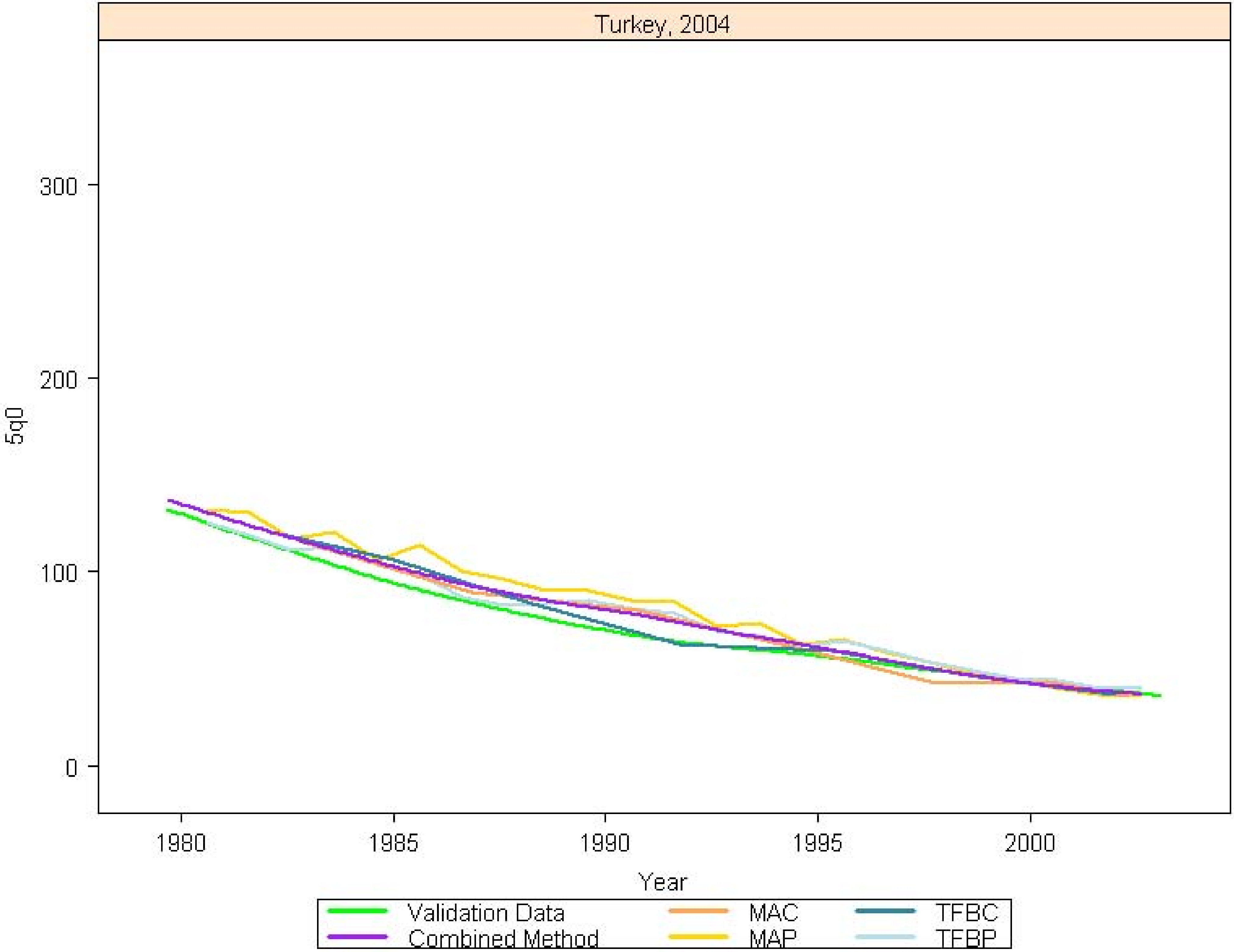
Turkey, 2004
5q0
300
200
100
0
1980
1985
1990
1995
2000
Year
Validation Data
Combined Method
MAC
MAP
TFBC
TFBP

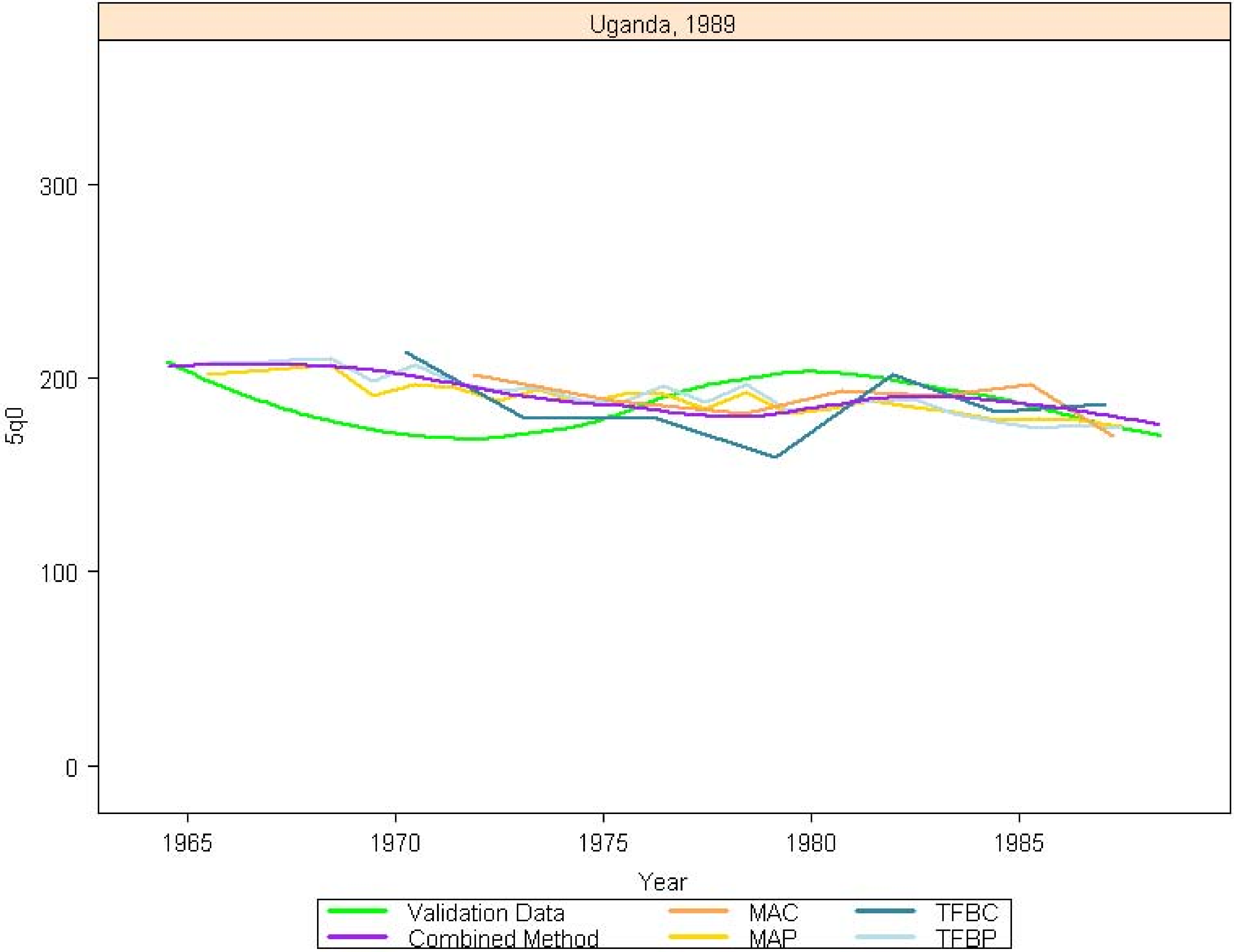
Uganda, 1989
300
200
100
0
5q0
1965
1970
1975
1980
1985
Year
Validation Data
Combined Method
MAC
MAP
TFBC
TFBP

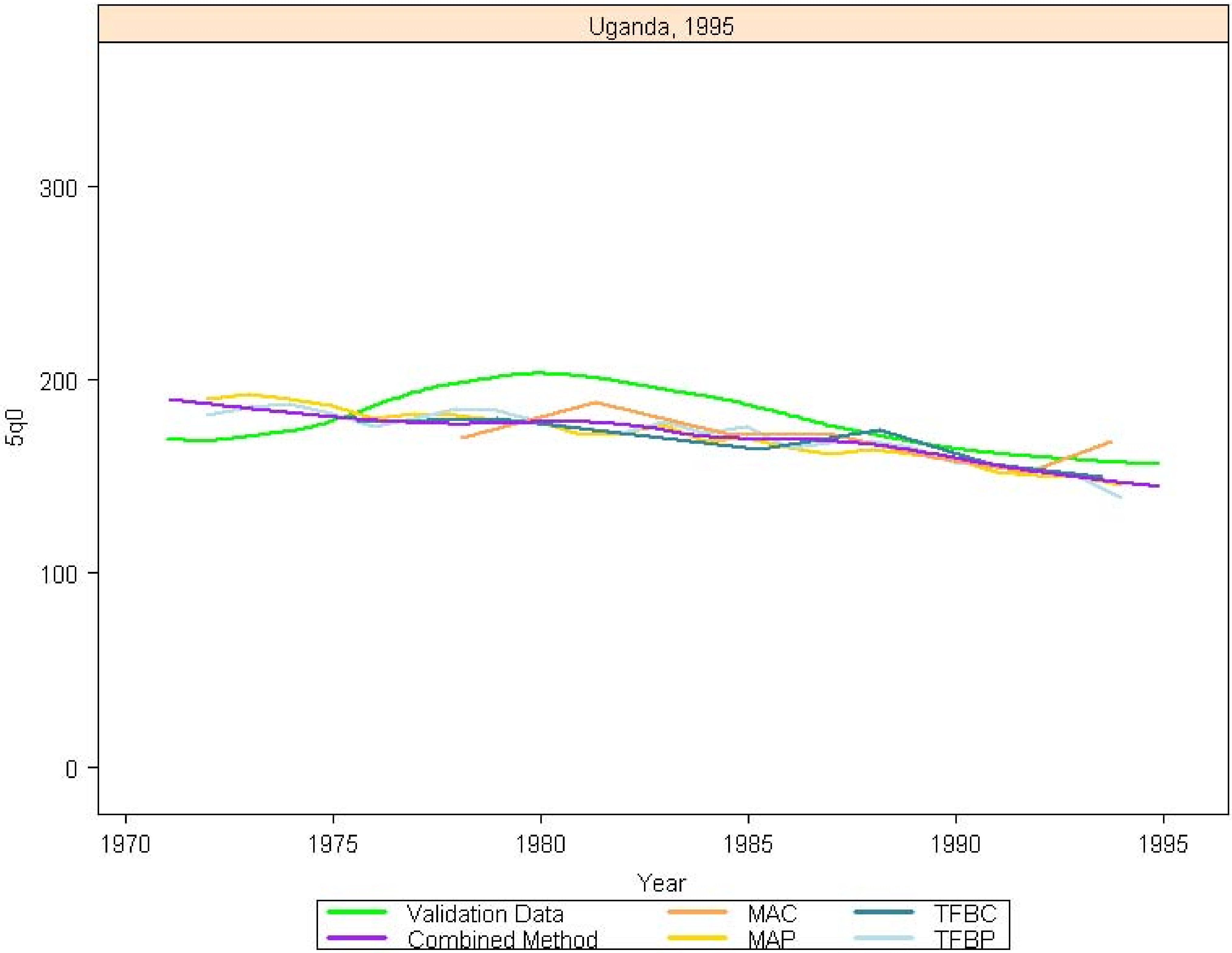

Uganda, 1995
300
200
100
0
5q0
1970
1975
1980
1985
1990
1995
Year
Validation Data
Combined Method
MAC
MAP
TFBC
TFBP

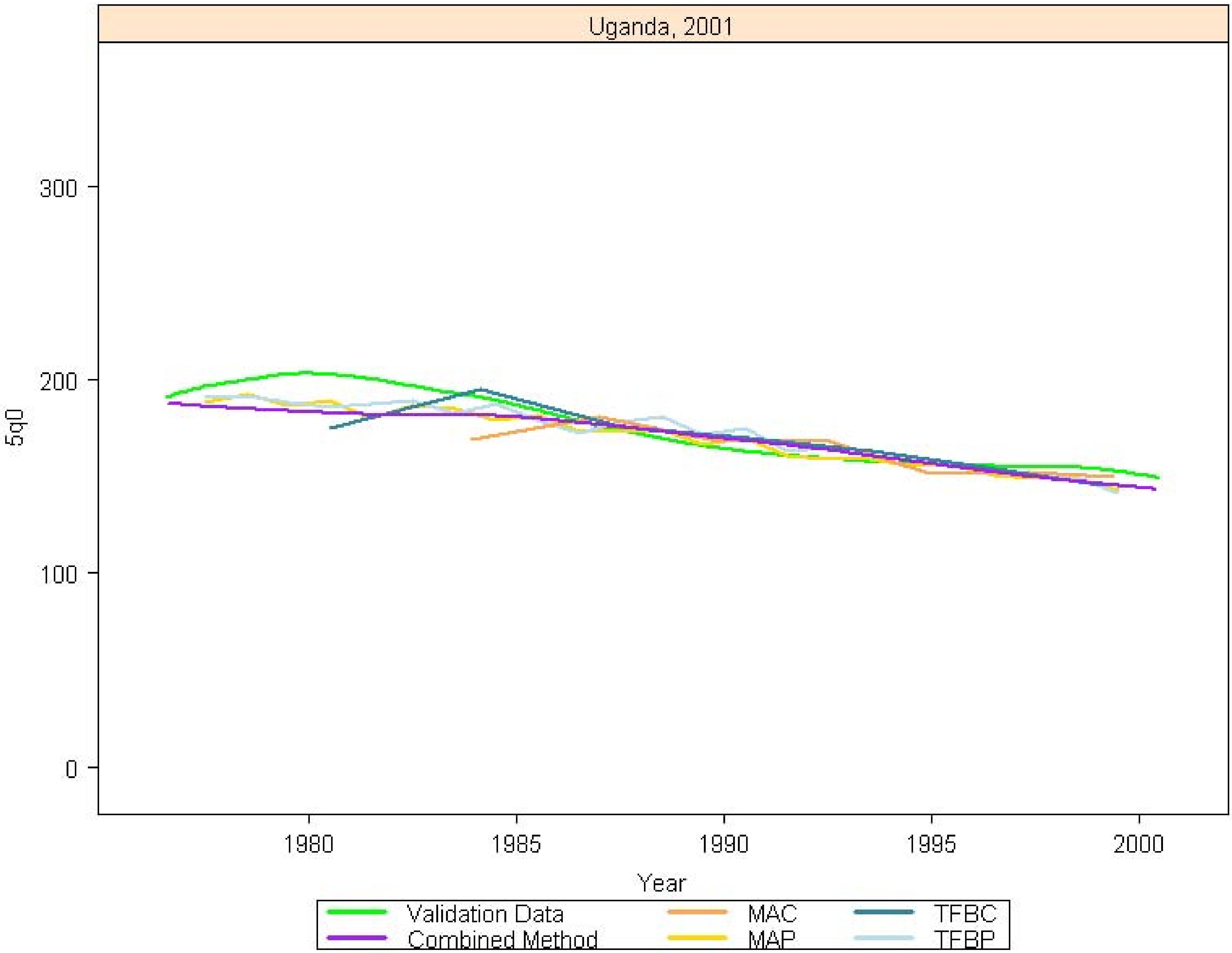
Uganda, 2001
300
200
100
0
5q0
1980
1985
1990
1995
2000
Year
Validation Data
Combined Method
MAC
MAP
TFBC
TFBP

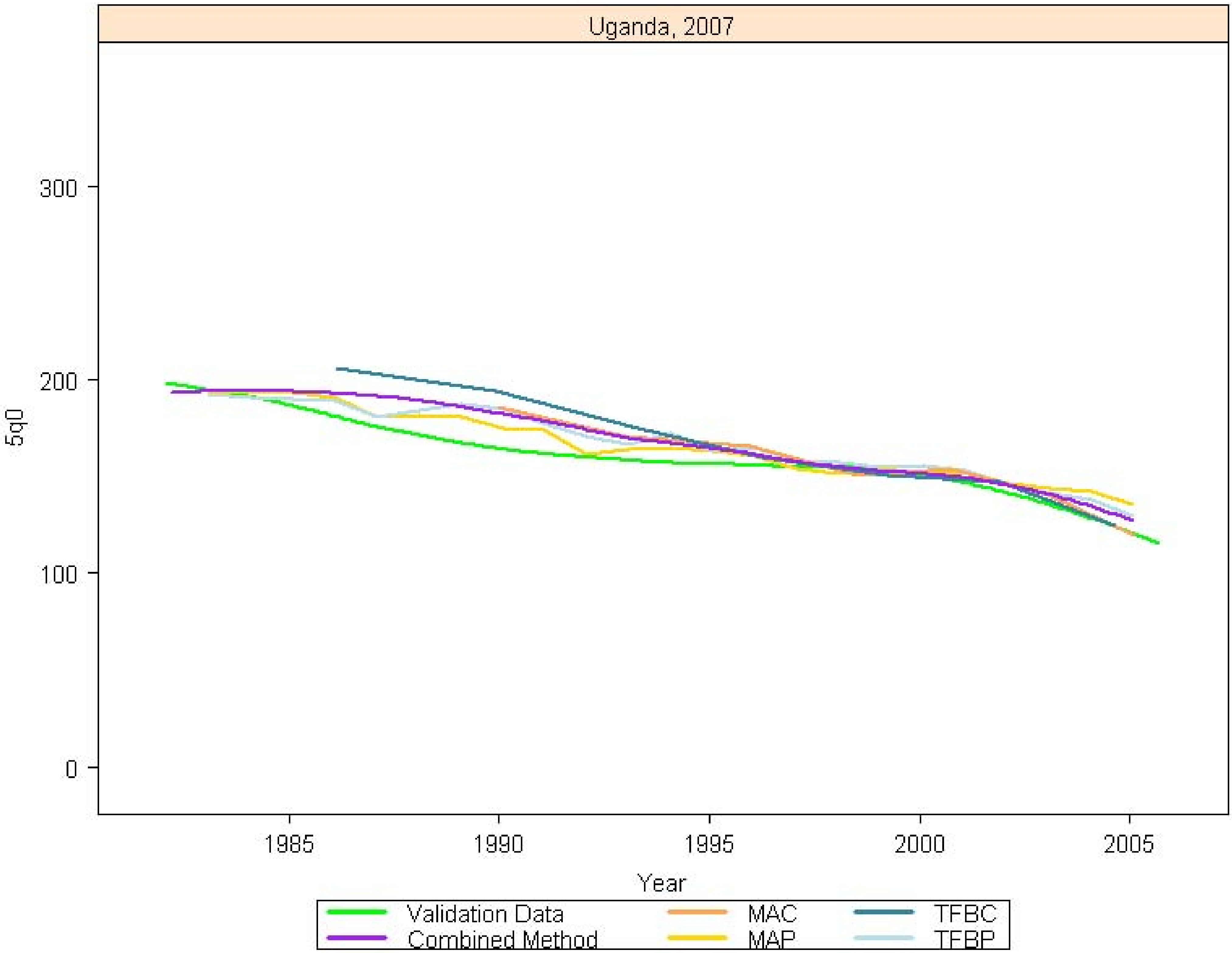

Uganda, 2007
300
200
100
0
5q0
1985
1990
1995
2000
2005
Year
Validation Data
Combined Method
MAC
MAP
TFBC
TFBP

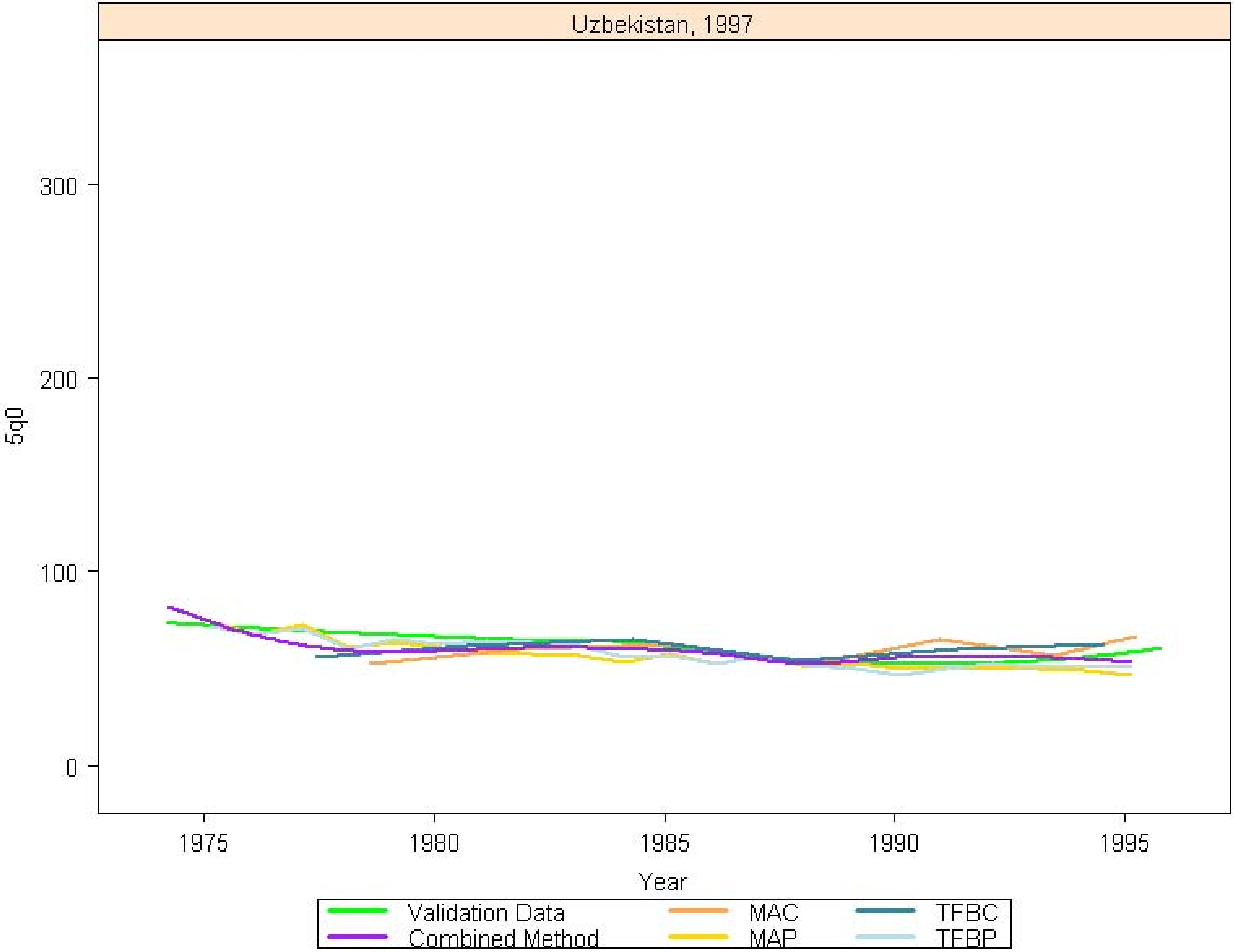
Uzbekistan, 1997
5q0
0
100
200
300
1975
1980
1985
1990
1995
Year
Validation Data
Combined Method
MAC
MAP
TFBC
TFBP

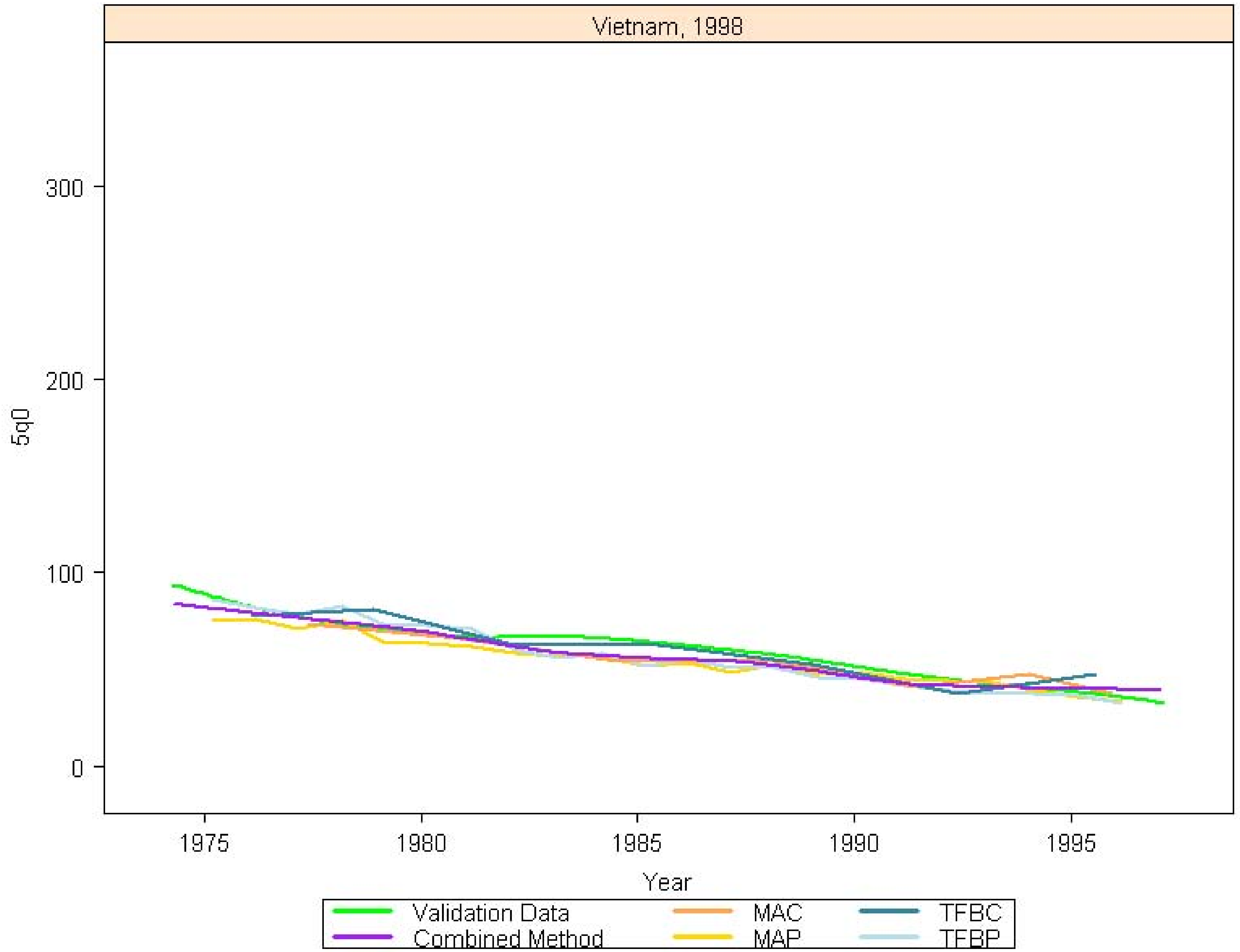
Vietnam, 1998
300
200
100
0
5q0
1975
1980
1985
1990
1995
Year
Validation Data
Combined Method
MAC
MAP
TFBC
TFBP

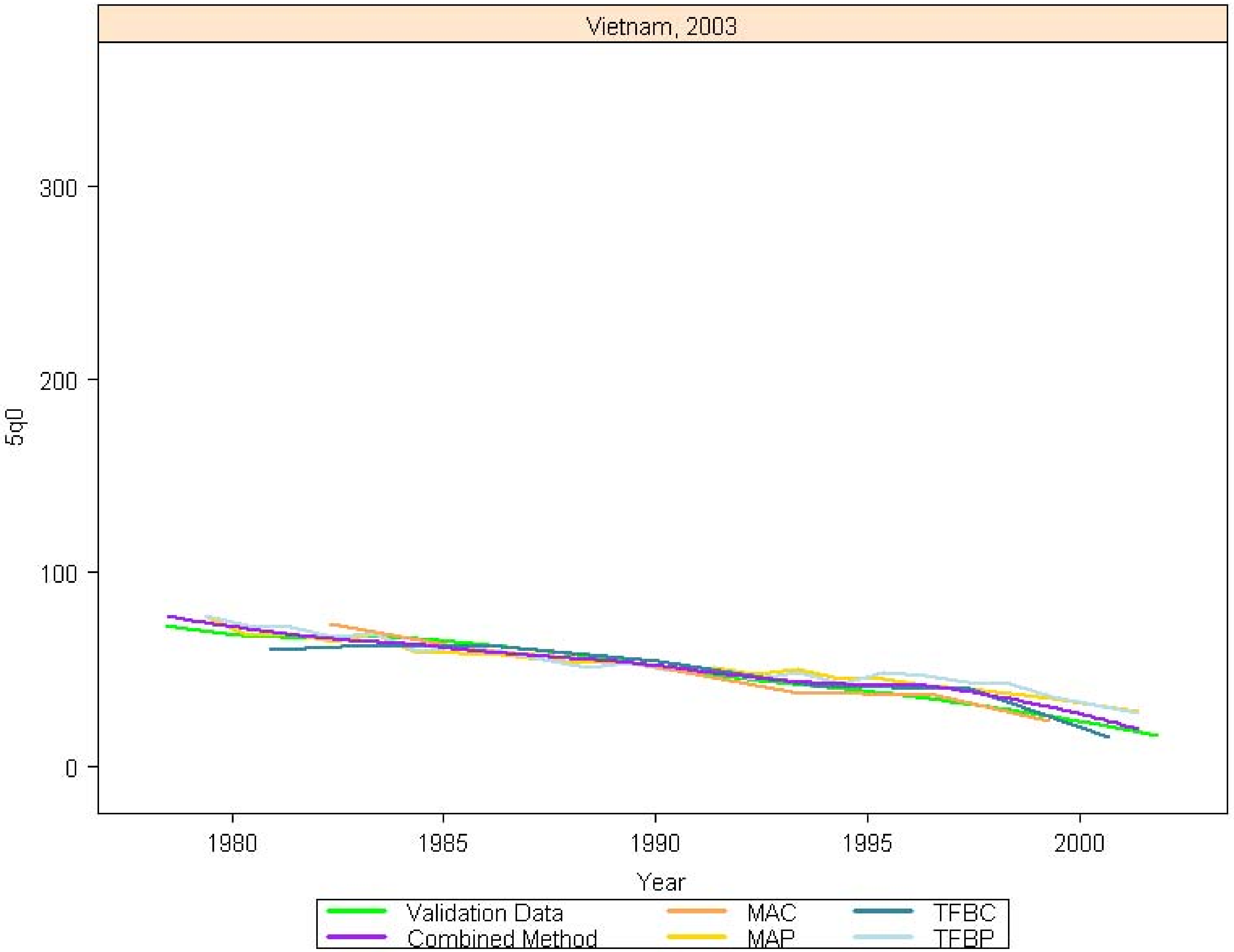
Vietnam, 2003
5q0
300
200
100
0
1980
1985
1990
1995
2000
Year
Validation Data
Combined Method
MAC
MAP
TFBC
TFBP

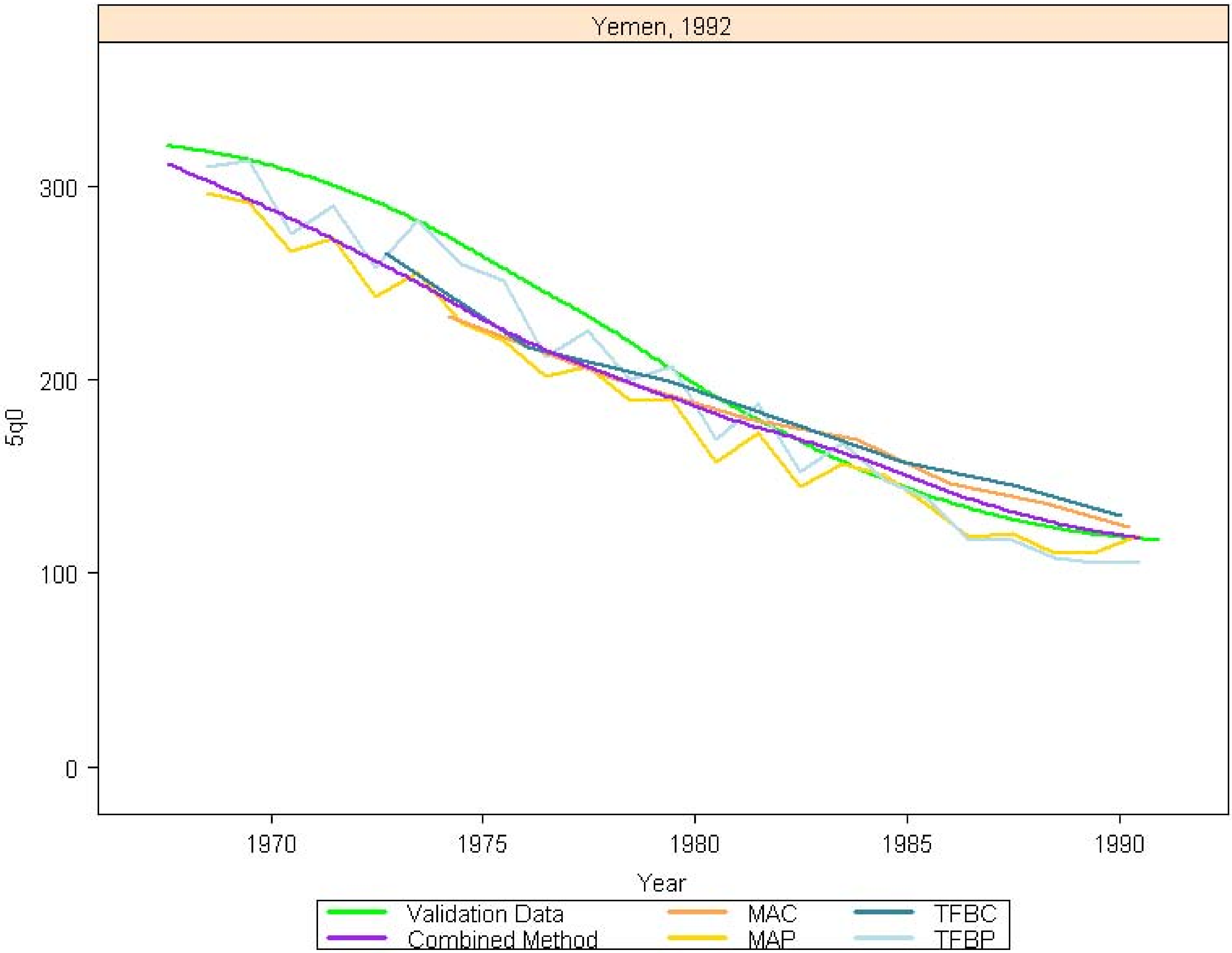
Yemen, 1992
300
200
100
0
5q0
1970
1975
1980
1985
1990
Year
Validation Data
Combined Method
MAC
MAP
TFBC
TFBP

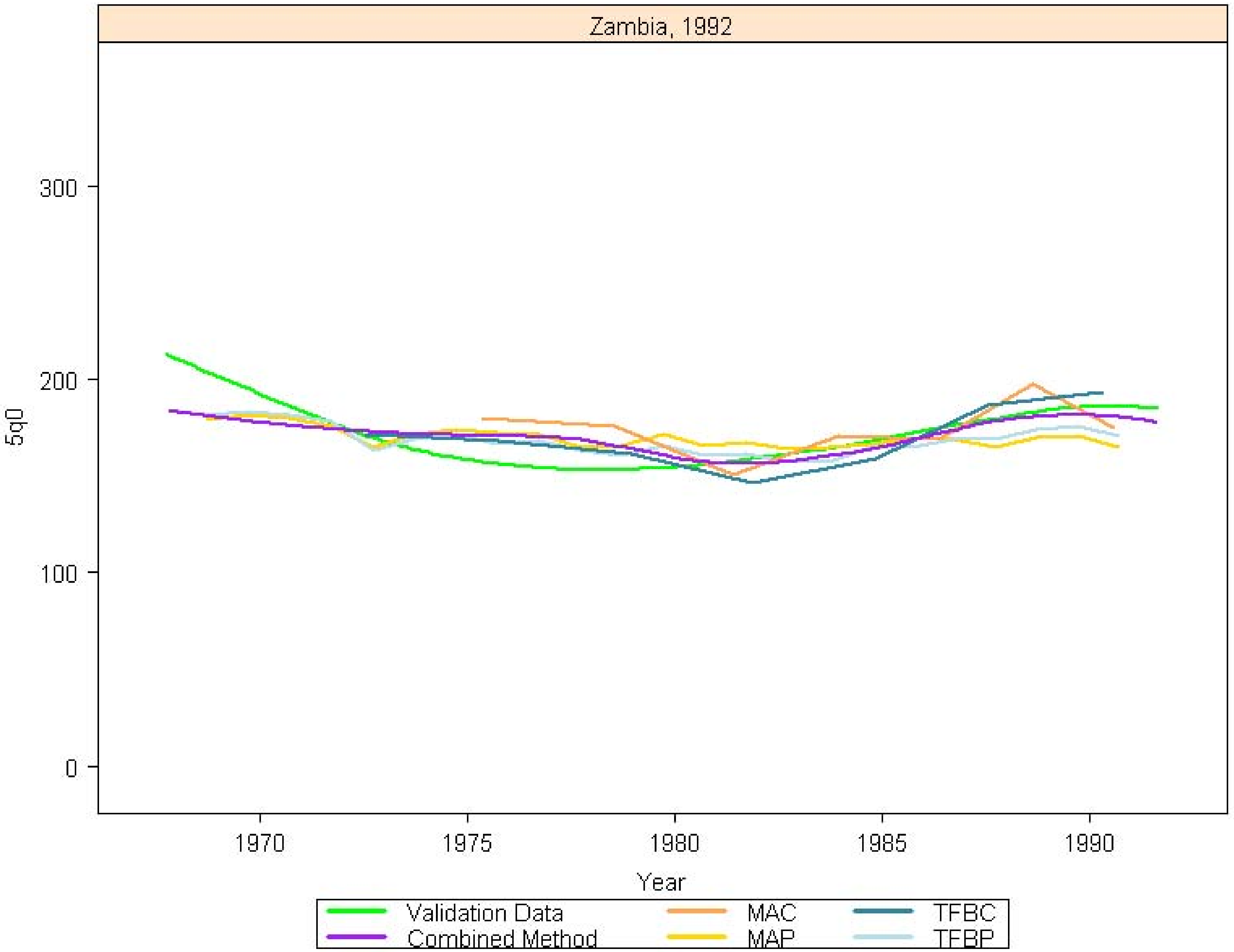
Zambia, 1992
300
200
100
0
5q0
1970
1975
1980
1985
1990
Year
Validation Data
Combined Method
MAC
MAP
TFBC
TFBP

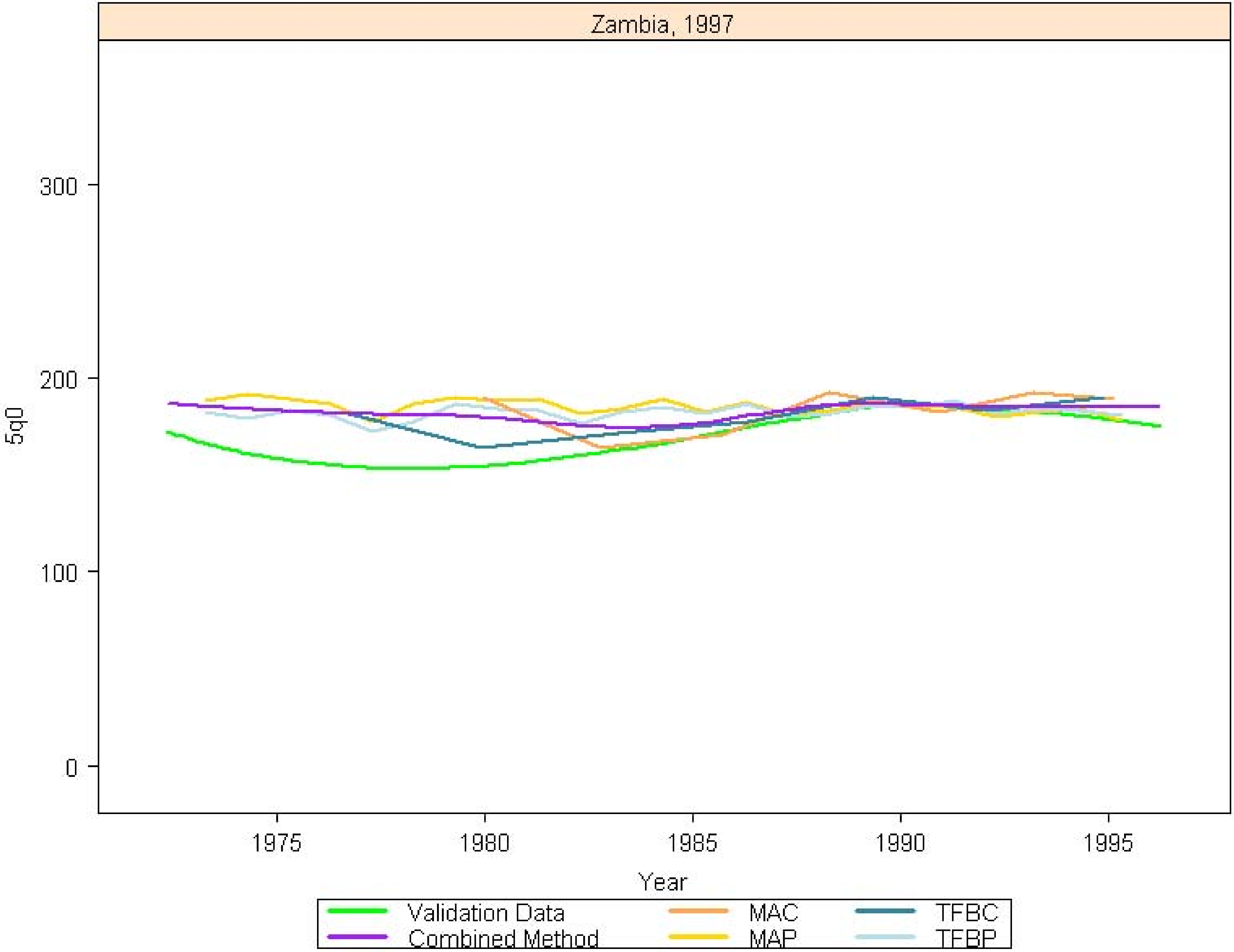
Zambia, 1997
5q0
300
200
100
0
1975
1980
1985
1990
1995
Year
Validation Data
Combined Method
MAC
MAP
TFBC
TFBP

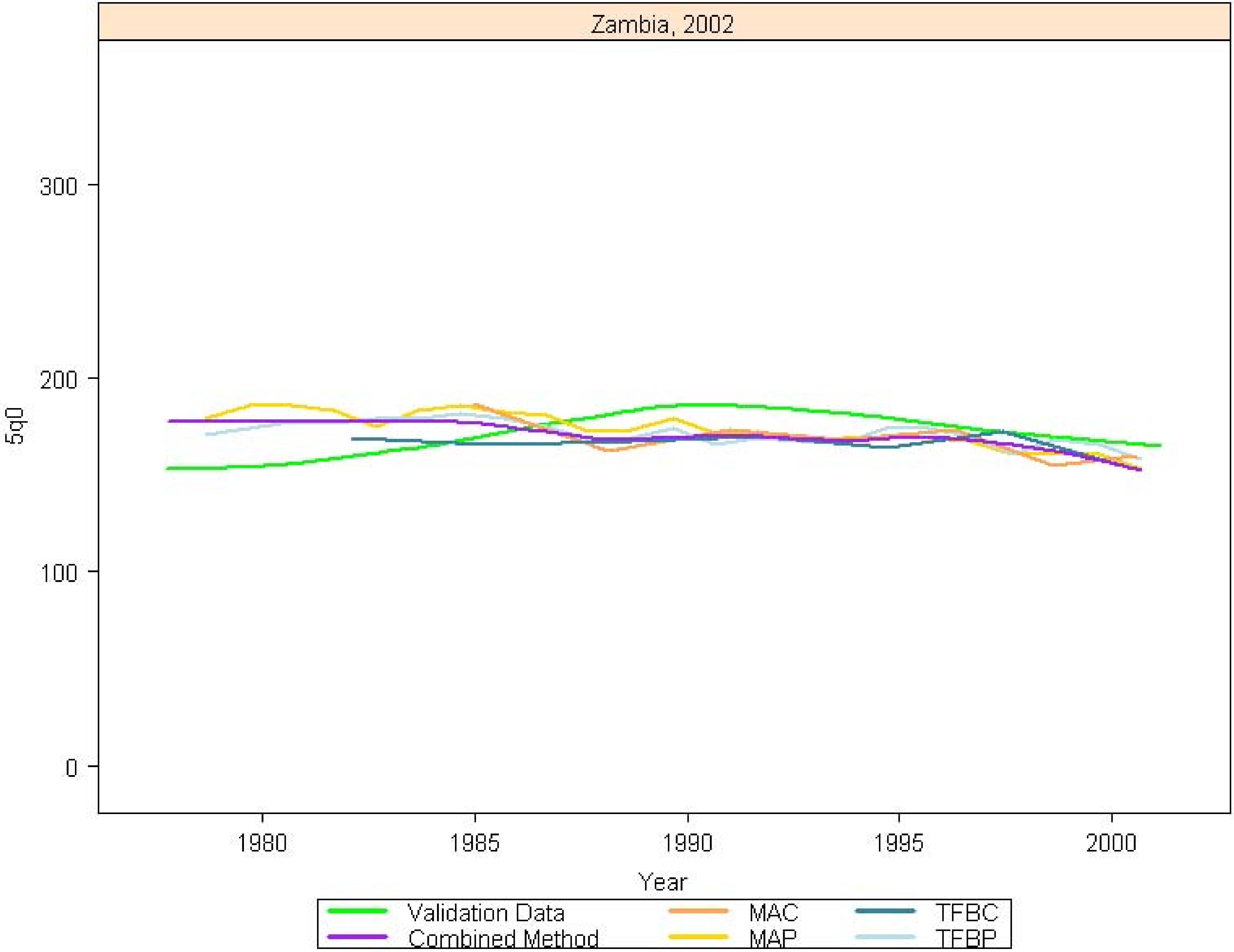
Zambia, 2002
300
200
100
0
5q0
1980
1985
1990
1995
2000
Year
Validation Data
Combined Method
MAC
MAP
TFBC
TFBP

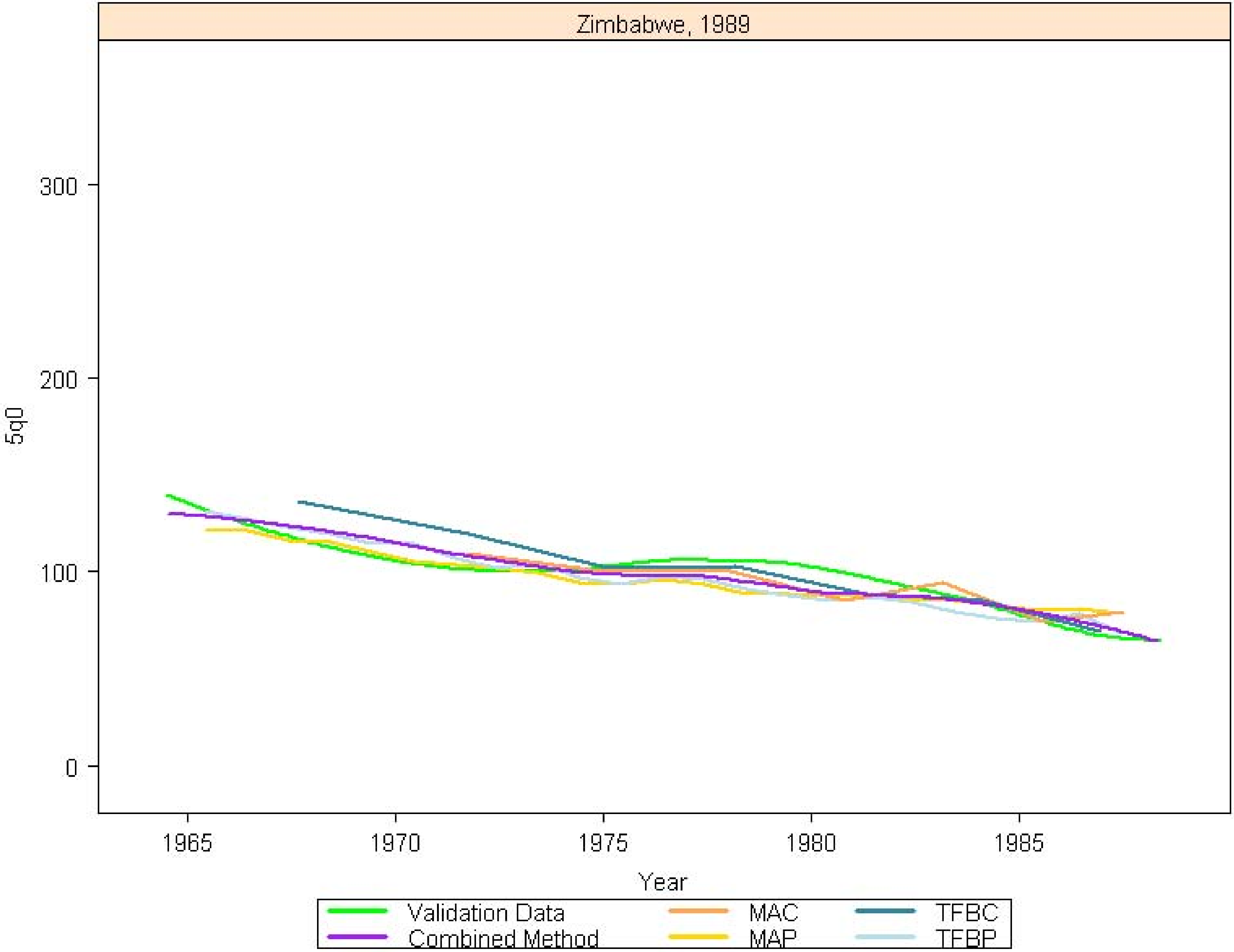

Zimbabwe, 1989
300
200
100
0
5q0
1965
1970
1975
1980
1985
Year
Validation Data
Combined Method
MAC
MAP
TFBC
TFBP

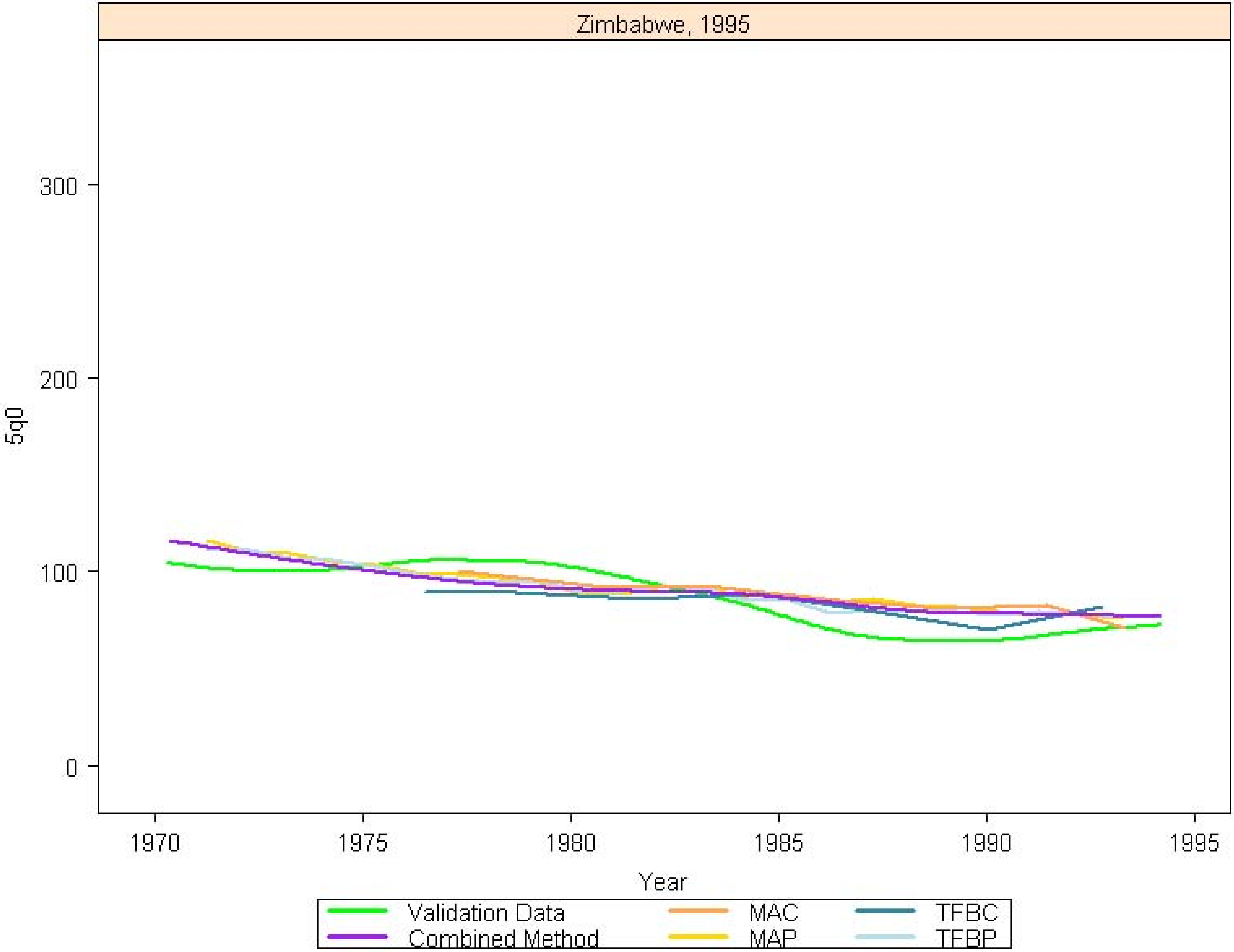
Zimbabwe, 1995
300
200
100
0
5q0
1970
1975
1980
1985
1990
1995
Year
Validation Data
Combined Method
MAC
MAP
TFBC
TFBP

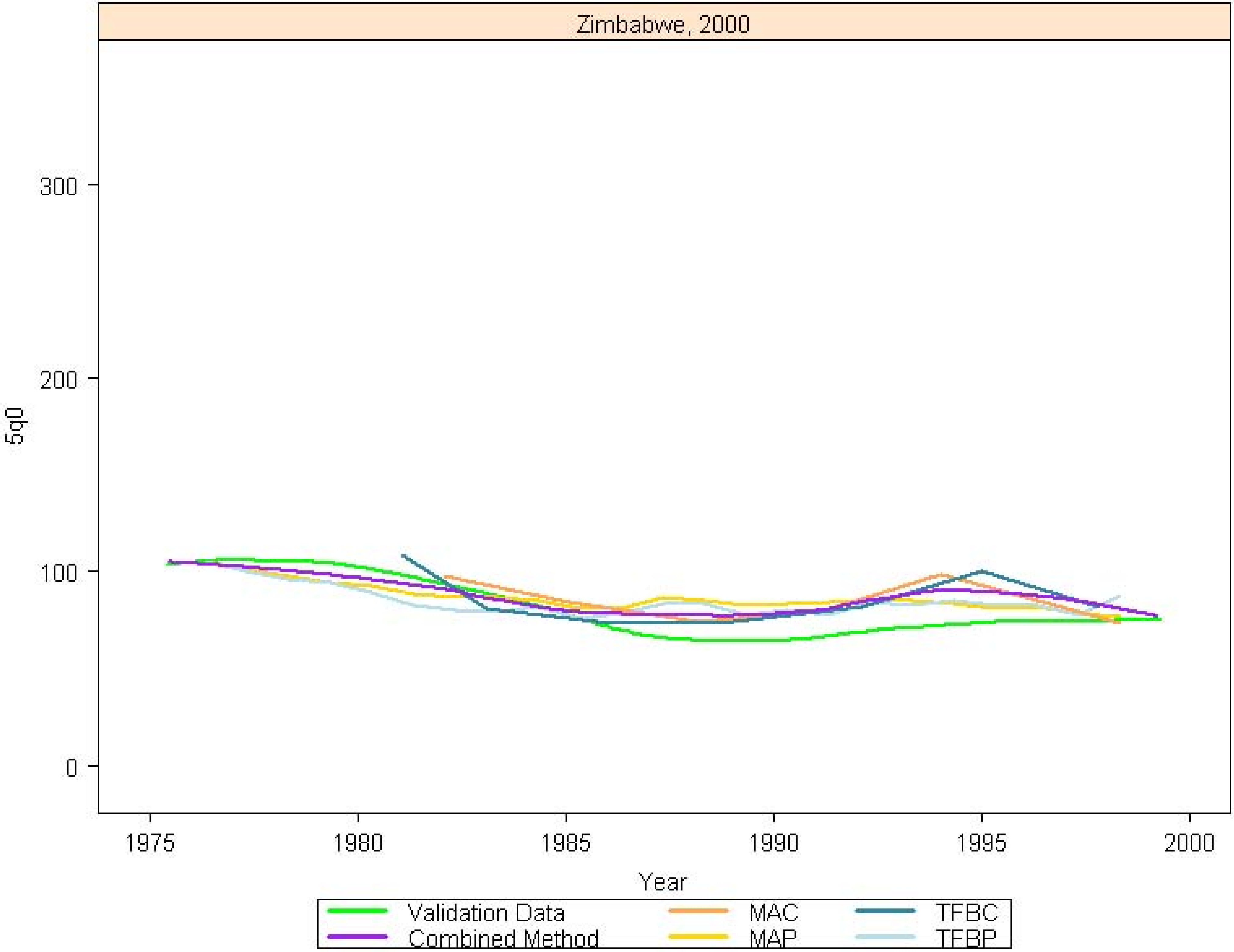
Zimbabwe, 2000
300
200
100
0
5q0
1975
1980
1985
1990
1995
2000
Year
Validation Data
Combined Method
MAC
MAP
TFBC
TFBP

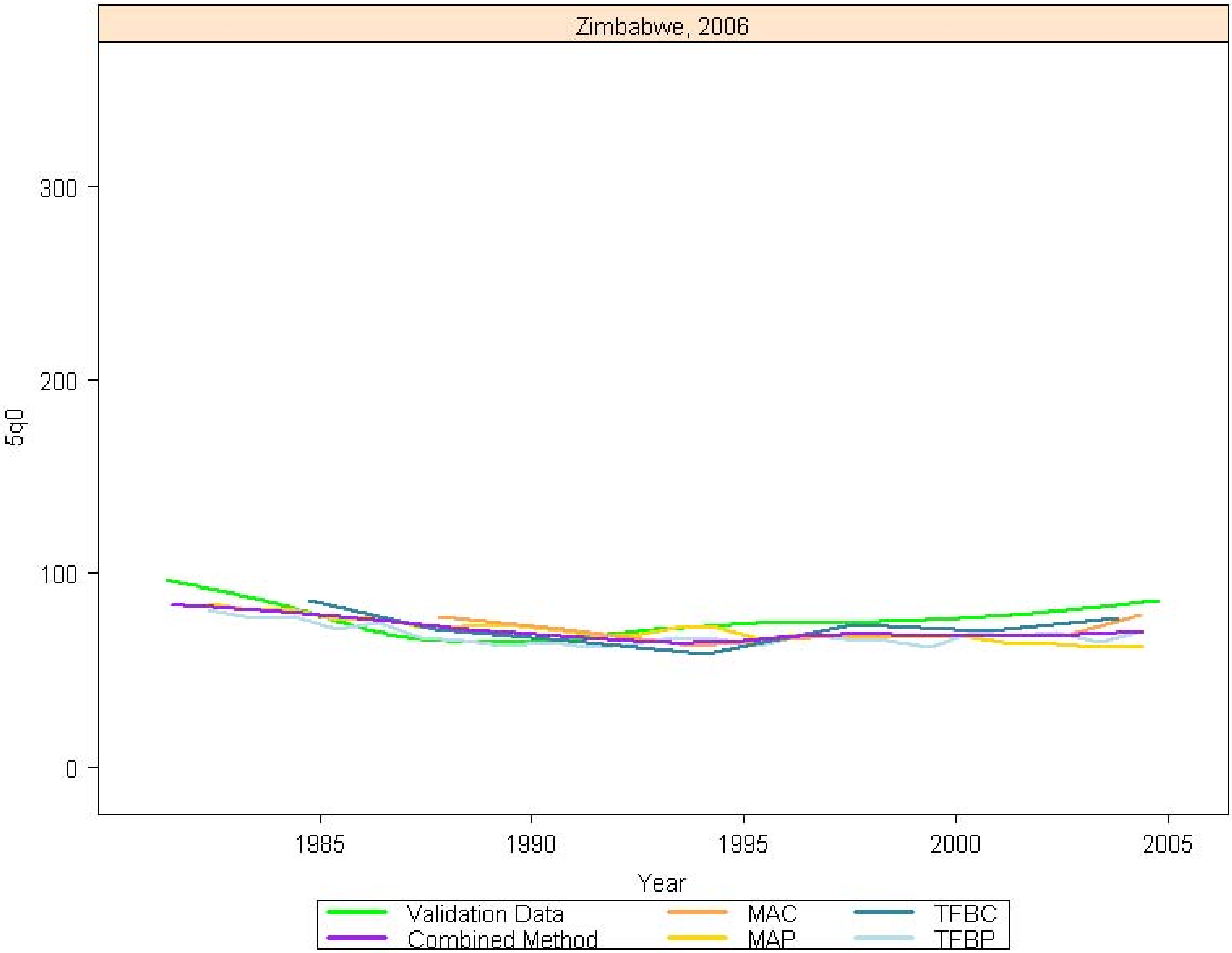

Zimbabwe, 2006
300
200
100
0
5q0
1985
1990
1995
2000
2005
Year
Validation Data
Combined Method
MAC
MAP
TFBC
TFBP
