# Supplementary material for: Measuring Under-Five Mortality: Validation of New Low-Cost Methods
Source: PLoS Med. 2010 Apr 13;7(4):e1000253. doi: 10.1371/journal.pmed.1000253 (PMC2854123; doi:10.1371/journal.pmed.1000253)
Supplement: Table S1 — Characteristics of the DHS used to build models relating under-five mortality to the CD/CEB ratio and other covariates. (0.18 MB PDF) [file pmed.1000253.s003.pdf]

Supporting Information Table S1

Country	Year	Number of Respondents	Total CEB	Total CD	Average CEB	Average CD
Armenia	2000	6430	10891	688	1.69	0.11
Armenia	2005	6566	9970	517	1.52	0.08
Azerbaijan	2006	8444	13264	1045	1.57	0.12
Bangladesh	2004	11290	33867	4800	3.00	0.43
Bangladesh	1993/94	9495	32851	6134	3.46	0.65
Bangladesh	1996/97	8982	29564	4985	3.29	0.55
Bangladesh	1999/2000	10358	32325	5085	3.12	0.49
Benin	1996	5491	18872	4036	3.44	0.73
Benin	2001	6219	19247	3564	3.09	0.57
Benin	2006	17794	56205	8818	3.16	0.50
Bolivia	1989	7923	22094	3690	2.79	0.47
Bolivia	1994	8603	23647	3896	2.75	0.45
Bolivia	1998	11187	27679	3415	2.47	0.31
Bolivia	2003	17654	44130	5403	2.50	0.31
Botswana	1988	4368	11272	815	2.58	0.19
Brazil	1986	5892	11983	1298	2.03	0.22
Brazil	1996	12612	24478	1924	1.94	0.15
Burkina Faso	2003	12477	41270	8390	3.31	0.67
Burkina Faso	1992/93	6354	22205	5119	3.49	0.81
Burkina Faso	1998/99	6445	22987	5499	3.57	0.85
Burundi	1987	3970	11998	2370	3.02	0.60
Cambodia	2000	15351	39681	5767	2.58	0.38
Cambodia	2005	16823	38823	4983	2.31	0.30
Cameroon	1991	3871	12357	2180	3.19	0.56
Cameroon	1998	5501	16018	2593	2.91	0.47
Cameroon	2004	10656	29288	4506	2.75	0.42
Central African Republic	1994/95	5884	17012	2984	2.89	0.51
Chad	2004	6085	22571	4848	3.71	0.80
Chad	1996/97	7454	26127	5760	3.50	0.77
Colombia	1986	5331	11615	888	2.18	0.17
Colombia	1990	8489	16688	981	1.97	0.12
Colombia	1995	11140	21498	1182	1.93	0.11
Colombia	2000	11585	20787	944	1.79	0.08
Colombia	2005	38355	68038	2750	1.77	0.07
Comoros	1996	3050	7913	1127	2.59	0.37
Congo (Brazzaville)	2005	7051	16799	2084	2.38	0.30

Table S1: Characteristics of the Demographic and Health Surveys (DHS) used to build models relating under-five mortality to the ratio of children dead to children ever born (CD/CEB) and other covariates.

Country	Year	Number of Respondents	Total CEB	Total CD	Average CEB	Average CD
Cote d'Ivoire	1994	8099	25026	4250	3.09	0.52
Cote d'Ivoire	2005	5183	12957	1807	2.50	0.35
Cote d'Ivoire	1998/99	3040	8421	1467	2.77	0.48
Dominican Republic	1986	7645	18328	2090	2.40	0.27
Dominican Republic	1991	7320	15761	1351	2.15	0.18
Dominican Republic	1996	8422	18079	1451	2.15	0.17
Dominican Republic	2002	23384	49952	3107	2.14	0.13
Dominican Republic	2007	27195	54485	2871	2.00	0.11
Ecuador	1987	4713	11835	1422	2.51	0.30
Egypt	1988	8911	35795	6480	4.02	0.73
Egypt	1992	9864	38211	5801	3.87	0.59
Egypt	1995	14779	54732	7875	3.70	0.53
Egypt	2000	15573	54623	6238	3.51	0.40
Egypt	2003	9159	29234	2661	3.19	0.29
Egypt	2005	19474	59884	5082	3.08	0.26
Eritrea	1995	5054	15196	2757	3.01	0.55
Ethiopia	2000	15367	47501	10724	3.09	0.70
Ethiopia	2005	14070	44191	7801	3.14	0.55
Gabon	2000	6183	15763	1662	2.55	0.27
Ghana	1988	4488	14216	2436	3.17	0.54
Ghana	1993	4562	13298	1953	2.91	0.43
Ghana	1998	4843	12758	1692	2.63	0.35
Ghana	2003	5691	14413	1806	2.53	0.32
Guatemala	1987	5160	14698	1983	2.85	0.38
Guatemala	1995	12403	35433	3850	2.86	0.31
Guatemala	1998/99	6021	17025	1558	2.83	0.26
Guinea	1999	6753	23122	5348	3.42	0.79
Guinea	2005	7954	26972	6009	3.39	0.76
Haiti	2000	10159	25412	4298	2.50	0.42
Haiti	2005	10757	23928	3243	2.22	0.30
Haiti	1994/95	5356	12759	2217	2.38	0.41
Honduras	2005	19948	47452	2651	2.38	0.13
India	1992/93	89425	277158	41653	3.10	0.47
India	1998/99	90303	270074	35572	2.99	0.39
India	2005/2006	124385	280872	32730	2.26	0.26
Indonesia	1987	11884	40392	5875	3.40	0.49
Indonesia	1991	22909	71466	9514	3.12	0.42
Indonesia	1994	28168	85923	10469	3.05	0.37
Indonesia	1997	28810	81262	8604	2.82	0.30
Indonesia	2002/2003	29483	78325	7036	2.66	0.24

Table S1: Characteristics of the Demographic and Health Surveys (DHS) used to build models relating under-five mortality to the ratio of children dead to children ever born (CD/CEB) and other covariates.

Country	Year	Number of Respondents	Total CEB	Total CD	Average CEB	Average CD
Jordan	1990	6461	32889	1883	5.09	0.29
Jordan	1997	5548	23941	1099	4.32	0.20
Jordan	2002	6006	24324	949	4.05	0.16
Kazakhstan	1995	3771	6847	387	1.82	0.10
Kazakhstan	1999	4800	8467	647	1.76	0.13
Kenya	1989	7150	26232	2775	3.67	0.39
Kenya	1993	7540	23882	2637	3.17	0.35
Kenya	1998	7881	22814	2588	2.89	0.33
Kenya	2003	8195	22548	2675	2.75	0.33
Kyrgyz Republic	1997	3848	9047	878	2.35	0.23
Lesotho	2004	7095	14585	1561	2.06	0.22
Liberia	1986	5239	16343	4075	3.12	0.78
Madagascar	1992	6260	20036	3730	3.20	0.60
Madagascar	1997	7060	22698	4209	3.21	0.60
Madagascar	2003/2004	7949	23389	3110	2.94	0.39
Malawi	1992	4849	16883	4509	3.48	0.93
Malawi	2000	13220	41404	9375	3.13	0.71
Malawi	2004	11698	35452	6349	3.03	0.54
Mali	1987	3200	12682	3956	3.96	1.24
Mali	2001	12849	49286	12612	3.84	0.98
Mali	2006	14583	52530	12407	3.60	0.85
Mali	1995/96	9704	38492	10687	3.97	1.10
Mauritania	2000/01	7728	20138	2381	2.61	0.31
Mexico	1987	9310	23617	2220	2.54	0.24
Morocco	1987	5982	25518	3769	4.27	0.63
Morocco	1992	9256	22657	2840	2.45	0.31
Morocco	2003-2004	16798	31697	2807	1.89	0.17
Mozambique	1997	8779	26871	6177	3.06	0.70
Mozambique	2003	12418	38951	8310	3.14	0.67
Namibia	1992	5421	13206	1347	2.44	0.25
Namibia	2000	6755	14508	1013	2.15	0.15
Nepal	1996	8429	28828	5345	3.42	0.63
Nepal	2001	8726	28775	4415	3.30	0.51
Nepal	2006	10793	26340	3351	2.44	0.31
Nicaragua	2001	13060	32644	2503	2.50	0.19
Nicaragua	1997/98	13634	35328	2884	2.59	0.21
Niger	1992	6503	25048	8000	3.85	1.23
Niger	1998	7577	29784	9134	3.93	1.21
Niger	2006	9223	36861	9162	4.00	0.99

Table S1: Characteristics of the Demographic and Health Surveys (DHS) used to build models relating under-five mortality to the ratio of children dead to children ever born (CD/CEB) and other covariates.

Country	Year	Number of Respondents	Total CEB	Total CD	Average CEB	Average CD
Nigeria	1990	8781	29075	6033	3.31	0.69
Nigeria	2003	7620	23579	5432	3.09	0.71
Pakistan	1990/91	6611	26870	3578	4.06	0.54
Pakistan	2006/07	10023	38824	4198	3.87	0.42
Paraguay	1990	5827	14438	845	2.48	0.14
Peru	1986	4999	13291	1853	2.66	0.37
Peru	1992	15882	35910	4133	2.26	0.26
Peru	1996	28951	64997	6345	2.25	0.22
Peru	2000	27843	58785	5373	2.11	0.19
Peru	2004-2008	11717	22722	1686	1.94	0.14
Philippines	1993	15029	34560	2589	2.30	0.17
Philippines	1998	13983	30142	2158	2.16	0.15
Philippines	2003	13633	29714	1758	2.18	0.13
Rwanda	1992	6551	20107	3994	3.07	0.61
Rwanda	2000	10421	28966	6351	2.78	0.61
Rwanda	2005	11321	30377	6139	2.68	0.54
Senegal	1986	4415	14389	3432	3.26	0.78
Senegal	1997	8593	26367	4471	3.07	0.52
Senegal	2005	14602	38770	5815	2.66	0.40
Senegal	1992/93	6310	20815	4012	3.30	0.64
South Africa	1998	11735	22756	1809	1.94	0.15
Sri Lanka	1987	5864	17644	1009	3.01	0.17
Sudan	1990	5860	25805	3752	4.40	0.64
Swaziland	2006	4987	11383	1136	2.28	0.23
Tanzania	1992	9238	28688	4896	3.11	0.53
Tanzania	1996	8120	25063	4081	3.09	0.50
Tanzania	1999	4029	11787	2078	2.93	0.52
Tanzania	2004	10329	30077	4530	2.91	0.44
Thailand	1987	6775	18612	1501	2.75	0.22
Togo	1988	3360	10782	2002	3.21	0.60
Togo	1998	8569	25119	4160	2.93	0.49
Trinidad and Tobago	1987	3806	7837	375	2.06	0.10
Tunisia	1988	4184	16463	1779	3.93	0.43
Turkey	1993	6519	19827	2418	3.04	0.37
Turkey	1998	8576	17209	1753	2.01	0.20
Turkey	2003	8075	21173	1697	2.62	0.21

Table S1: Characteristics of the Demographic and Health Surveys (DHS) used
to build models relating under-five mortality to the ratio of children dead to children ever born (CD/CEB) and other covariates.

Country	Year	Number of Respondents	Total CEB	Total CD	Average CEB	Average CD
Uganda	1988	4730	16523	3152	3.49	0.67
Uganda	1995	7070	24087	4097	3.41	0.58
Uganda	2006	8531	30155	4961	3.53	0.58
Uganda	2000/01	7246	24922	4209	3.44	0.58
Uzbekistan	1996	4415	9979	603	2.26	0.14
Vietnam	1997	5664	15805	940	2.79	0.17
Vietnam	2002	5665	14394	808	2.54	0.14
Yemen	1991/92	5687	27798	4872	4.89	0.86
Zambia	1992	7060	21921	3802	3.10	0.54
Zambia	1996	8021	24358	4589	3.04	0.57
Zambia	2001/02	7658	23211	4047	3.03	0.53
Zimbabwe	1988	4201	12405	1212	2.95	0.29
Zimbabwe	1994	6128	16495	1549	2.69	0.25
Zimbabwe	1999	5907	13628	1242	2.31	0.21
Zimbabwe	2005/06	8907	19174	1425	2.15	0.16

Table S1: Characteristics of the Demographic and Health Surveys (DHS) used to build models relating under-five mortality to the ratio of children dead to children ever born (CD/CEB) and other covariates.
