# Supplementary material for: Measuring Under-Five Mortality: Validation of New Low-Cost Methods
Source: PLoS Med. 2010 Apr 13;7(4):e1000253. doi: 10.1371/journal.pmed.1000253 (PMC2854123; doi:10.1371/journal.pmed.1000253)
Supplement: Text S1 — Application of summary birth history methods for estimating under-five mortality. (7.27 MB ZIP) [file pmed.1000253.s004.zip › How to apply SBH methods by Rajaratnam et al.pdf]

Application of Summary Birth History Methods for Estimating Under-five Mortality

Linda N. Tran and Julie Knoll Rajaratnam

Table of Contents

Introduction 4
Getting oriented 5
Brief introduction to Stata 6
The interface 6
Setting up Stata 7
Brief introduction to R 8
The interface 8
Setting up R 9
Getting Ready to Apply Summary Birth History Methods 9
What you need 9
How the microdata should look before starting 9
Prepare the microdata 10
Maternal Age Cohort Method (MAC) 11
Quick review 11
Familiarize yourself with the MAC datasets 12
Prepare data to apply MAC method 12
Apply the MAC method 13
Maternal Age Period-derived Method (MAP) 15
Quick review 15
Familiarize yourself with the MAP datasets 16
Prepare data: Create age groups 16
Prepare data: Obtain the expected number of children ever born for each year prior to the survey date 17
Prepare data: Obtain the expected number of dead children for each year prior to the survey date .. 18
Prepare data to apply MAP model 20
Apply the MAP method 20
Time Since First Birth Methods (TFBC and TFBP) 22
Applying the Models to Aggregated Data 22
Combined Method 23
Quick review 23

Apply the Combined Method 23
Resources 25
Zip file 25
References 25
Contacts 25

Introduction

To estimate a time series of under-five mortality from summary birth histories, two pieces of information must be estimated: 1) the estimate of under-five mortality itself and 2) the date to which the estimate pertains, called the *reference time*. The Cohort-derived and Period-derived Methods, as discussed by Rajaratnam et al (2010), provide two different ways to obtain these two pieces of information, either by using the mother's age or the time since her first birth, to construct a series of under-five mortality across time. This guide has been constructed to show you how to apply these Summary Birth History Methods, step-by-step.

The next section in this chapter, "Getting Oriented", will show you what you need to know in order to smoothly apply the Summary Birth History Method while using this guide. This tutorial describes the application of these methods using two statistical software packages: Stata and R. The second and third sections of this manual are brief introductions to both Stata and R, which you are welcome to skip if you are familiar with the platforms. This guide assumes that you are able to download and install both software packages on your computer, and that the data to which you will apply the methods is in Stata format. However, the tutorial for the direct application of the methods has been written assuming you have little knowledge of Stata or R or even programming in general.

The next chapters will guide you through applying the Summary Birth History Methods step-by-step using mainly Stata. You will not have to use R until near the end. Each section will start off with a "Quick Review" section to provide a brief overview of the methods.

Lastly, we should mention that this guide does not provide instructions on constructing the uncertainty bounds for the estimates. Since we wanted to make this guide accessible to all users and since generating uncertainty intervals requires a lot of computational power, we have excluded the uncertainty functionality of the methods. IHME is currently developing a free, easy-to-use application which will allow users to apply these methods to their own data and will include uncertainty intervals with the output. You can register on IHME's website to be notified when it is released.

Getting oriented

Throughout this guide, the *red italicized text* will be replaced manually by you. To give you an idea of what you'll need to replace, we have listed some of the *red italicized text* here:

- *directory*: the path that contains all the relevant data files, e.g. your microdata and unzipped files provided. This is also the directory where the results will be saved by the program.
- *microdata*: the name of your microdata file. This zip file contains a sample microdata file containing summary birth history data from a fictitious population census in Cambodia, during the year 1234.
- *log_filename*: name of your log file

Each line will start with a "greater than sign" (**>**) to indicate a new line of code. ***Don't type this symbol into the command line.***

Although you will be tempted to just type the code directly into the command line while moving along with the guide, we do not recommend this. If you make any small error, you'll have to retype everything again in order to return to where you were at. We recommend using a text editor like Notepad to type in the code and copy and paste it into the command line.

Brief introduction to Stata

This section will provide you with a cursory introduction in Stata, just enough to get you through this guide. If you want to feel more comfortable with Stata, you can find some resources for learning Stata here: http://www.stata.com/links/resources1.html. Also, we assume that you have Stata installed and ready to go.

The interface

Below is a sample Stata window with some basic components labeled. The **variable list** shows you what variables are in the dataset you have open. The **command line** is where you will type in commands; those commands are recorded in the **review panel** and also displayed in the **results window**, along with the command output. Throughout this guide, commands to be typed into the **command line** will be in `Courier` font.

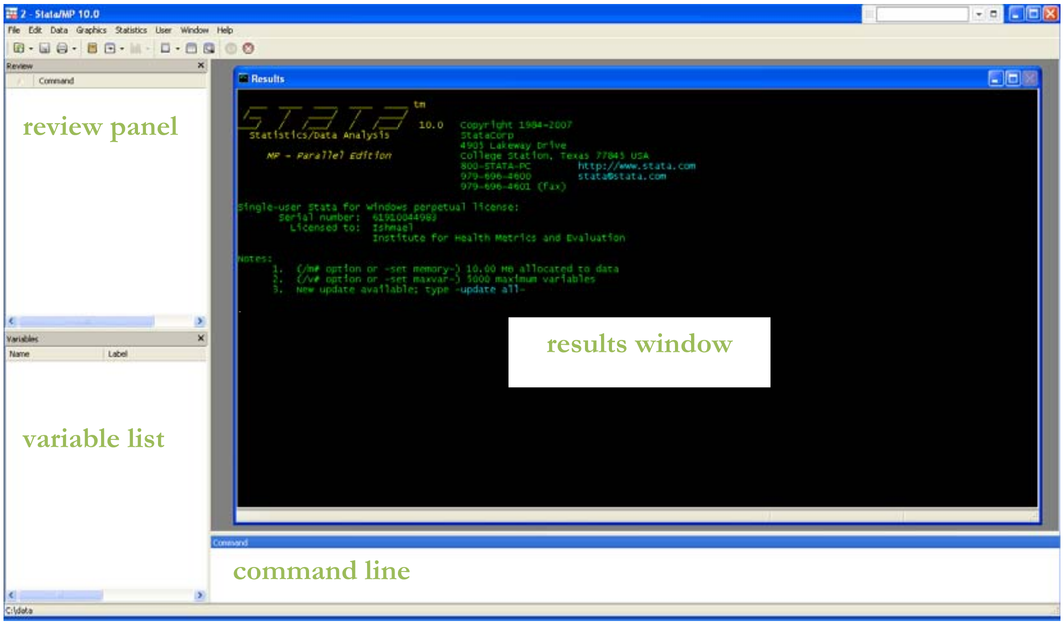

When you start the exercise, the following tips will help you use Stata comfortably:

- Pressing "Enter" on your keyboard will execute commands.
- To return to a previously typed command, press the *Page Up* button on your keyboard
- Stata does not have an "Undo" function like Microsoft Excel. To avoid losing your work, type all commands into a text editor, like Notepad or Microsoft Word; that way you can redo everything by copying and pasting the commands into Stata again.
- The Data Browser (the window that pops up after typing `browse`) must be closed in order to type commands into the command line.

- When typing in commands, be careful to mimic all quotation marks, spaces, line breaks, commas, parentheses, etc. that you see in the instructions to ensure the proper execution of the command. ***Be careful of this funny-shaped symbol:*** **`** . In the following example, it appears just prior to the word "year":

```
1   forvalues year = 2000/2009 {
2     display `year'
3   }
```

Notice that it looks like year is enclosed with single quotes on line 2. That's only half true—the second single quote *is* a single quote, but the first "single quote" is actually that weird symbol. On most keyboards, you can find this weird symbol on the left side of the "1" key near the top left corner of your keyboard (marked red in the keyboard diagram below). If you have a different type of keyboard, it's most likely on the same row as your spacebar.

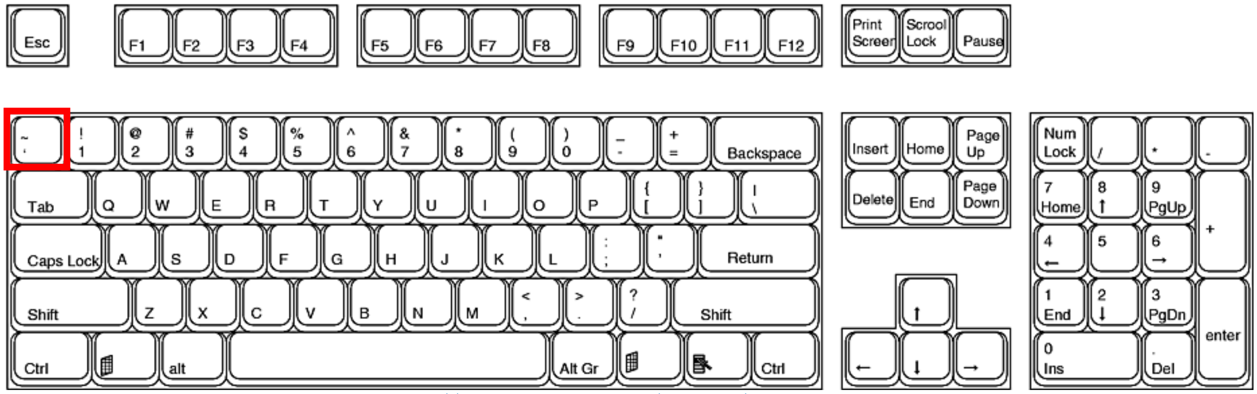

Keyboard diagram taken from: RedGrittyBrick (http://www.redgrittybrick.org/terminals/keyboards.html)

Setting up Stata

1. Launch Stata.

2. Stata's default memory allocation (10mb) is smaller than what you will need to read in your microdata, so you need to increase it before opening your dataset.

   > set memory *500m*

   The above line tells Stata to increase its memory to 500 megabytes. You might want to increase the size depending on the size of your microdata file. As a rule of thumb, you increase the memory a little bit more than the size of the microdata file.

3. Set the working directory. This tells Stata where to look for data:

   > cd "*directory*"

4. Open a log file. This will record the output of the results window from now until you close it. You may find it to be a useful reference later.

   > log using *log_filename*, text

Opening the enclosed data files in Stata

1. To open Stata files (with the file extension .dta), simply use the command:

```
> use "filename.dta", clear
```

2. If you are unable to open a Stata file because it was created with a later version of Stata than the version you are using, try accessing the comma-delimited data (.csv) files, using this command:

```
> insheet using "filename.csv", c clear
```

3. You can then save them to a Stata format that your version of Stata can read by typing:

```
> save "filename.dta", replace
```

Brief introduction to R

This section will provide you with a cursory introduction to R, just enough to get you through this guide. If you want to feel more comfortable with R, you can find some R manuals here: http://cran.r-project.org/manuals.html. Also, we assume that you have R installed and ready to go. R is free to the public. You can download the software here: http://cran.r-project.org/.

The interface

Below is a sample R window with some basic components labeled. The **console** is where you will type in commands and will show the output resulting from your command; your commands will be in red and the results will be in dark blue. Throughout this guide, commands to be typed into the console will be in `Courier` font. There is also the **graphics** window, where all your plots will appear. You can resize these windows to your liking.

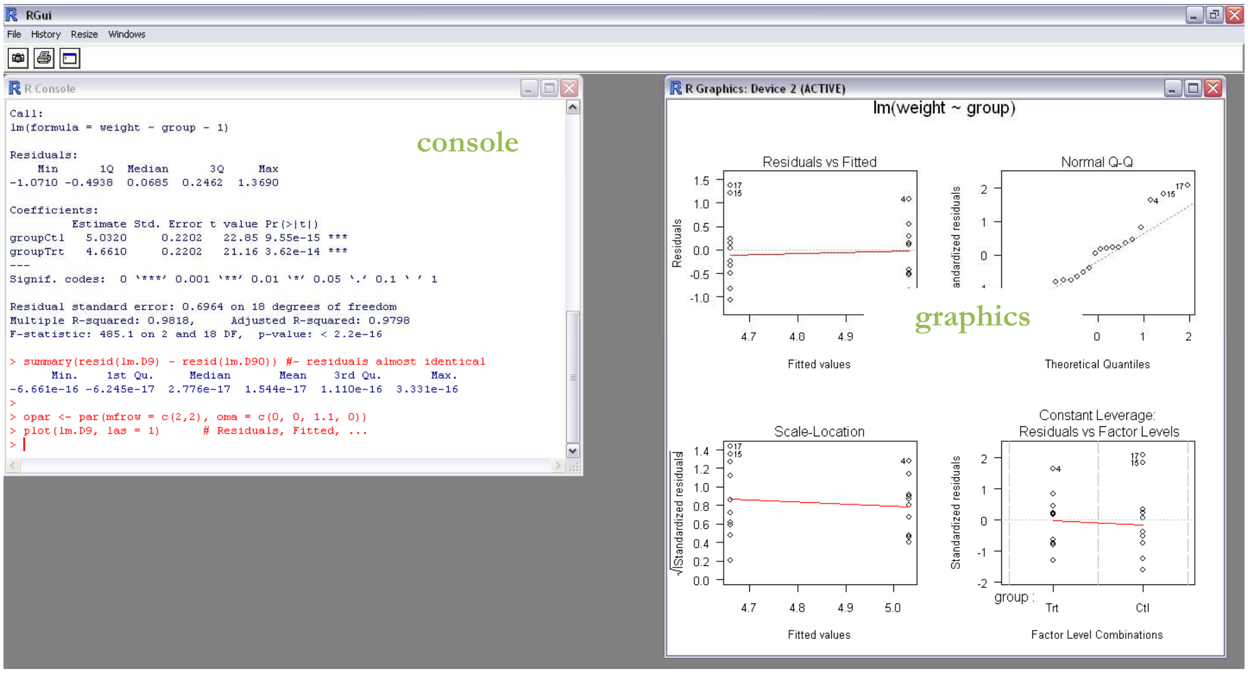

When you start the exercise, the following tips will help you use R comfortably:

- Pressing "Enter" on your keyboard will execute commands.
- To return to a previously typed command, press the *Up Arrow* on your keyboard
- R does not have an "Undo" function like Microsoft Excel. To avoid losing your work, type all commands into a text editor, like Notepad; that way you can redo everything by copying and pasting the commands into R again.

Setting up R

1. Launch R.

2. Set the working directory. This tells R where to look for data. Note that R only reads forward slashes "/" so if you cut and paste the directory path from Windows Explorer, you will need to replace back slashes "\" with forward slashes.

```
> setwd("directory")
```

Getting Ready to Apply Summary Birth History Methods

What you need

To apply the Summary Birth History Methods, the following information is needed from each female in the survey or census:

1. her age and/or time since her first birth,
2. total number of children that she has ever borne, and
3. total number of children that have survived.

Besides this information (called "microdata"), you will also need the coefficients estimated by Rajaratnam et al. These are included in the zip file through which you downloaded this document and are also accessible on the IHME website (http://www.healthmetricsandevaluation.org). All of these files should be in the same folder.

How the microdata should look before starting

You will need to format your microdata before moving on with this guide. We assume that you have done the necessarily data quality checks (e.g. make sure each respondent has a plausible number of children and a plausible age) before deciding to apply the methods. Also, your microdata need to be in a Stata format or, in other words, have an file extension of ".dta".

To give you an idea of what your microdata should look like, an example of microdata is provided below.

iso3	country	region	svdate	wtper	sex	age	timefb	ceb	cd
KHM	Cambodia	ASIA	1234	10.1	1	34	.	.	.
KHM	Cambodia	ASIA	1234	8.5	2	30	12	3	0
KHM	Cambodia	ASIA	1234	9.2	1	4	.	.	.
KHM	Cambodia	ASIA	1234	10.1	1	43	.	.	.
KHM	Cambodia	ASIA	1234	7.2	2	43	27	5	1
KHM	Cambodia	ASIA	1234	8.3	2	17	.	0	0

```
KHM   Cambodia    ASIA     1234     7.2     2    63     48      7    2
KHM   Cambodia    ASIA     1234     8.5     2    25      .      0    0
```

The variable names in your microdata ***must match exactly in spelling and case*** with those in this example in order to continue on with this exercise. Below is a description of the variables that you ***must have*** in your dataset:

- `iso3`: the iso3 code of the country from which your microdata come. If you don't know the code for your country, you can look it up at: http://en.wikipedia.org/wiki/ISO_3166-1_alpha-3
- `region`: the region that the country belongs in. You must choose one of the five regions below. Unfortunately, the methods will not produce estimates for countries falling outside these five regions. The following four-letter codes have been assigned to each region is as follows and must be used to identify the region for your dataset:
    - `ASIA` = Asia
    - `LATC` = Latin America and the Caribbean
    - `NAME` = North Africa / Middle East
    - `SASE` = Sub-Saharan Africa, South/East
    - `SAWC` = Sub-Saharan Africa, West/Central
- `svdate`: the year that the survey/census was finished.
- `wtper`: sample weight given to the respondent. If no sample weights are provided, then generate the `wtper` variable with a value of 1 for each respondent.
- `sex`: sex of the respondent, where 1 indicates a male and 2 is female.
- `age` (age of the respondent in years) and/or `timefb` (time since first birth of the respondent in years)
- `ceb`: total number of children ever born to the respondent. Not important for the males in the sample, we will drop them later.
- `cd`: total number of dead children. Again, not important for the males in the sample.

Things to check in the microdata:

- Check the "unknowns", "missings", or "don't knows". We recommend filling them in using an appropriate imputation method. If not, replace them with Stata's missing value of ".".
- Make sure that the number of children ever born is higher than the number of children surviving/dead for each person. If your dataset is relatively "clean", the error here might be caused by the way that "unknowns", "missings", or "don't knows" are coded, e.g. with 99s.

Prepare the microdata

1. Open a Stata session and initialize the session (steps outlined in "Brief Introduction to Stata"). Remember to change the directory, set the memory, and open a log file. The directory that you set will be where your microdata and the files from the zip file are located and where your output will be generated.

2. Just in case you forgot how your microdata looks, familiarize yourself with the dataset that you'll be manipulating in the next section. You can do this by first opening the dataset and then browsing it, via the following commands. Note: if you have trouble opening the Stata files

included with this tutorial, see the section above (p. 8) for opening the enclosed data files in Stata.

```
> use “microdata.dta”, clear
> browse
```

Also, the following command will display a description of all variables in your dataset.

```
> codebook
```

But if you only want to know about one of them, this would work:

```
> codebook variable
```

If you want to look at the unique values of the variables, the following command will give you a frequency table of the variable’s unique values. Make sure you don’t use it on continuous variables!

```
> tab variable
```

3. Drop all males.

```
> drop if sex == 1
```

4. Keep only women between the ages of 15 to 49.

```
> keep if age >= 15 & age < 50
```

5. Keep the relevant variables and save the modified microdata to be used later.

```
> save “microdata modified.dta”, replace
```

Maternal Age Cohort Method (MAC)

Quick review

With the Cohort Methods, under-five mortality and reference time are estimated through birth history information from the mothers and, in this case, her age (the alternative is the time since her first birth).

Here are the formulas that quantify the MAC method:

reference time component

$$reftime_{ijk} = \beta_{0i} + \beta_{1i}\frac{CD_{ijk}}{CEB_{ijk}} + \beta_{2i}CEB_{ijk} + \beta_{3i}\frac{P(15-19)_{jk}}{P(20-24)_{jk}} + \beta_{4i}\frac{P(20-24)_{jk}}{P(25-29)_{jk}} + \epsilon_{ijk}$$

${}_5q_0$ component

$$\text{logit}({}_5q_{0\,ijk}) = \beta_{0i} + U_{ij} + \beta_{1i}\text{logit}\left(\frac{CD_{ijk}}{CEB_{ijk}}\right) + \beta_{2i}CEB_{ijk} + \beta_{3i}\frac{P(15-19)_{jk}}{P(20-24)_{jk}} + \beta_{4i}\frac{P(20-24)_{jk}}{P(25-29)_{jk}} + \epsilon_{ijk}$$

$$\begin{aligned} i &= \text{5-year maternal age group} \in \{15\text{-}19, 20\text{-}24, \ldots, 45\text{-}49\} \\ j &= \text{country} \\ k &= \text{survey} \\ P(\ldots) &= \text{parity (average CEB) for specified maternal age group} \\ CD_i &= \text{total dead children from maternal age group } i \\ CEB_i &= \text{total children ever born from maternal age group } i \end{aligned}$$

You will calculate the following quantities from your data, to which you will then apply the coefficients from the model. Country-specific random effects (U_{ij}) are also provided:

$$\frac{CD_{ijk}}{CEB_{ijk}}, \text{logit}\left(\frac{CD_{ijk}}{CEB_{ijk}}\right), CEB_{ijk}, \frac{P(15-19)}{P(20-24)}, \frac{P(20-24)}{P(25-29)}$$

All coefficients vary by maternal age group, as indicated by *i*, and the random effects also vary by country, as indicated by *j*. After obtaining the aforementioned items, you can calculate $reftime_{ijk}$ and $\text{logit}(5q0)_{ijk}$ (to eventually get ${}_5q_0$).

Familiarize yourself with the MAC datasets

Familiarize yourself with the datasets that you'll be using throughout this exercise. As a reminder, you can do this using the following commands, where `file` is replaced by one of the file names listed below. (Don't forget that the Data Browser—the window that pops up after typing `browse`—must be closed in order to type commands into the command line. Also, if Stata returns a prompt of "more…" just type any key to continue).

```
> use "file.dta", clear
> browse
> codebook
> codebook variable
> tab variable
```

Here's a list of files to look at:

a. `"MAC - 5q0 coefficients.dta"`
b. `"MAC - random effects.dta"`
c. `"MAC - reftime coefficients.dta"`

Prepare data to apply MAC method

1. Open the microdata you prepared in the last section.

```
> use "microdata modified.dta", clear
```

2. Create the following 5-year maternal age groups: 15-19, 20-24, 25-29, 30-34, 35-39, 40-44, and 45-49. Let's first initialize the variable for age group:

```
> generate agegroup = ""
```

Now let's assign an age group to each mother:

```
> replace agegroup = "15-19" if age >= 15 & age < 20
> replace agegroup = "20-24" if age >= 20 & age < 25
> replace agegroup = "25-29" if age >= 25 & age < 30
> replace agegroup = "30-34" if age >= 30 & age < 35
> replace agegroup = "35-39" if age >= 35 & age < 40
> replace agegroup = "40-44" if age >= 40 & age < 45
> replace agegroup = "45-49" if age >= 45 & age < 50
```

3. Incorporate sample weights into the data before aggregating it.

```
> generate n = 1 * wtper
> replace ceb = ceb * wtper
> replace cd = cd * wtper
```

4. Aggregate microdata by the 5-year maternal age group[1].

```
> collapse (sum) n ceb cd, by(iso3 svdate agegroup)
```

Prepare the data to apply the coefficients. The following equation will be helpful:

$$\text{logit}(p) = \log\left(\frac{p}{1-p}\right)$$

```
> generate cdceb = cd/ceb
> generate logitcdceb = log(cdceb/(1-cdceb))
> generate parity = ceb/n
> generate parityratio1 = parity[1]/parity[2]
> generate parityratio2 = parity[2]/parity[3]
> drop ceb
> rename parity ceb
```

Apply the MAC method

1. Merge the newly prepared dataset with the dataset containing coefficients to calculate ${}_5q_0$.

```
> merge agegroup using "MAC - 5q0 coefficients.dta", sort
```

The above line tells Stata to match the corresponding coefficients to each age group and to keep your data if the observations from the coefficient dataset don't match up with your dataset.

2. Check if the datasets merged correctly.

```
> tab _merge
```

The datasets did not merge correctly if you see anything besides "3" as a value for _merge.

[1] Note that you lose the region variable when you do this collapse. This is okay. The Cohort-derived methods do not require the region variable. It will become important when you apply the Period-derived methods.

3. If everything merged correctly, you can drop the `_merge` variable.

```
> drop _merge
```

4. Now merge in the random effects dataset.

```
> merge iso3 agegroup using “MAC - random effects.dta”, sort nokeep
> tab _merge
```

This step requires extra attention. If the datasets didn't merge correctly (i.e. if you see values of "1" for _merge instead of "3"), it means that your country doesn't belong to the list of countries with a random effect. If this is the case, you should replace the `random_effect` variable with zeroes:

```
> replace random_effect = 0
```

Drop the `_merge` variable.

```
> drop _merge
```

5. Calculate the logit of ${}_5q_0$ using your prepared dataset and the merged coefficients.

```
> generate logit_5q0 = coef_intercept + random_effect + coef_logitcdceb *
  logitcdceb + coef_ceb * ceb + coef_parityratio1 * parityratio1 +
  coef_parityratio2 * parityratio2
```

6. Take the inverse logit of logit(${}_5q_0$) to obtain ${}_5q_0$. The inverse logit function is as follows:

$$\text{invlogit}(x) = \frac{e^x}{1+e^x}$$

```
> generate v5q0 = exp(logit_5q0)/(1+exp(logit_5q0))
```

The convention is to look at ${}_5q_0$ in units of deaths per 1000 children.

```
> replace v5q0 = v5q0*1000
```

7. Drop unnecessary variables.

```
> drop coef* random_effect logit_5q0
```

8. Repeat steps 1 through 3 to merge in the coefficients for calculating reference time.

```
> merge agegroup using "MAC - reftime coefficients.dta", sort
```

Check if the datasets merged correctly. You should only see values of "3" for _merge.

```
> tab _merge
```

Drop the `_merge` variable if there were no problems with the merging.

```
> drop _merge
```

9. Calculate reference time.

```
> generate reftime = coef_intercept + coef_cdceb * cdceb + coef_ceb * ceb +
  coef_parityratio1 * parityratio1 + coef_parityratio2 * parityratio2
```

Reference time provides the number of years prior to the survey that the estimate pertains to. Since you have the year of your survey or census, you can calculate the exact year.

```
> generate year = svdate - reftime
```

10. Congratulations! You have completed the MAC method. Add a `method` variable to indicate that these numbers were produced using the MAC method, keep the relevant variables, and save the results to calculate the Combined Method later.

```
> generate method = "MAC"
> keep iso3 svdate method agegroup reftime year v5q0
> order iso3 svdate method agegroup reftime year v5q0
> save "MAC results.dta", replace
```

11. Lastly, take a look at these numbers on a graph.

```
> scatter v5q0 year, connect(l) ytitle("5q0 (per 1000)") title("country") sort
```

Maternal Age Period-derived Method (MAP)

Quick review

The Period-derived Methods use distributions of child birthdays and death days for different categories of mothers, stratified by maternal information (region, age, and number of children ever born/dead children). These distributions are used to find the expected number of children ever born and dead in every year prior to the survey (up to 25 years) for a mother in each particular strata. By applying these distributions to each mother in each strata, and then summing across all strata, you will generate an expected number of CEB and CD for each year prior to the survey. Then, you can find the ratio of dead children to children ever born for every year.

Here is the formula quantifying the MAP method:

$$\text{logit}\left(5q0_{tjk}\right) = \beta_t^0 + U_{tj} + \beta_t^1 \text{logit}\left(\frac{CD_{tjk}}{CEB_{tjk}}\right) + \epsilon_{tjk}$$

$$\begin{aligned} t &= \text{index of calendar time} \in [0, 24] \\ j &= \text{country} \\ k &= \text{survey} \\ CD_{tjk} &= \text{total dead children in time bin } t \\ CEB_{tjk} &= \text{total children ever born in time bin } t \end{aligned}$$

A separate model (unique set of coefficients) was developed for each year prior to the survey (up to 25 years), as indicated by *t*, and the random effect also varies by country, as indicated by *j*. Only

$$\text{logit}\left(\frac{CD_{tjk}}{CEB_{tjk}}\right)$$

will be calculated by you (not as simple as the MAC method—it involves a lot of work!) and the rest (U_{tj} and β_t) are coefficients that will be provided to you. After getting those pieces of information, you can calculate $\text{logit}(5q0)_{tjk}$ (to eventually get ${}_5q_0$) for every year prior to the survey.

Familiarize yourself with the MAP datasets

Again, familiarize yourself with the datasets that you'll be using throughout this exercise. As a reminder, you can do this using the following commands.

```
> use "file.dta", clear
> browse
> codebook
> codebook variable
> tab variable
```

Here's a list of files to look at:

a. "MAP – distribution of birthdays.dta"
b. "MAP – distribution of death days.dta"
c. "MAP - 5q0 coefficients.dta"
d. "MAP – random effects.dta"

It may be helpful to visualize part of the dataset: for dataset (a),

```
> use "MAP – distribution of birthdays.dta", clear
> preserve
> reshape long t_, i(region agegroup ceb) j(reftime)
> rename t_ prob_ceb
> graph bar prob_ceb if region == "ASIA" & agegroup == "30-31" & ceb == 8,
  over(reftime, reverse) b2title("years prior to survey")
> restore
```

and for dataset (b),

```
> use "MAP – distribution of death days.dta", clear
> preserve
> reshape long t_, i(region agegroup cd) j(reftime)
> rename t_ prob_cd
> graph bar prob_cd if region == "ASIA" & agegroup == "30-31" & cd == 4,
  over(reftime, reverse) b2title("years prior to survey")
> restore
```

These are the distributions of births and deaths over time for categories of maternal age (e.g. 30-31) and number of children born (from dataset a) and died (from dataset b).

Prepare data: Create age groups

1. Open the microdata you prepared.

```
> use "microdata modified.dta", clear
```

2. Create 2-year maternal age groups, except for the first age group of 15-17, e.g. 15-17, 18-19, 20-21, …., 46-47, 48-49. Since there are many more age groups as compared to the seven 5-year age groups in the MAC method, here's a trick to quickly create 2-year age groups.

```
> generate start_age = floor(age/2)*2
> generate end_age = start_age + 1
> replace start_age = 15 if age >= 15 & age <= 17
> replace end_age = 17 if age >= 15 & age <= 17
> generate agegroup = string(start_age) + "-" + string(end_age)
> drop start_age end_age
> list age agegroup in 1/25
```

3. The dataset you have just created will be used twice to obtain the expected birthdays and death days of each mother's children, so let's save it.

```
> save MAP_microdata, replace
```

Prepare data: Obtain the expected number of children ever born for each year prior to the survey date

4. Drop unnecessary information for this section.

```
> drop if ceb == 0
> drop cd
```

5. Aggregate microdata by 2-year maternal age group and number of children ever born.

```
> collapse (sum) wtper, by(iso3 svdate region agegroup ceb)
```

6. Merge the dataset with the probabilities of a child having a birthday each year prior to the survey date.

```
> merge region agegroup ceb using "MAP – distribution of birthdays.dta", sort
  nokeep
```

Check if the datasets merged correctly. All values of _merge should be "2" or "3". There should be no values of "1".

```
> tab _merge
```

If the datasets merged correctly,

```
> drop _merge
```

7. With the probabilities that were merged in the previous step[2], obtain the expected number of children that were born every year prior to the survey date for each group of mothers. Also, scroll through the dataset to conceptualize what is happening in this step. First, for each group of mothers,

```
> forvalues year = 0/24 {
>   generate tm_`year' = wtper * ceb * t_`year'
> }
> browse iso3-ceb_group t_3 tm_3
```

At the browse command, you will see:
- `t_3`: probability of a child being born to a single mother in year 3 prior to the survey for each specific age/CEB group
- `tm_3`: total expected number of children born to all mothers in year 3 prior to the survey for each age/CEB group

Then, for all mothers in the census, sum up the expected children born and died in each year prior to the survey (summing across age groups):

```
> collapse (sum) tm*, by(iso3 svdate)
> browse
```

8. Reshape the data into the correct format to apply the coefficients later.

```
> reshape long tm_, i(iso3 svdate) j(reftime)
> rename tm_ ceb
> replace reftime = reftime + 0.5
```

Look at the dataset that you created. For each year prior to the survey, you will have an expected value of the total number of children born in that year.

```
> browse
```

9. Save the file to be merged with the similar dataset on the number of expected dead children.

```
> save MAP_birthdays, replace
```

Prepare data: Obtain the expected number of dead children for each year prior to the survey date

Note: this process will be very similar to the last section.

10. Reopen the microdata that you prepared earlier.

```
> use MAP_microdata, clear
```

[2] Recall that each t_*year* variable contains the probability that a woman in a specific age group, CEB, and region would have had a child during that particular year.

11. Drop unnecessary information for this section.

```
> drop if cd == 0
> drop ceb
```

12. Aggregate microdata by maternal age group and number of dead children.

```
> collapse (sum) wtper, by(iso3 svdate region agegroup cd)
```

13. Merge the dataset with the probabilities of a child having a death day each year prior to the survey date.

```
> merge region agegroup cd using "MAP – distribution of death days.dta", sort
  nokeep
```

Check if the datasets merged correctly. All values of the _merge variable should be “3”.

```
> tab _merge
```

If the datasets merged correctly,

```
> drop _merge
```

14. With the probabilities that were merged in the previous step[3], obtain the expected number of children that died every year prior to the survey date for each group of mothers. Also, scroll through the dataset to conceptualize what is happening in this step. First, for each group of mothers,

```
> forvalues year = 0/24 {
>   generate tm_`year' = wtper * cd * t_`year'
> }
> browse iso3-region t_10 tm_10
```

then, for all mothers in the census,

```
> collapse (sum) tm*, by(iso3 svdate)
> browse
```

15. Reshape the data into the correct format to apply the coefficients later.

```
> reshape long tm_, i(iso3 svdate) j(reftime)
> rename tm_ cd
> replace reftime = reftime + 0.5
```

Look at the dataset you just created.

---

[3] Recall that each t_*year* variable contains the probability that a child borne by a woman in a specific age group, CD, and region would have died during that particular year.

```
> browse
```

16. Merge this dataset with the previous dataset (with the number of expected children ever born).

```
> merge iso3 svdate reftime using MAP_birthdays, sort
```

17. Check to make sure the datasets merged correctly (values of "3"). If so, drop the `_merge` variable.

```
> tab _merge
> drop _merge
```

Look at your newly created dataset.

```
> browse
```

Prepare data to apply MAP model

18. Prepare the data to apply the coefficients. Here's the logit function again:

$$\text{logit}(p) = \log\left(\frac{p}{1-p}\right)$$

```
> generate cdceb = cd/ceb
> generate logitcdceb = log(cdceb/(1-cdceb))
```

Apply the MAP method

1. Merge the newly prepared dataset with the dataset containing coefficients to calculate ${}_5q_0$.

```
> merge reftime using "MAP - 5q0 coefficients.dta", sort nokeep
```

The above line tells Stata to merge according to reference time.

2. Check if the datasets merged correctly. You should only see values of "3" for the `_merge` variable

```
> tab _merge
```

3. If everything merged correctly, drop the `_merge` variable.

```
> drop _merge
```

4. Merge in the country-level random effects.

```
> merge iso3 reftime using "MAP - random effects.dta", sort nokeep
> tab _merge
```

Reminder: If the datasets didn't merge correctly (`_merge` values of "1"), it means that your country doesn't belong to the subset of countries that have a random effect. If this is the case, you should replace the `random_effect` variable with zeroes.

```
> replace random_effect = 0
```

Drop the `_merge` variable after your diagnosis.

```
> drop _merge
```

5. Calculate the logit of ${}_5q_0$.

```
> generate logit_5q0 = coef_intercept + random_effect + coef_logitcdceb *
  logitcdceb
```

6. Take the inverse logit of logit(${}_5q_0$) to obtain ${}_5q_0$. Here's the inverse logit function again:

$$\text{invlogit}(x) = \frac{e^x}{1 + e^x}$$

```
> generate v5q0 = exp(logit_5q0)/(1+exp(logit_5q0))
```

The tradition is to look at ${}_5q_0$ in units of deaths per 1000 children.

```
> replace v5q0 = v5q0*1000
```

7. Calculate the year that the estimate pertains to.

```
> generate year = svdate - reftime
```

8. Again, add a `method` variable to indicate that these numbers were produced by the MAP method, keep the relevant variables, and save the results to calculate the Combined Method later.

```
> generate method = "MAP"
> keep iso3 svdate method reftime year v5q0
> order iso3 svdate method reftime year v5q0
> save "MAP results.dta", replace
```

9. Look at the results on a graph, and compare to the MAC estimates.

```
> append using "MAC results.dta"
> graph twoway (scatter v5q0 year if method == "MAP", connect(l) sort) (scatter
  v5q0 year if method == "MAC", connect(l) sort), ytitle("5q0 (per 1000)")
  legend(label(1 "MAP") label(2 "MAC")) title("country")
```

10. Let's clean up unwanted files from the hard drive.

```
> erase “MAP_microdata.dta”
> erase “MAP_birthdays.dta”
```

Time Since First Birth Methods (TFBC and TFBP)

The Time Since First Birth methods are essentially the same as the Maternal Age Cohort methods—with the Time Since First Birth Cohort-derived (TFBC) method being similar to the Maternal Age Cohort-derived (MAC) method, and the Time Since First Birth Period-derived (TFBP) method being similar to the Maternal Age Cohort-derived (MAP) method—and by "similar" or "same", we mean in both concept and code.

Most surveys and censuses are not designed to have time since first birth information, as with the census microdata taken from IPUMS. If your survey doesn't have `timefb` (time since first birth variable), you can still move on with the analysis and use the MAC and MAP results to apply the Combined Method; the Combined Method doesn't require TFBC and/or TFBP to be applied.

If you have the fortune of having `timefb` in your microdata, you'll need to adapt the code outlined in the MAC and MAP sections by just simply replacing the appropriate parts of the code, e.g. `age` to `timefb`, `agegroup` to `timefbgroup`, `MAC` to `TFBC`, `MAP` to `TFBP`. You will also need to modify the category cutpoints for `timefbgroup` to be 0-4, 5-9, 10-14, 15-19, 20-24, 25-29 and 30-34 for the cohort-derived method and 0-1, 2-3, 4-5, …, 30-31, and 32-34 for the period-derived method.

Applying the Models to Aggregated Data

If your data set only consists of aggregated data, you can still apply the MAC method (and TFBC method if you have data aggregated by time since first birth groups as well). Skip the steps of formatting the data and go directly to merging the model coefficients in with your dataset. Remember to rename all the variables in your dataset to be consistent with these instructions in order for the model to be applied appropriately.

You can also apply the period methods to aggregated data if the data have been aggregated in the format required (2-year groupings) for the MAP and/or TFBP methods.

Combined Method

Quick review

The Combined Method averages the estimates from MAC, MAP, TFBC, and TFBP while taking into account nonlinear trends using a statistical method called LOESS. Each point is weighted by the inverse of the number of points generated by the method that produced it. For example, the MAC method usually produces 7 estimates of under-five mortality across time so each estimate gets a weighting of 1/7. Similarly, the MAP method usually produces 25 estimates across time, so each estimate gets a weighting of 1/25. In this way, the Combined Method weights each series of estimates equally. Unfortunately, Stata doesn't allow for weighting in the LOESS procedure so you will have to port to R for this session.

Apply the Combined Method

1. Close Stata to get ready to move into R. Remember to close your log file before closing Stata!

```
> log close
```

2. Open an R session and initialize the session (steps outlined in "Brief Introduction to R"). Remember to change the directory with `setwd`. The directory that you set will be where the MAC and MAP results are located and where your output will be located.

3. Since you'll be reading in data files produced by Stata, you need to load a package called "foreign" in order to do this in R.

```
> library(foreign)
```

4. Now you can load in the MAC and MAP results. Take a look at them while you're at it.

```
> mac = read.dta("MAC results.dta")
> mac
> map = read.dta("MAP results.dta")
> map
```

5. Create weights for the MAC and MAP estimates and make sure the weights look correct.

```
> mac$weight = 1/nrow(mac)
> mac
> map$weight = 1/nrow(map)
> map
```

6. Merge the MAC and MAP results to be inputted into the LOESS procedure.

```
> input.data = merge(mac, map, all = TRUE)
```

7. Take out the MAC estimates from the 15-19 age group. Because the number of mothers in this age category is so small, the predictions from this age group can still be quite noisy, so we exclude them from the Combined Method.

```
> input.data.no15to19 = subset(input.data, agegroup != "15-19" |
  is.na(agegroup))
```

Take a look at the data and see if the code took out the group correctly.

```
> input.data.no15to19
```

8. Apply the Combined Method, i.e. fit the LOESS model on the input data.

```
> fit = loess(v5q0 ~ year, input.data.no15to19, weights = weight, span = 0.5)
```

9. Create a new object for the years that you want LOESS predictions for. Since you can't predict outside of the range that you inputted into the LOESS procedure, let's do it for every year between the minimum and maximum years of the input dataset.

```
> years.predict = seq(min(input.data.no15to19$year),
  max(input.data.no15to19$year))
> combined.method = data.frame(iso3 = unique(input.data$iso3), svdate =
  unique(input.data$svdate), method = "Combined Method", year = years.predict)
> combined.method$reftime = combined.method$svdate - combined.method$year
```

10. Predict ${}_5q_0$ values for those years.

```
> combined.method$v5q0 = predict(fit, combined.method)
```

Take a look at the data to see if the predictions look plausible.

```
> combined.method
```

11. View all the results on a graph.

```
> plot(combined.method$year, combined.method$v5q0, col = "purple", type = "l",
  lwd = 2, xlab = "year", ylab = "5q0 (in 1000)", main = "country")
> points(mac$year[mac$agegroup != "15-19"], mac$v5q0[mac$agegroup != "15-19"],
  col = "red", pch = 19)
> points(mac$year[mac$agegroup == "15-19"], mac$v5q0[mac$agegroup == "15-19"],
  col = "red")
> points(map$year, map$v5q0, col = "blue", pch = 19)
> legend("topright", legend = c("MAC", "MAP", "Combined Method"), col = c("red",
  "blue", "purple"), pch = c(19, 19, NA), lty = c(0, 0, 1), lwd = c(0, 0, 2))
```

If you like, you can save the graph by first clicking the graph window, click on "File" and then "Save as", choose the file type of your choice, name the file, and then press "Save".

12. Merge all the results (MAC, MAP and Combined Method) and then save it as both a comma separated (csv) file and a Stata data file (dta).

```
> all.results = merge(input.data, combined.method, all = TRUE)
> write.csv(all.results, file = "country results.csv", row.names = FALSE)
> write.dta(all.results, file = "country results.dta")
```

If you like, you can delete the MAC and MAP results since they were merged into the file you just outputted in the last step.

```
> file.remove("MAC results.dta")
> file.remove("MAP results.dta")
```

That's it! Congratulations, you've completed the Summary Birth History Method!

Resources

Zip file

The zip file can be found at the Institute for Health Metrics and Evaluation website: (http://www.healthmetricsandevaluation.org).

References

Rajaratnam JK, Tran LN, Murray CJL, Lopez AD. Measuring Under-Five Mortality: Validation of New Low-Cost Methods. *PLoS Medicine*, 2010.

Contacts

Please contact us if you have any questions, comments, or suggestions.

Linda N Tran l.tran@sph.uq.edu.au
Julie Knoll Rajaratnam jrajarat@uw.edu
